# Heteroallene Capture and Exchange at Functionalised Heptaphosphane Clusters

**DOI:** 10.1002/chem.202103737

**Published:** 2021-12-21

**Authors:** Bono van IJzendoorn, Inigo J. Vitorica‐Yrezabal, George F. S. Whitehead, Meera Mehta

**Affiliations:** ^1^ Department of Chemistry University of Manchester Oxford Road Manchester M13 9PL United Kingdom; ^2^ X-ray Diffraction Facility University of Manchester Oxford Road Manchester M13 9PL United Kingdom

**Keywords:** main group chemistry, polyphosphides, small molecule capture, Zintl anions

## Abstract

Despite being known for decades the chemical reactivity of homoatomic seven‐atom phosphorus clusters towards small molecules remains largely unexplored. Here, we report that neutral tris(silyl) functionalised heptaphosphane (P_7_(SiR_3_)_3_) cages are capable of heteroallene capture between the P−Si bonds of the cluster. A range of isocyanates and an isothiocyanate were investigated. In the case of isocyanates, silyl bonding at oxygen or nitrogen is regioselectively directed by the functional group on the isocyanate and substituents on the silyl moiety. Above all, we find that captured isothiocyanate molecules can be exchanged for isocyanate molecules, indicative of small molecule catch and release. Small molecule catch and release at these Zintl‐derived clusters reveals their potential application as chemical storage materials or as reusable probes.

## Graphical abstract

**Three equivalents of heteroallene** can be captured between the Si−P bonds of Zintl‐derived clusters. Silyl coordination at either the nitrogen or chalcogen of the heteroallene can be controlled by tuning the groups on the silyl moiety or functional groups at the heteroallene. It was also found that one captured small molecule can be exchanged for another, evidencing small molecule catch and release at these clusters.

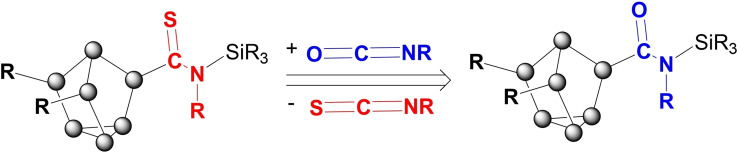

## Introduction

**Figure 1:**
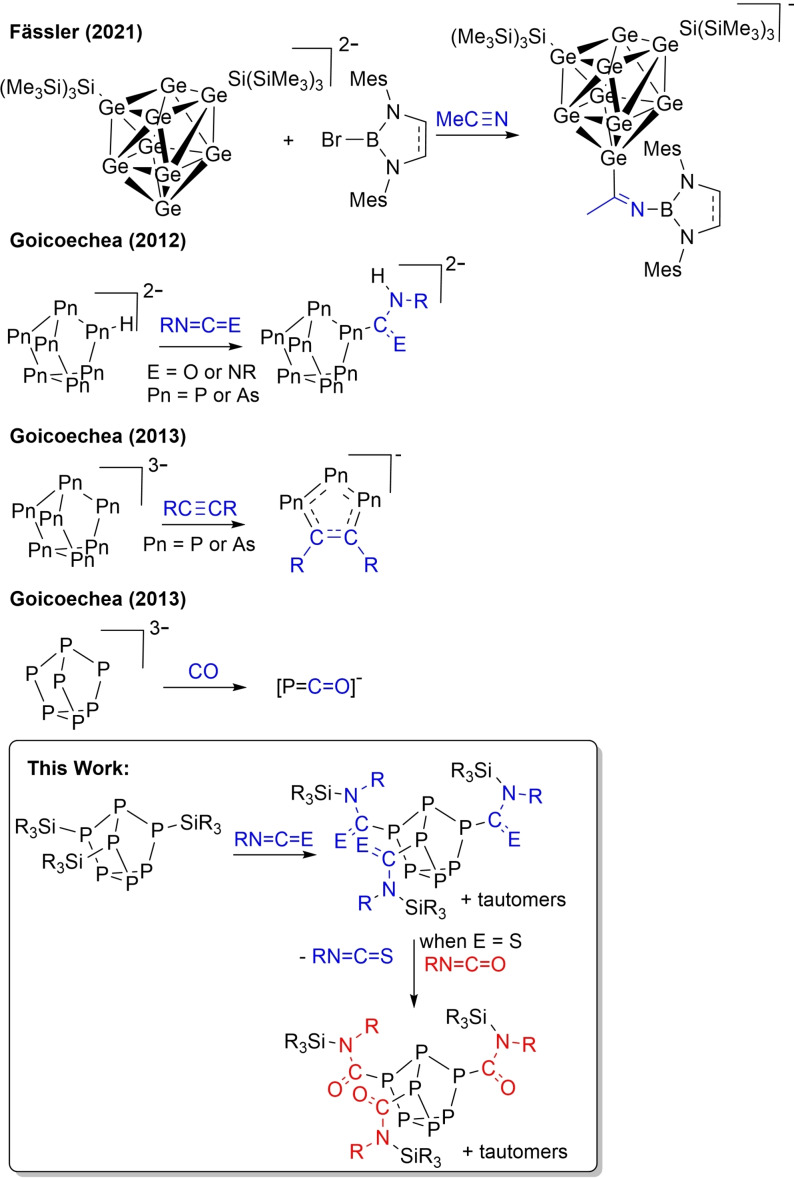
Previous examples of small molecule activations with Zintl clusters and overview of this work.

Zintl clusters, polyatomic clusters of p‐block elements, are of continued interest because of their structure, bonding, and physical properties both as solid‐state phases and as solution‐state extracted salts.^[1]^ Recent developments in the field are largely focused on the construction and isolation of large and heteroatomic clusters, salt metathesis reactions with Group 14 electrophiles, and coordination chemistry with d‐ and f‐block metals.[^1c^, ^1d^, ^1e^, ^1f^, ^1g^, ^1h^, ^1i^, ^1j^, ^1k^] For example in 2020, Weller and Goicoechea reported the coordination of [Ge_9_] to catalytically active rhodium to affect the hydrogenation of alkenes.^[2]^ While, Scheschkewitz and co‐workers reported the coordination of silicon clusters to catalytically active iridium to mediate the isomerisation of alkenes.^[3]^ In both of these reports, the Group 14 cluster plays the role of a supporting ligand. Early this year, Fässler published the capture of acetonitrile between a bromoborane and functionalised [Ge_9_] cluster (Figure 1).^[4]^ This small molecule activation is reminiscent of that observed in frustrated Lewis pair (FLP) chemistry.^[5]^

The heptaphosphide trianion, [P_7_]^3−^, is an interesting and attractive feedstock because of its structural relationship to red phosphorus.[^1h^, ^6^] Tris‐functionalised [P_7_] cages are soluble in common laboratory solvents, such as toluene, ether, dichloromethane, and tetrahydrofuran (THF). This solubility allows for rapid assessment of solution‐state reactivity. While, red phosphorus is a difficult to study heterogeneous material. [P_7_] cages can be considered molecular models, and perhaps inform what types of functionalised red phosphorus materials can be targeted for chemical storage.

Examples of small molecule activation with [P_7_] cages are limited (Figure 1). In 2012 and 2013, Goicoechea and co‐workers employed the protonated heptapnictide cage [HPn_7_]^2−^ (Pn=P, As) in hydropnictination reactions with carbodiimides and isocyanates.^[7]^ The same group also found that reactions of [Pn_7_]^3−^ (Pn=P, As) with alkynes transferred a [Pn_3_]^−^ unit to afford 1,2,3‐tripnictolides,^[8]^ while reaction with carbon monoxide transferred P^−^ to give the [PCO]^−^ anion.^[9]^

Herein, we find that three equivalents of heteroallene, namely isocyanates (RN=C=O) and an isothiocyanate (RN=C=S), related to the greenhouse gas CO_2_ (O=C=O) are captured at these cages. All three of the Si−P bonds of silyl functionalised heptaphosphanes (P_7_(SiR_3_)_3_) capture a heteroallene via either C=N or C=O bond activation. While small molecule capture into the labile Si−P bonds of simple silylphosphines (R_2_P−SiR_3_) has been previously reported,^[10]^ our results represent the first demonstration of this reactivity with Zintl‐derived main group clusters. Moreover, not only are heteroallenes captured with this class of compound but, in addition, we find that one heteroallene can be exchanged for another. Small molecule capture followed by unprecedented displacement with Zintl‐derived clusters is discovered. This type of displacement would be a key advantage when designing re‐usable chemical storage materials.

## Results and Discussion

**Scheme 1:**
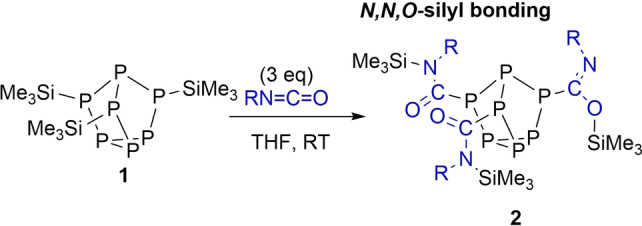
Reactions of phenyl isocyanate (R=Ph) with **1**.

**Figure 2:**
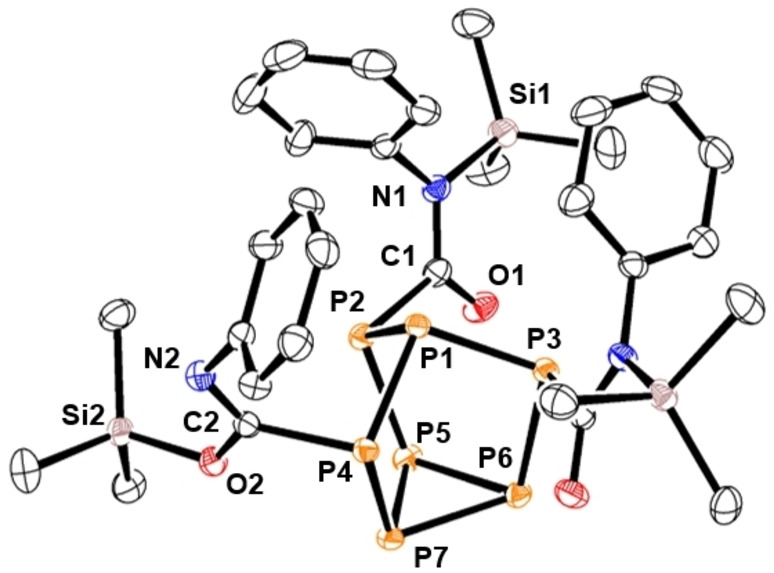
Molecular structure of 2. Anisotropic displacement ellipsoids pictured at 50 % probability. Hydrogen atoms omitted for clarity. Phosphorus: Orange; Silicon: Pink; Carbon: White; Oxygen: Red; Nitrogen: Blue. Selected bond lengths [Å]: P1−P2 2.2005(5), P1−P3 2.1873(5), P1−P4 2.1983(5), P2−P5 2.2105(5), P3−P6 2.2095(5), P4−P7 2.2097(5), P5−P6 2.2030(6), P5−P7 2.2020(5), P6−P7 2.2003(5), P2−C1 1.8903(16), C1−N1 1.358(2), C1−O1 1.2279(19), N1−Si1 1.7991(13), P4−C2 1.8795(15), C2−N2 1.266(2), C2−O2 1.3467(18), O1−Si1 1.6995(11).

First P_7_(SiMe_3_)_3_ (**1**) was prepared using literature methods,^[11]^ and reacted with three equivalents of phenyl isocyanate in THF overnight (Scheme 1). ^29^Si NMR experiments revealed complete consumption of the starting material and formation of a new product with resonances characteristic of Si−O and Si−N bond formation (see Supporting Information). X‐ray diffraction (XRD) studies authenticated the structure of **2**. The bridging phosphorus atoms of the cluster are bonded to the isocyanate carbon atoms, while two Me_3_Si units are bonded to the isocyanate nitrogen atoms and one to the oxygen atom. The single‐crystal XRD data for **2** showed C−O and C−N bond lengths of 1.347(2) and 1.266(2) Å, respectively, for the imidate moiety [RC(=NR)OR]. For the amide linkage [RC(=O)NR_2_] the C−O and C−N bond lengths average 1.225 and 1.358 Å, respectively (Figure 2). The bond lengths are consistent with single bond C−O and double bond C−N character in the imidate moiety, and vice versa in the amide moiety.

Variable temperature (VT) NMR studies and solid‐state (SS) NMR studies were undertaken to probe tautomerization between the imidate and amide moieties of **2**. ^31^P VT NMR studies between −70 to 50 °C revealed no indication of tautomerization; only **2** was observed. ^31^P SS NMR studies gave resonances too broad to be conclusive. The ^29^Si SS NMR spectrum was consistent with that collected in the solution‐state and showed no evidence of tautomerization. To investigate if the Me_3_Si‐group prefers oxygen bond formation and to force tautomerization the reaction mixture was heated at 50 °C, but only decomposition of the cage was observed.

Compound **1** was also treated with one and two equivalents of phenyl isocyanate to isolate the partially inserted products, but only formation of **2** and unreacted starting material could be detected by NMR spectroscopy and XRD studies. This suggests that tris‐insertion is preferred over mono‐ or bis‐insertion of the heteroallene.

**Scheme 2:**
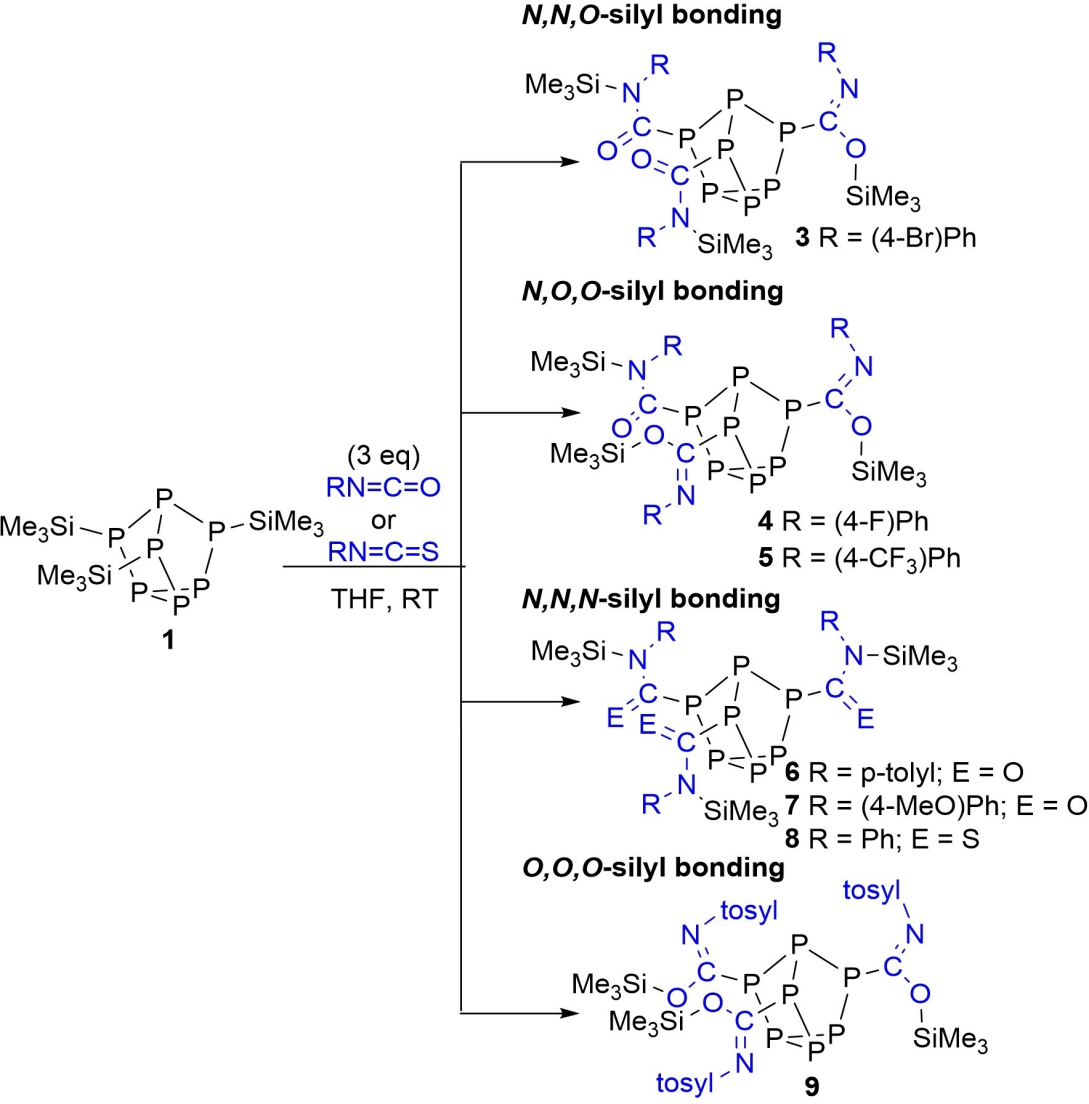
Reactions of isocyanates and an isothiocyanate with **1**.

To investigate the role of the isocyanate on silyl regioselectivity, substituted phenyl isocyanate derivatives with electron‐donating and electron‐withdrawing groups were reacted with **1**. Treatment of **1** with 4‐bromophenyl isocyanate afforded the related insertion product **3** (Scheme 2), again with *N,N,O*‐silyl bonding.

**Figure 3:**
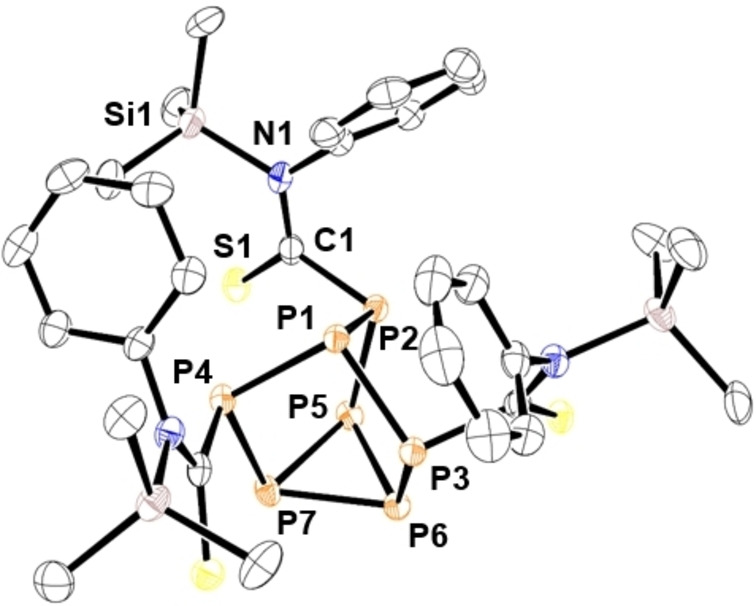
Molecular structure of **8**. Anisotropic displacement ellipsoids pictured at 50 % probability. Hydrogen atoms omitted for clarity. Phosphorus: Orange; Silicon: Pink; Carbon: White; Nitrogen: Blue; Sulfur: Yellow. Selected bond lengths [Å]: P1−P2 2.1991(8), P1−P3 2.2028(9), P1−P4 2.1983(8), P2−P5 2.2045(8), P3−P6 2.2117(8), P4−P7 2.2106(8), P5−P6 2.2098(10), P5−P7 2.2127(9), P6−P7 2.2159(9), P2−C1 1.860(3), C1−N1 1.362(3), C1−S1 1.660(3), N1−Si1 1.814(2).

**Figure 4:**
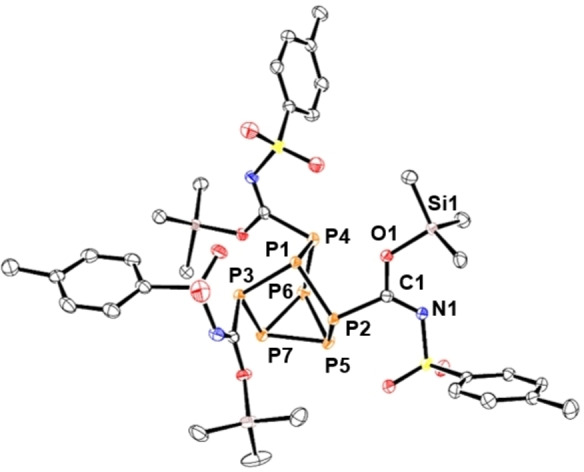
Molecular structure of **9**. Anisotropic displacement ellipsoids pictured at 50 % probability. Hydrogen atoms omitted for clarity. Phosphorus: Orange; Silicon: Pink; Carbon: White; Oxygen: Red; Nitrogen: Blue; Sulfur: Yellow. Selected bond lengths [Å]: P1−P2 2.2038(7), P1−P3 2.1823(7), P1−P4 2.1994(7), P2−P5 2.2282(7), P3−P6 2.2230(7), P4−P7 2.2183(7), P5−P6 2.2125(7), P5−P7 2.1947(8), P6−P7 2.2069(8), P2−C1 1.8903(19), C1−N1 1.285(2), C1−O1 1.323(2), O1−Si1 1.7214(14).

In contrast, treatment of **1** with 4‐fluorophenyl isocyanate or 4‐(trifluoromethyl)phenyl isocyanate showed ^1^H and ^29^Si NMR spectra consistent with the formation of **4** or **5**, respectively, which contains *N,O,O*‐silyl bonding. Reaction of **1** with *p*‐tolyl isocyanate or 4‐(methoxy)phenyl isocyanate resulted in formation of compounds **6** or **7**, correspondingly. NMR and XRD studies into **6** and **7** revealed that the Me_3_Si units selectively bonded to the isocyanate nitrogen atoms to give three amide groups. To eliminate the possibility of Si−O bond formation, **1** was reacted with phenyl isothiocyanate and found to give compound **8** (Figure 3). XRD data of compounds **6**–**8** were all consistent with localised single C−N bonds and double C−E bonds, where E is either O or S (**6**: C−N 1.360(av) Å, C−O 1.217(av) Å; **7**: C−N 1.356(av) Å, C−O 1.212(av) Å; **8**: C−N 1.349(av) Å, C−S 1.662(av) Å). Finally, *p*‐toluenesulfonyl isocyanate (*p*‐toluenesulfonyl=tosyl) was found to insert into the P−Si bonds of **1** with selective Si−O bond formation to give three imidate groups (Figure 4).

**Figure 5:**
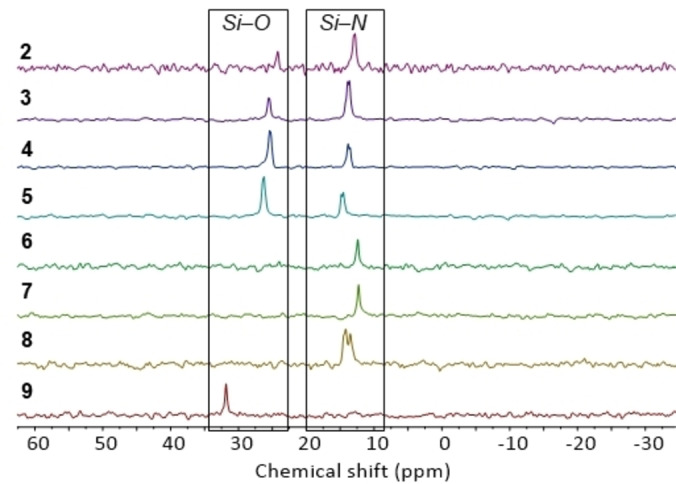
^29^Si DEPT90 NMR spectra (THF‐d_8_) of **2**–**9**.

Formation of insertion products **2**–**9** could be monitored using ^29^Si NMR spectroscopy with the resonance at 7.16 ppm (d, ^1^ *J*_PSi_=43 Hz) diminishing in intensity and new resonances being observed between 12 and 32 ppm. The silyl regioselectivity upon insertion was determined using ^1^H and ^29^Si NMR spectroscopy, Figure 5 shows the diagnostic ^29^Si NMR signals. In the case of **2**, **6**–**9** silyl regioselectivity was further validated by single‐crystal XRD studies. As electron‐withdrawing groups are introduced on the isocyanate aromatic ring, upon insertion, the silyl group prefers coordination to the oxygen atom.

**Scheme 3:**
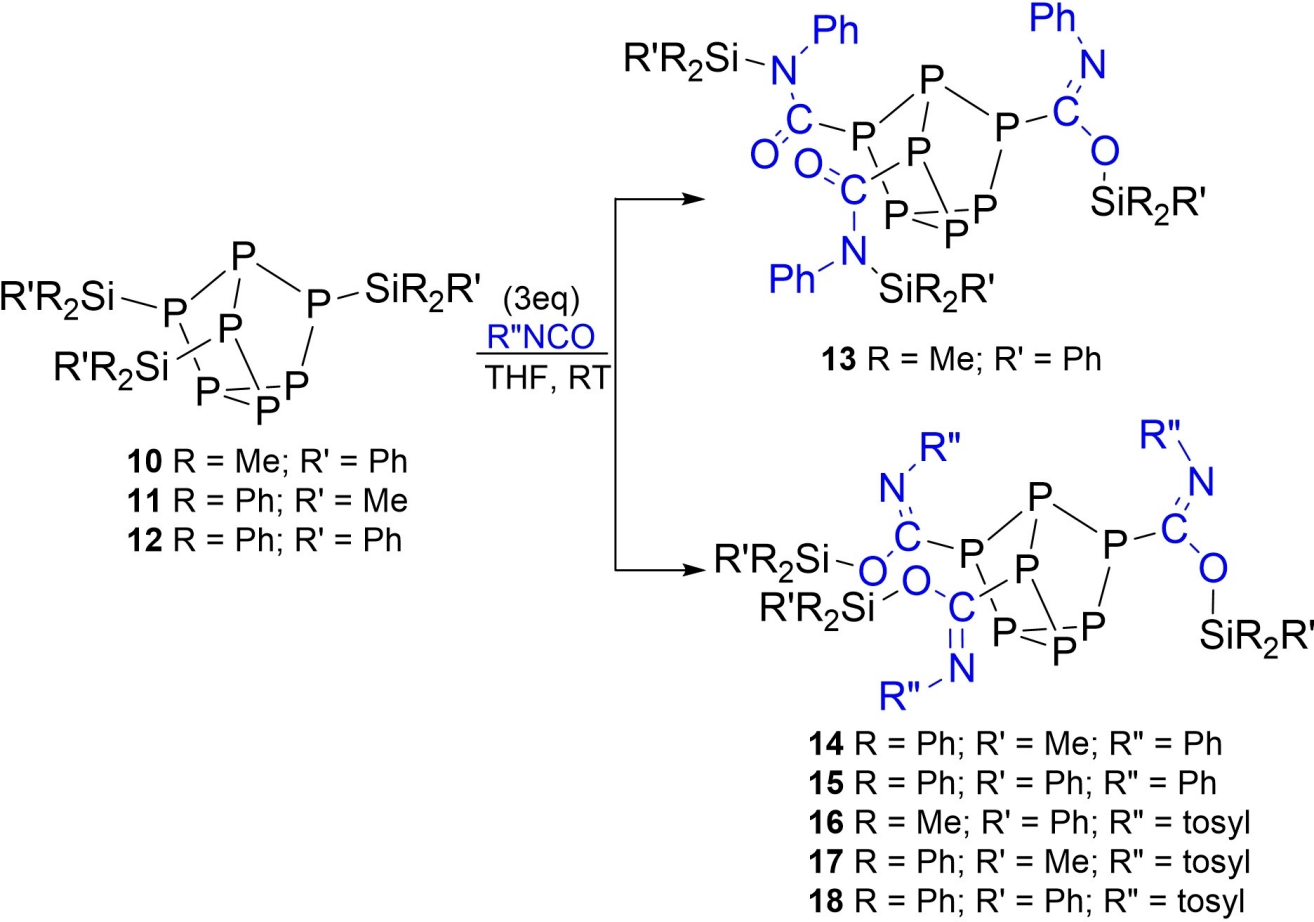
Reaction of isocyanates with compounds **10**–**12**.

**Figure 6:**
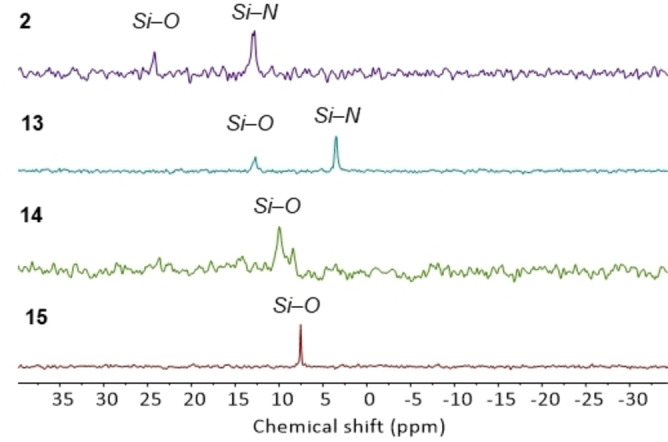
^29^Si DEPT90 NMR spectra (THF‐d_8_) of **2**, **13**–**15**.

**Figure 7:**
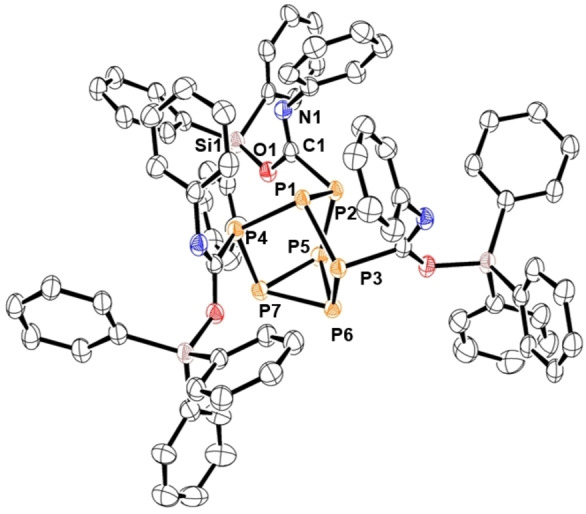
Molecular structure of **15**. Anisotropic displacement ellipsoids pictured at 50 % probability. Hydrogen atoms omitted for clarity. Phosphorus: Orange; Silicon: Pink; Carbon: White; Oxygen: Red; Nitrogen: Blue. Selected bond lengths [Å]: P1−P2 2.1888(14), P1−P3 2.1888(14), P1−P4 2.1889(14), P2−P5 2.2180(18), P3−P6 2.2180(18), P4−P7 2.2180(18), P5−P6 2.2058(17), P5−P7 2.2058(17), P6−P7 2.2058(17), P2−C1 1.869(4), C1−N1 1.257(6), C1−O1 1.353(5), N1−Si1 1.693(3).

To probe the effect of the silyl group on isocyanate insertion compounds **10**–**12** were prepared where methyl groups on Si are sequentially replaced with phenyl groups (Scheme 3). Treatment of **10** with three equivalents of phenyl isocyanate gave product **13** with *N,N,O*‐bonding of the Me_2_PhSi units. In contrast, treatment of **11** and **12** with three equivalents of phenyl isocyanate gave products **14** and **15**, respectively. Compounds **14** and **15** were determined to have *O,O,O*‐silyl bonding in the solution‐state from NMR (Figure 6), and also the solid‐state structure of **15** (Figure 7). When **10**–**12** were reacted with tosyl isocyanate, products **16**–**18** were formed, which also have *O,O,O*‐silyl bonding.

Notably, although compounds **1** and **10**–**12** all showed reactivity towards phenyl isocyanate and tosyl isocyanate, reaction times significantly differed, with **12** requiring nearly 30 days to go to completion. DFT investigations revealed that the fluoride ion affinity (FIA) of the silyl group increases in the order **1**<**10**<**11**∼**12**. Consistent with Lewis acidity at the Si centre increasing as methyl groups are replaced with phenyl groups. [P_7_] bonded silyl groups also showed smaller FIA values when compared to traditional Lewis acids, such as B(C_6_F_5_)_3_, SbF_5_, and [Me_3_Si]^+^ (Table 1). Comparison of the ^29^Si NMR resonances showed that as methyl groups on the silane are replaced with phenyl groups there is an upfield shift and an increase in the ^1^ *J*_PSi_ coupling (Table 1). This is consistent with an increase in P‐Si bond strength in the order **1**<**10**<**11**<**12** and the observed increase in reaction times necessary to insert within those bonds. Comparison of the Si−P bond distances of **1**, **10**–**12**, determined by single‐crystal XRD studies, showed no appreciable change and were all within the error of measurement.^[12]^

**Table 1:** Summary of FIA Data and ^29^Si DEPT90 NMR Data for Select Lewis Acids and Compounds **1**, **10**–**12**.

Compound	FIA [kJ/mol]^[a]^	^29^Si DEPT90 NMR δ [ppm]
B(C_6_F_5_)_3_	444	–
SbF_5_	489	–
[Me_3_Si]^+^	948	–
**1**	324	7.16 (d, ^1^ *J*_PSi_=43 Hz)
**10**	335	0.76 (d, ^1^ *J*_PSi_=45 Hz)
**11**	349	−4.33 (d, ^1^ *J*_PSi_=49 Hz)
**12**	353	−7.73 (d, ^1^ *J*_PSi_=53 Hz)

[a] FIAs were computed BP86/SV(p) level of theory, following literature method reported by Greb.^[13]^

As methyl groups are replaced with phenyl groups on the silyl unit preferential oxygen bonding is observed. This preference can be attributed to a combination of electronic and steric effects. The increased Lewis acidity (FIA data, Table 1) and steric demand at the silyl group drives toward Si−O bond formation.

The ^31^P NMR spectra for compounds **9** and **16**–**18** showed three resonances for the apical, bridging, and basal phosphorus atoms confirming the presence of a single isomer in the solution‐state. In contrast, the ^31^P NMR spectra for compounds **2**–**8** and **13**–**15** revealed multiple isomers in the solution‐state and are further discussed in the Supporting Information. Compositional purity for compounds **2**–**11** and **13**–**18** was further confirmed by elemental analysis and/or mass spectrometry.

**Scheme 4:**
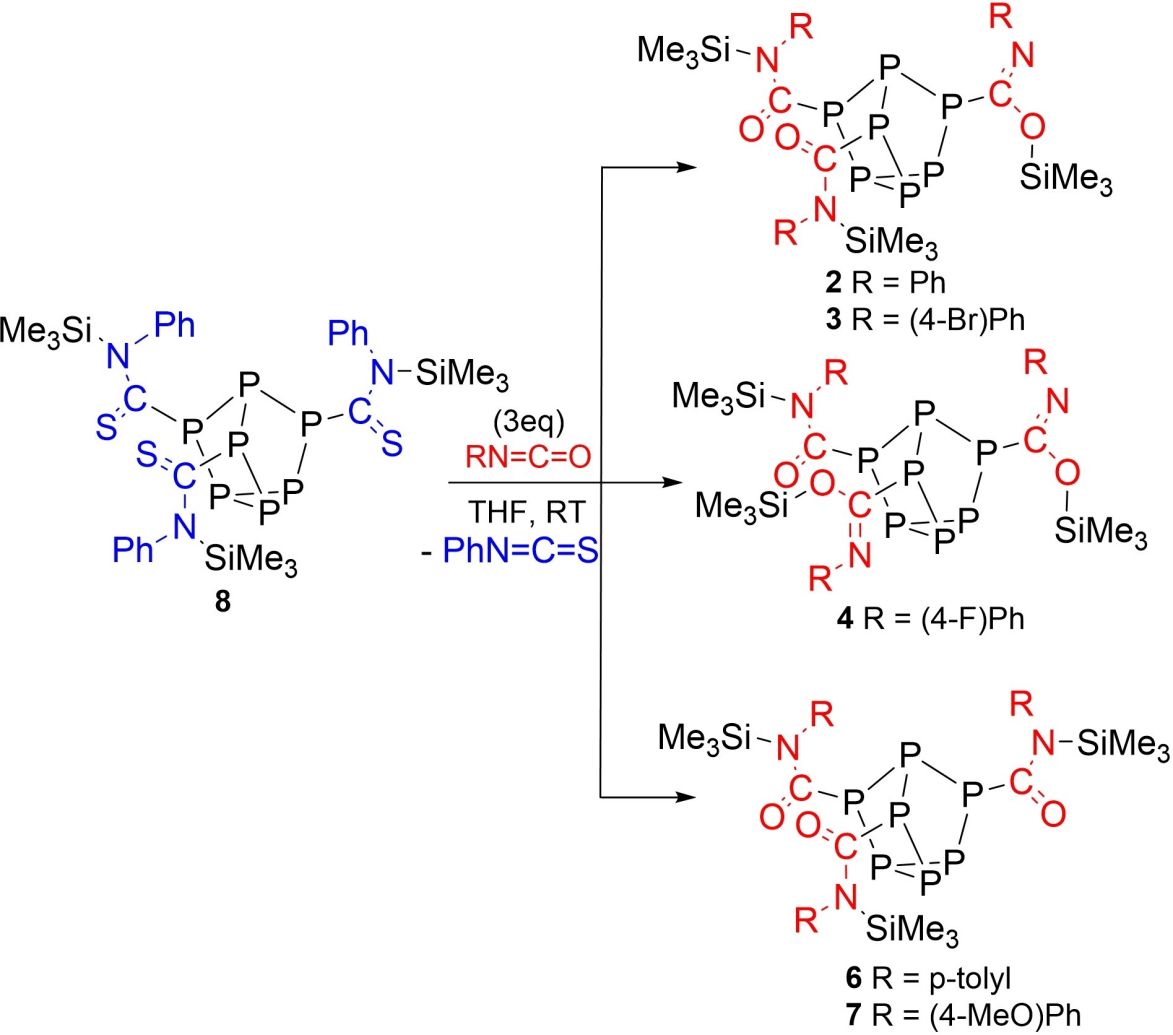
Displacement of captured isothiocyanates of **8** for isocyanates.

To probe reversible binding of the captured heteroallenes exchange reactions were undertaken. It was found that when compound **8** was allowed to react with three equivalents of phenyl isocyanate complete conversion to **2** was achieved after 7 h at 50 °C, observed by ^31^P NMR spectroscopy (Scheme 4). From this reaction mixture crystals suitable for XRD studies of **2** could also be obtained and are the same as those obtained from its independent synthesis. In a similar fashion, the isothiocyanates captured in **8** could be exchanged for 4‐fluorophenyl isocyanate, 4‐bromophenyl isocyanate, 4‐(methoxy)phenyl isocyanate, and p‐tolyl isocyanate (Scheme 4). From the latter two reactions crystals of **6** and **7** could be obtained and XRD confirmed the structures. In contrast, treatment of **8** with three equivalents of 4‐(trifluoromethyl)phenyl isocyanate under similar conditions showed no reaction. It was also found that the inserted phenyl isocyanates of **2** showed no exchange with phenyl isothiocyanate. Additional efforts to exchange the capture isocyanates of **2**, **3**, and **5** with another isocyanate required long reaction times at elevated temperatures and led to significant cage decomposition.

**Figure 8:**
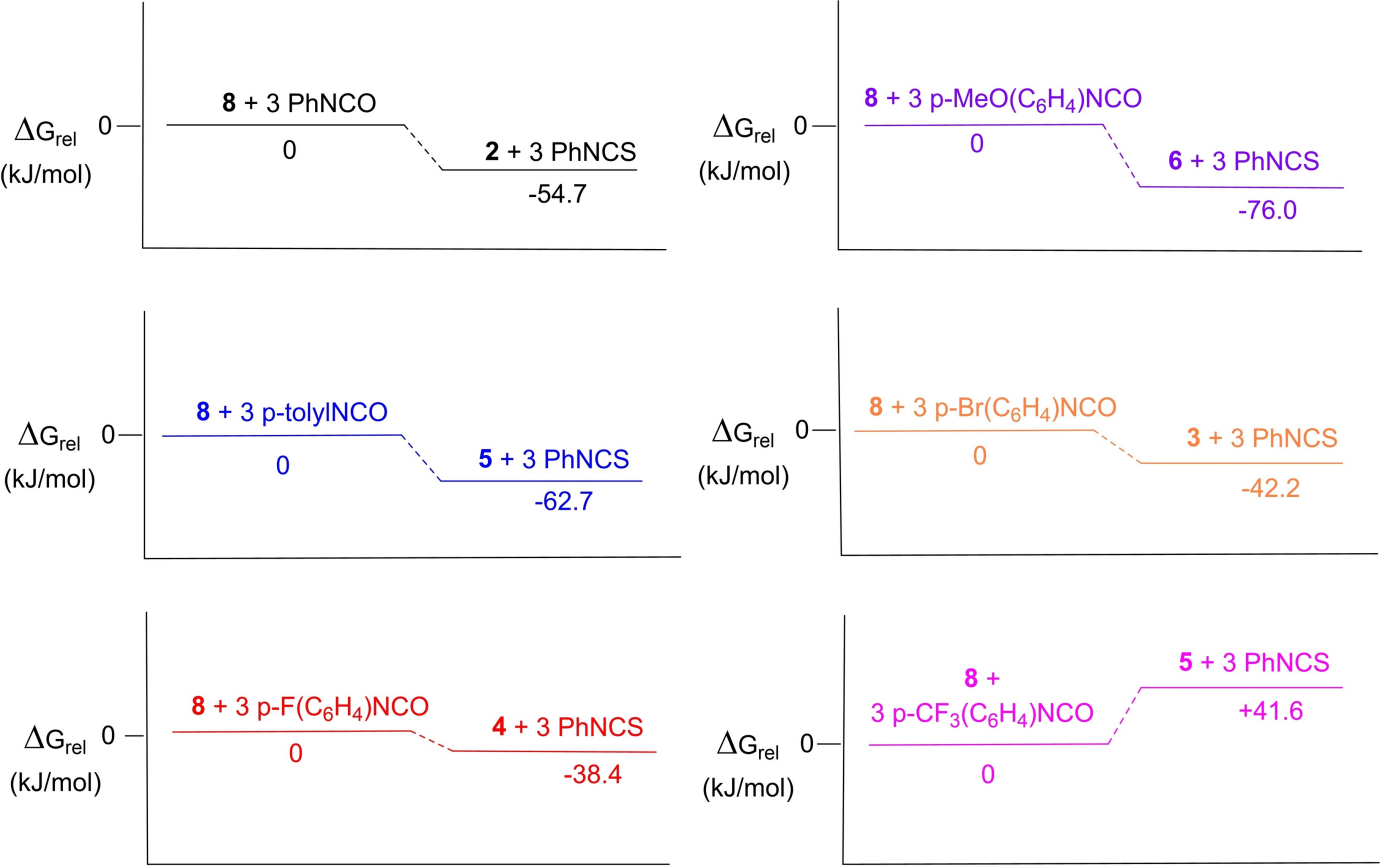
Computed energies for exchange of isothiocyanates captured at **8** for isocyanates. Preformed at PBE1PBE/6‐311G(d,p) level of theory.

DFT investigations were undertaken to understand the thermodynamics of phenyl isothiocyanate displacement in **8** (Figure 8). By comparing the calculated Gibbs energies It was found that exchange of the captured phenyl isothiocyanate with phenyl isocyanate, 4‐(methoxy)phenyl isocyanate, p‐tolyl isocyanate, 4‐bromophenyl isocyanate, and 4‐fluorophenyl isocyanate, is thermodynamically exergonic by −54.7, −76.0, −62.7, −42.2, and −38.4 kJ/mol, respectively. Exchange with 4‐(trifluoromethyl)phenyl isocyanate is thermodynamically endergonic by 41.6 kJ/mol. These computational findings show that the exchange is more favourable with the introduction of electron‐donating groups on the isocyanate, and were consistent with the experimental exchange observations.

## Conclusion

In conclusion, we report on the capture of isocyanates and an isothiocyanate between the Si−P bonds of tris(silyl) functionalised heptaphosphide clusters. In the case of isocyanates, it was found that both O−Si and N−Si bond formation was possible. Preferential oxygen bonding was observed when aromatic groups were incorporated on the silyl unit or when isocyanates featuring electron‐withdrawing groups were utilised. Further, it was found that in the case of **8**, the captured isothiocyanates could be exchanged for a range of isocyanate small molecules. This reactivity is reminiscent of (masked) FLP small molecule activations.^[14]^ Accessing small molecule capture and FLP chemistry with Zintl clusters unlocks their potential application as chemical storage materials or main group catalysts. The heteroallenes studied in this report are valence isoelectronic with CO_2_. Future investigations are focused on CO_2_ capture with Zintl clusters and the subsequent reduction chemistry of the activated heteroallenes.

## Experimental Section

The general information, experimental procedures, characterisation data, and computational details are available in the Supporting Information.

Deposition Numbers 2101379 (for **2**), 2101381 (for **6**), 2101382 (for **7**), 2101376 (for **8**), 2101377 (for **9**), 2101378 (for **10**), 2110872 (for **11**), 2101374 (for **15**), and 2101380 (for **18**) contain the supplementary crystallographic data for this paper. These data are provided free of charge by the joint Cambridge Crystallographic Data Centre and Fachinformationszentrum Karlsruhe Access Structures service.

## Conflict of interest

The authors declare no conflict of interest.

1

## Supporting information

As a service to our authors and readers, this journal provides supporting information supplied by the authors. Such materials are peer reviewed and may be re‐organized for online delivery, but are not copy‐edited or typeset. Technical support issues arising from supporting information (other than missing files) should be addressed to the authors.

Supporting Information

## Data Availability

The data that support the findings of this study are available in the supplementary material of this article.
