# Supplementary material for: Heteroallene Capture and Exchange at Functionalised Heptaphosphane Clusters
Source: Chemistry. 2021 Dec 21;28(6):e202103737. doi: 10.1002/chem.202103737 (PMC9300033; doi:10.1002/chem.202103737)
Supplement: Supplementary file 1 — Supporting Information [file CHEM-28-0-s001.pdf]

Chemistry–A European Journal

Supporting Information

Heteroallene Capture and Exchange at Functionalised Heptaphosphane Clusters

Bono van IJzendoorn, Inigo J. Vitorica-Yrezabal, George F. S. Whitehead, and Meera Mehta*

Table of Contents

1. Methods and Materials 4

1.1. Experimental Considerations 4

1.2. Analytical Considerations 4

1.3. X-ray diffraction studies 5

1.4. General computational considerations 6

1.5. NMR spectra simulations 6

2. Synthesis and Characterisation Data 7

2.1. Synthesis and Characterisation of $(R_3Si)_3P_7$ Compounds 7

2.1.1. Synthesis $(Me_2PhSi)_3P_7$ (10) 7

2.1.2. Synthesis $(MePh_2Si)_3P_7$ (11) 9

2.2. Synthesis and Characterisation of Isocyanates or Isothiocyanates Inserted Products 12

2.2.1. Identifying the Symmetric and Unsymmetric Isomers 12

2.2.2. Synthesis $(TMS\text{-}PhNCO)_3P_7$ (2) 14

2.2.3. Synthesis $(TMS\text{-}Br(C_6H_4)NCO)_3P_7$ (3) 20

2.2.4. Synthesis $(TMS\text{-}F(C_6H_4)NCO)_3P_7$ (4) 22

2.2.5. Synthesis $(TMS\text{-}CF_3(C_6H_4)NCO)_3P_7$ (5) 24

2.2.6. Synthesis $(TMS\text{-}TolylNCO)_3P_7$ (6) 27

2.2.7. Synthesis $(TMS\text{-}MeO(C_6H_4)NCO)_3P_7$ (7) 31

2.2.8. Synthesis $(TMS\text{-}PhNCS)_3P_7$ (8) 34

2.2.9. Synthesis $(TMS\text{-}TosylNCO)_3P_7$ (9) 37

2.2.10. Synthesis $(Me_2PhSi\text{-}PhNCO)_3P_7$ (13) 41

2.2.11. Synthesis $(MePh_2Si\text{-}PhNCO)_3P_7$ (14) 43

2.2.12. Synthesis $(Ph_3Si\text{-}PhNCO)_3P_7$ (15) 45

2.2.13. Synthesis $(Me_2PhSi\text{-}TosylNCO)_3P_7$ (16) 48

2.2.14. Synthesis $(MePh_2Si\text{-}TosylNCO)_3P_7$ (17) 52

2.2.15. Synthesis $(Ph_3Si\text{-}TosylNCO)_3P_7$ (18) 55

3. Heteroallene Exchange Studies 59

3.1. Exchange studies 59

3.1.1. Exchange of PhNCS for PhNCO in 8 59

3.1.2. Further exchange studies 60

4. Crystallography Tables 62

5. Computational studies 65

5.1. Energies of 1 and 2 Symmetric and Asymmetric Isomers 65

5.2. Fluoride Ion Affinity of Silyl Unit on compounds 1, 10-12 66
5.3. Heteroallene exchange 68
5.4. Geometry optimised structures 71
5.4.1. $(Me_3Si)_3P_7$ (1) symmetric isomer 71
5.4.2. $(Me_3Si)_3P_7$ (1) asymmetric isomer 72
5.4.3. $(TMS\text{-}PhNCO)_3P_7$ (2) symmetric isomer 74
5.4.4. $(TMS\text{-}PhNCO)_3P_7$ (2) asymmetric isomer B1 80
5.4.5. $(TMS\text{-}PhNCO)_3P_7$ (2) asymmetric isomer B2 83
5.4.6. $(TMS\text{-}PhNCO)_3P_7$ (2) asymmetric isomer B3 86
5.4.7. CF_2O 89
5.4.8. $[CF_3O]^-$ 89
5.4.9. $B(C_6F_5)_3$ 89
5.4.10. $[FB(C_6F_5)_3]^-$ 91
5.4.11. SbF_5 92
5.4.12. $[SbF_6]^-$ 92
5.4.13. $[Me_3Si]^+$ 92
5.4.14. Me_3SiF 93
5.4.15. $[(Me_3Si)_2P_7]^-$ 93
5.4.16. Me_2PhSiF 95
5.4.17. $(Me_2PhSi)_3P_7$ (10) 95
5.4.18. $[(Me_2PhSi)_2P_7]^-$ 98
5.4.19. $MePh_2SiF$ 99
5.4.20. $(MePh_2Si)_3P_7$ (11) 100
5.4.21. $[(MePh_2Si)_2P_7]^-$ 103
5.4.22. Ph_3SiF 105
5.4.23. $(Ph_3Si)_3P_7$ (12) 107
5.4.24. $[(Ph_3Si)_2P_7]^-$ 110
5.4.25. $(TMS\text{-}BrPhNCO)_3P_7$ (3) 113
5.4.26. $(TMS\text{-}FPhNCO)_3P_7$ (4) 116
5.4.27. $(TMS\text{-}CF_3PhNCO)_3P_7$ (5) 119
5.4.28. $(TMS\text{-}TolylNCO)_3P_7$ (6) 122
5.4.29. $(TMS\text{-}MeOPhNCO)_3P_3$ (7) 126
5.4.30. $(TMS\text{-}PhNCS)_3P_7$ (8) 129
5.4.31. PhNCO 132
5.4.32. $4\text{-}Br(C_6H_4)NCO$ 132

5.4.33. 4-F(C_6H_4)NCO 133
5.4.34. 4-CF_3(C_6H_4)NCO 134
5.4.35. PhNCS 134
5.4.36. p-tolylNCO 135
5.4.37. 4-MeO(C_6H_4)NCO 135
6. References 136

1. Methods and Materials

1.1. Experimental Considerations

All manipulations were performed under an inert atmosphere using standard Schlenk-line, and glovebox techniques. Glassware was flame dried prior to use.

Dry THF, Et_2O, toluene, and pentane were obtained using Innovative Technologies anhydrous engineering solvent purification systems and subsequently degassed. DME, and hexane were dried over Na, purified by distillation. THF-d_8, C_6D_6, and toluene-d_8, were dried over activated 3 Å molecular sieves. All solvents were stored over activated 3 Å molecular sieves.

Elemental phosphorus (Sigma-Aldrich), Naphthalene (Fluorochem), Me_3SiCl (Sigma-Aldrich), $Me_2PhSiCl$ (Acros organics), $MePh_2SiCl$ (Alfa Aesar), Ph_3SiCl (Fluorochem), PhNCO (Sigma-Aldrich), 4-Br(C_6H_4)NCO (Fluorochem), 4-F(C_6H_4)NCO (Fluorochem), 4-CF_3(C_6H_4)NCO (Sigma-Aldrich), PhNCS (Sigma-Aldrich), p-tolylNCO (Sigma-Aldrich), 4-MeO(C_6H_4)NCO (Fluorochem) and tosylNCO (Fluorochem) were purchased and used without further purification. Elemental sodium (Scientific Laboratories Supplies) was cleaned by removal of the oxide layers and washing with toluene/hexane. $[Na(DME)_x]_3P_7$,[1] $(Me_3Si)_3P_7$ (**1**),[1] and $(Ph_3Si)_3P_7$ (**12**) were synthesised according to previously reported synthetic procedures.[2]

1.2. Analytical Considerations

NMR Spectroscopy. ^1H, $^{13}C\{^1H\}$, ^{19}F, ^{29}Si DEPT90, ^{31}P NMR spectra were recorded on a Bruker AVIII 400 spectrometer (operating frequencies: 399.78 MHz, 100.53 MHz, 376.17 MHz, 79.48 MHz and 161.83 MHz for ^1H, ^{13}C, ^{19}F, ^{29}Si and ^{31}P, respectively). Variable temperature ^{31}P NMR spectra were recorded on a Bruker AVII 500 spectrometer (operating frequency: 202.46 MHz). Solid Sate ^1H, ^{13}C, ^{29}Si, ^{31}P NMR spectra were recorded on a Bruker AVIII 400 solid state spectrometer (operating frequencies: 100.53 MHz, 79.48 MHz and 161.83 MHz for ^{13}C, ^{29}Si and ^{31}P, respectively). ^1H and $^{13}C\{^1H\}$ NMR chemical shifts were internally referenced to the residual solvent resonances (C_6D_6 (benzene-d_6): ^1H δ = 7.16 ppm, $^{13}C\{^1H\}$ δ = 128.02

ppm, THF-d_8 (Tetrahydrofuran-d_8): ^1H δ = 3.58, 1.73 ppm, $^{13}C\{^1H\}$ δ = 67.57, 25.37 ppm, C_7D_8 (toluene-d_8): ^1H δ = 7.09, 7.00, 6.98, 2.09 ppm, $^{13}C\{^1H\}$ δ = 137.86, 129.24, 128.33, 125.49, 20.4 ppm). ^{19}F, ^{29}Si, ^{31}P chemical shifts were externally referenced to $CFCl_3$, Me_4Si, H_3PO_4, respectively. Solution phase NMR samples were prepared under an inert atmosphere in 5 mm J Youngs NMR tubes. Solid state NMR sample were prepared under an inert atmosphere in 4 mm rotors. Data was analysed using MestReNova V14.0.0 software or Topspin V3.6.1 software.

Elemental Analysis. Elemental analysis was carried out by the microanalysis service of the University of Manchester using a Flash 2000 elemental analyser.

Mass spectrometry. Mass spectrometry samples were measured by the mass spectrometry service of the University of Manchester using an electrospray ionisation equipped Thermo Orbitrap Executive Plus Extended Mass Range mass spectrometer. Samples were prepared under a nitrogen atmosphere and directly injected into the ionisation source of the mass spectrometer.

1.3. X-ray diffraction studies

Data collection: X-ray diffraction data were collected for compounds **2**, **6**, **7**, **8**, **9**, **10**, **11**, **15** and **18** at 100K on a dual source (Mo (λ = 0.71073 Å) and Cu (λ = 1.54146 Å)) Rigaku FR-X rotating anode diffractometer with a Hypix-6000HE detector and an Oxford Cryosystems nitrogen flow gas system. Data were measured with CrisAlisPro software.

Crystal structure determination and refinements: X-ray data were processed and reduced using CrysAlisPro. Absorption correction was performed using empirical methods (SCALE3 ABSPACK) based upon symmetry-equivalent reflections combined with measurements at different azimuthal angles. The crystal structure was solved and refined against all F^2 values using the SHELX and Olex2 suite of programmes.[3] All atoms were refined anisotropically with the exception of the disordered diethyl ether solvent molecules in compound **15**. Hydrogen atoms were placed in calculated positions and refined using idealised geometries and assigned fixed isotropic displacement parameters. Four chemically identical molecules of compound **6** were

found disordered and modelled over two positions in the crystal structure. Disordered diethyl ether molecules were modelled and refined in crystals structures of **15** and **18.** Atomic displacement parameters were restrained using a rigid body approach by applying SHELX RIGU commands and to be similar using SHELX SIMU commands.

Crystallographic data have been deposited with the CCDC (CCDC 2101374, 2101376-2101382, 2110872).

1.4. General computational considerations

Density Functional Theory (DFT) calculations were performed with the Gaussian09 program package3 (version g09, rev.d01).[4] Geometry optimisations and frequency calculations were conducted at the BP86/SV(p), PBE1PBE/6-31G(d,p), or PBE1PBE/6-311G(d,p) level. No symmetry constraints were applied during optimisation. All minima were confirmed by the absence of imaginary frequencies. Initial geometries were prepared using X-ray diffraction coordinates where possible and Facio V22.1.1.64 software. Gibbs free reaction energies and enthalpies were calculated for standard conditions (p = 1 atm, T = 298 K) and are unscaled. Images were prepared using Mercury 4.3.1. software.

1.5. NMR spectra simulations

NMR spectra simulations were performed with the gNMR software package (version 5.06).[5] Experimental NMR spectra were imported into the gNMR package using the gCVT and gSPG applications. Initial values for the chemical shift, linewidth, and coupling constant parameters for the fitting procedure were taken from the experimental data, and modified manually until the calculated spectrum closely resembled the experimental spectrum. Simulated spectra were refined using the iterate function in gNMR. Some $^{2}J_{PP}$ coupling constants were set to 0 Hz and excluded from the iteration process as their inclusion in the fitting process did not make a clear change to the observed calculated band shapes.

2. Synthesis and Characterisation Data

2.1. Synthesis and Characterisation of $(R_3Si)_3P_7$ Compounds

2.1.1. Synthesis $(Me_2PhSi)_3P_7$ (10)

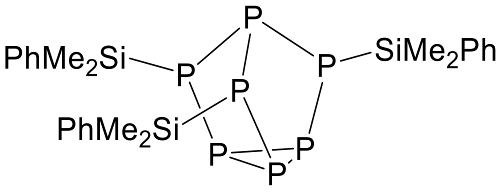

To a Schlenk flask charged with a stir bar and $[Na(DME)_x]_3[P_7]$ (500 mg, ~0.84 mmol) toluene (5 mL) was added to give a dark green suspension. The suspension was cooled to –80 °C and $Me_2PhSiCl$ (0.43 mL, 2.56 mmol) was added dropwise. The reaction mixture was allowed to slowly warm to room temperature over the course of 1h, and a black suspension observed. The reaction was allowed to stir overnight. The mixture was filtered, and the residue was rinsed with toluene (5 mL). The filtrate was cooled to 5 °C overnight allowing for crystals to form. The mother liquor was removed *via* filtration and the crystals were washed with toluene (3 mL). The crystals were dried under reduced pressure to give a fine yellow solid. Crystals suitable for single crystals X-ray diffraction analysis were obtained through cooling down a concentrated toluene solution.
Isolated Yield: 516 mg, 96%.

^{1}H NMR (400 MHz, 298 K, C_6D_6): δ = 7.42 - 7.32 (m, 6H, *Ph*), 7.05 - 6.95 (m, 9H, *Ph*), 0.27 (s, 18H, *Me*) ppm. **^{13}C{^{1}H} NMR (101 MHz, 298 K, C_6D_6):** δ = 138.38 (s, *Ph*), 134.39 (s, *Ph*), 133.40 (s, *Ph*), 129.93 (s, *Ph*), 2.08 (s, *Me*) ppm. **^{29}Si DEPT90 NMR (79 MHz, 298 K, C_6D_6):** δ = 0.75 (d, $^{1}J_{PSi}$ = 45 Hz) ppm. **^{31}P NMR (162 MHz, 298 K, C_6D_6):** δ = 5.75 - –2.81 (m, 3P, bridging), –97.80 - –98.98 (qq, $^{1}J_{PP}$ = 325 Hz, $^{2}J_{PP}$ = 46 Hz, 1P, apical), –151.18 - –159.05 (m, 3P, basal) ppm.
Elemental analysis for $C_{24}H_{33}P_7Si_3$: calcd.: C 46.30, H 5.34, N 0.0; found: C 45.97, H 5.31, N 0.0.

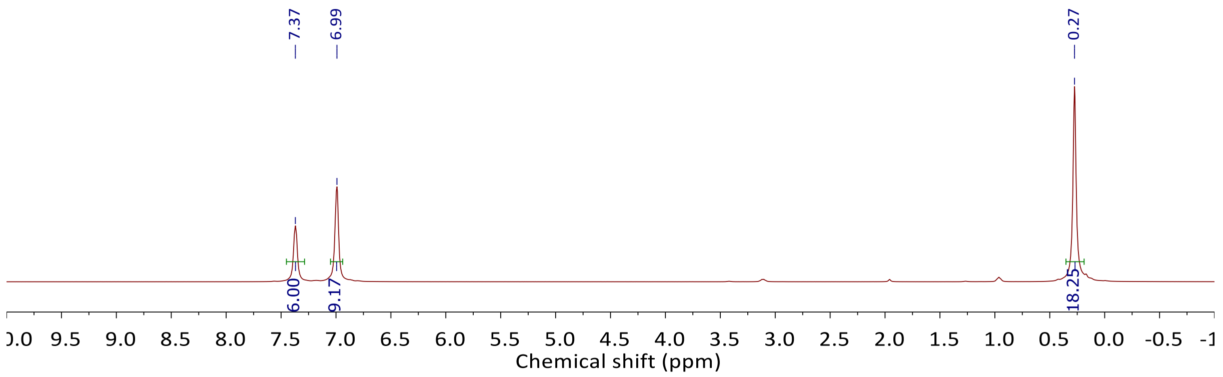

Figure S1. ^{1}H NMR spectrum (C_6D_6) of **10**.

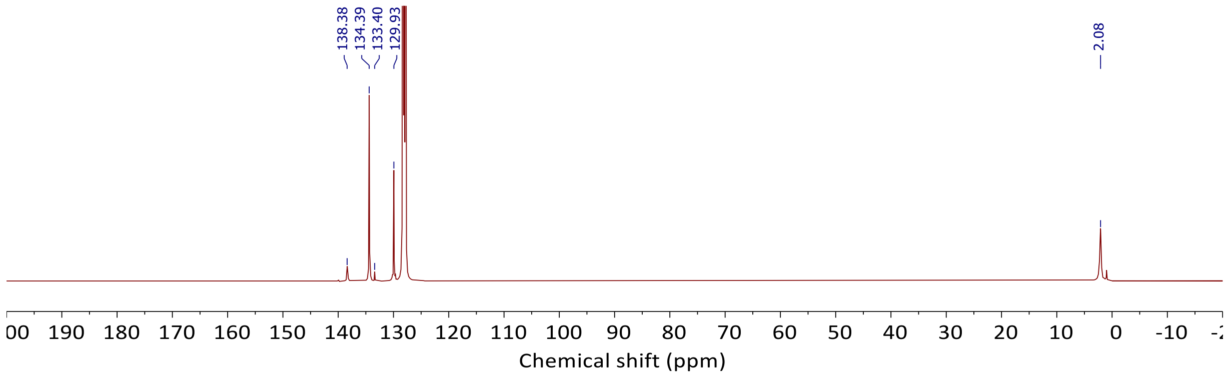

Figure S2. $^{13}C\{^{1}H\}$ NMR spectrum (C_6D_6) of **10**.

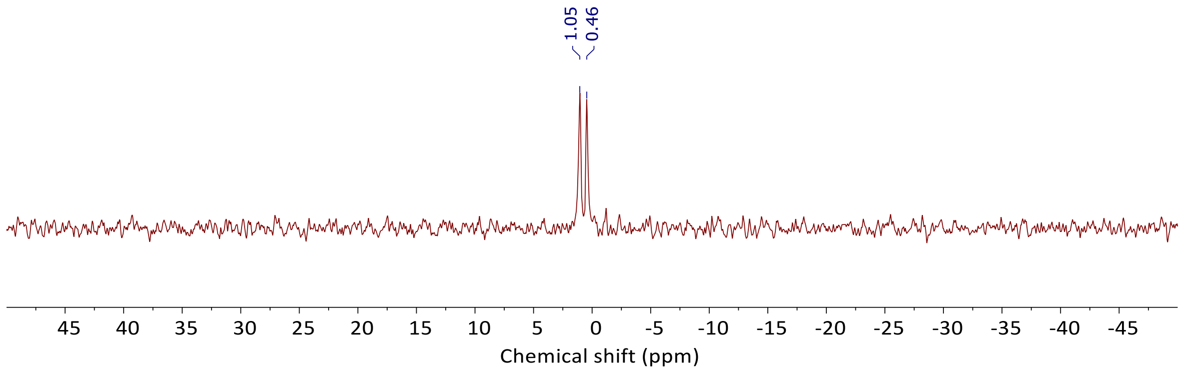

Figure S3. ^{29}Si DEPT90 NMR spectrum (C_6D_6) of **10**.

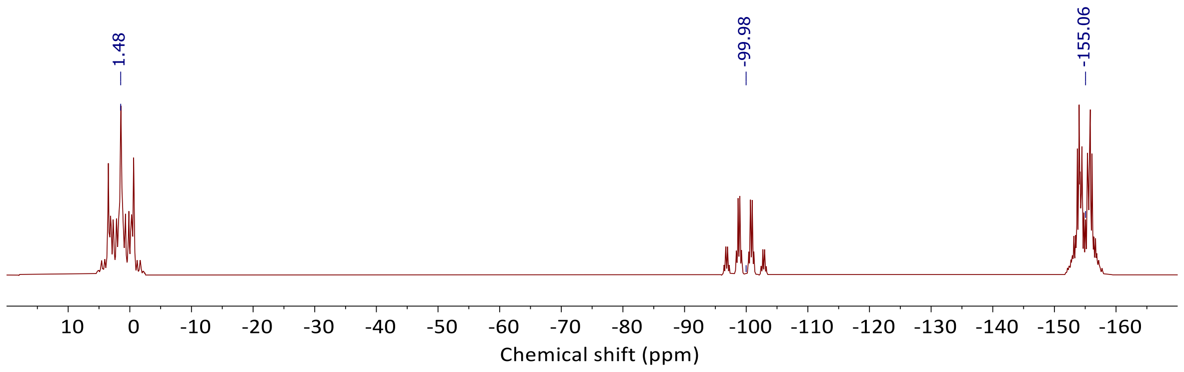

Figure S4. ^{31}P NMR spectrum (C_6D_6) of **10**.

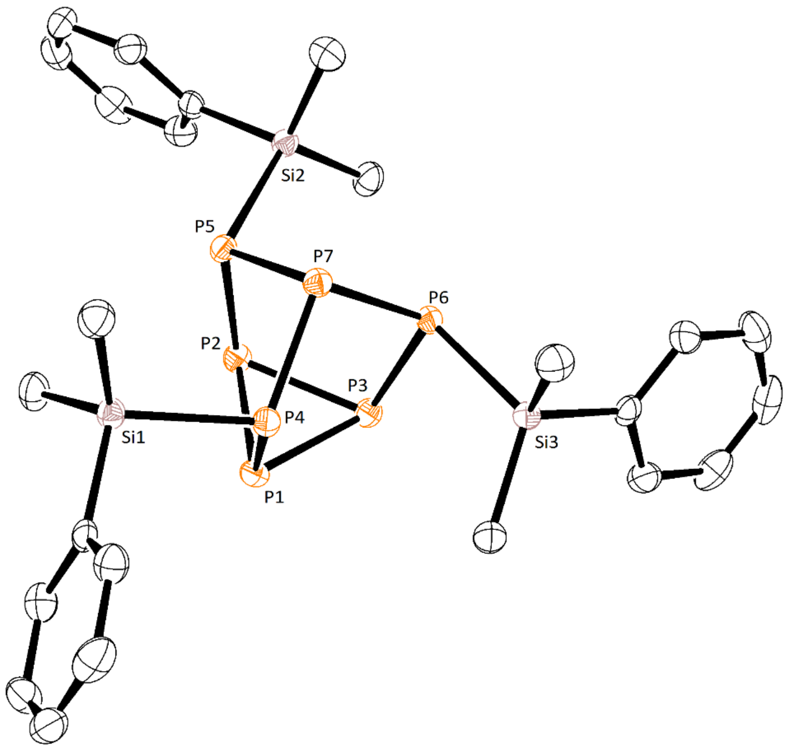

Figure S5. Molecular structure of **10**. Anisotropic displacement ellipsoids pictured at 50% probability. Hydrogen atoms omitted for clarity. Phosphorus: Orange; Silicon: Pink; Carbon: White.

2.1.2. Synthesis $(MePh_2Si)_3P_7$ (11)

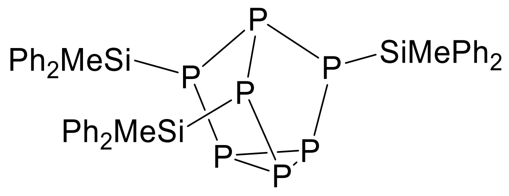

To a Schlenk flask charged with a stir bar and $[Na(DME)_x]_3$[**1**] (500 mg, ~0.84 mmol) toluene (5 mL) was added to give a dark green suspension. The suspension was cooled to –80 °C and $MePh_2SiCl$ (1.05 mL, 4.90 mmol) was added dropwise. The reaction mixture was allowed to slowly warm to room temperature over the course of 1h, and a black suspension observed. The reaction was allowed to stir overnight. The mixture was filtered, and the residue was rinsed with toluene (5 mL) yielding a clear yellow solution. The solvent was removed under reduced pressure and the residue was dissolved in Et_2O. Upon concentrating of the solvent, precipitation was observed. The residual solvent was removed *via* filtration and the precipitate was dried in vacuo,

yielding yellow solids. Crystals suitable for single crystals X-ray diffraction analysis were obtained through slow evaporation of a concentrated Et_2O solution.

Isolated Yield: 224 mg, 33%.

^{1}H NMR (400 MHz, 298 K, Tol-d$_8$): δ = 7.50 - 7.38 (m, 12H, *Ph*), 7.10 - 6.94 (m, 18H, *Ph*), 0.55 (s, 9H, *Me*) ppm. **^{13}C{^{1}H} NMR (101 MHz, 298 K, Tol-d$_8$):** δ = 136.58 (s, *Ph*), 134.67 (s, *Ph*), 129.13 (s, *Ph*), 127.27 (s, *Ph*), 0.35 (s, *Me*) ppm. **^{29}Si DEPT90 NMR (79 MHz, 298 K, Tol-d$_8$)**: δ = –4.33 (d, $^1J_{SiP}$ = 48 Hz) ppm. **^{31}P NMR (162 MHz, 298 K, Tol-d$_8$):** δ = 3.74 - –3.81 (m, 3P, bridging), –95.57 - –103.51 (qq, $^1J_{PP}$ = 329 Hz, $^2J_{PP}$ = 45 Hz, 1P, apical), –148.87 - –155.65 (m, 3P, basal) ppm.

Elemental analysis for $C_{39}H_{39}P_7Si_3$: calcd.: C 57.92, H 4.86, N 0.0; found: C 58.28, H 4.92, N 0.0.

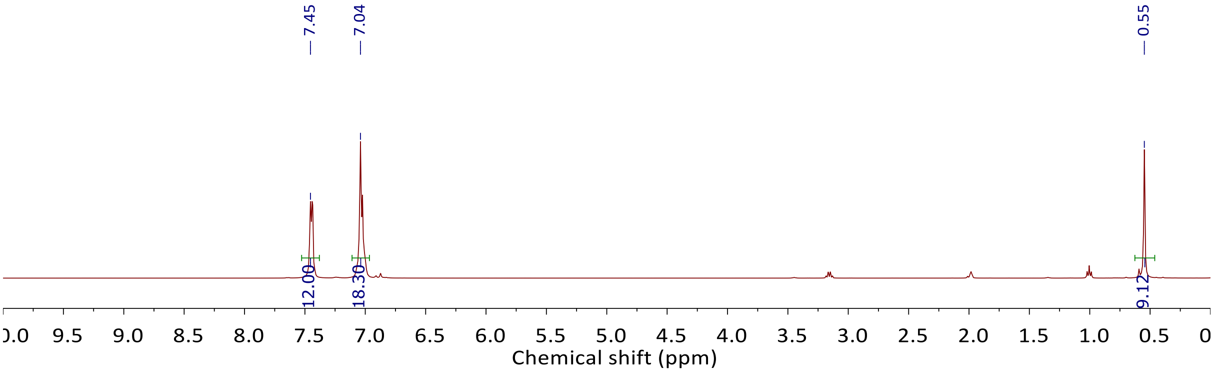

Figure S6. ^{1}H NMR spectrum (Tol-d$_8$) of **11**.

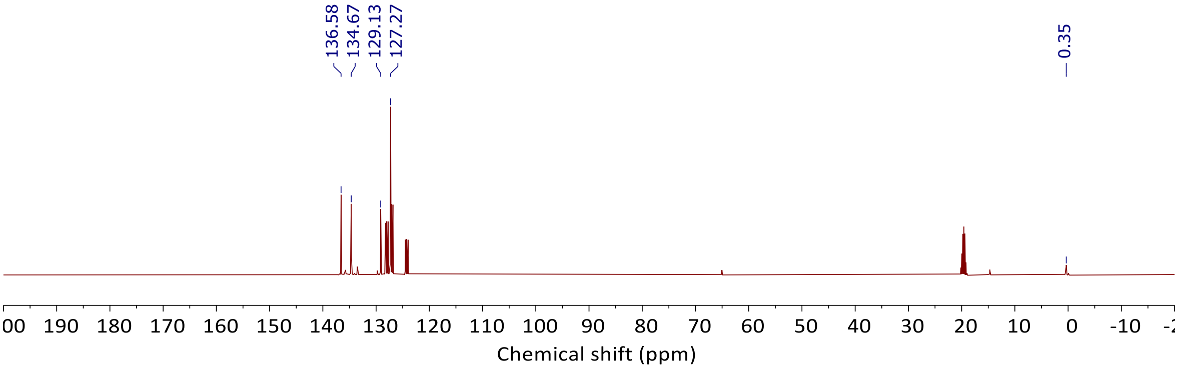

Figure S7. ^{13}C{^{1}H} NMR spectrum (Tol-d$_8$) of **11**.

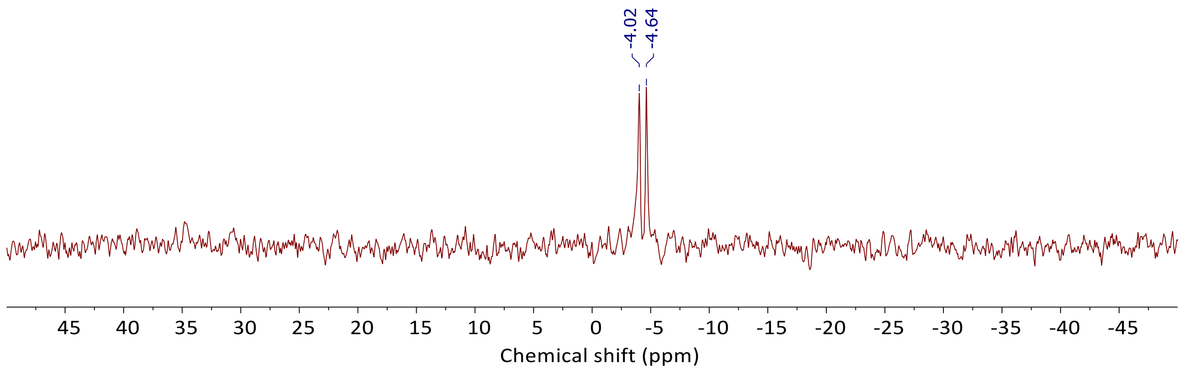

Figure S8. ^{29}Si DEPT NMR spectrum (Tol-d_8) of **11**.

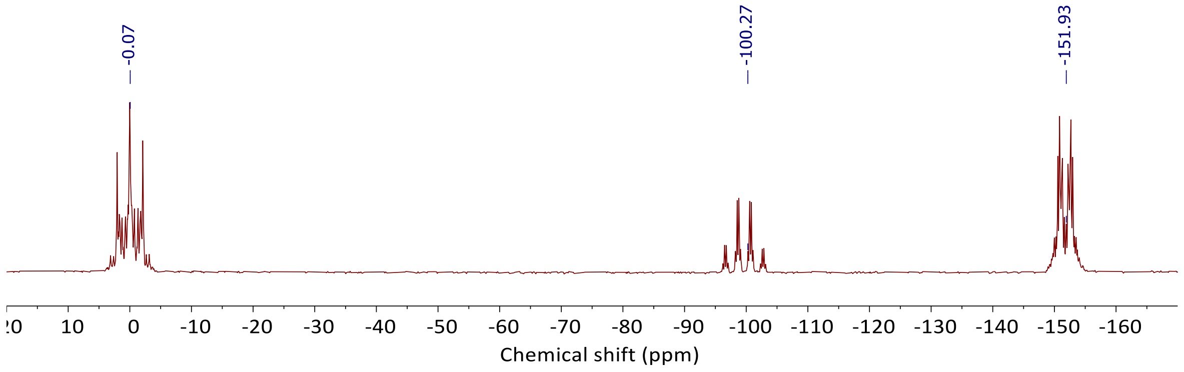

Figure S9. ^{31}P NMR spectrum (Tol-d_8) of **11**.

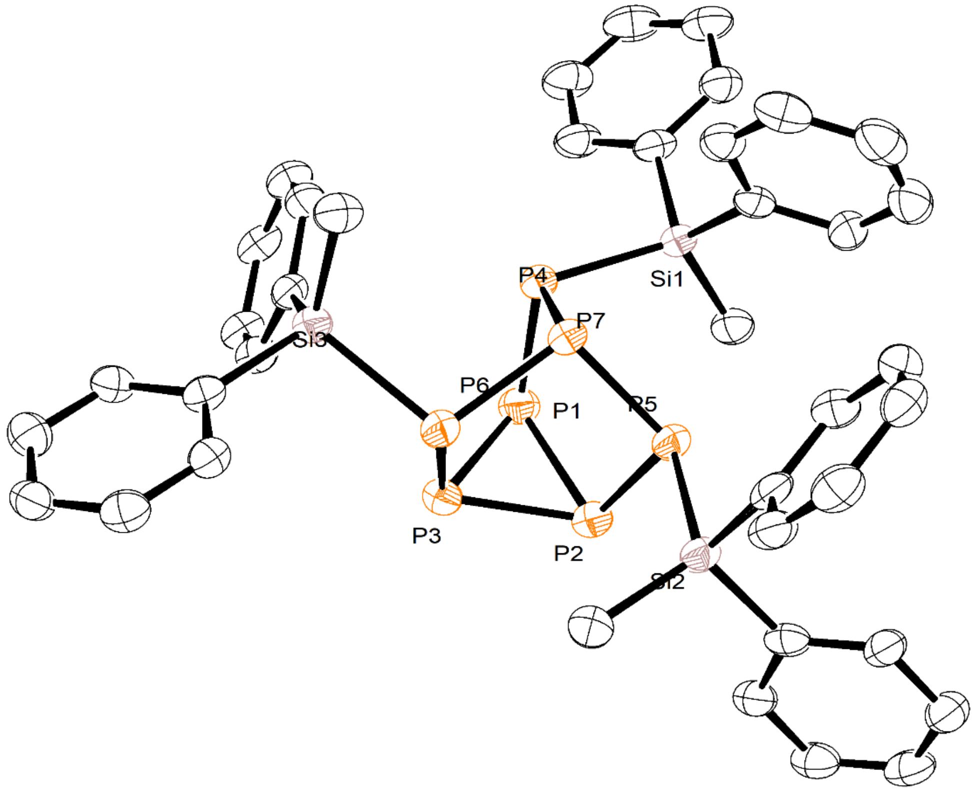

Figure S10. Molecular structure of **11**. Anisotropic displacement ellipsoids pictured at 50% probability. Hydrogen atoms omitted for clarity. Phosphorus: Orange; Silicon: Pink; Carbon: White.

2.2. Synthesis and Characterisation of Isocyanates or Isothiocyanates Inserted Products

2.2.1. Identifying the Symmetric and Unsymmetric Isomers

The geometry at the bridging phosphorus atoms of the [P_7] core for functionalised clusters is pyramidal and introduces chirality. Further, there are two possible isomers a symmetric coordination mode (Figure S11, **A**) and an asymmetric coordination mode (Figure S11, **B**). In 2011, Hassler and co-workers studied the **A** and **B** isomers of compound **1** using density functional theory (DFT) and ^{31}P NMR spectroscopy.[6] The symmetric coordination mode **A** gives three unique phosphorus magnetic environments (apical, bridging, and basal), while in the asymmetric coordination mode **B** all seven of the phosphorus atoms are magnetically inequivalent. Different tautomers, allowed by the amide/imidate mixed functional groups, further complicates the NMR spectroscopy studies as there are three possible asymmetric coordination modes each with seven inequivalent phosphorus magnetic environments (Figure S11, **III**). Additionally, each of **A** and **B** isomers would be expected to exist as racemic mixtures. XRD experiments of **2**, **6-9**, **15**, and **18** all co-crystallized both of the **A** enantiomers (Figure S11, A). The symmetric coordination mode **A** is expected to be the thermodynamic product, and can be selectively detected with [P_7] cages that feature bulky functional groups.[6] The ^{31}P NMR spectrum of **9**, and **16**-**18** showed three resonances corresponding to the apical, bridging, and basal phosphorus environments. Compounds **9**, and **16**-**18** solely exhibit the symmetric coordination isomer **A**, as a result of the sterically bulky tosyl groups on nitrogen and selective silyl coordination to oxygen (Figure S11, I). Whereas, the ^{31}P NMR spectrum of **6**-**8**, **14**, and **15** display ten resonances, in line with both **A** and **B** isomers being present, made possible by the smaller functional groups on the isocyanate or isothiocyanate, and selective silyl coordination to either nitrogen or oxygen (Figure S11, **II**). Finally, the ^{31}P NMR spectrum of **2**-**5**, and **13** revealed 24 overlapping resonances, consistent with both **A** and **B** isomers being present and the differing silyl tautomers (Figure S11, **III**). DFT investigation showed that the energy difference for **2** between **A** and the iterations of **B** are between 11 and 25 kJ/mol (Section 5.1), in agreement with all isomers being observed by ^{31}P NMR spectroscopy.

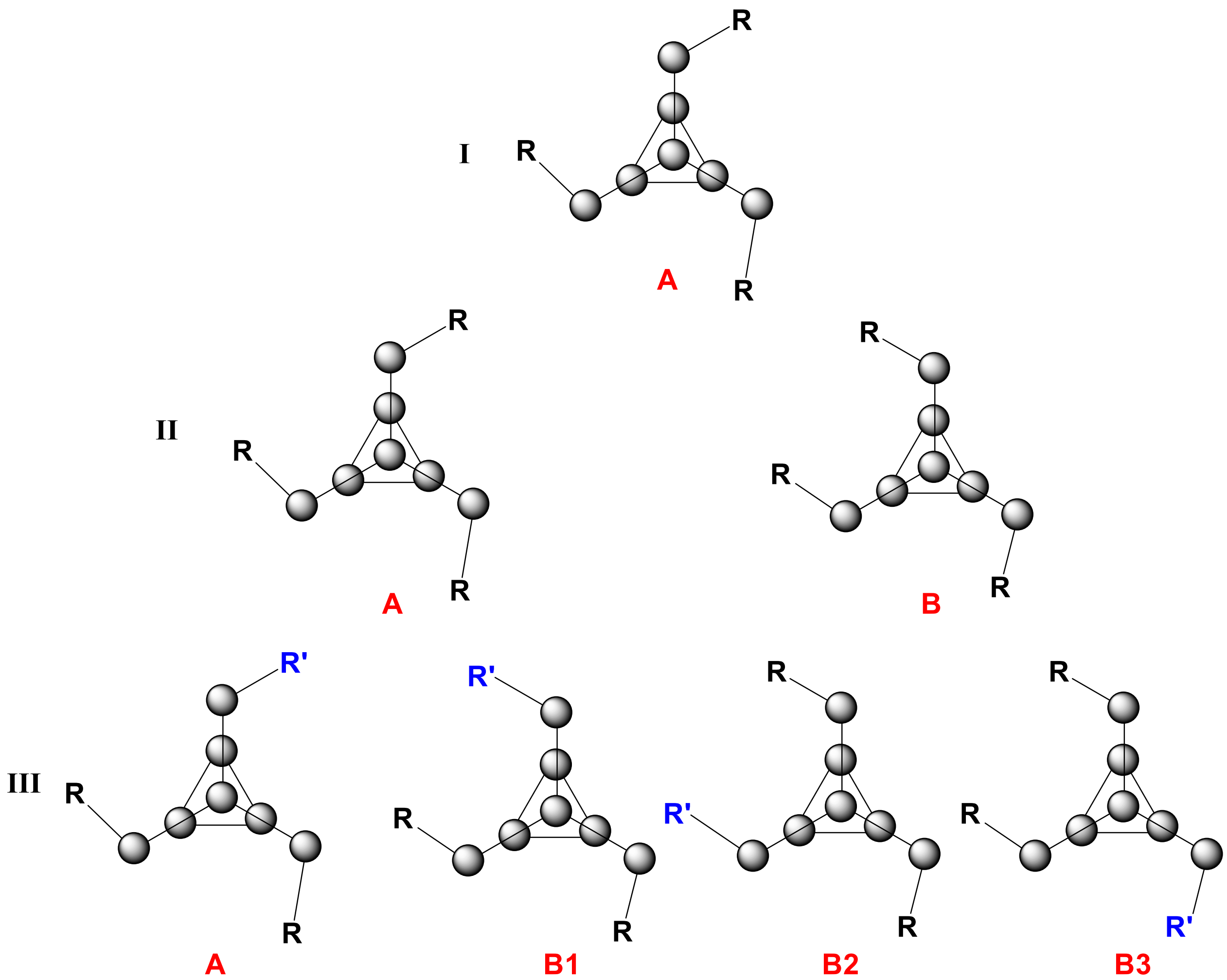

Figure S11. Symmetric (**A**) and Asymmetric (**B**) Isomers of Functionalised [P_7] Cages

For clusters **2-5**, and **13** where the silyl units are bound to both N and O atoms of the captured isocyanate, 24 signals are expected in the ^{31}P NMR spectrum corresponding to the **A**, **B1**, **B2**, and **B3** isomeres (Figure S11, **III**). Compound **2** was selected as a model compound and investigated using ^{31}P COSY experiments to assign the **A** and **B** isomers. Using this COSY data, integration, and line shape isomers for **3-5**, and **13** were assigned.

For clusters **6-8** where the silyl unit are bound to only the N atoms of the captured isocyanate, 10 signals are expected in the ^{31}P NMR corresponding to the **A** and **B** isomers (Figure S11, II). Similary, for clusters **14**, and **15** where the silyl unit are bound to only the O atoms of the captured isocyanate, 10 signals are expected in the ^{31}P NMR corresponding to the **A** and **B** isomers (Figure S11, **II**). Compound **6** was

selected as model compounds and investigated using ^{31}P COSY experiments to assign the **A** and **B** isomers. Using this COSY data, integration, and line shape isomers for **7**, **8**, **14**, and **15** were assigned.

2.2.2. Synthesis (TMS-PhNCO)$_3$P$_7$ (2)

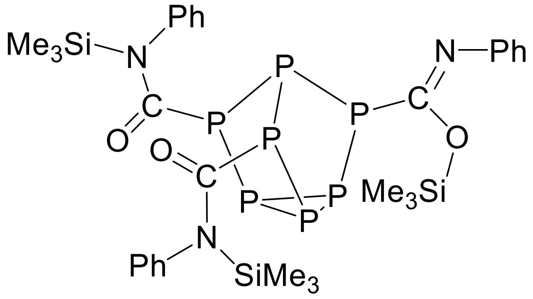

To a J young ampoule charged with a stir bar and (Me$_3$Si)$_3$P$_7$ (**1**) (100 mg, 0.23 mmol, 1.0 eq.) THF (0.4 mL) was added. To a separate vial, PhNCO (82 mg, 0.690 mmol, 3.0 eq.) and THF (0.4 mL) was added. The solution of PhNCO was added to the (Me$_3$Si)$_3$P$_7$ solution and allowed to react overnight at room temperature. The volatiles were removed yielding a glassy solid. The glassy solid was dissolved in Et$_2$O (8 mL). Slow evaporation of the solvent yielded block shaped crystals. The crystals were washed with Et$_2$O (5 mL) and residual solvent removed, yielding a white crystalline solid. Crystals suitable for single crystals X-ray diffraction analysis were obtained through slow evaporation of a concentrated Et$_2$O solution.
Isolated Yield: 159 mg, 87%.

^{1}H NMR (400 MHz, 298 K, THF-d$_8$): δ = 7.79 - 6.26 (m, 15H, *Ph*), 0.22 - 0.13 (overlapping singlets, 9H, *Me*), 0.14 - –0.16 (overlapping singlets, 18H, *Me*) ppm. **^{13}C{^{1}H} NMR (101 MHz, 298 K, THF-d$_8$):** δ = 182.26 (d, $^1J_{CP}$ = 46 Hz, (TMS-PhN*C*O)$_3$P$_7$), 150.91 (s, *Ph*), 150.59 (s, *Ph*), 143.42 (s, *Ph*), 143.00 (s, *Ph*), 142.16 (s, *Ph*), 133.66 (s, *Ph*), 132.67 (s, *Ph*), 132.46 (s, *Ph*), 132.30 (s, *Ph*), 131.04 (s, *Ph*), 130.66 (s, *Ph*), 130.51 (s, *Ph*), 130.08 (s, *Ph*), 129.92 (s, *Ph*), 129.69 (s, *Ph*), 123.98 (s, *Ph*), 123.74 (s, *Ph*), 1.33 (s, *Me*), 0.90 (s, *Me*) ppm. **^{29}Si DEPT90 NMR (79 MHz, 298 K, THF-d$_8$):** δ = 24.19 (s, Me$_3$*Si*–O), 13.04 (s, Me$_3$*Si*–N), 12.78 (s, Me$_3$*Si*–N) ppm. **^{31}P NMR (162 MHz, 298 K, THF-d$_8$):** δ = 140.29 - 134.63 (m, 4P, *bridging B*), 134.38 - 128.87 (m, 3P, *bridging A*), 125.41 - 115.00 (m, 3P, *bridging B*), 104.26 - 94.38 (m,

2P, *bridging B*), –117.66 - –134.21 (m, 4P, *apical A and B*), –167.78 - –186.47 (m, 12P, *basal A and B*).

Mass spectrometry (ESI neg/pos): $C_{30}H_{42}N_3O_3P_7Si_3$+Na ([M+Na]$^+$): calcd.: 816.0589; found: 816.0585.

Elemental analysis for $C_{30}H_{42}N_3O_3P_7Si_3$: calcd.: C 45.40, H 5.33, N 5.29; found: C 45.68, H 5.33, N 5.30.

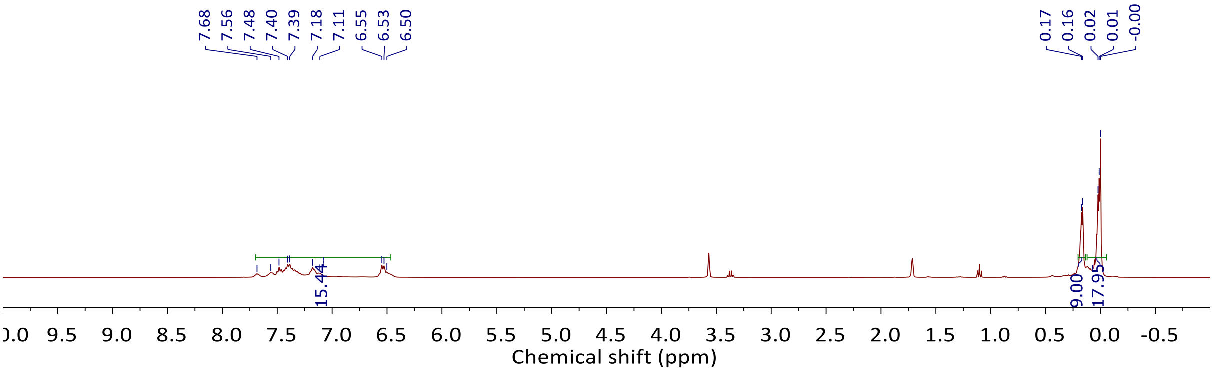

Figure S12. ^{1}H NMR spectrum (THF-d_8) of **2**.

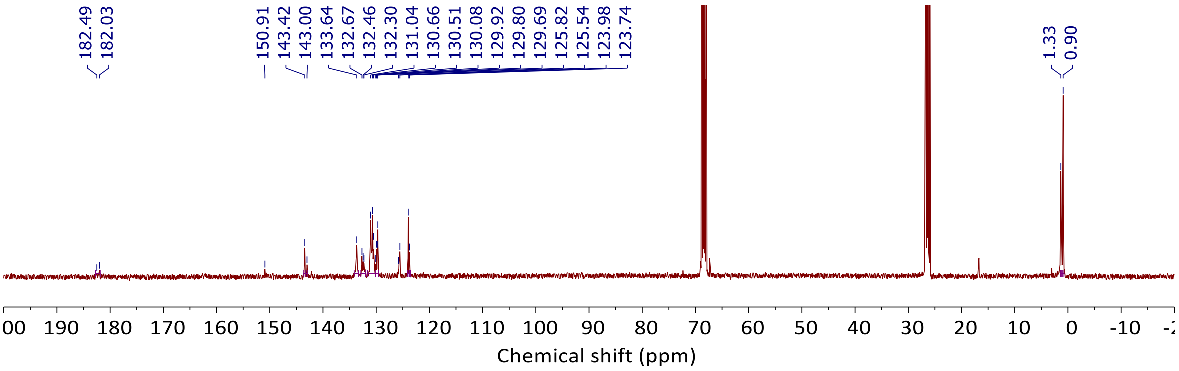

Figure S13. ^{13}C{^{1}H} NMR spectrum (THF-d_8) of **2**.

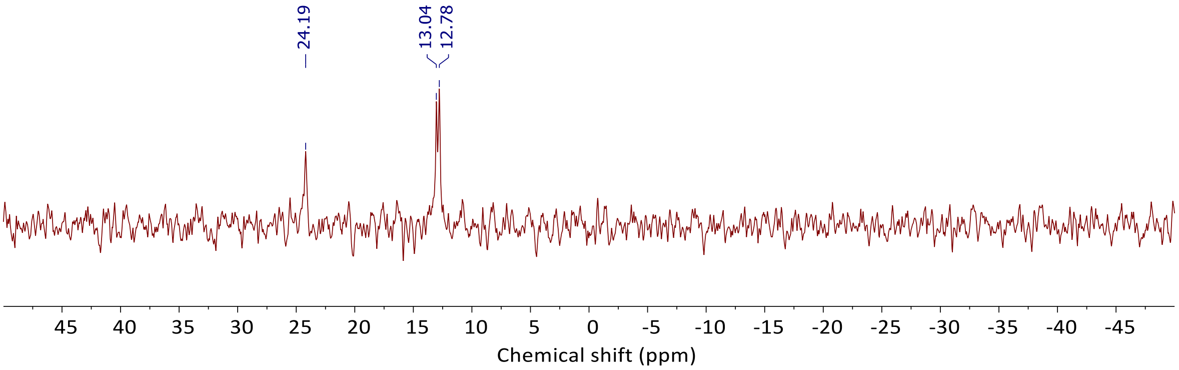

Figure S14. ^{29}Si DEPT90 NMR spectrum (THF-d_8) of **2**.

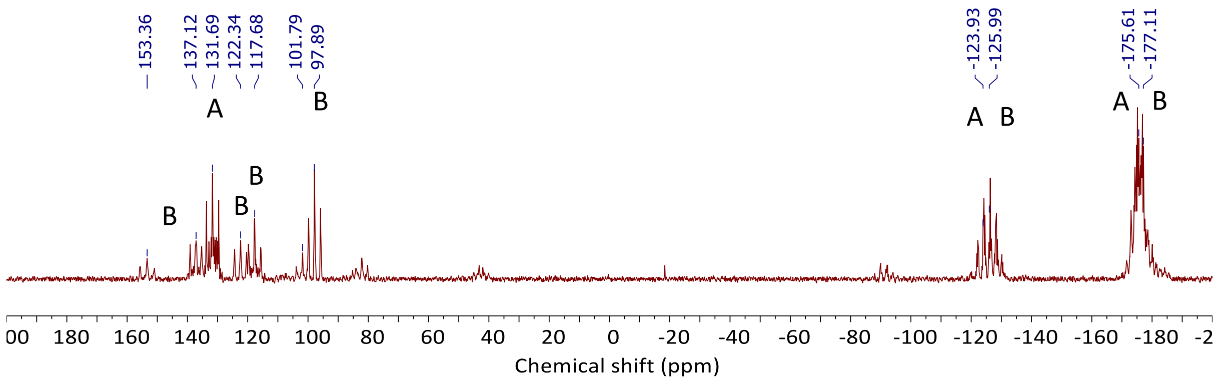

Figure S15. 31P NMR spectrum (THF-d8) of **2**.

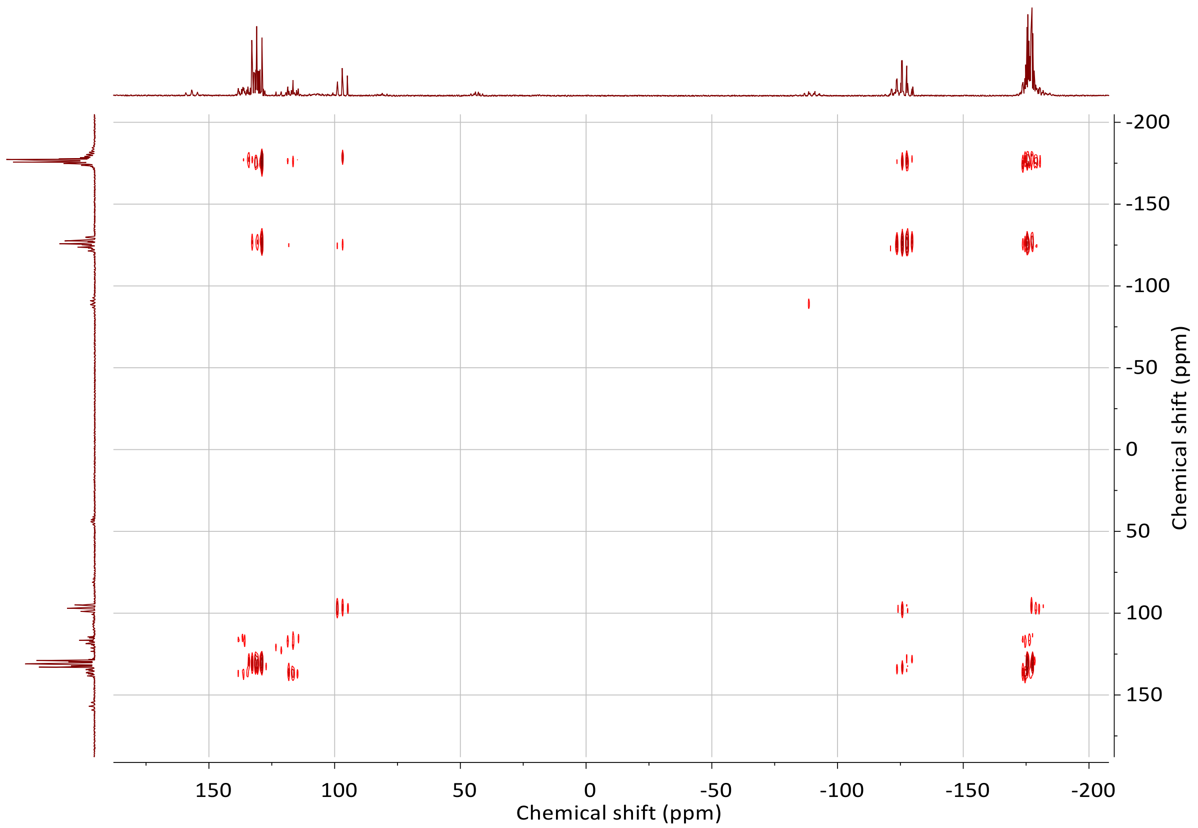

Figure S16. ^{31}P COSY NMR spectrum (THF-d_8) of **2**.

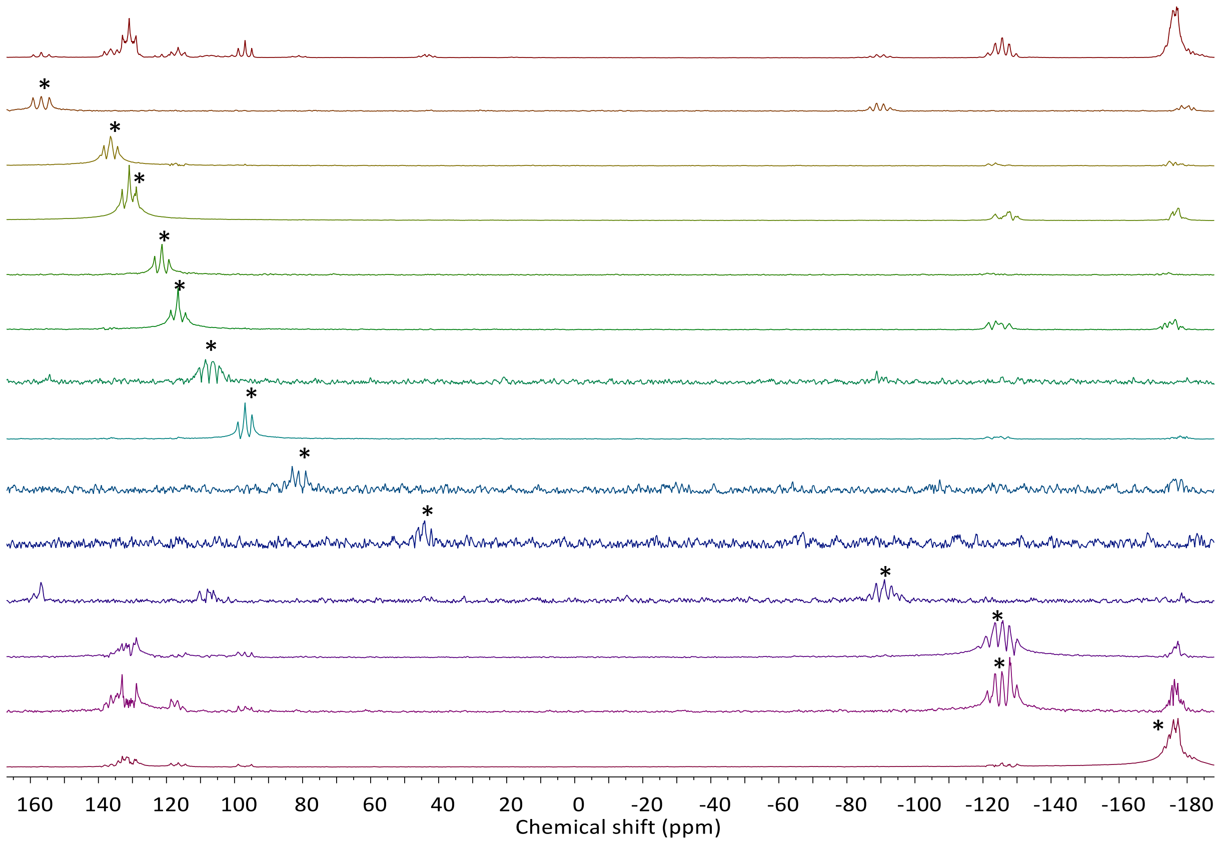

Figure S17. ^{31}P 1D selective COSY NMR spectra (THF-d_8) of **2**. Top spectrum ^{31}P NMR followed by selective radiation (marked by *) of resoances at 156.92, 136.58, 130.89, 121.55, 116.65, 107.57, 96.98, 81.02, 43.50, –89.77, –122.93, –126.57, and –176.23 ppm. Optimised for 330 Hz coupling.

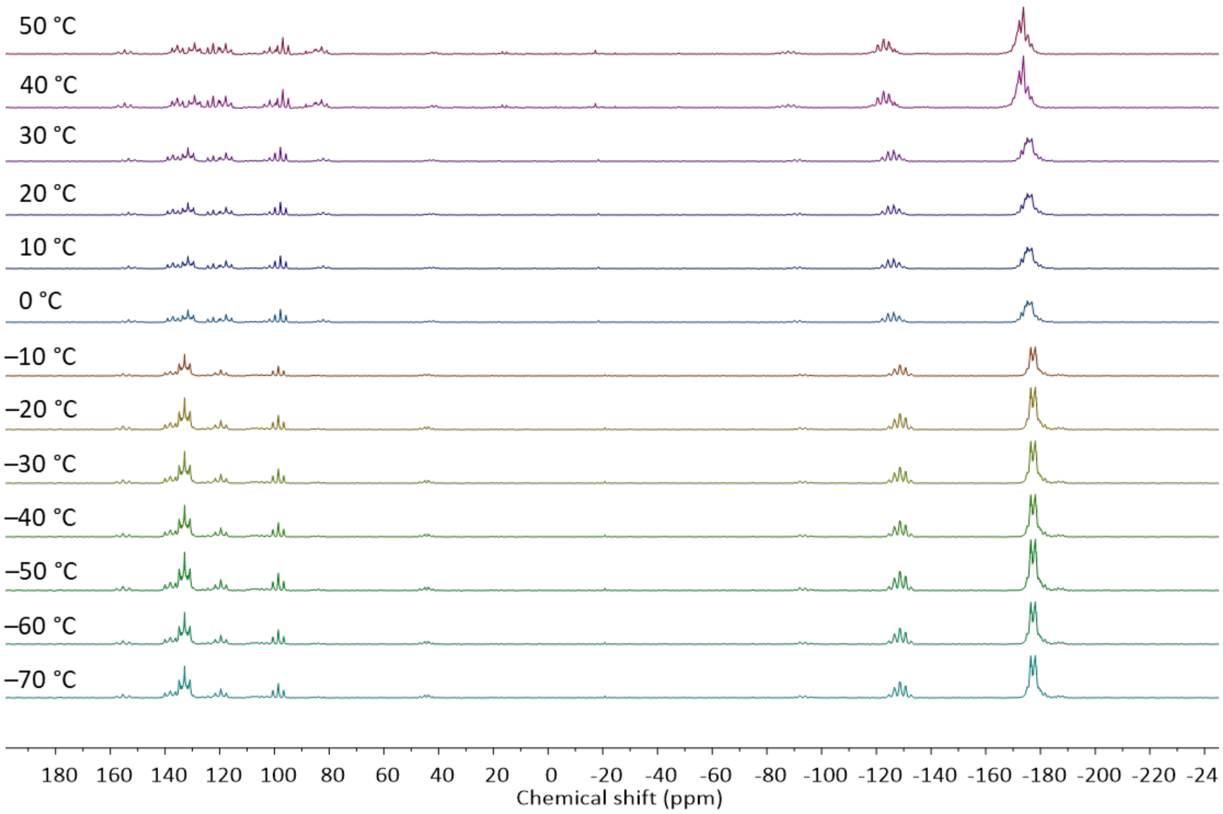

Figure S18. ^{31}P NMR spectra (THF-d_8) of **2** at variable temperature.

*Note: Some decomposition of **2** was observed at 40 and 50 °C. Prolonged exposure at 50 °C resulted in complete decomposition.*

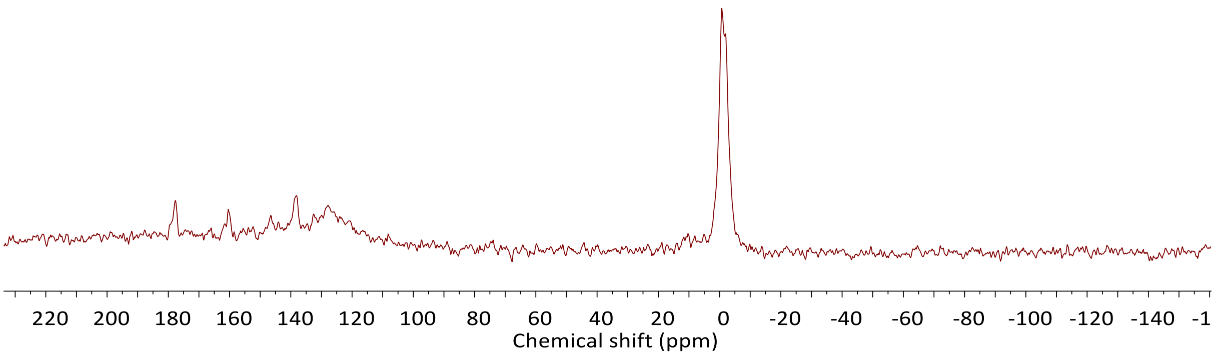

Figure S19. ^{13}C SS NMR spectra of **2**. MAS 12KHz.

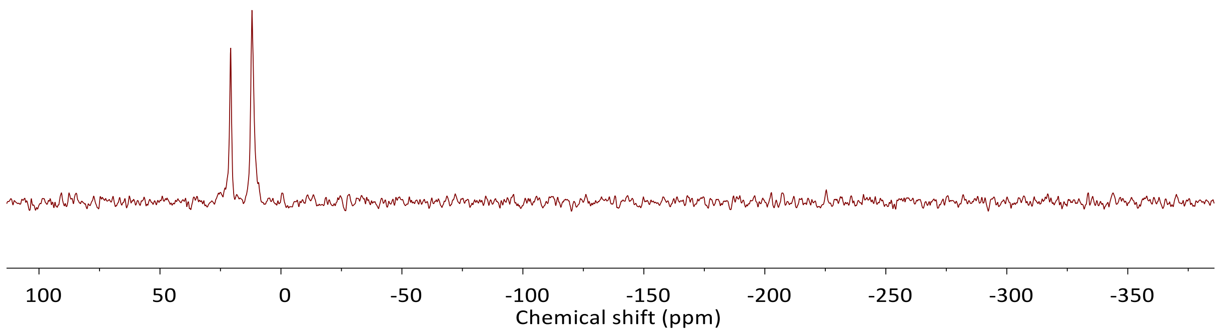

Figure S20. ^{29}Si SS NMR spectra of **2**. MAS 12KHz.

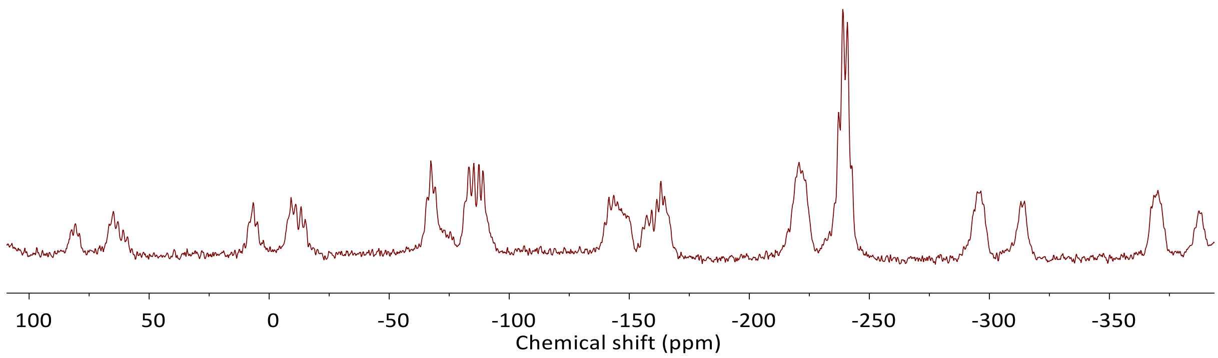

Figure S21. ^{31}P SS NMR spectra of **2**. MAS 12KHz.

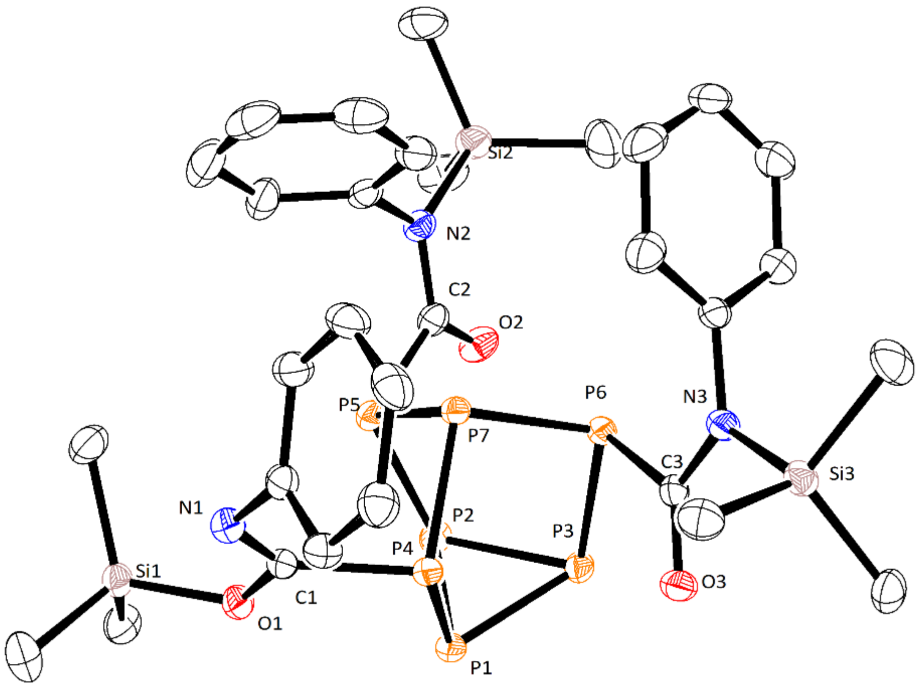

Figure S22. Molecular structure of **2**. Anisotropic displacement ellipsoids pictured at 50% probability. Hydrogen atoms omitted for clarity. Phosphorus: Orange; Silicon: Pink; Carbon: White; Oxygen: Red; Nitrogen: Blue.

2.2.3. Synthesis (TMS-Br(C_6H_4)NCO)$_3$$P_7$ (3)

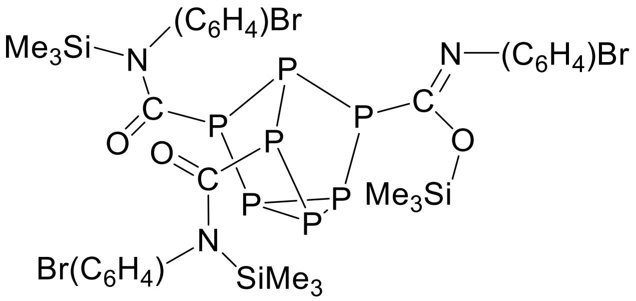

To a J young ampoule charged with a stir bar and (Me_3Si)$_3$$P_7$ (**1**) (100 mg, 0.23 mmol, 1.0 eq.) THF (0.4 mL) was added. To a separate vial, 4-Br(C_6H_4)NCO (137 mg, 0.69 mmol, 3.0 eq.) and THF (0.4 mL) was added. The solution of 4-Br(C_6H_4)NCO was added to the (Me_3Si)$_3$$P_7$ solution and allowed to react overnight at room temperature. The volatiles were removed yielding a glassy solid.

Isolated Yield: 93 mg, 39%.

^{1}H NMR (400 MHz, 298 K, THF-d_8): δ = 7.93 - 6.20 (m, 12H, *Ar*), 0.17 - 0.10 (overlapping singlets, 9H, *Me*), 0.04 - –0.02 (overlapping singlets, 18H, *Me*) ppm. **^{13}C{^{1}H} NMR (101 MHz, 298 K, THF-d_8):** δ = 181.82 (d, $^1J_{CP}$ = 51 Hz, (TMS-Br(C_6H_4)N*C*O)$_3$$P_7$), 181.23 (d, $^1J_{CP}$ = 43 Hz, (TMS-Br(C_6H_4)N*C*O)$_3$$P_7$), 163.15 (s, *Ar*), 162.30 (s, *Ar*), 150.18 (s, *Ar*), 149.74 (s, *Ar*), 142.30 (s, *Ar*), 142.21 (s, *Ar*), 135.02 (s, *Ar*), 134.83 (s, *Ar*), 134.31 (s, *Ar*), 134.22 (s, *Ar*), 133.76 (s, *Ar*), 133.64 (s, *Ar*), 133.61 (s, *Ar*), 133.31 (s), 126.05 (s, *Ar*), 125.69 (s, *Ar*), 123.51 (m, *Ar*), 119.04 (s, *Ar*), 118.65 (s, *Ar*), 1.21 (s, *Me*), 0.95 (s, *Me*), 0.90 (s, *Me*) ppm. **^{29}Si DEPT90 NMR (79 MHz, 298 K, THF-d_8):** δ = 25.57 (s, Me_3*Si*–O), 25.36 (s, Me_3*Si*–O), 14.23 (s, Me_3*Si*–N), 13.95 (s, Me_3*Si*–N), 13.89 (s, Me_3*Si*–N), 13.62 (s, Me_3*Si*–N) ppm. **^{31}P NMR (162 MHz, 298 K, THF-d_8):** δ = 141.20 - 127.93 (m, 4P, *bridging A and B*), 127.60 - 116.10 (m, 3P, *bridging B*), 108.25 - 97.42 (m, 3P, *bridging B*), 92.33 - 81.72 (m, 2P, *bridging B*), –118.96 - –134.66 (m, 4P, *apical A and B*), –170.26 - –183.86 (m, 12P, *basal A and B*) ppm.

Mass spectrometry (ESI neg/pos): $C_{30}H_{39}Br_3N_3O_3P_7Si_3$+Na ([M+Na]$^+$): calcd.: 1053.7870; found: 1053.7849.

*Note: N,N,O-coordination of the silyl units is assigned based on the ^1H NMR data. Compound **3** decomposed while weighing for elemental analysis.*

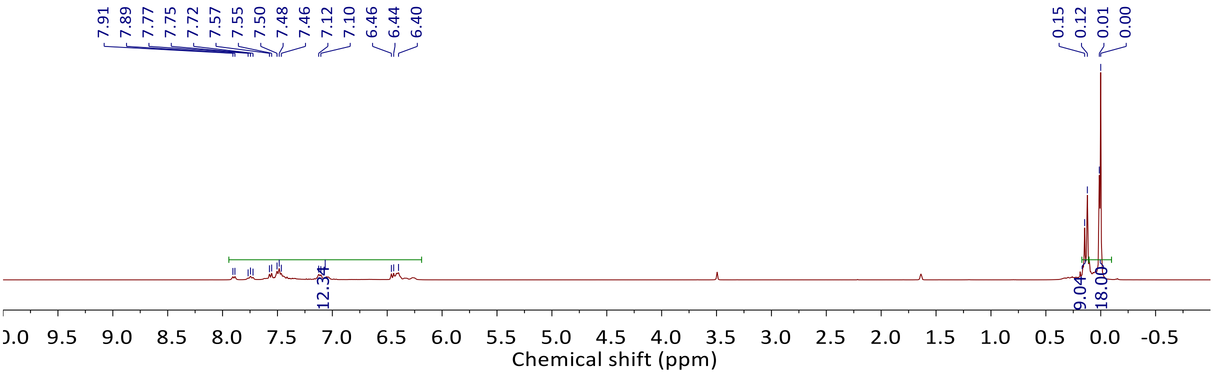

Figure S23. ^1H NMR spectrum (THF-d_8) of **3**.

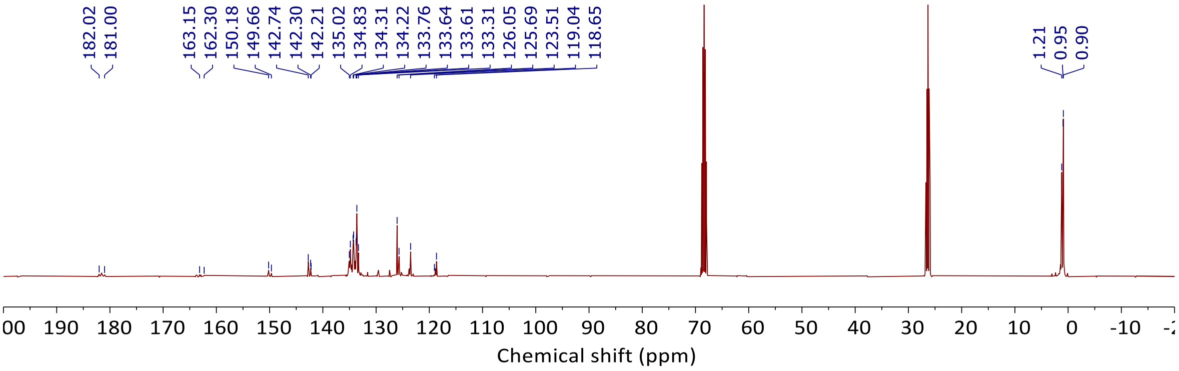

Figure S24. $^{13}C\{^1H\}$ NMR spectrum (THF-d_8) of **3**.

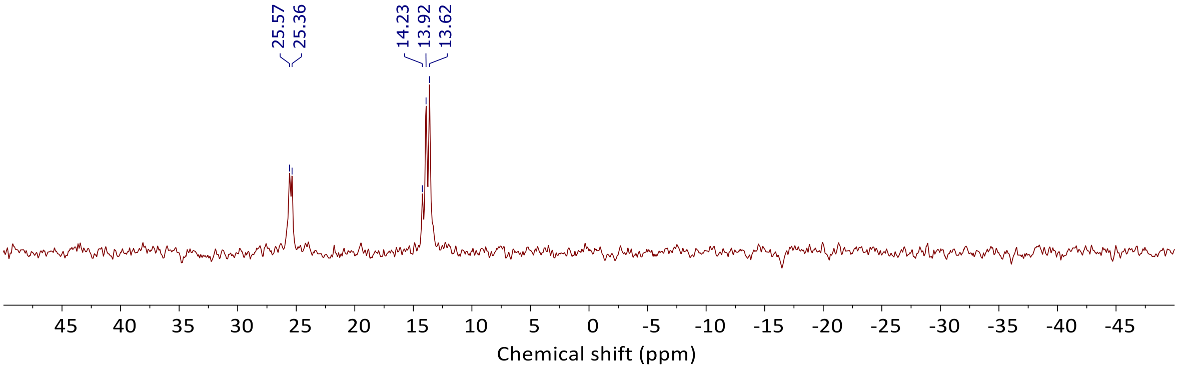

Figure S25. ^{29}Si DEPT90 NMR spectrum (THF-d_8) of **3**.

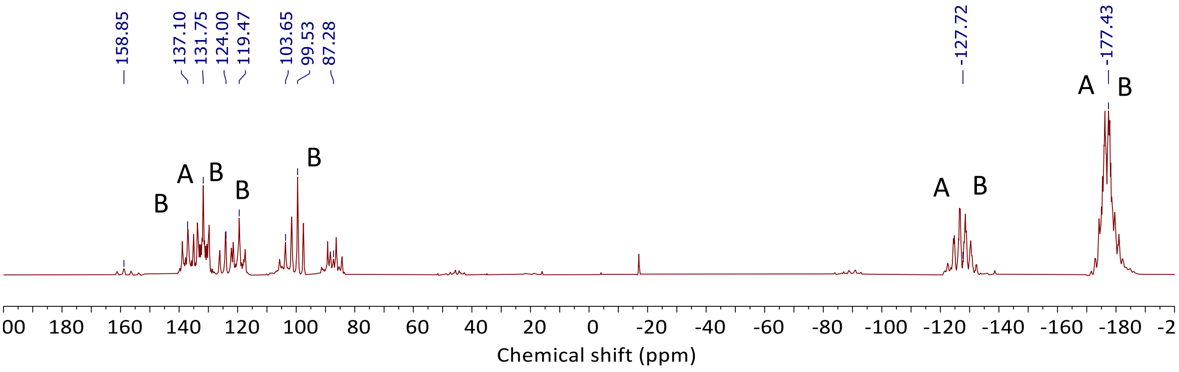

Figure S26. ^{31}P NMR spectrum (THF-d_8) of **3**.

2.2.4. Synthesis (TMS-F(C_6H_4)NCO)$_3$$P_7$ (4)

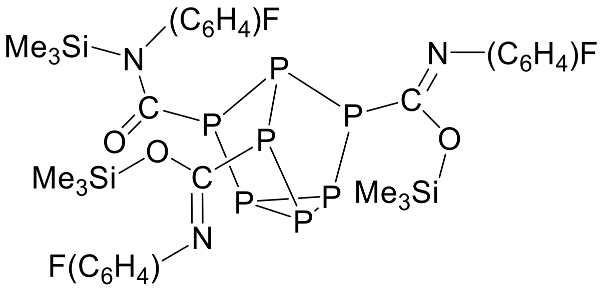

To a J young ampoule charged with a stir bar and $(Me_3Si)_3P_7$ (**1**) (100 mg, 0.23 mmol, 1.0 eq.) THF (0.4 mL) was added. To a separate vial, 4-F(C_6H_4)NCO (95 mg, 0.69 mmol, 3.0 eq.) and THF (0.4 mL) was added. The solution of 4-F(C_6H_4)NCO was added to the $(Me_3Si)_3P_7$ solution and allowed to react overnight at room temperature. The volatiles were removed yielding an oily solid.

Isolated Yield: 132 mg, 68%.

^{1}H NMR (400 MHz, 298 K, THF-d$_8$): δ = 7.71 - 6.30 (m, 12H, *Ar*), 0.11 - –0.07 (overlapping singlets, 18H, *Me*), –0.07 - –0.27 (overlapping singlets, 9H, *Me*) ppm. **^{13}C{^{1}H} NMR (101 MHz, 298 K, THF-d$_8$):** δ = 182.29 (d, $^1J_{CP}$ = 52 Hz, (TMS-F(C_6H_4)N*C*O)$_3$$P_7$), 165.84 (s, *Ar*), 165.53 (s, *Ar*), 163.08 (s, *Ar*), 160.84 (s, *Ar*), 147.39 (s, *Ar*), 139.73 (s, *Ar*), 139.31 (s, *Ar*), 138.43 (s, *Ar*), 134.88 (s, *Ar*), 129.65 (s, *Ar*), 127.56 (s, *Ar*), 125.40 (s, *Ar*), 118.51 - 116.34 (multiple overlapping singlets, *Ar*), 1.44 (s, *Me*), 1.30 (s, *Me*), 0.90 (s, *Me*) ppm. **^{19}F NMR (376 MHz, 298 K, THF-d$_8$):** δ = –113.70 (s, 1F, *F*–Ar), –114.08 (s, 1F, *F*–Ar), –114.14 (s, 1F, *F*–Ar), –114.42 (s, 1F, *F*–Ar), –114.72 (s, 1F, *F*–Ar), –114.91 (s, 3F, *F*–Ar), –120.40 (s, 1F, *F*–Ar), –120.68 (s, 1F, *F*–Ar), –120.78 (overlapping singlets, 1F, *F*–Ar), –121.04 (s, 1F, *F*–Ar) ppm. **^{29}Si DEPT90 NMR (79 MHz, 298 K, THF-d$_8$):** δ = 25.42 (s, Me_3*Si*–O), 25.19 (s, Me_3*Si*–O), 13.82 (s, Me_3*Si*–N), 13.51 (s, Me_3*Si*–N) ppm. **^{31}P NMR (162 MHz, 298 K, THF):** δ = 156.37 - 149.19 (m, 2P, *bridging B*), 139.19 - 132.84 (m, 1P, *bridging B*), 132.84 - 126.02 (m, 3P, *bridging A*), 124.38 - 112.85 (m, 2P, *bridging B*), 111.67 - 95.21(m, 2P, *bridging B*), 85.33 - 80.04 (m, 2P, *bridging B*), –120.02 - –135.20 (m, 4P, *apical A and B*), –164.72 - –187.18 (m, 12P, *basal B*), ppm.

Mass spectrometry (ESI neg/pos): $C_{30}H_{39}F_3N_3O_3P_7Si_3$+Na ($[M+Na]^+$): calcd.: 870.0307; found: 870.0280.

Elemental analysis for $C_{30}H_{39}F_3N_3O_3P_7Si_3$: calcd.: C 42.51, H 4.64, N 4.96; found: C 43.09, H 4.33, N 4.46.

Note: N,O,O-coordination of the silyl units is assigned based on the ^{1}H NMR data.

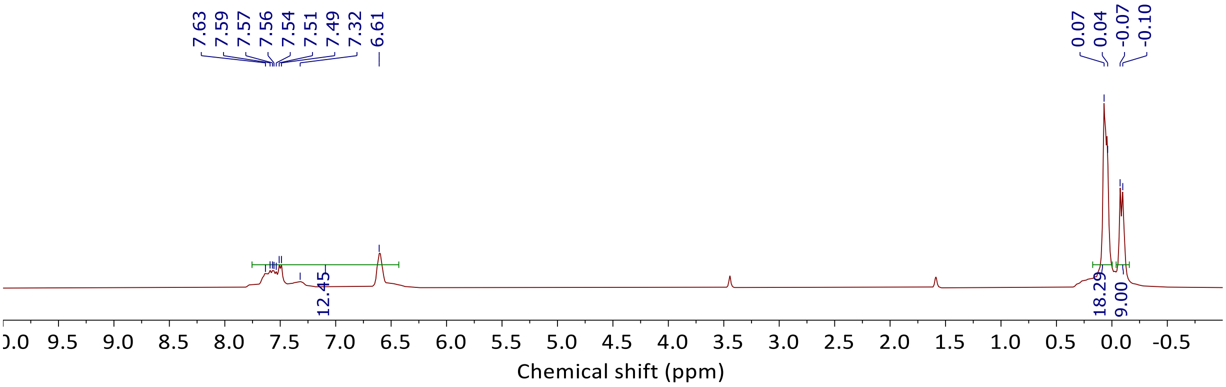

Figure S27. ^{1}H NMR spectrum (THF-d_8) of **4**.

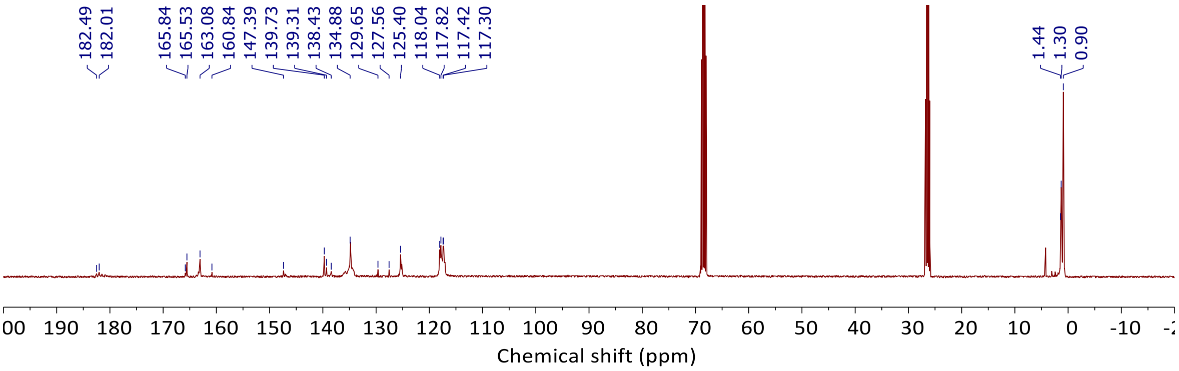

Figure S28. $^{13}C\{^1H\}$ NMR spectrum (THF-d_8) of **4**.

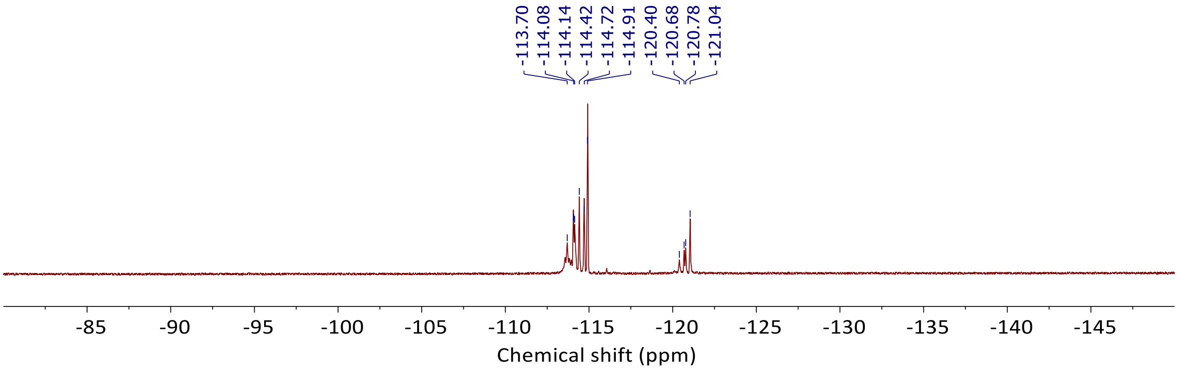

Figure S29. ^{19}F NMR spectrum (THF-d_8) of **4**.

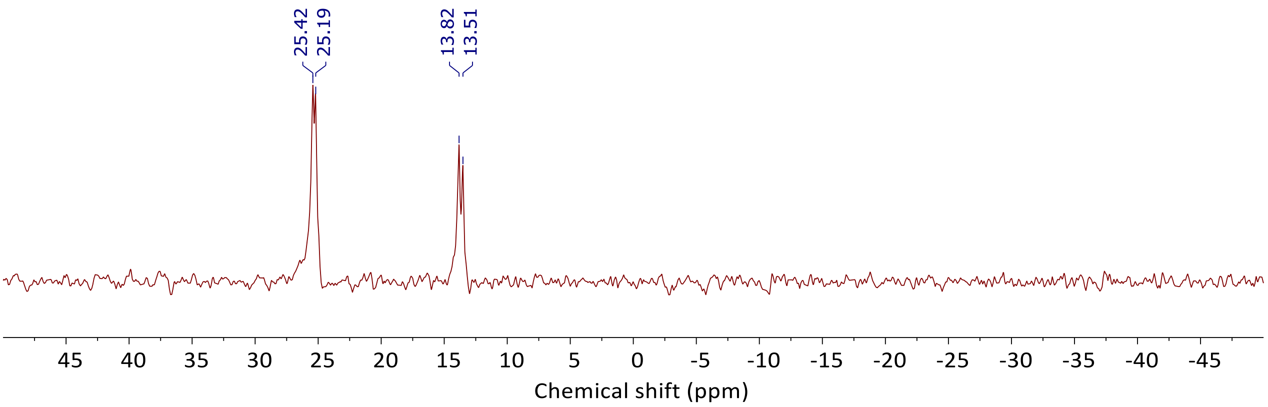

Figure S30. ^{29}Si DEPT90 NMR spectrum (THF-d_8) of **4**.

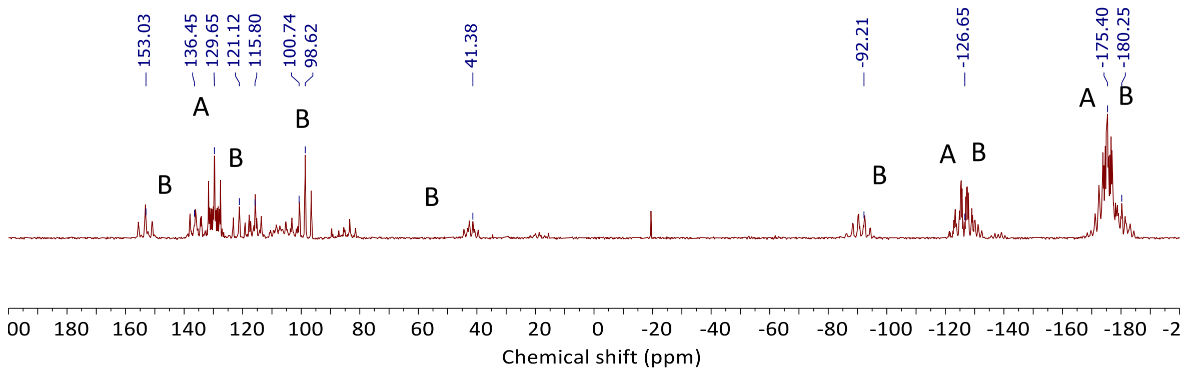

Figure S31. ^{31}P NMR spectrum (THF-d_8) of **4**.

2.2.5. Synthesis (TMS-CF$_3$(C$_6$H$_4$)NCO)$_3$P$_7$ (5)

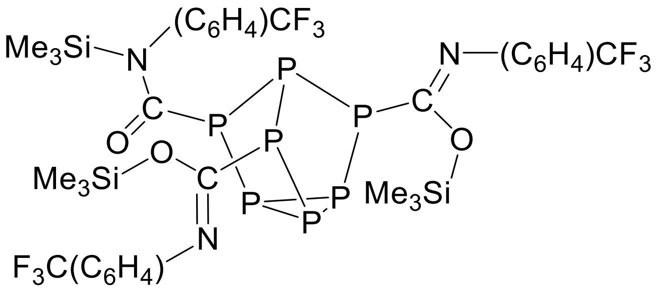

To a J young ampoule charged with a stir bar and $(Me_3Si)_3P_7$ (**1**) (100 mg, 0.23 mmol, 1.0 eq.) THF (0.4 mL) was added. To a separate vial, 4-$CF_3(C_6H_4)NCO$ (129 mg, 0.69 mmol, 3.0 eq.) and THF (0.4 mL) was added. The solution of 4-$CF_3(C_6H_4)NCO$ was added to the $(Me_3Si)_3P_7$ solution and allowed to react overnight at room temperature. The volatiles were removed yielding an oily solid.

Isolated Yield: 142 mg, 62%.

^{1}H NMR (400 MHz, 298 K, THF-d$_8$): δ = 7.97 - 6.60 (m, 12H, *Ar*), 0.34 – 0.23 (overlapping singlets, 18H, *Me*), 0.23 - –0.07 (overlapping singlets, 9H, *Me*) ppm. **^{13}C{^{1}H} NMR (101 MHz, 298 K, THF-d$_8$):** δ = 181.84 - 179.84 (m, (TMS-$CF_3(C_6H_4)N$*C*$O)_3P_7$), 163.28 (s, *Ar*), 162.66 (s, *Ar*), 161.88 (s, *Ar*), 153.86 (s, *Ar*), 153.37 (s, *Ar*), 146.83 (s, *Ar*), 146.45 (s, *Ar*), 133.35 (s, *Ar*), 132.25 (s, *Ar*), 128.30 (s *Ar*), 127.81 (s *Ar*), 127.76 (s *Ar*), 124.17 (s, *Ar*), 123.85 (s, *Ar*), 0.95 (s *Me*), 0.90 (s, *Me*), 0.56 (s *Me*), 0.52 (s *Me*) ppm. **^{19}F NMR (376 MHz, 298 K, THF-d$_8$):** δ = –62.15 (s, 3F, C*F_3*–Ar), –62.24 (s, 9F, C*F_3*–Ar), –62.45 (s, 9F, C*F_3*–Ar), –62.47 (s, 3F, C*F_3*–Ar F), –62.56 (s, 3F, C*F_3*–Ar), –62.64 (s, 3F, C*F_3*–Ar), –62.96 (s, 3F, C*F_3*–Ar), –62.99 (s, 3F, C*F_3*–Ar) ppm. **^{29}Si DEPT90 NMR (79 MHz, 298 K, THF-d$_8$):** δ = 26.45 (s, Me_3*Si*–O), 26.22 (s, Me_3*Si*–O), 25.98 (s, Me_3*Si*–O), 14.84 (s, Me_3*Si*–N), 14.54 (s, Me_3*Si*–N), 14.27 (s, Me_3*Si*–N) ppm. **^{31}P NMR (162 MHz, 298 K, THF-d$_8$):** δ = 141.87 - 126.66 (m, 1P, *bridging B*), 127.37 - 116.52 (m, 3P, *bridging A*), 103.91 - 94.12 (m, 4P, *bridging B*), 92.59 - 80.32 (m, 4P, *bridging B*), –113.17 - –131.21 (m, 4P, *apical A and B*), –166.00 - –181.21 (m, 12P, *basal A and B*) ppm.

Mass spectrometry (ESI neg/pos): $C_{33}H_{39}F_9N_3O_3P_7Si_3$+Na ($[M+Na]^+$): calcd.: 1020.0211; found: 1020.0199.

*Note: N,O,O-coordination of the silyl units is assigned based on the ^{1}H NMR data. Compound **5** decomposed while weighing for elemental analysis.*

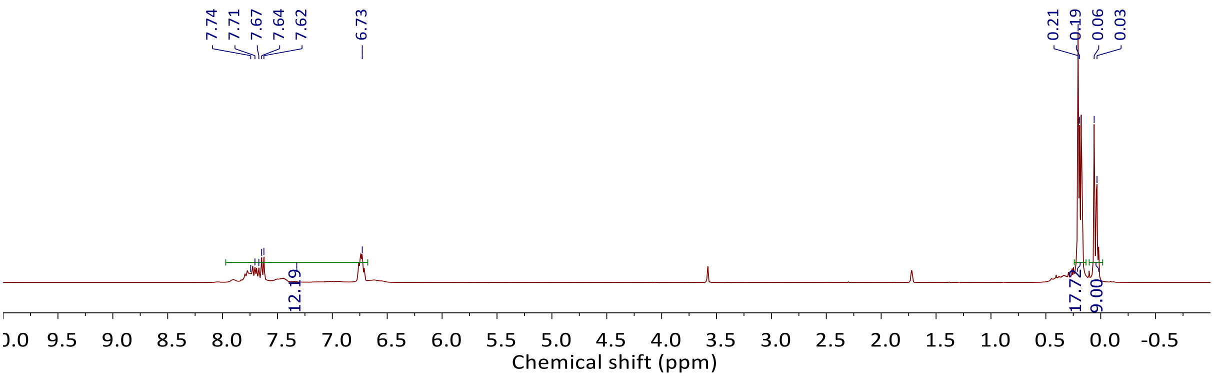

Figure S32. ^{1}H NMR spectrum (THF-d$_8$) of **5**.

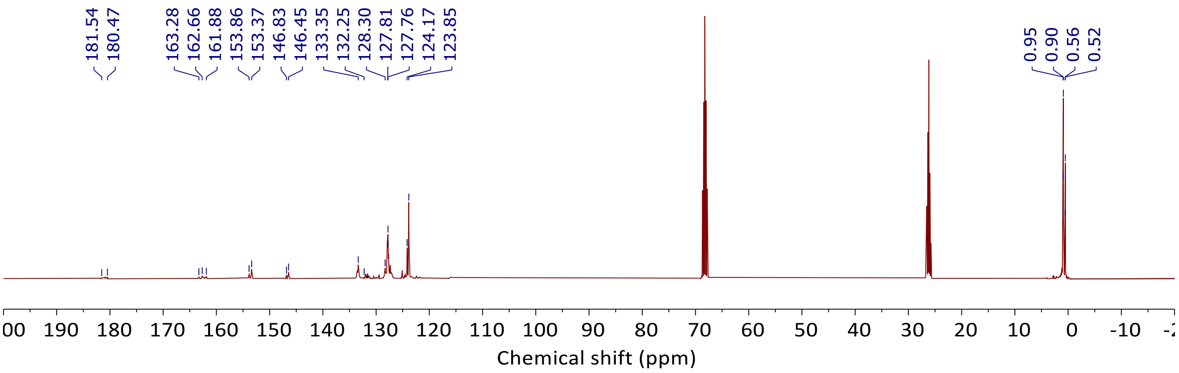

Figure S33. $^{13}C\{^{1}H\}$ NMR spectrum (THF-d$_8$) of **5**.

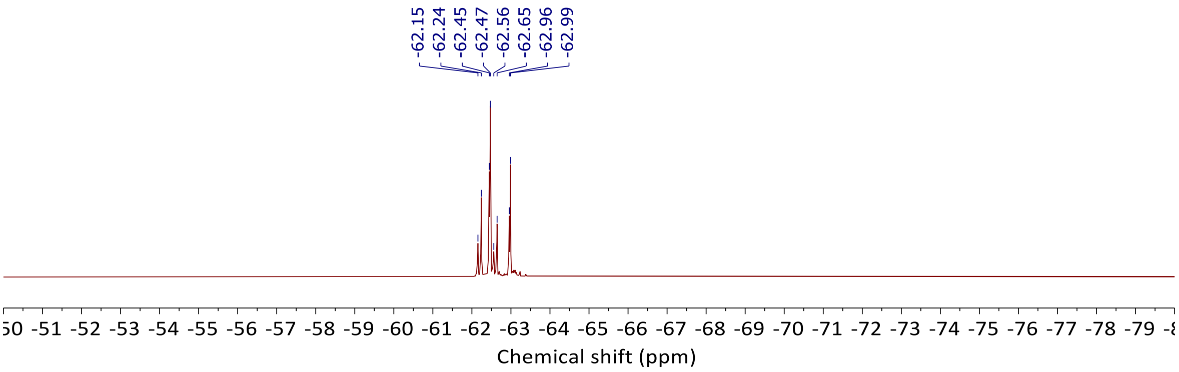

Figure S34. ^{19}F NMR spectrum (THF-d$_8$) of **5**.

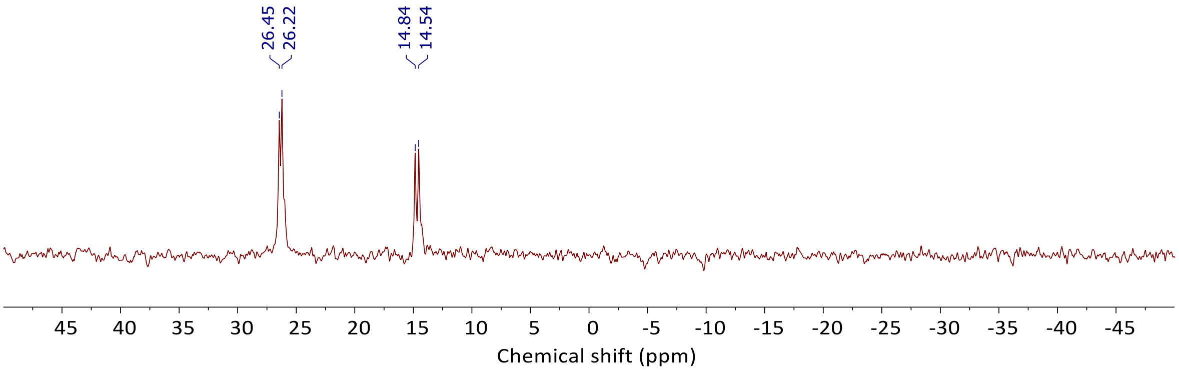

Figure S35. ^{29}Si DEPT90 NMR spectrum (THF-d$_8$) of **5**.

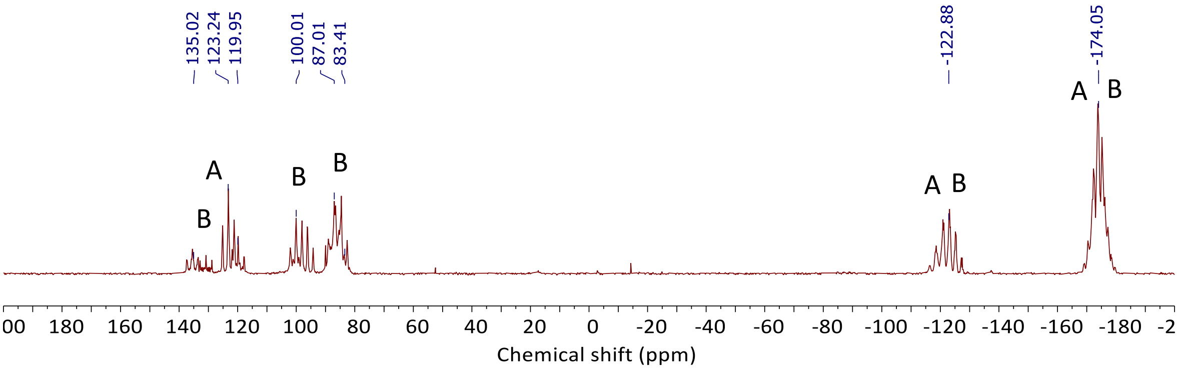

Figure S36. ^{31}P NMR spectrum (THF-d$_8$) of **5**.

2.2.6. Synthesis $(TMS\text{-}TolylNCO)_3P_7$ (6)

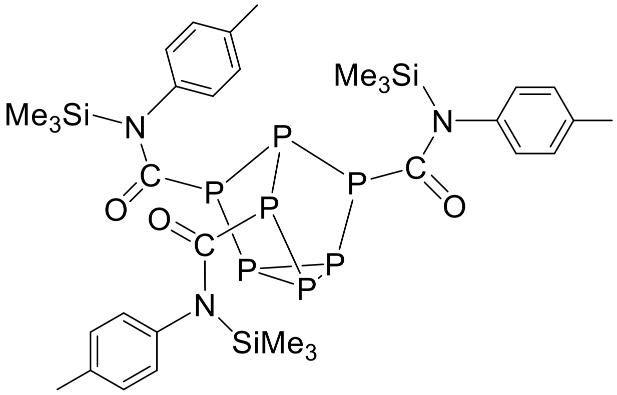

To a J young ampoule charged with a stir bar and $(Me_3Si)_3P_7$ (**1**) (100 mg, 0.23 mmol, 1.0 eq.) THF (0.4 mL) was added. To a separate vial, p-tolylNCO (912 mg, 0.69 mmol, 3.0 eq.) and THF (0.4 mL) was added. The solution of p-tolylNCO was added to the $(Me_3Si)_3P_7$ solution and allowed to react overnight at room temperature. The volatiles were removed yielding a glassy solid. The glassy solid was dissolved in pentane (5 mL) and cooled to –20 °C. Block shaped crystals formed, the mother liquor was removed. The crystals were washed with pentane and the solvent removed, yielding a crystalline solid. Crystals suitable for single crystals X-ray diffraction analysis were obtained through cooling down a concentrated pentane solution.
Isolated Yield: 102 mg, 53%.

^{1}H NMR (400 MHz, 298 K, THF-d_8): δ = 7.64 - 6.83 (m, 12H, *Ar*), 2.48 (s, 9H, *Me*), 0.00 (s, 27H, Si-*Me*) ppm. **^{13}C{^{1}H} NMR (101 MHz, 298 K, THF-d_8):** δ = 182.47 (d, $^{1}J_{CP}$ = 47 Hz, $(TMS\text{-}TolylNCO)_3P_7$), 140.89 (s, *Ar*), 139.38 (s, *Ar*), 133.18 (s, *Ar*), 132.54 (s, *Ar*), 131.62 (s, *Ar*), 131.02 (s, *Ar*), 130.70 (s, *Ar*), 123.74 (s, *Ar*), 22.30 (s, *Me*), 0.85 (s, Si-*Me*) ppm. **^{29}Si DEPT90 NMR (79 MHz, 298 K, THF-d_8):** δ = 12.65 (s, Me_3*Si*–N), 12.39 (s, Me_3*Si*–N) ppm. **^{31}P NMR (162 MHz, 298 K, THF-d_8):** δ = 161.27 - 151.91 (m, 1P, *bridging B*), 143.44 - 126.36 (m, 3P, *bridging A*), 121.68 - 109.66 (m, 1P, *bridging B*), 102.95 - 92.45 (m, 1P, *bridging B*), –118.94 - –132.48 (m, 2P, *apical A and B*), –171.32 - –183.84 (m, 6P, *basal A and B*) ppm.
Mass spectrometry (ESI neg/pos): $C_{33}H_{48}N_3O_3P_7Si_3$+Na ($[M+Na]^+$): calcd.: 858.1059; found: 858.1050.

Elemental analysis for $C_{33}H_{48}N_3O_3P_7Si_3$: calcd.: C 47.42, H 5.79, N 5.03; found: C 47.42, H 5.78, N 4.97.

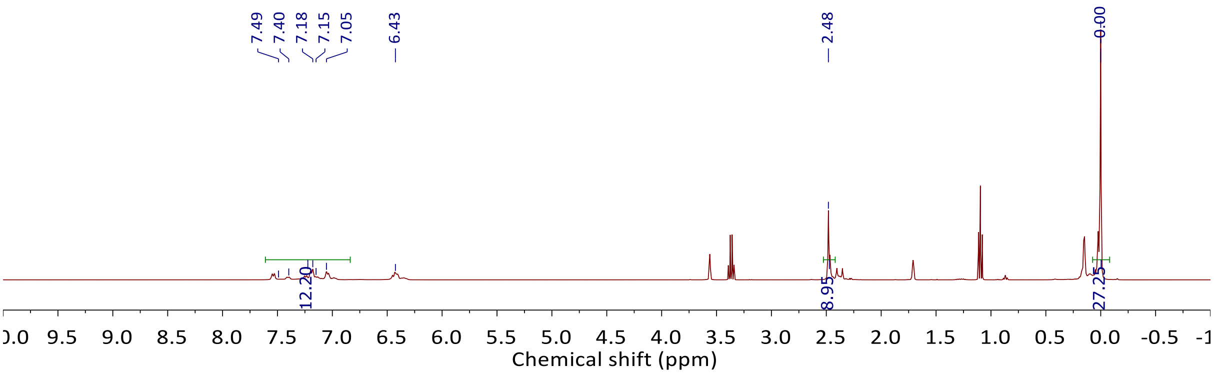

Figure S37. ^{1}H NMR spectrum (THF-d_8) of **6**.

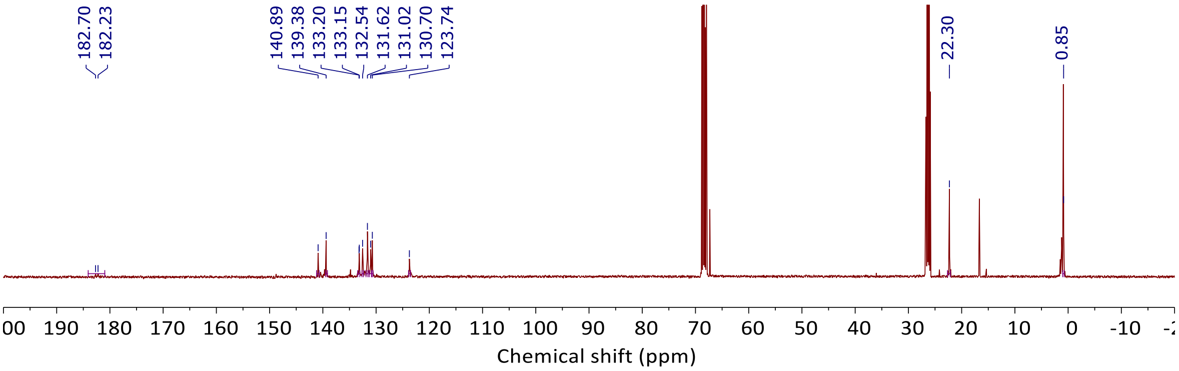

Figure S38. $^{13}C\{^{1}H\}$ NMR spectrum (THF-d_8) of **6**.

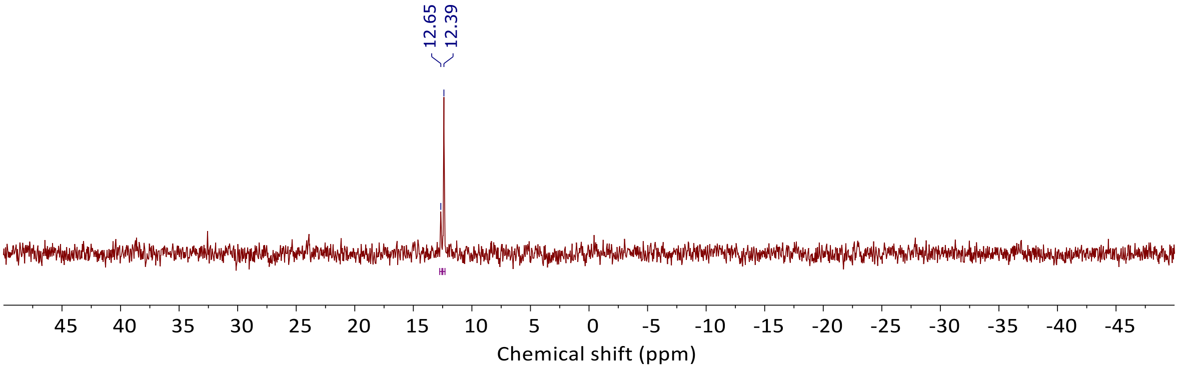

Figure S39. ^{29}Si DEPT90 NMR spectrum (THF-d_8) of **6**.

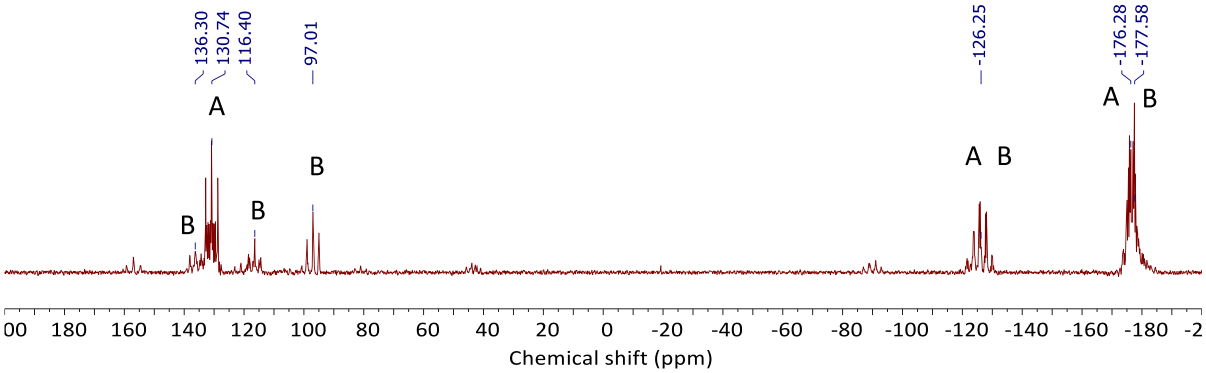

Figure S40. ^{31}P NMR spectrum (THF-d_8) of **6**.

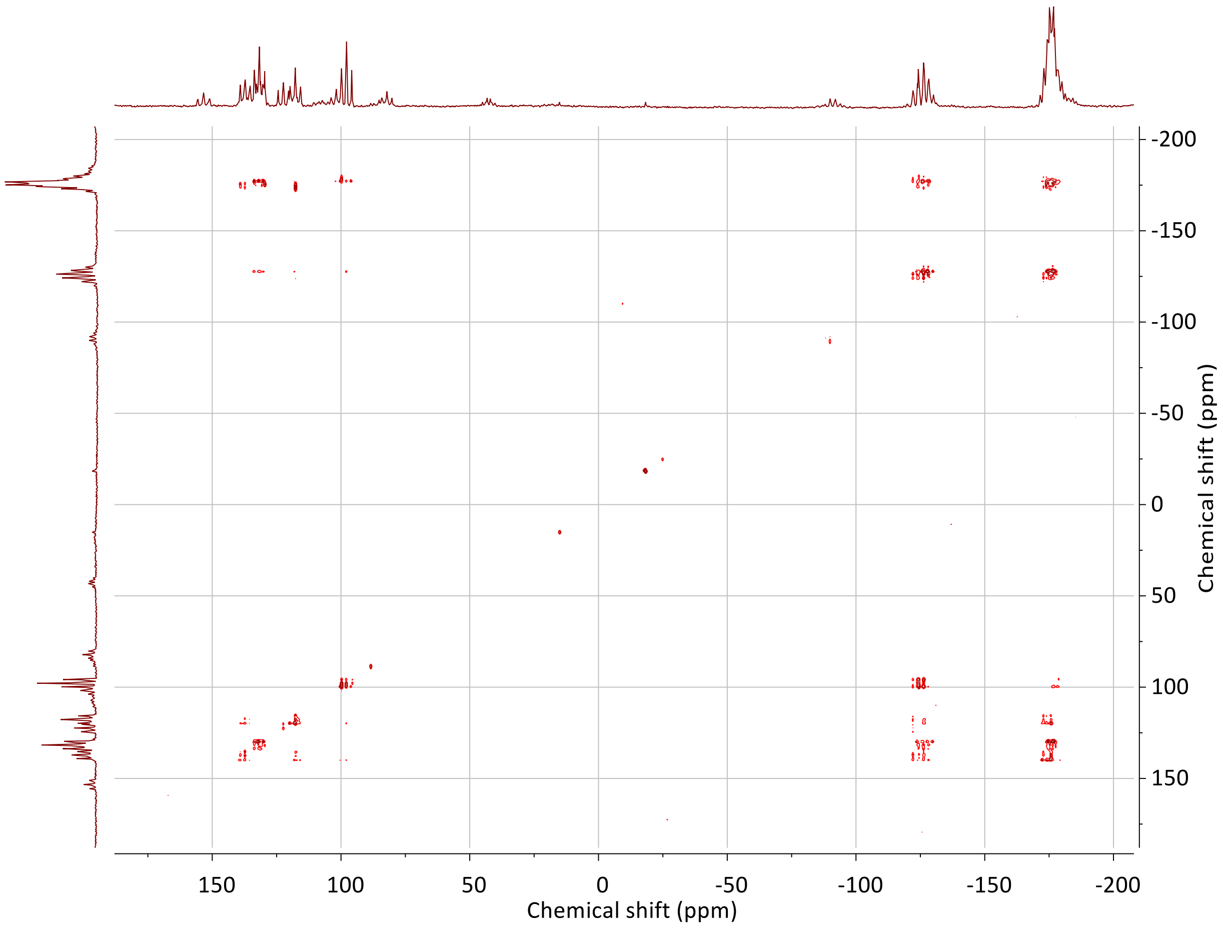

Figure S41. ^{31}P COSY NMR spectrum (THF-d_8) of **6**.

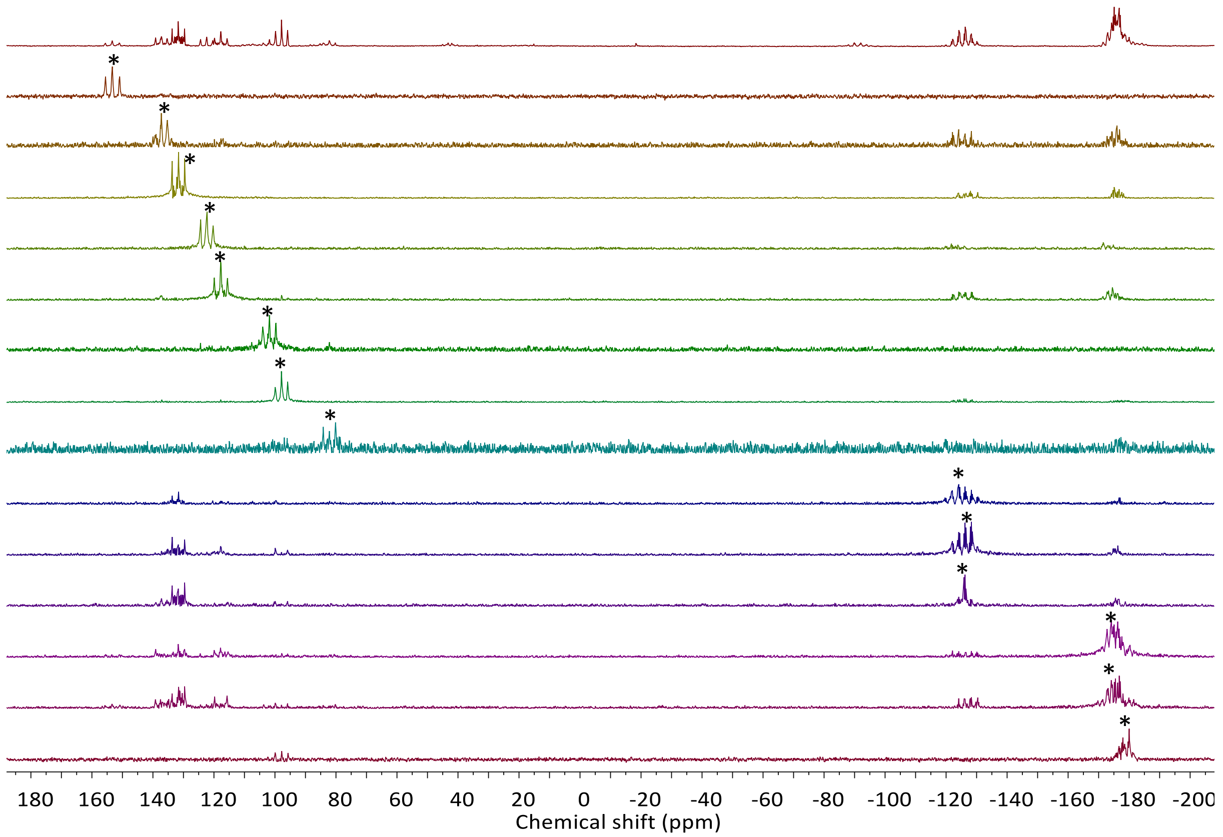

Figure S42. ^{31}P 1D selective COSY NMR spectra of **6**. Top spectrum ^{31}P NMR followed by selective radiation (marked by *) of resonances at 153.32, 137.19, 131.67, 122.41, 117.75, 101.86, 97.90, 84.84, 82.23, 42.59, –90.90, –123.40, –126.17, –128.34, –173.94, –175.94, and –179.96 ppm. Optimised for 330 Hz coupling.

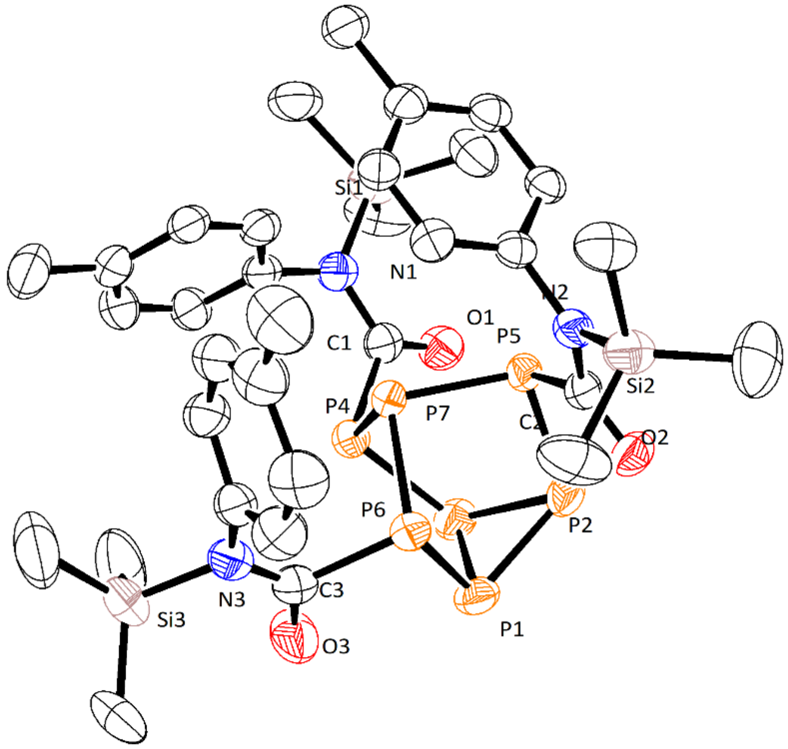

Figure S43. Molecular structure of **6**. Anisotropic displacement ellipsoids pictured at 25% probability. Hydrogen atoms omitted for clarity. Phosphorus: orange; Nitrogen: blue; Oxygen: red; Silicon: pink; Carbon: white.

2.2.7. Synthesis (TMS-MeO(C_6H_4)NCO)$_3$$P_7$ (7)

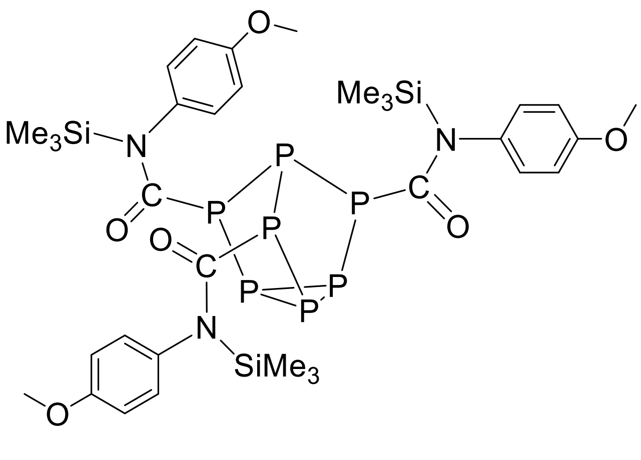

To a J young ampoule charged with a stir bar and $(Me_3Si)_3P_7$ (**1**) (100 mg, 0.23 mmol, 1.0 eq.) THF (0.4 mL) was added. To a separate vial, 4-MeO(C_6H_4)NCO (103 mg, 0.69 mmol, 3.0 eq.) and THF (0.4 mL) was added. The solution of 4-MeO(C_6H_4)NCO was

added to the $(Me_3Si)_3P_7$ solution and allowed to react overnight at room temperature. The volatiles were removed yielding a glassy solid. The glassy solid was partially dissolved in pentane (5 mL) and cooled to –20 °C. Block shaped crystals formed, the mother liquor were removed. The crystals were washed with pentane and solvent removed, yielding a crystalline solid. Crystals suitable for single crystals X-ray diffraction analysis were obtained through cooling down a concentrated pentane solution.

Isolated Yield: 31 mg, 15%.

^{1}H NMR (400 MHz, 298 K, THF-d_8): δ = 7.39 - 6.41 (m, 12H, *Ar*), 4.12 - 3.74 (overlapping singlets, 9H, O*Me*), 0.45 - –0.19 (overlapping singlets, 27H, *Me*) ppm. **^{13}C{^{1}H} NMR (101 MHz, 298 K, THF-d_8):** δ = 182.60 (d, $^1J_{CP}$ = 45 Hz, (TMS-MeO(C_6H_4)NCO$)_3P_7$), 161.06 (s, *Ar*), 135.76 (s, *Ar*), 134.13 (s, *Ar*), 133.36 (s, *Ar*), 124.49 (s, *Ar*), 116.10 (s, *Ar*), 115.54 (s, *Ar*), 114.54 (s, *Ar*), 56.54 (s, O*Me*), 0.68 (s, *Me*) ppm. **^{29}Si DEPT90 NMR (79 MHz, 298 K, THF-d_8):** δ = 12.32 (s, Me_3*Si*–N). **^{31}P NMR (162 MHz, 298 K, THF-d_8):** δ = 141.20 - 134.85 (m, 1P, *bridging B*), 134.85 - 125.87 (m, 6P, *bridging A*), 119.11 - 112.34 (m, 1P, *bridging B*), 102.54 - 94.53 (m, 1P, *bridging B*), –119.78 - –133.31 (m, 3P, *apical A and B*), –170.59 - –189.23 (m, 9P, *basal A and B*) ppm.

Mass spectrometry (ESI neg/pos): $C_{33}H_{48}N_3O_6P_7Si_3$+Na ($[M+Na]^+$): calcd.: 906.0906; found: 906.0904.

Elemental analysis for $C_{33}H_{48}N_3O_6P_7Si_3$: calcd.: C 44.85, H 5.47, N 4.75; found: C 45.18, H 5.46, N 4.77.

Note: the ^{31}P NMR spectrum shows a 1:2 asym:sym mixture, therefore the total integration is 21 phosphorus atoms.

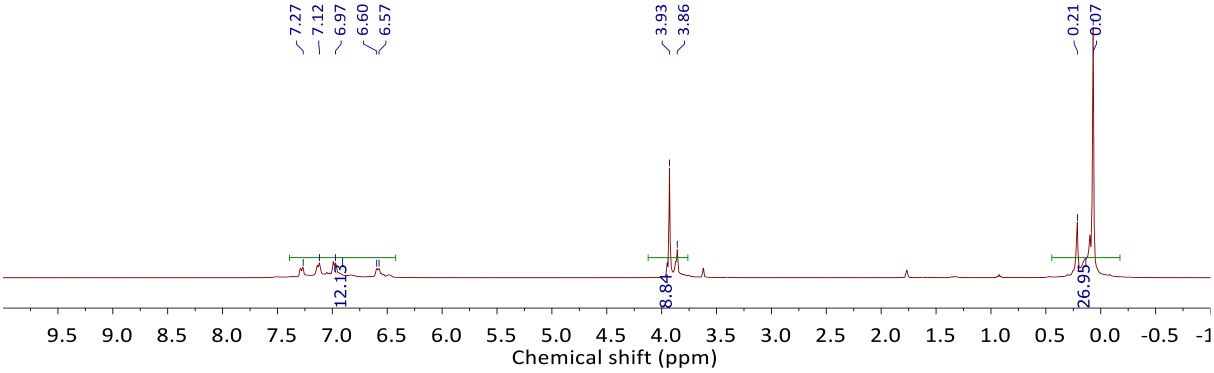

Figure S44. ^{1}H NMR spectrum (THF-d_8) of **7**.

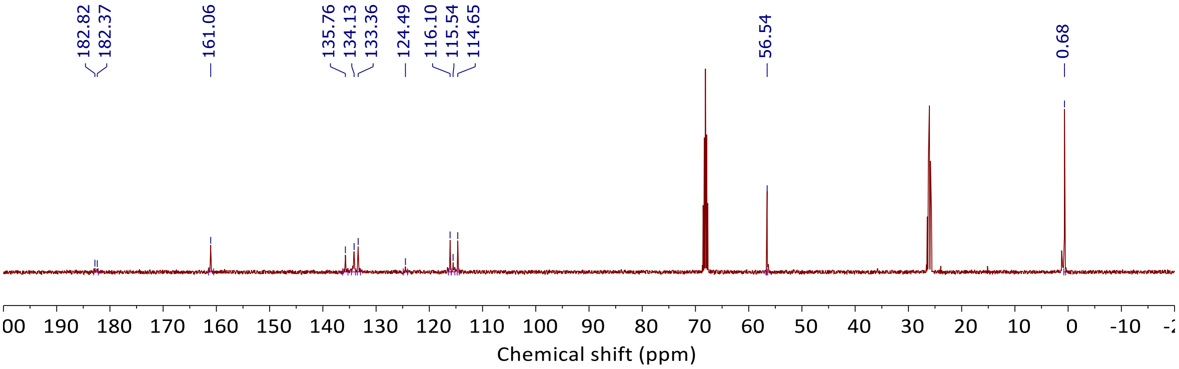

Figure S45. $^{13}C\{^{1}H\}$ NMR spectrum (THF-d_8) of **7**.

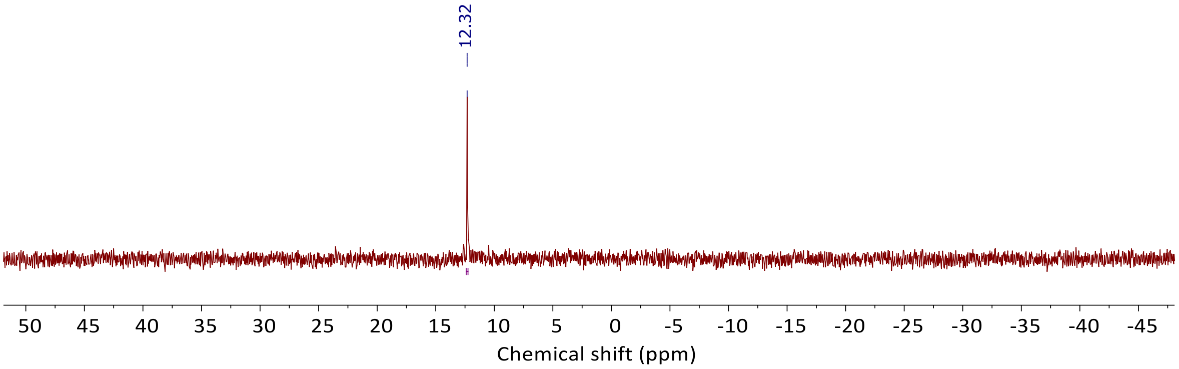

Figure S46. ^{29}Si DEPT90 NMR spectrum (THF-d_8) of **7**.

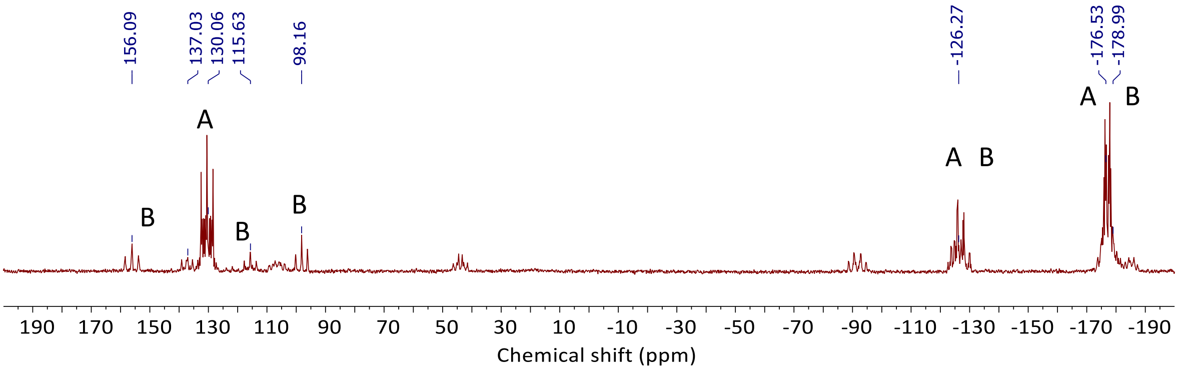

Figure S47. ^{31}P NMR spectrum (THF-d_8) of **7**.

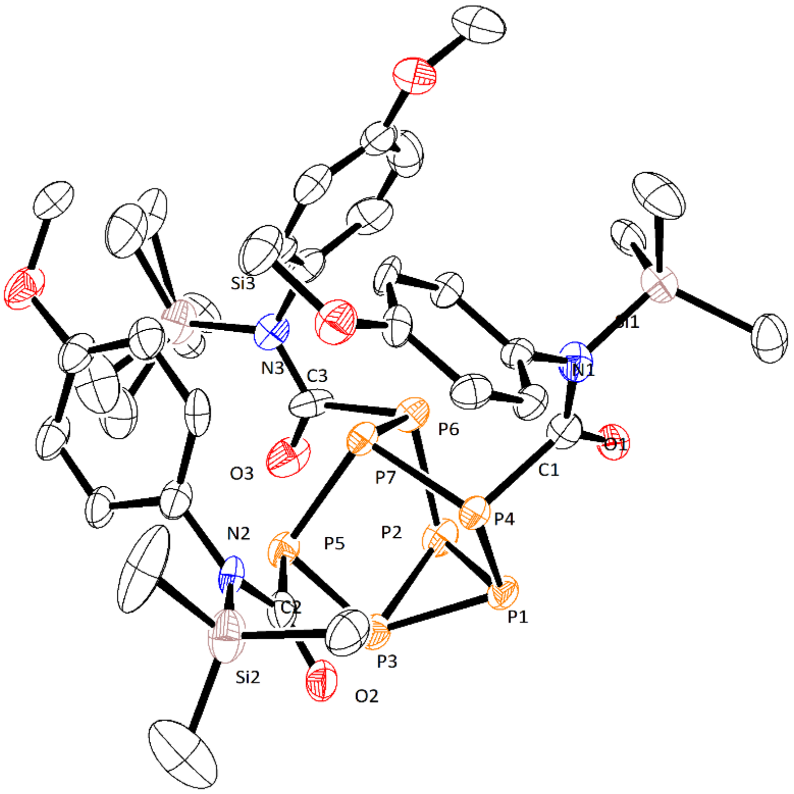

Figure S48. Molecular structure of **7**. Anisotropic displacement ellipsoids pictured at 50% probability. Hydrogen atoms omitted for clarity. Phosphorus: Orange; Silicon: Pink; Carbon: White; Oxygen: Red; Nitrogen: Blue.

2.2.8. Synthesis (TMS-PhNCS)$_3$P$_7$ (8)

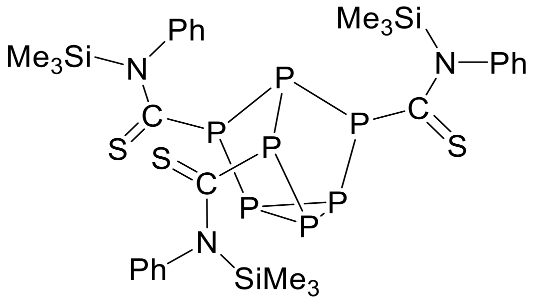

To a J young ampoule charged with a stir bar and (Me$_3$Si)$_3$P$_7$ (**1**) (100 mg, 0.23 mmol, 1.0 eq.) THF (0.4 mL) was added. To a separate vial, PhNCS (93 mg, 0.69 mmol, 3.0 eq.) and THF (0.4 mL) was added. The solution of PhNCS was added to the (Me$_3$Si)$_3$P$_7$ solution and allowed to react overnight at room temperature, the colour changed from yellow to orange. The volatiles were removed yielding a glassy solid. The glassy solid

was washed with Et_2O (3 x 5 mL) and dried under reduced pressure, yielding an orange powder. Crystals suitable for single crystals X-ray diffraction analysis were obtained through slow evaporation of a concentrated Et_2O solution.

Isolated Yield: 169 mg, 87%.

^{1}H NMR (400 MHz, 298 K, THF-d$_8$): δ = 7.98 - 6.65 (m, 15H, *Ar*), 0.25 (s, 27H, *Me*) ppm. **^{13}C{^{1}H} NMR (101 MHz, 298 K, THF-d$_8$):** δ = 217.80 - 214.78 (m, (TMS-PhN*C*S)$_3$P$_7$), 146.80 (s, *Ar*), 146.24 (s, *Ar*), 144.89 (s, *Ar*), 130.74 (s, *Ar*), 130.32 (s, *Ar*), 130.02 (s, *Ar*), 129.80 (s, *Ar*), 129.47 (s, *Ar*), 129.16 (s, *Ar*), 128.71 (s, *Ar*), 125.97 (s, *Ar*), 1.21 (s, *Me*), 0.91(s, *Me*) ppm. **^{29}Si DEPT90 NMR (79 MHz, 298 K, THF-d$_8$):** δ = 14.52 (s, Me$_3$*Si*–N), 14.18 (s, Me$_3$*Si*–N), 13.51 (s, Me$_3$*Si*–N) ppm. **^{31}P NMR (162 MHz, 298 K, THF-d$_8$):** δ = 195.90 - 184.80 (m, 1P, *bridging B*), 183.73 - 174.96 (m, 3P, *bridging A*), 141.53 (br, 1P, *bridging B*), 52.40 (br, 1P, *bridging B*), –75.79 - –89.40 (m, 1P, *apical B*), –115.86 (br, 1P, *basal B*), –124.12 - –135.57 (m, 1P, *apical A*), –153.71 (br, 1P, *basal B*), –164.75 - –178.53 (m, 4P, *basal A and B*) ppm.

Mass spectrometry (ESI neg/pos): $C_{30}H_{42}N_3P_7S_3Si_3$+Na ([M+Na]$^+$): calcd.: 863.9904; found: 863.9893.

Elemental analysis for $C_{30}H_{42}N_3S_3P_7Si_3$: calcd.: C 42.80, H 5.03, N 4.99, S 11.42; found: C 42.74, H 5.14, N 4.96, S 11.34.

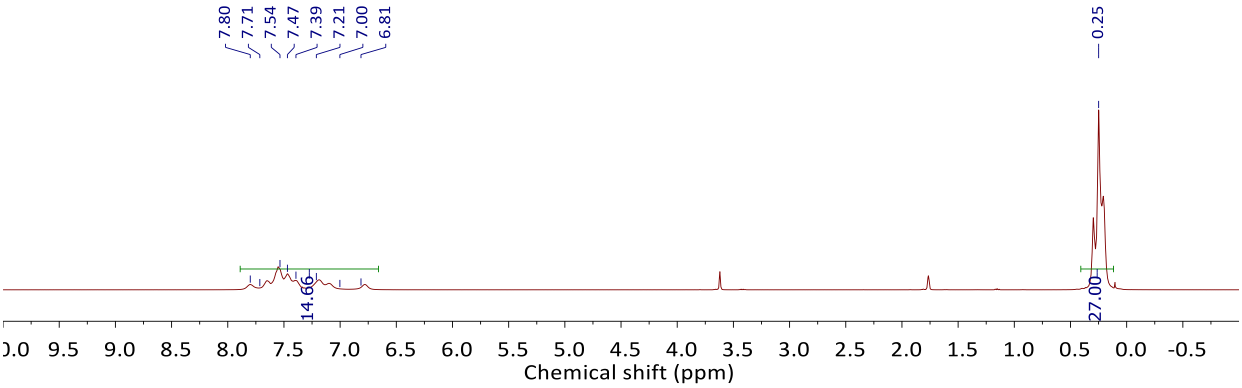

Figure S49. ^{1}H NMR spectrum (THF-d$_8$) of **8**.

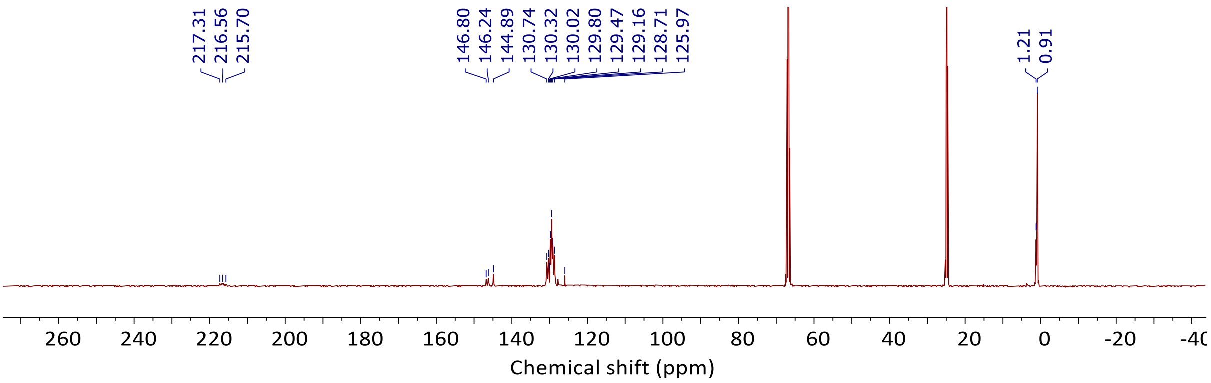

Figure S50. $^{13}C\{^{1}H\}$ NMR spectrum (THF-d8) of **8**.

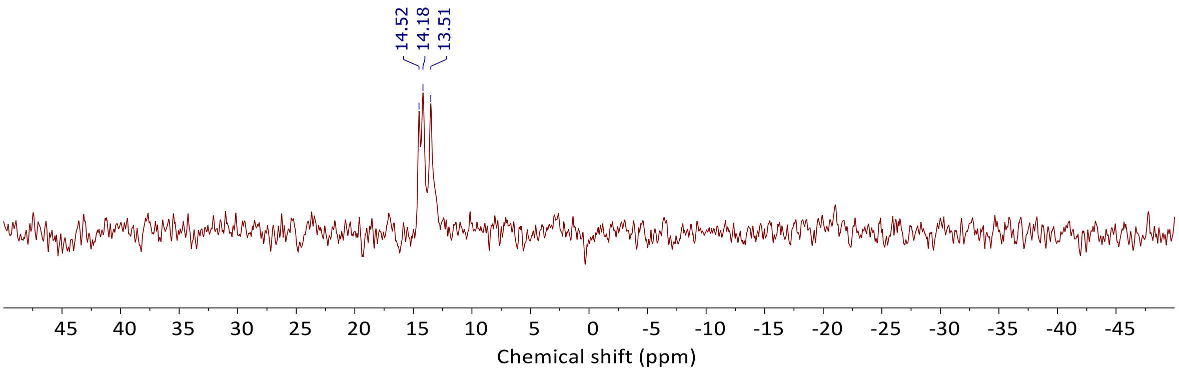

Figure S51. ^{29}Si DEPT90 NMR spectrum (THF-d8) of **8**.

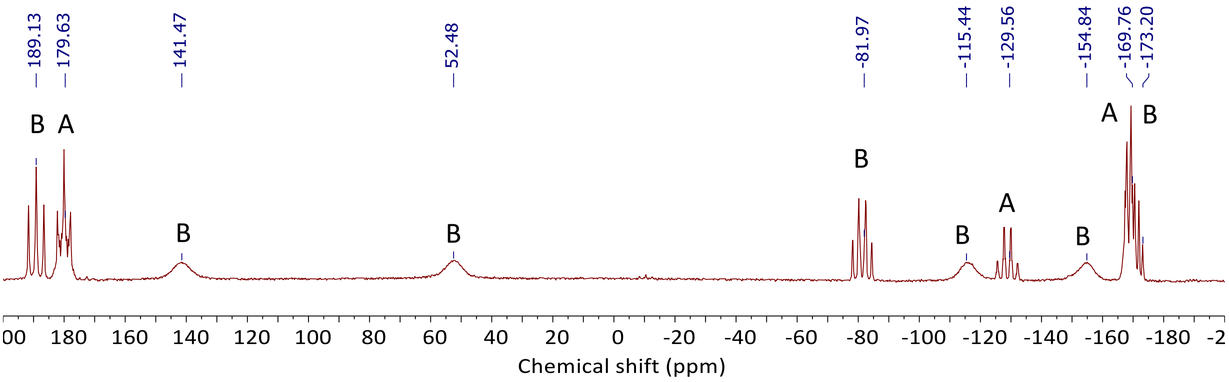

Figure S52. ^{31}P NMR spectrum (THF-d8) of **8**.

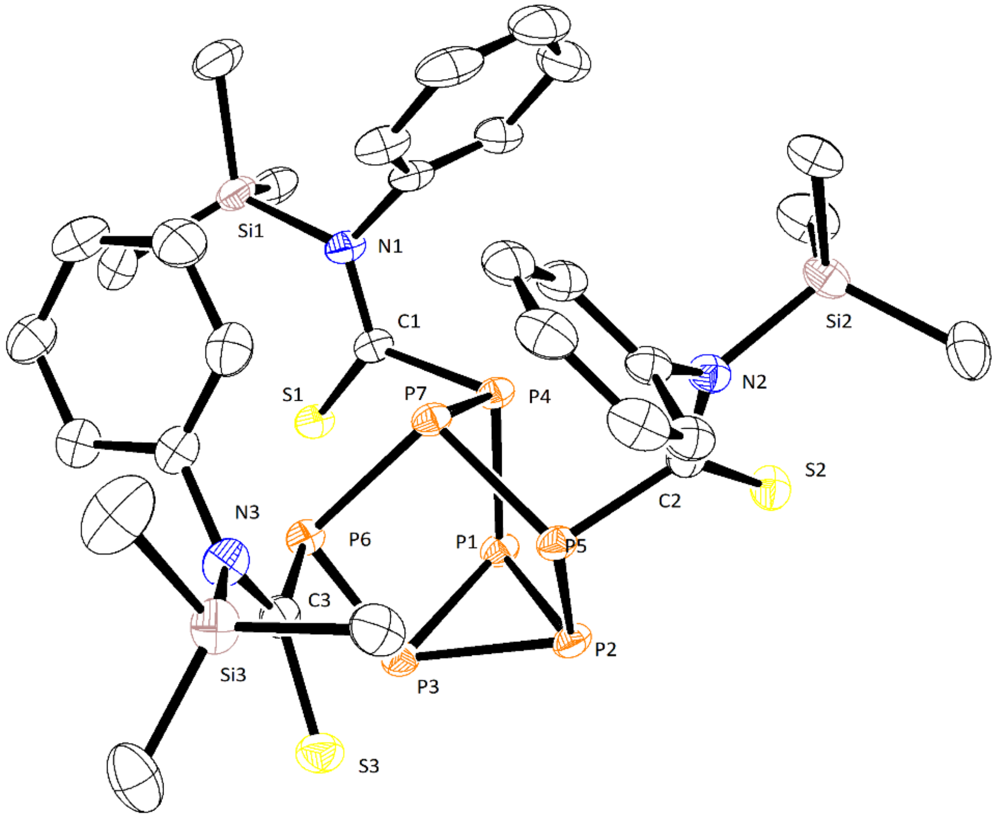

Figure S53. Molecular structure of **8**. Anisotropic displacement ellipsoids pictured at 50% probability. Hydrogen atoms omitted for clarity. Phosphorus: orange; Nitrogen: blue; Silicon: pink; Sulfur: yellow; Carbon: white.

2.2.9. Synthesis (TMS-TosylNCO)$_3$P$_7$ (9)

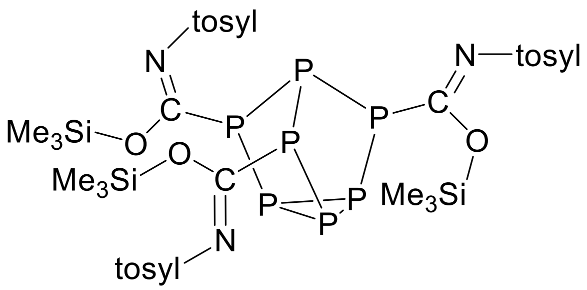

To a J young ampoule charged with a stir bar and $(Me_3Si)_3P_7$ (**1**) (100 mg, 0.23 mmol, 1.0 eq.) THF (0.4 mL) was added. To a separate vial, tosylNCO (136 mg, 0.69 mmol, 3.0 eq.) and THF (0.4 mL) was added. The solution of tosylNCO was added to the $(Me_3Si)_3P_7$ solution and allowed to react overnight at room temperature. The volatiles

were removed yielding a glassy solid. The glassy solid was dissolved in Et_2O (8 mL). Slow evaporation of the solvent yielded block shaped crystals. The crystals were washed with Et_2O (5 mL), yielding a white crystalline solid. Crystals suitable for single crystals X-ray diffraction analysis were obtained through slow evaporation of a concentrated Et_2O solution.

Isolated Yield: 231 mg, 98%.

^{1}H NMR (400 MHz, 298 K, THF-d$_8$): δ = 7.82 (d, $^3J_{HH}$ = 8.3 Hz, 6H, *Ar*), 7.32 (d, $^3J_{HH}$ = 8.2 Hz, 6H, *Ar*), 2.39 (s, 9H, *Me*), 0.28 (s, 27H, Si–*Me*) ppm. **^{13}C{^{1}H} NMR (101 MHz, 298 K, THF-d$_8$):** δ = 177.86 (d, $^1J_{CP}$ = 81 Hz, (TMS-TsN*C*O)$_3$P$_7$), 145.47 (s, *Ar*), 140.39 (s, *Ar*), 131.22 (s, *Ar*), 128.86 (s, *Ar*), 22.46 (s, *Me*), 0.90 (s, Si–*Me*) ppm. **^{29}Si DEPT90 NMR (79 MHz, 298 K, THF-d$_8$):** δ = 31.83 (s, Me$_3$*Si*–O) ppm. **^{31}P NMR (162 MHz, 298 K, THF-d$_8$):** δ = 103.95 - 93.65 (m, 3P, *bridging A*), –119.20 - –124.56 (q, $^1J_{PP}$ = 327 Hz, 1P, *apical A*), –167.22 - –171.61 (m, 3P, *basal A*) ppm.

Elemental analysis for $C_{33}H_{48}N_3O_9P_7S_3Si_3$: calcd.: C 38.56, H 4.71, N 4.09, S 9.36; found: C 38.68, H 4.76, N 4.09, S 9.12.

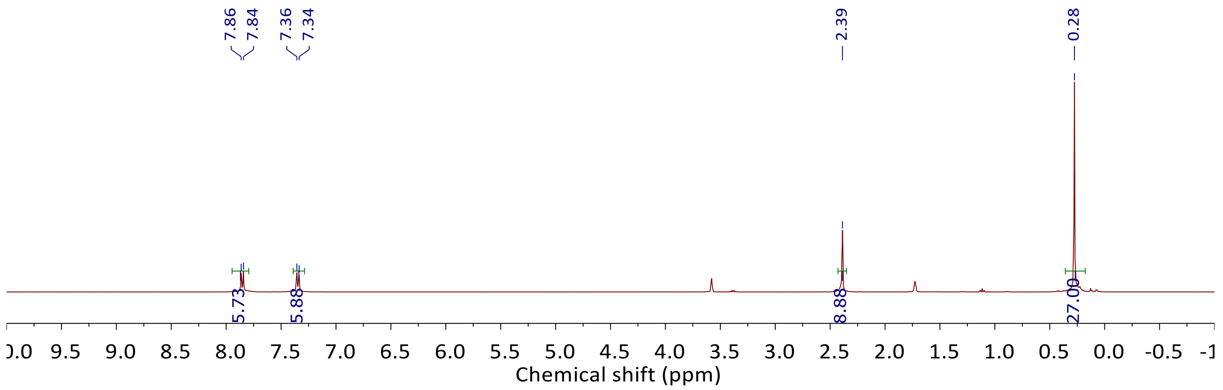

Figure S54. ^{1}H NMR spectrum (THF-d$_8$) of **9**.

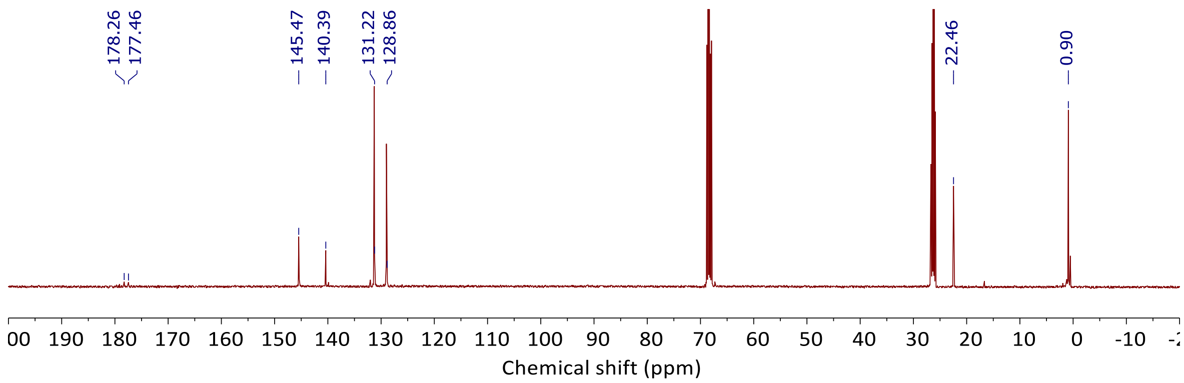

Figure S55. ^{13}C{^{1}H} NMR spectrum (THF-d$_8$) of **9**.

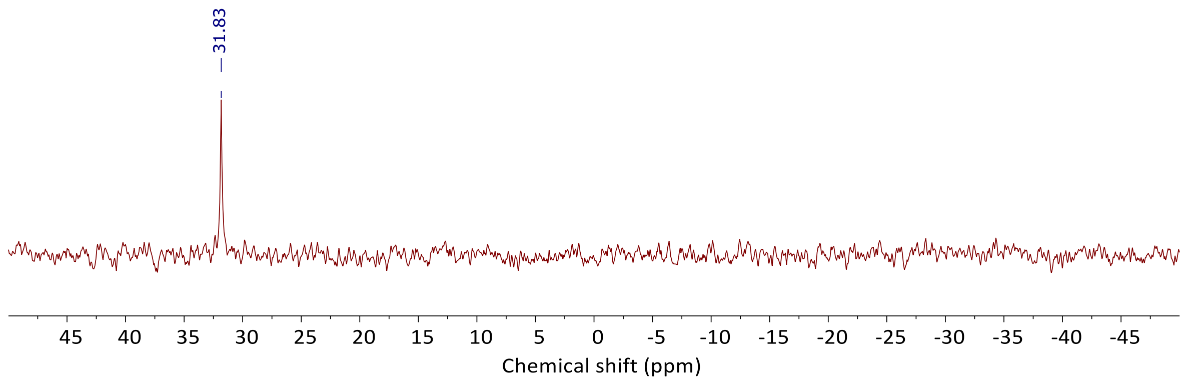

Figure S56. ^{29}Si DEPT90 NMR spectrum (THF-d_8) of **9**.

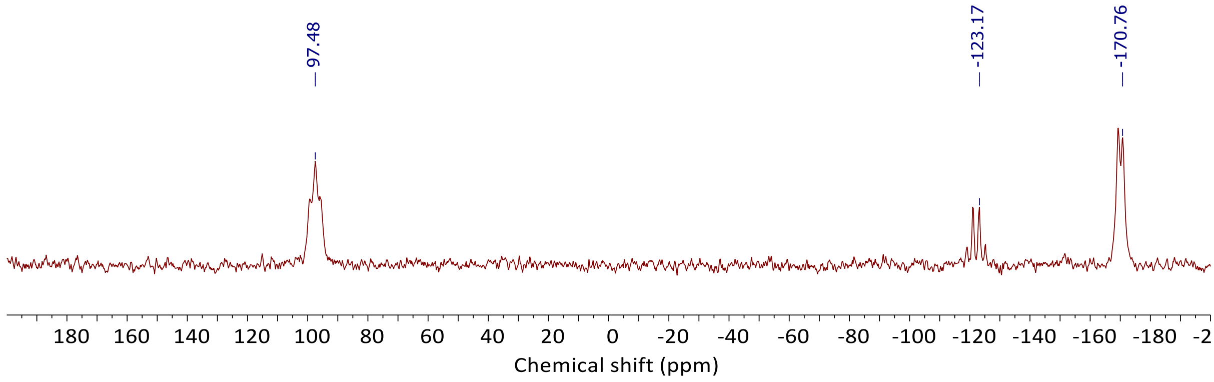

Figure S57. ^{31}P NMR spectrum (THF-d_8) of **9**.

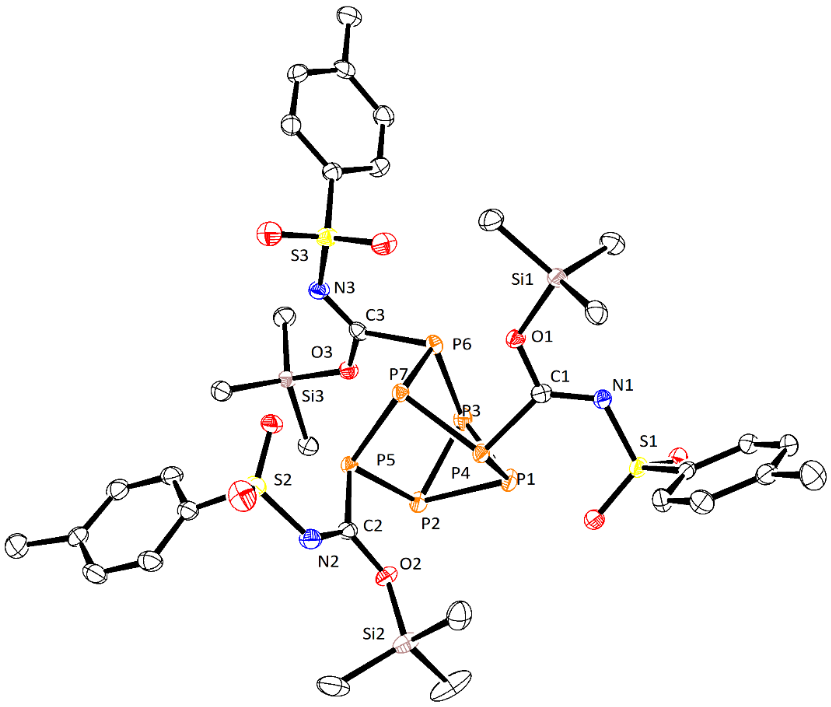

Figure S58. Molecular structure of **9**. Anisotropic displacement ellipsoids pictured at 50% probability. Hydrogen atoms omitted for clarity. Phosphorus: orange; Nitrogen: blue; Oxygen: red; Silicon: pink; Sulfur: yellow; Carbon: white.

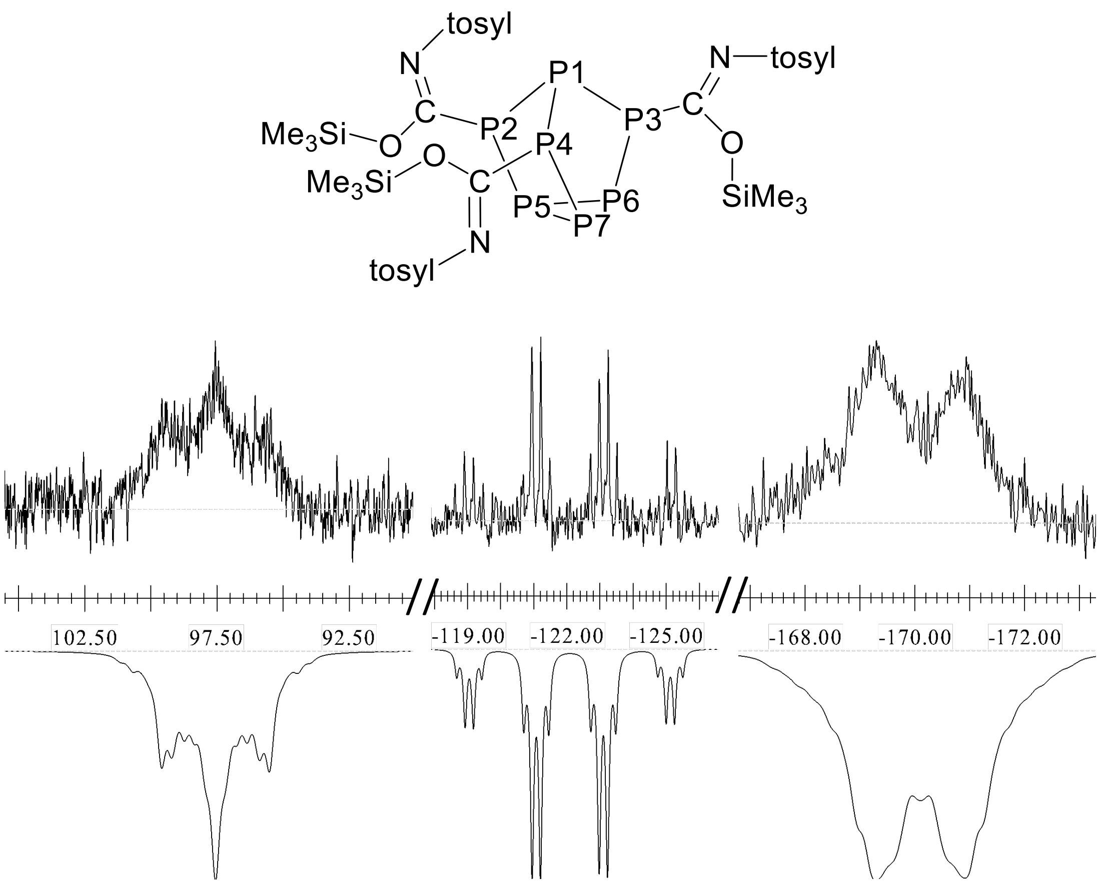

Figure S59. ^{31}P NMR spectra of **9**. Top: experimental, bottom: simulated.

Table S1. Simulated NMR spectroscopic values of **9**.

Nucleus	Chemical shift (ppm)
P1	–122.08
P2, P3, P4	97.54
P5, P6, P7	–170.10
***J* coupling**	**Coupling constant (Hz)**
$^{1}J_{P1–P2}$	324.86
$^{1}J_{P1–P3}$	324.86
$^{1}J_{P1–P4}$	324.86
$^{2}J_{P1–P5}$	44.03
$^{2}J_{P1–P6}$	44.03
$^{2}J_{P1–P7}$	44.03
$^{1}J_{P2–P5}$	331.25
$^{1}J_{P3–P6}$	331.25
$^{1}J_{P4–P7}$	331.25
$^{1}J_{P5–P6}$	213.32
$^{1}J_{P5–P7}$	213.32
$^{1}J_{P6–P7}$	213.32
Final residual	1.26e+05

2.2.10. Synthesis $(Me_2PhSi\text{-}PhNCO)_3P_7$ (13)

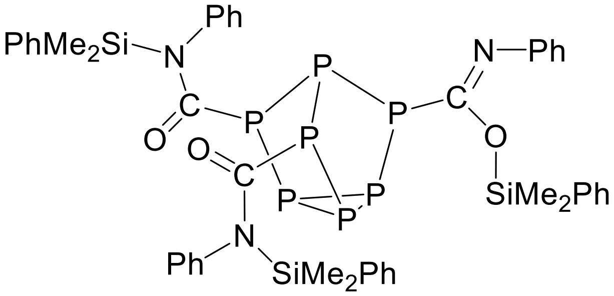

To a J young ampoule charged with a stir bar and $(Me_2PhSi)_3P_7$ (**10**) (72 mg, 0.12 mmol, 1.0 eq.) THF (0.4 mL) was added. To a separate vial, PhNCO (41 mg, 0.36 mmol, 3.0 eq.) and THF (0.4 mL) was added. The solution of PhNCO was added to the $(Me_2PhSi)_3P_7$ solution and allowed to react for 2 days at room temperature. The volatiles were removed yielding a glassy solid. The glassy solid was dissolved in Et_2O (8 mL). Slow evaporation of the solvent yielded a fine powder that was washed with Et_2O (5 mL) and solvent removed.

Isolated Yield: 105 mg, 89%.

^{1}H NMR (400 MHz, 298 K, THF-d_8): δ = 7.71 - 6.32 (m, 30H, *Ph*), 0.56 - 0.22 (overlapping singlets, 18H, *Me*) ppm. **^{13}C{^{1}H} NMR (101 MHz, 298 K, THF-d_8):** δ = 135.66 (s, *Ph*) 133.87 (s, *Ph*), 133.56 (s, *Ph*), 129.50 (s, *Ph*), 129.21 (s, *Ph*),128.92 (s, *Ph*), 128.72 (s, *Ph*), 127.44 (s, *Ph*), 127.26 (s, *Ph*), 121.91 (s, *Ph*), 121.69 (s, *Ph*), –1.75 - –2.02 (m, *Me*), –2.34 (s, *Me*) ppm. **^{29}Si DEPT90 NMR (79 MHz, 298 K, THF-d_8):** δ = 12.98 (s, Me_3*Si*–O), 12.83 (s, Me_3*Si*–O), 12.69 (s, Me_3*Si*–O), 3.59 (s, Me_3*Si*–N), 3.51 (s, Me_3*Si*–N), 3.42 (s, Me_3*Si*–N) ppm. **^{31}P NMR (162 MHz, 298 K, THF-d_8):** δ = 144.29 - 129.65 (m, 6P, *bridging A*), 127.33 - 114.43 (m, 2P, *bridging B*), 107.33 - 93.12 (m, 2P, *bridging B*), 91.24 - 77.03 (m, 2P, *bridging B*), –119.40 - –135.78 (m, 4P, *apical A and B*), –164.77 - –190.14 (m, 12P, *basal A and B*) ppm.

Mass spectrometry (ESI neg/pos): $C_{45}H_{48}N_3O_3P_7Si_3$+Na ($[M+Na]^+$): calcd.: 1002.1064; found: 1002.1043.

Elemental analysis for $C_{45}H_{48}N_3O_3P_7Si_3$: calcd.: C 55.15, H 4.94, N 4.29; found: C 55.13, H 5.20, N 4.11.

Note: A doublet resonance associated with the carbonyl carbon bounded to the P_7 cluster in the ^{13}C{^{1}H} NMR spectrum is not observed, possibly due to insufficient

solubility of in THF. N,N,O-coordination of the silyl units is assigned based on the ^{1}H and ^{31}Si NMR data.

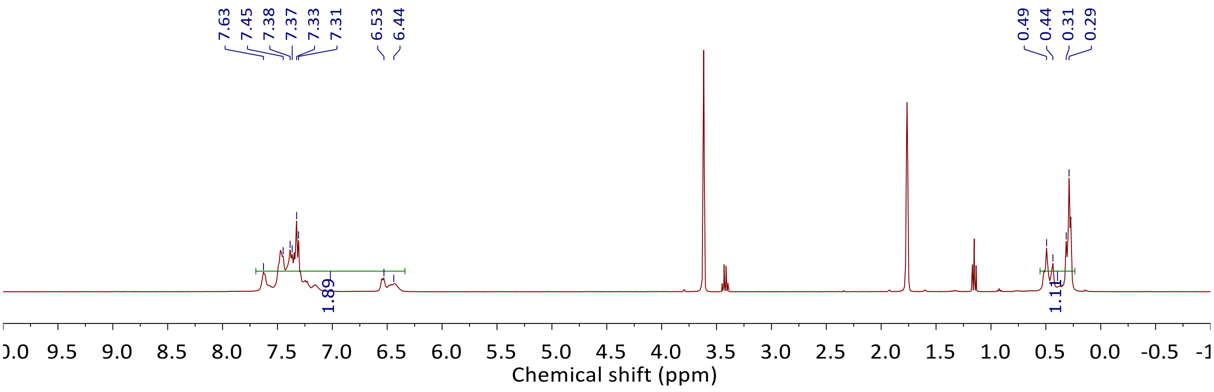

Figure S60. ^{1}H NMR spectrum (THF-d_8) of **13**.

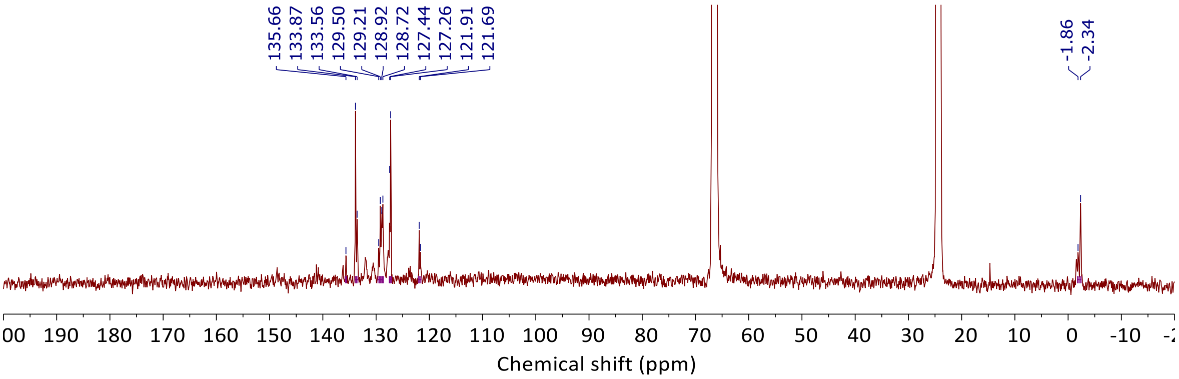

Figure S61. ^{13}C{^{1}H} NMR spectrum (THF-d_8) of **13**.

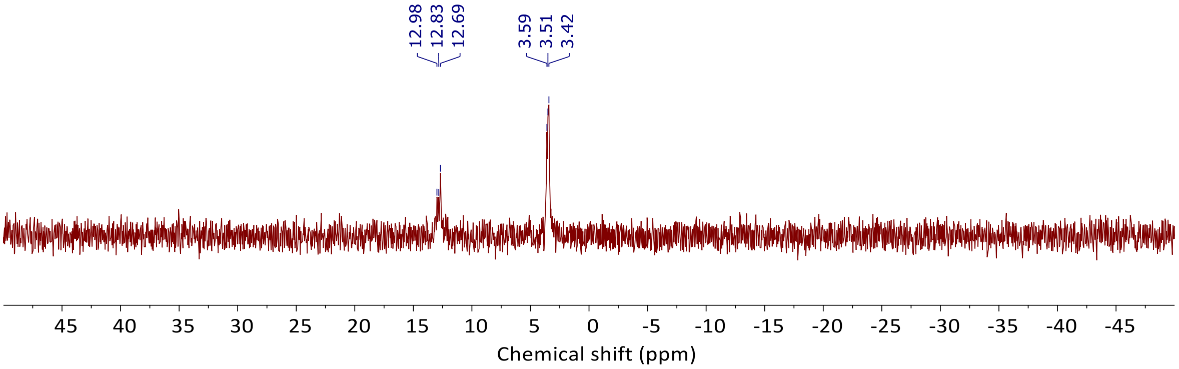

Figure S62. ^{29}Si DEPT90 NMR spectrum (THF-d_8) of **13**.

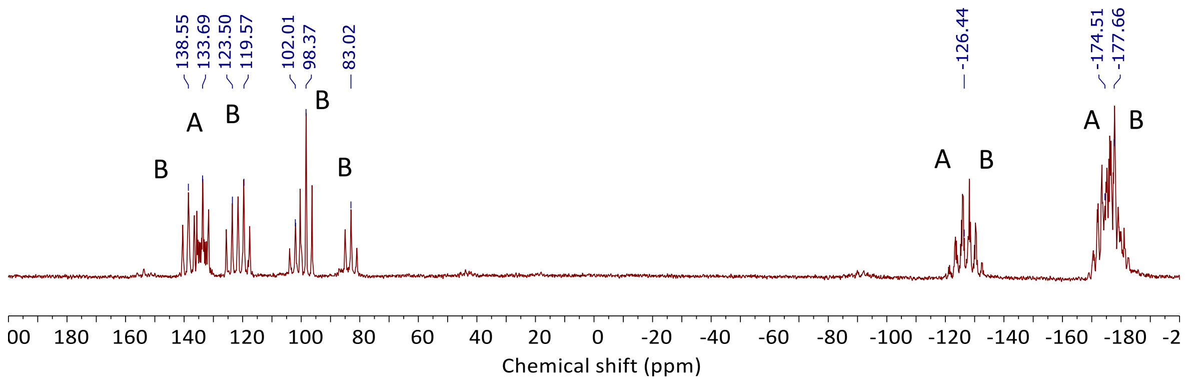

Figure S63. ^{31}P NMR spectrum (THF-d_8) of **13**.

2.2.11. Synthesis $(MePh_2Si\text{-}PhNCO)_3P_7$ (14)

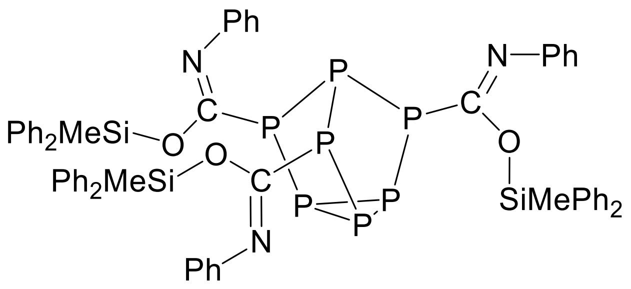

To a J young ampoule charged with a stir bar and $(MePh_2Si)_3P_7$ (**11**) (93 mg, 0.12 mmol, 1.0 eq.) THF (0.4 mL) was added. To a separate vial, PhNCO (42 mg, 0.36 mmol, 3.0 eq.) and THF (0.4 mL) was added. The solution of PhNCO was added to the $(MePh_2Si)_3P_7$ solution and allowed to react for 3 days at room temperature. The volatiles were removed yielding an oily solid.

Isolated Yield: 99 mg, 71%.

^{1}H NMR (400 MHz, 298 K, THF-d_8): δ = 7.90 - 6.07 (m, 45H, *Ar*), 0.78 - 0.24 (overlapping singlets, 9H, *Me*) ppm. **^{13}C{^{1}H} NMR (101 MHz, 298 K, THF-d_8):** δ = 181.27 (d, $^{1}J_{CP}$ = 41 Hz), 135.97 (s, *Ar*), 135.62 (s, *Ar*), 134.83 (s, *Ar*), 131.29 (s, *Ar*), 130.66 (s, *Ar*), 130.43 (s, *Ar*), 130.32 (s, *Ar*), 129.89 (s, *Ar*), 129.75 (s, *Ar*), 129.66 (s, *Ar*), 128.86 (s, *Ar*), 128.44 (s, *Ar*), 128.29 (s, *Ar*), 124.78 (s, *Ar*), 122.71 (s, *Ar*), 122.50 (s, *Ar*), –2.01 (s, *Me*) ppm. **^{29}Si DEPT90 NMR (79 MHz, 298 K, THF-d_8):** δ = 9.98 (s, Me_3*Si*–O), 8.39 (s, Me_3*Si*–O) ppm. **^{31}P NMR (162 MHz, 298 K, THF-d_8):** δ = 142.58 - 127.13 (m, 1P, *bridging B*), 127.58 - 114.09 (m, 1P, *bridging B*), 110.91 - 92.73 (m,

3P, *bridging A*), 92.73 - 75.91 (m, 1P, *bridging B*), –112.60 - –138.67 (m, 2P, *apical A and B*), –149.42 - –199.13 (m, 6P, *basal A and B*) ppm.

Mass spectrometry (ESI neg/pos): $C_{60}H_{54}N_3O_3P_7Si_3$+Na ([M+Na]$^+$): calcd.: 1188.1528; found: 1188.1505.

Elemental analysis for $C_{60}H_{54}N_3O_3P_7Si_3$: calcd.: C 61.80, H 4.67, N 3.60; found: C 60.81, H 4.29, N 3.01

Note: O,O,O-coordination of the silyl units is assigned based on the ^{1}H and ^{29}Si NMR data. Best elemental analysis is presented.

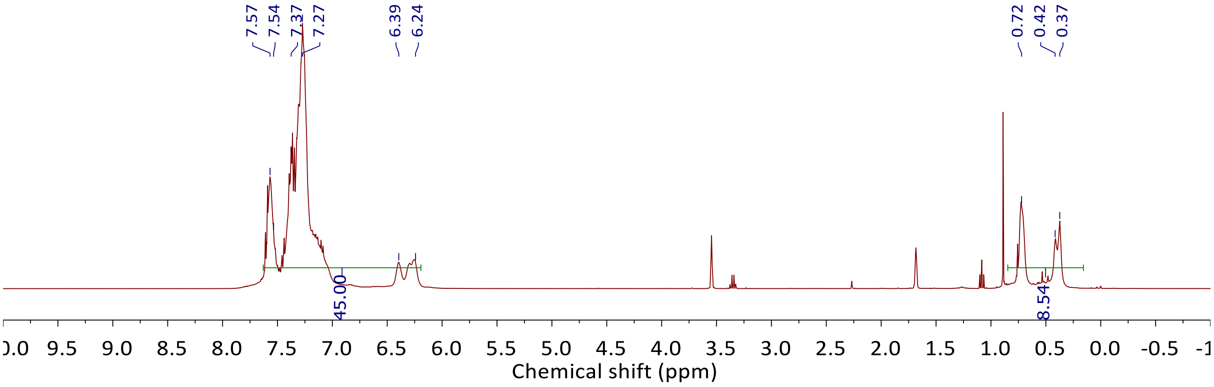

Figure S64. ^{1}H NMR spectrum (THF-d_8) of **14**.

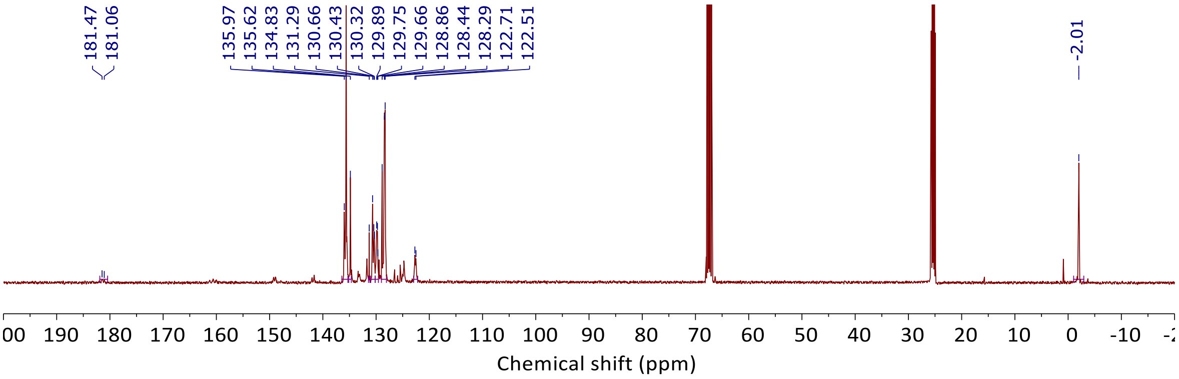

Figure S65. ^{13}C{^{1}H} NMR spectrum (THF-d_8) of **14**.

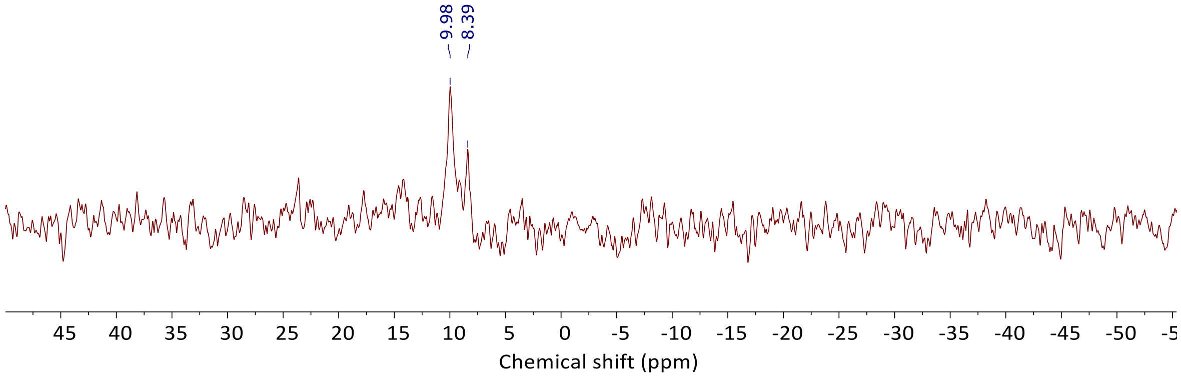

Figure S66. ^{29}Si DEPT90 NMR spectrum (THF-d_8) of **14**.

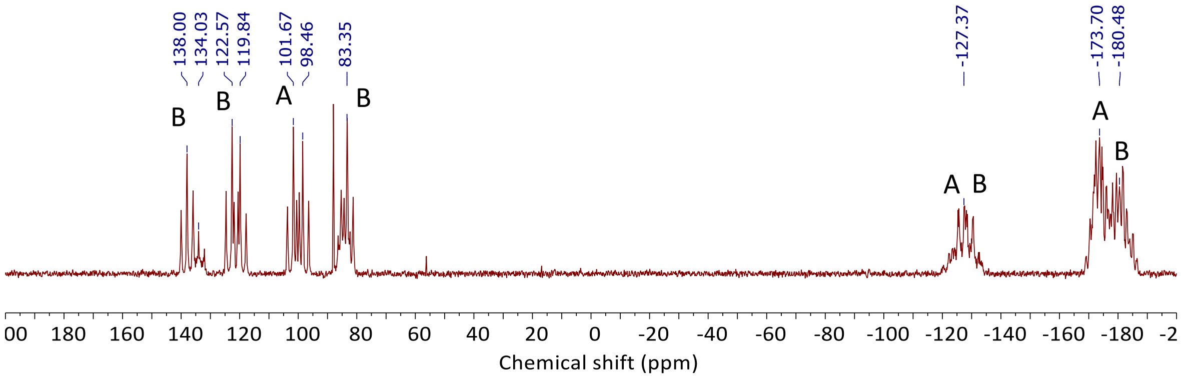

Figure S67. ^{31}P NMR spectrum (THF-d_8) of **14**.

2.2.12. Synthesis $(Ph_3Si\text{-}PhNCO)_3P_7$ (15)

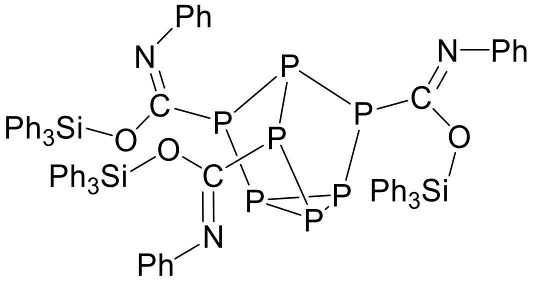

To a J young ampoule charged with a stir bar and $(Ph_3Si)_3P_7$ (**12**) (114 mg, 0.12 mmol, 1.0 eq.) THF (0.4 mL) was added. To a separate vial, PhNCO (42 mg, 0.36 mmol, 3.0 eq.) and THF (0.4 mL) was added. The solution of PhNCO was added to the $(Ph_3Si)_3P_7$ solution and allowed to react for 21 days at 50 °C. The volatiles were removed yielding a glassy solid. The glassy solid was dissolved in Et_2O (2 mL). Cooling down to –30 °C yielded block shaped crystals. The crystals were washed with Et_2O (5 mL) and solvent removed, yielding a pale crystalline solid. Crystals suitable for single crystals X-ray

diffraction analysis were obtained through slow evaporation of a concentrated Et_2O solution.

Isolated Yield: 81 mg, 50%.

^1H NMR (400 MHz, 298 K, THF-d_8): δ = 7.49 - 7.43 (m, *Ar*) ppm. **$^{13}C\{^1H\}$ NMR (101 MHz, 298 K, THF-d_8):** δ = 147.63 (s, *Ar*), 135.95 (s, *Ar*), 132.93 (s, *Ar*), 129.97 (s , *Ar*), 129.00 (s, *Ar*), 127.54 (s, *Ar*), 124.11 (s, *Ar*), 121.33 (s, *Ar*) ppm. **^{29}Si DEPT90 NMR (79 MHz, 298 K, THF-d_8):** δ = 7.53 (s, Me_3*Si*–O) ppm. **^{31}P NMR (162 MHz, 298 K, THF-d_8):** δ = 126.85 - 114.59 (m, 1P, *bridging B*), 107.01 - 94.53 (m, 1P, *bridging B*), 90.52 - 72.02 (m, 4P, *bridging A and B*), –119.70 - –136.86 (m, 2P, *apical A and B*), –161.38 - –188.13 (m, 6P, *basal A and B*) ppm.

Mass spectrometry (ESI neg/pos): $C_{75}H_{60}N_3O_3P_7Si_3$+Na ([M+Na]$^+$): calcd.: 1374.1988; found: 1374.1979.

Elemental analysis for $C_{75}H_{60}N_3O_3P_7Si_3$: calc.: C 66.61, H 4.47, N 3.11; found: C 66.64, H 4.24, N 2.97.

Note: A doublet resonance associated with the carbonyl carbon bounded to the P_7 cluster in the $^{13}C\{^1H\}$ NMR spectrum is not observed, possibly due to insufficient solubility of in THF.

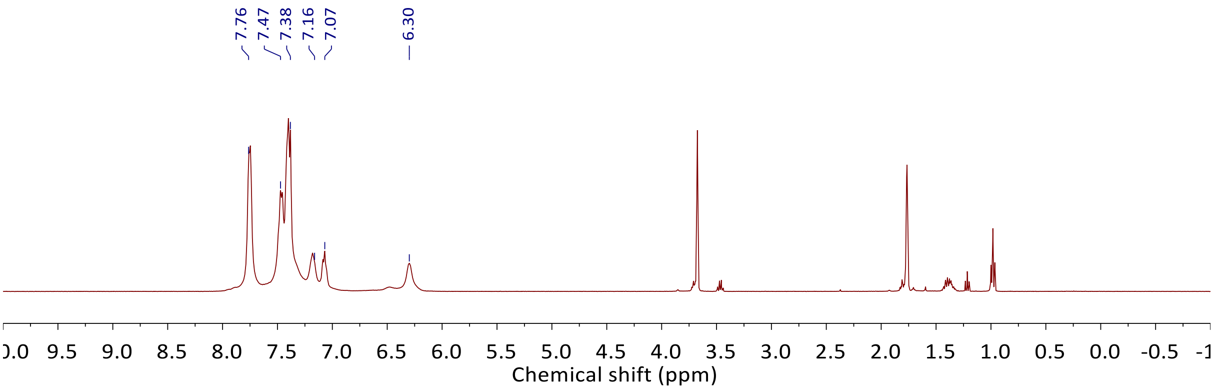

Figure S68. ^1H NMR spectrum (THF-d_8) of **15**.

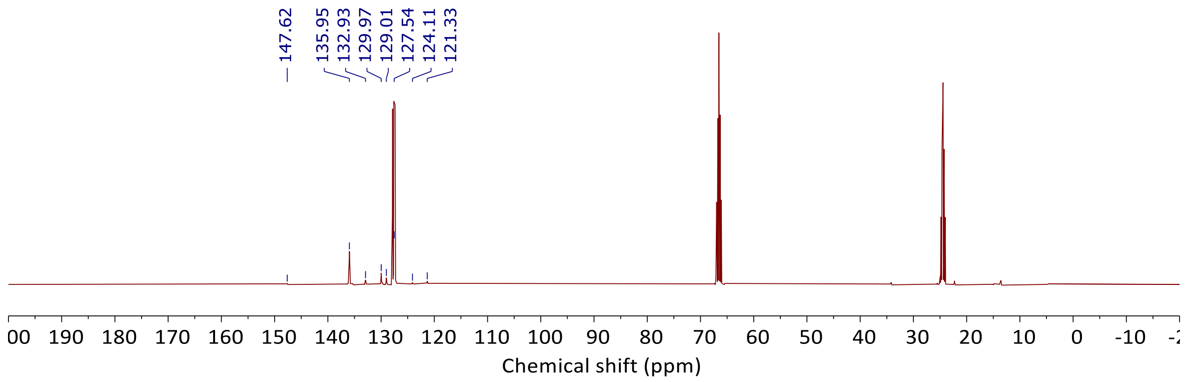

Figure S69. $^{13}C\{^{1}H\}$ NMR spectrum (THF-d_8) of **15**.

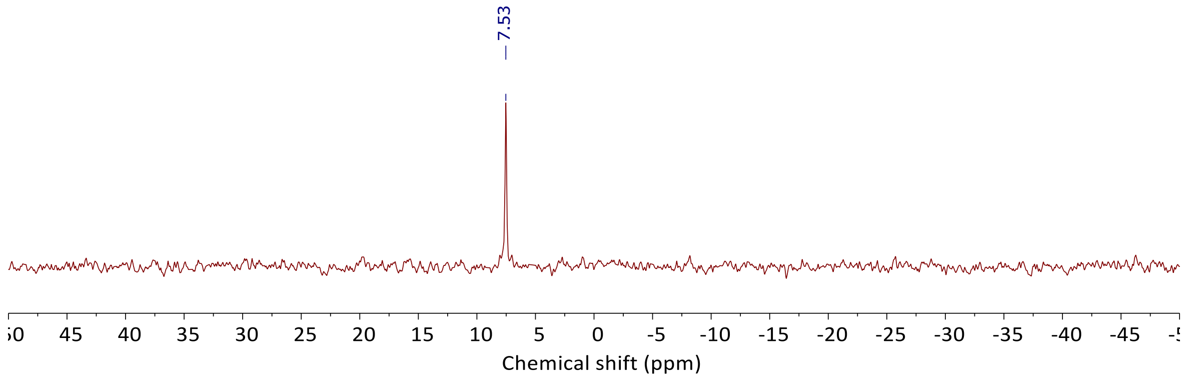

Figure S70. ^{29}Si DEPT90 NMR spectrum (THF-d_8) of **15**.

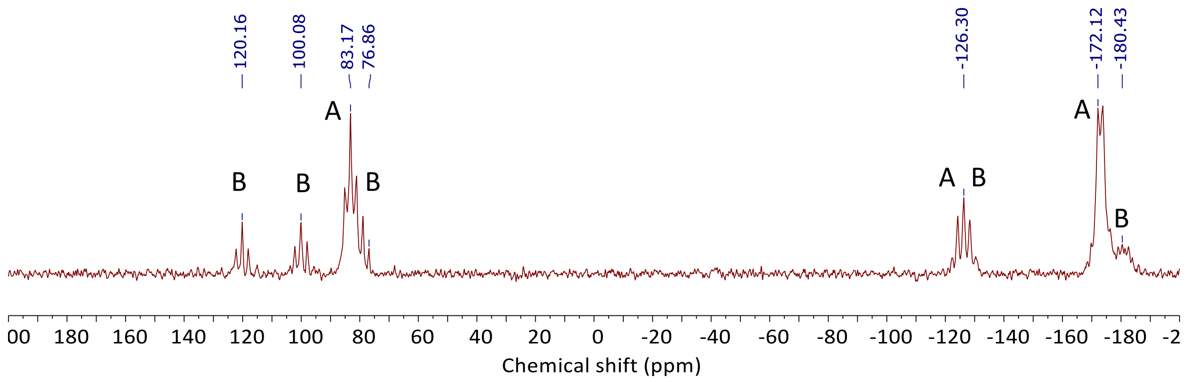

Figure S71. ^{31}P NMR spectrum (THF-d_8) of **15**.

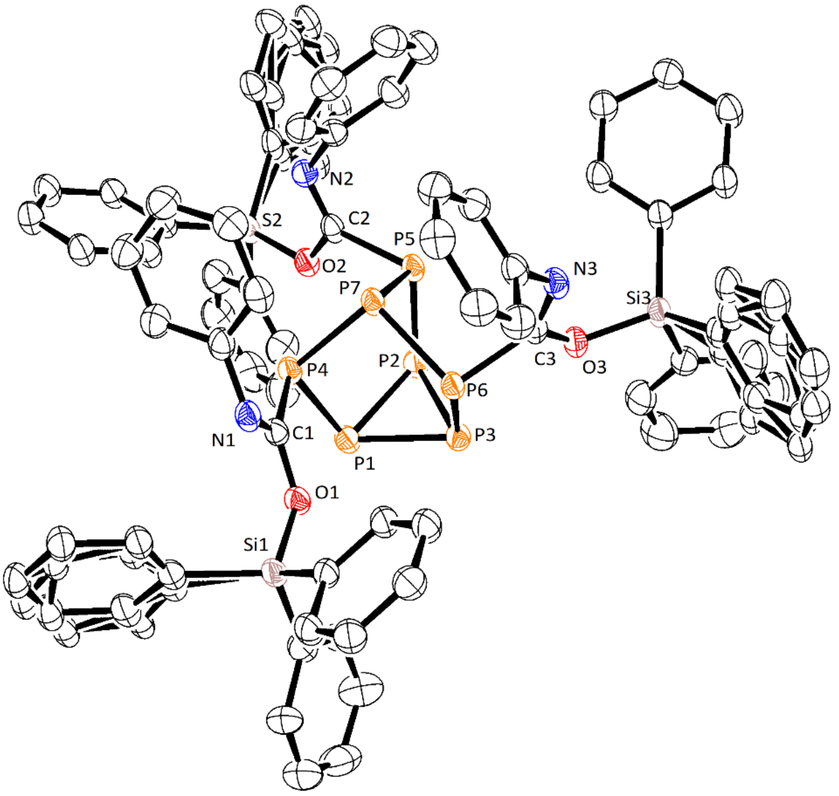

Figure S72. Molecular structure of **15**. Anisotropic displacement ellipsoids pictured at 50% probability. Hydrogen atoms and Et_2O solvent molecule omitted for clarity. Phosphorus: orange; Nitrogen: blue; Oxygen: red; Silicon: pink; Carbon: white.

2.2.13. Synthesis $(Me_2PhSi\text{-}TosylNCO)_3P_7$ (16)

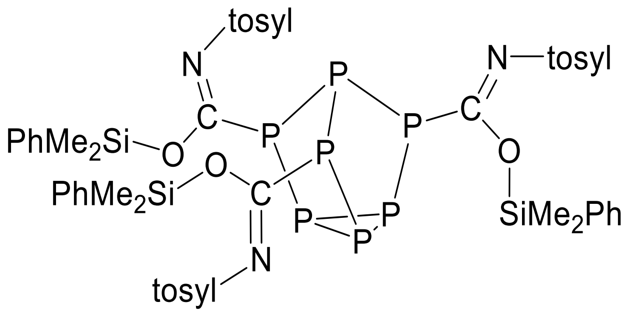

To a J young ampoule charged with a stir bar and $(Me_2PhSi)_3P_7$ (**10**) (50 mg, 0.08 mmol, 1.0 eq.) THF (0.4 mL) was added. To a separate vial, tosylNCO (48 mg, 0.24 mmol, 3.0 eq.) and THF (0.4 mL) was added. The solution of tosylNCO was added to

the $(Me_2PhSi)_3P_7$ solution and allowed to react overnight at room temperature. The volatiles were removed yielding a glassy solid. The glassy solids were washed with pentane (2 mL), and dried under reduced pressure.

Isolated yield: 39 mg, 40%.

^{1}H NMR (400 MHz, 298 K, THF-d$_8$): δ = 7.72 - 7.52 (m, 6H, *Ar*), 7.50 - 7.40 (m, 6H, *Ar*), 7.24 - 7.11 (m, 15H, Si–*Ph*), 2.27 (s, 9H, *Me*), 0.42 (s, 18H, Si–*Me*) ppm. **^{13}C{^{1}H} NMR (101 MHz, 298 K, THF-d$_8$):** δ = 176.81 (d, $^1J_{CP}$ = 83 Hz, $(Me_2PhSi\text{-}TsN\mathit{C}O)_3P_7$), 144.37 (s, *Ar*), 139.40 (s, *Ar*), 135.30 (s, *Ar*), 134.93 (s, *Ar*), 133.92 (s, *Ar*), 131.12 (s, *Ar*), 130.45 (s, *Ar*), 128.75 (s, *Ar*), 128.20 (s, *Ar*), 21.64 (s, *Me*), –1.39 (s, Si–*Me*) ppm. **^{29}Si DEPT90 NMR (79 MHz, 298 K, THF-d$_8$):** δ = 19.13 (s, Me_3*Si*–O) ppm. **^{31}P NMR (162 MHz, 298 K, THF-d$_8$:** δ = 107.81 - 83.26 (m, 3P, *bridging A*), –113.17 - –133.09 (qq, $^1J_{PP}$ = 330 Hz, $^2J_{PP}$ = 44 Hz, 1P, apical A), –161.35 - –175.71 (m, 3P, *basal A*) ppm.

Elemental analysis for $C_{48}H_{54}N_3O_9P_7S_3Si_3$: calcd.: C 47.48, H 4.48, N 3.46, S 7.92; found: C 47.11, H 4.61, N 3.48, S 7.92.

Note: O,O,O-coordination of the silyl units is assigned based on the ^{1}H and ^{29}Si NMR data.

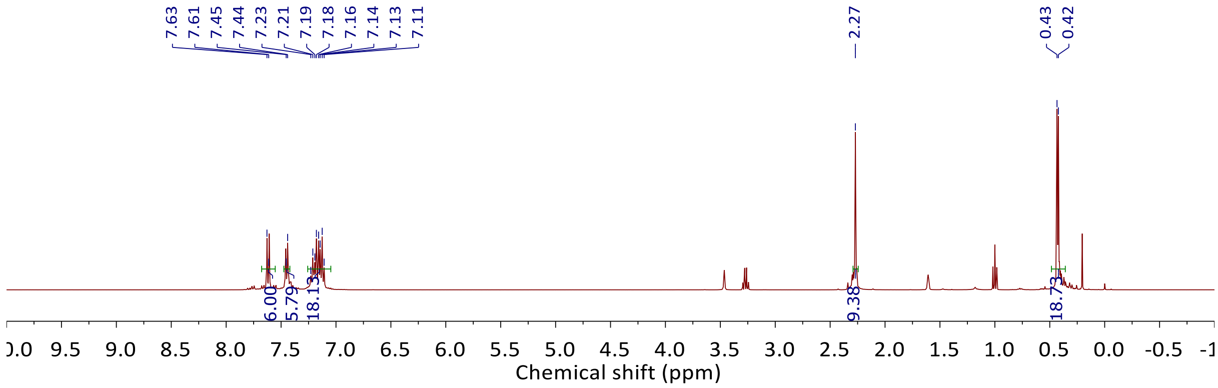

Figure S73. ^{1}H NMR spectrum (THF-d$_8$) of **16**.

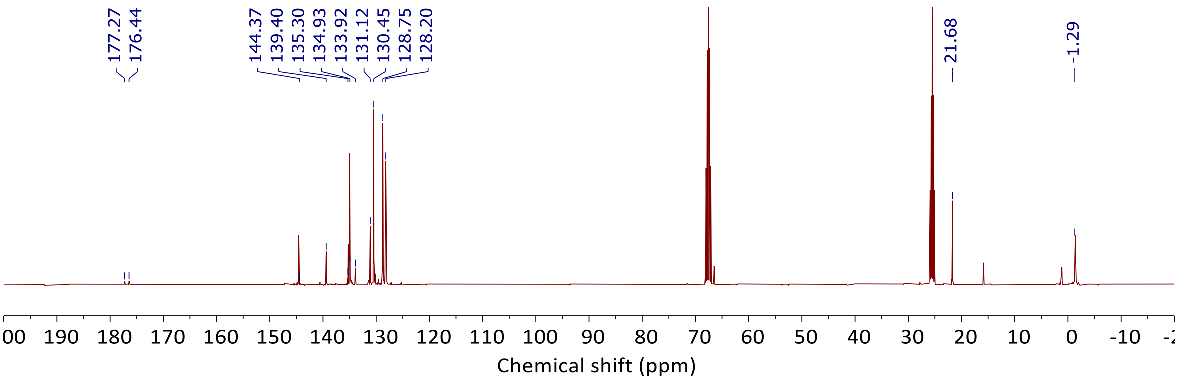

Figure S74. $^{13}C\{^1H\}$ NMR spectrum (THF-d_8) of **16**.

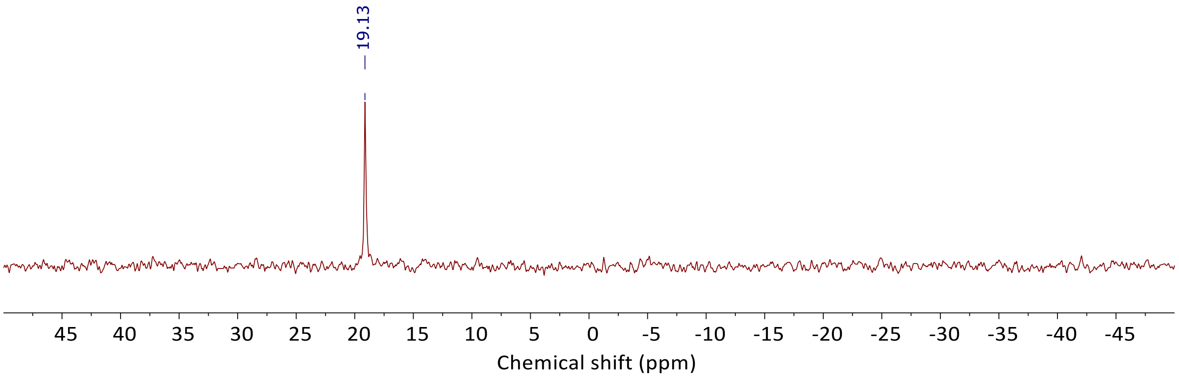

Figure S75. ^{29}Si DEPT90 NMR spectrum (THF-d_8) of **16**.

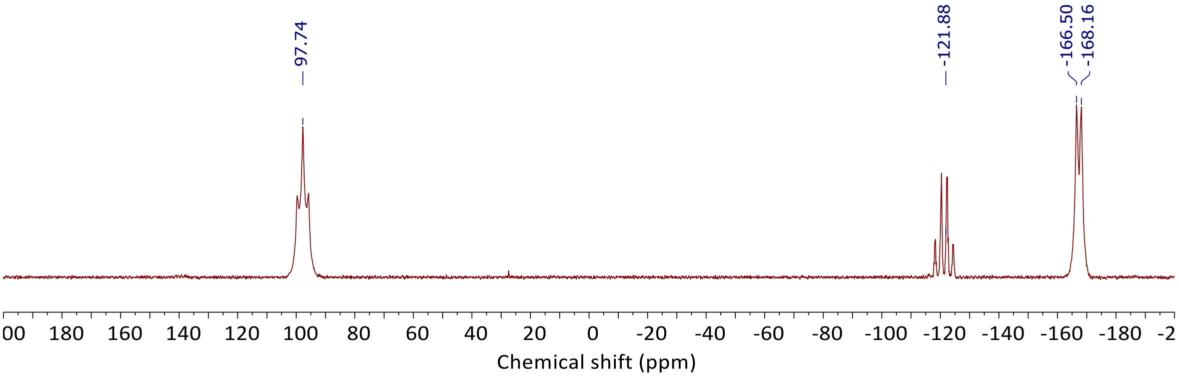

Figure S76. ^{31}P NMR spectrum (THF-d_8) of **16**.

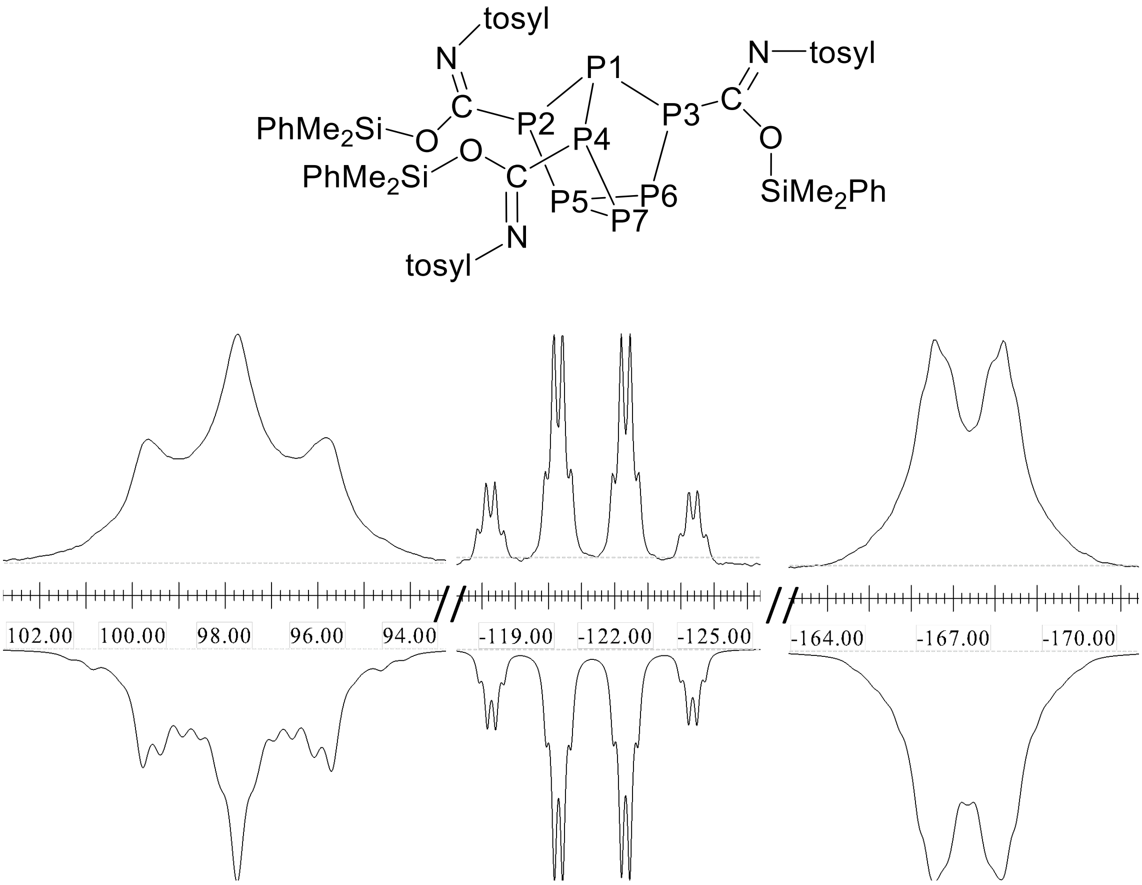

Figure S77. ^{31}P NMR spectra of **16**. Top: experimental, bottom: simulated.

Table S2. Simulated NMR spectroscopic values of **16**.

Nucleus	Chemical shift (ppm)
P1	–121.211
P2, P3, P4	97.628
P5, P6, P7	–167.123
***J* coupling**	**coupling constant (Hz)**
$^{1}J_{P1–P2}$	327.75
$^{1}J_{P1–P3}$	327.75
$^{1}J_{P1–P4}$	327.75
$^{2}J_{P1–P5}$	40.95
$^{2}J_{P1–P6}$	40.95
$^{2}J_{P1–P7}$	40.95
$^{1}J_{P2–P5}$	335.86
$^{1}J_{P3–P6}$	335.86
$^{1}J_{P4–P7}$	335.86
$^{1}J_{P5–P6}$	207.46
$^{1}J_{P5–P7}$	207.46
$^{1}J_{P6–P7}$	207.46
Final residual	1.26e+05

2.2.14. Synthesis $(MePh_2Si\text{-}TosylNCO)_3P_7$ (17)

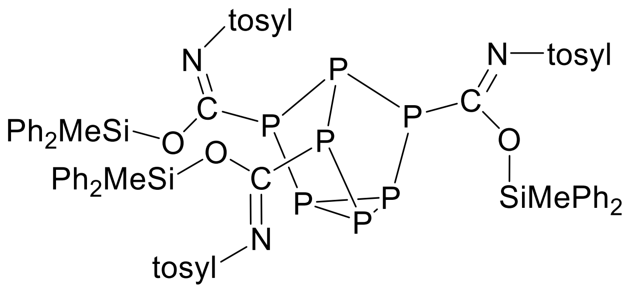

To a J young ampoule charged with a stir bar and $(MePh_2Si)_3P_7$ (**11**) (50 mg, 0.06 mmol, 1.0 eq.) THF (0.4 mL) was added. To a separate vial, tosylNCO (37 mg, 0.18 mmol, 3.0 eq.) and THF (0.4 mL) was added. The solution of tosylNCO was added to the $(Me_2PhSi)_3P_7$ solution and allowed to react overnight at room temperature. The volatiles were removed yielding an oily solid.
Isolated yield: 71 mg, 81%.

^{1}H NMR (400 MHz, 298 K, THF-d$_8$): δ = 7.79 - 7.03 (m, 42H, *Ar*), 2.37 (s, 9H, *Me*), 0.85 (s, 9H, *Si–Me*) ppm. **^{13}C{^{1}H} NMR (101 MHz, 298 K, THF-d$_8$):** δ = 176.20 (d, $^1J_{CP}$ = 85 Hz, *Ar*), 144.17 (s, *Ar*), 138.92 (s, *Ar*), 135.64 (s, *Ar*), 134.83 (s, *Ar*), 133.94 (s, *Ar*), 133.71 (s, *Ar*), 131.05 (s, *Ar*), 130.24 (s, *Ar*), 128.70 (s, *Ar*), 128.07 (s, *Ar*), 21.50 (s, *Me*), –2.52 (s, *Si–Me*) ppm. **^{29}Si DEPT90 NMR (79 MHz, 298 K, THF-d$_8$):** δ = 6.52 (s, Me_3*Si*–O) ppm. **^{31}P NMR (162 MHz, 298 K, THF-d$_8$):** δ = 106.42 - 81.87 (m, 3P, *bridging A*), –113.17 - –128.46 (qq, $^1J_{PP}$ = 331 Hz, $^2J_{PP}$ = 43 Hz, 1P, apical A), –159.96 - –182.66 (m, 3P, *basal A*) ppm.
Elemental analysis for $C_{63}H_{60}N_3O_9P_7S_3Si_3$: calcd.: C 54.03, H 4.32, N 3.00, S 6.87; found: C 52.81, H 4.02, N 2.48, S 6.21.

Note: O,O,O-coordination of the silyl units is assigned based on the ^{1}H and ^{29}Si NMR data. Best elemental analysis is presented.

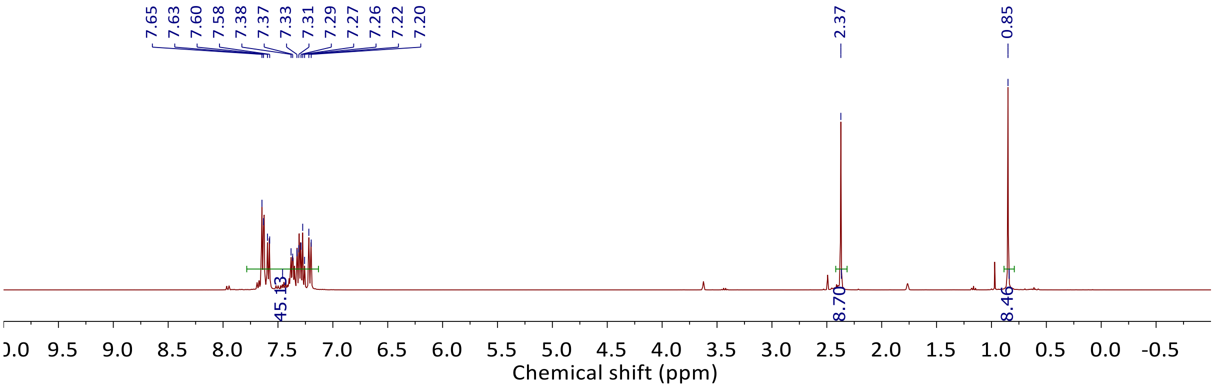

Figure S78. ^{1}H NMR spectrum (THF-d_8) of **17**.

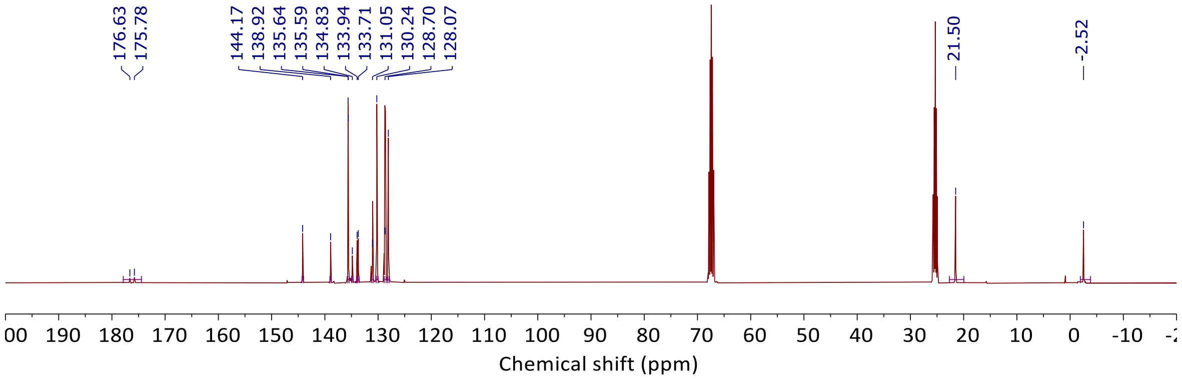

Figure S79. $^{13}C\{^{1}H\}$ NMR spectrum (THF-d_8) of **17**.

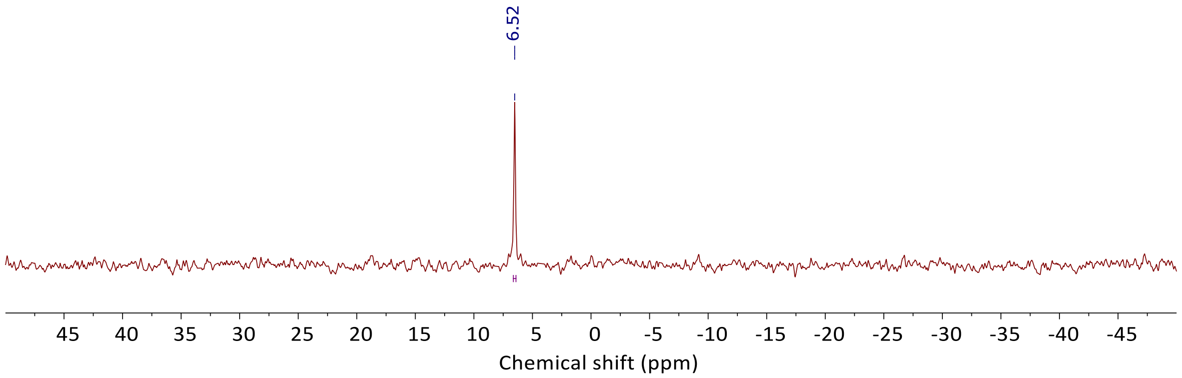

Figure S80. ^{29}Si DEPT90 NMR spectrum (THF-d_8) of **17**.

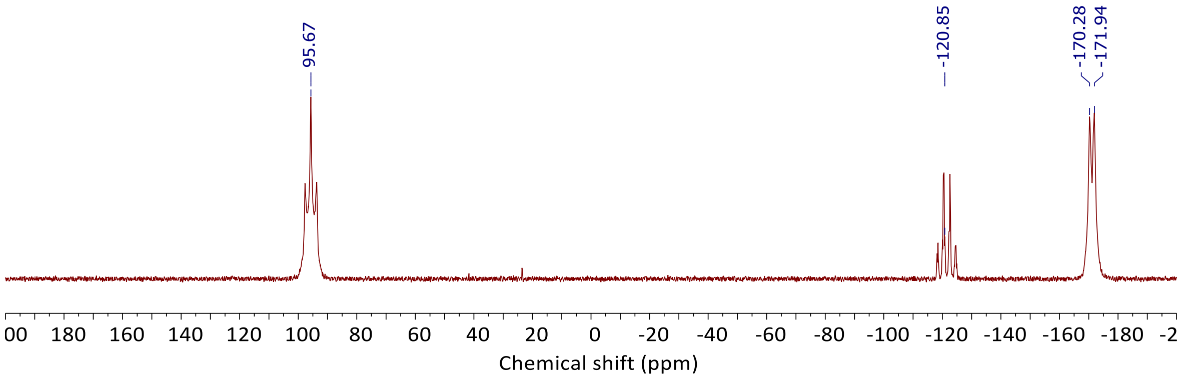

Figure S81. ^{31}P NMR spectrum (THF-d_8) of **17**.

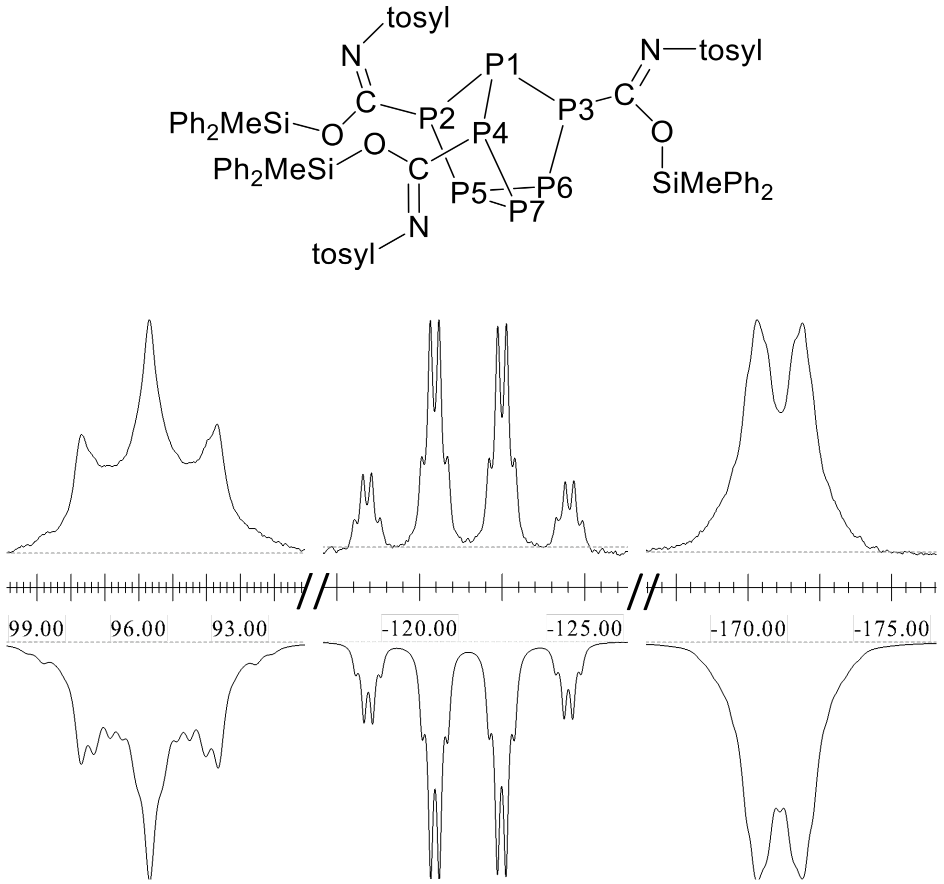

Figure S82. ^{31}P NMR spectra of **17**. Top: experimental, bottom: simulated.

Table S3. Simulated NMR spectroscopic values of **17**.

Nucleus	Chemical shift (ppm)
P1	–121.468
P2, P3, P4	95.666
P5, P6, P7	–171.100
***J* coupling**	**coupling constant (Hz)**
$^{1}J_{P1–P2}$	326.94
$^{1}J_{P1–P3}$	326.94
$^{1}J_{P1–P4}$	326.94
$^{2}J_{P1–P5}$	42.15
$^{2}J_{P1–P6}$	42.15
$^{2}J_{P1–P7}$	42.15
$^{1}J_{P2–P5}$	333.75
$^{1}J_{P3–P6}$	333.75
$^{1}J_{P4–P7}$	333.75
$^{1}J_{P5–P6}$	211.51
$^{1}J_{P5–P7}$	211.51
$^{1}J_{P6–P7}$	211.51
Final residual	5.70e+04

2.2.15. Synthesis $(Ph_3Si\text{-}TosylNCO)_3P_7$ (18)

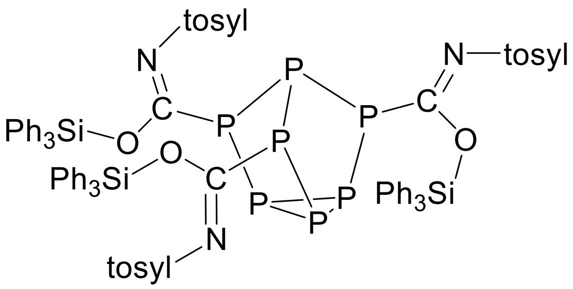

To a J young ampoule charged with a stir bar and $(Ph_3Si)_3P_7$ (**12**) (50 mg, 0.05 mmol, 1.0 eq.) THF (0.4 mL) was added. To a separate vial, tosylNCO (30 mg, 0.15 mmol, 3.0 eq.) and THF (0.4 mL) was added. The solution of tosylNCO was added to the $(Ph_3Si)_3P_7$ solution and allowed to react for 29 days at room temperature. The volatiles were removed yielding a glassy solid. The glassy solid was dissolved in Et_2O (10 mL). Cooling down to –30 °C yielded square shaped crystals. The crystals were washed with Et_2O (5 mL) and dried under reduced pressure, yielding a white crystalline solid. Crystals suitable for single crystals X-ray diffraction analysis were obtained through cooling down a concentrated Et_2O solution.

Isolated Yield: 32 mg, 40%.

^{1}H NMR (400 MHz, 298 K, THF-d_8): δ = 7.49 - 7.43 (m, 18H, *Ph*), 7.41 (d, $^{3}J_{HH}$ = 8.3 Hz, 6H, *Ar*), 7.25 - 7.19 (m, 9H, *Ph*), 7.17 - 7.11 (m, 18H, *Ph*), 6.94 (d, $^{3}J_{HH}$ = 8.0 Hz, 6H, *Ar*), 2.14 (s, 9H, *Me*) ppm. **^{13}C{^{1}H} NMR (101 MHz, 298 K, THF-d_8):** δ = 173.70 (d, $^{1}J_{CP}$ = 87 Hz, $(Ph_3Si\text{-}TsNCO)_3P_7$), 142.97 (s, *Ar*), 137.70 (s, *Ar*), 135.98 (s, *Ar*), 131.23 (s, *Ar*), 130.22 (s, *Ar*), 129.33 (s, *Ar*), 127.84 (s, *Ar*), 127.29 (s, *Ar*), 20.44 (s, *Me*) ppm. **^{29}Si DEPT90 NMR (79 MHz, 298 K, THF-d_8):** δ = –4.58 (s, Me_3*Si*–O) ppm. **^{31}P NMR (162 MHz, 298 K, THF-d_8):** δ = 101.07 - 90.95 (m, 3P, *bridging A*), –117.19 - –126.69 (q, $^{1}J_{PP}$ = 327 Hz), –173.32 - –181.36 (m, 3P, *basal A*) ppm.

Elemental analysis for $C_{78}H_{66}N_3O_9P_7S_3Si_3$: calcd.: C 59.05, H 4.19, N 2.65, S 6.06; found: C 58.81, H 4.27, N 2.60, S 5.75.

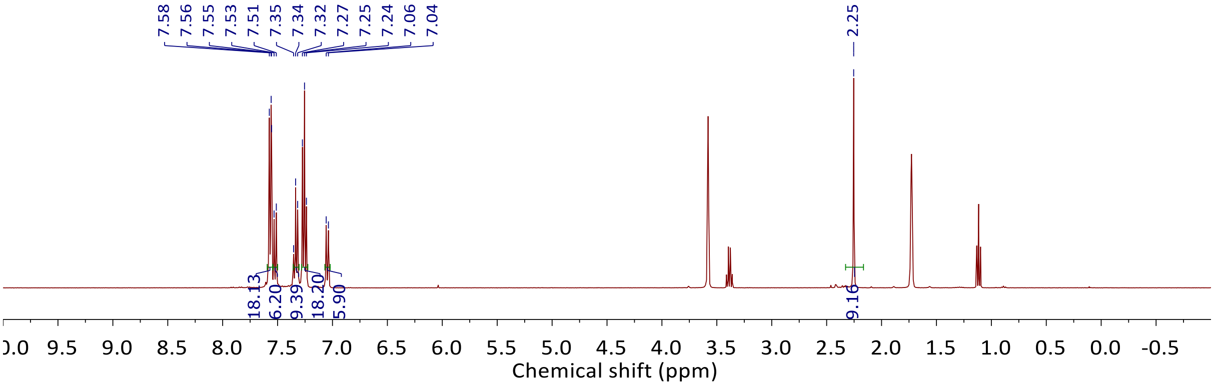

Figure S83. ^{1}H NMR spectrum (THF-d$_8$) of **18**.

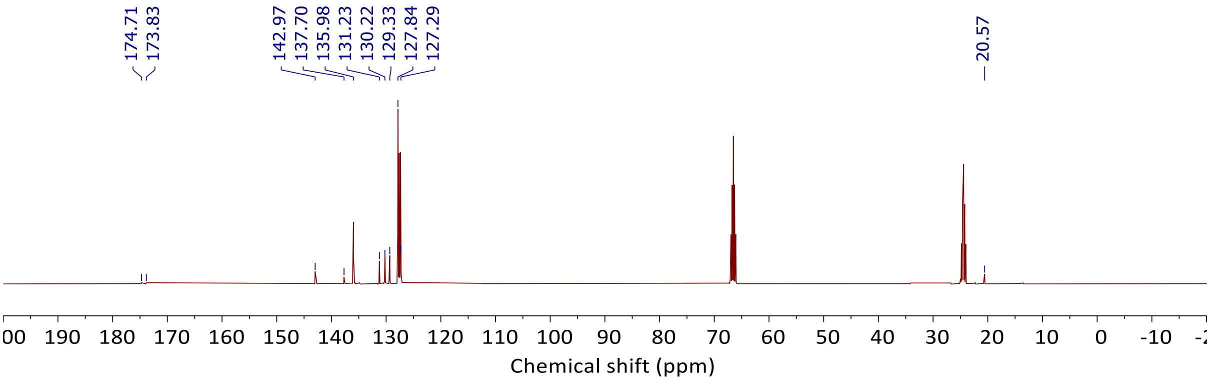

Figure S84. ^{13}C{^{1}H} NMR spectrum (THF-d$_8$) of **18**.

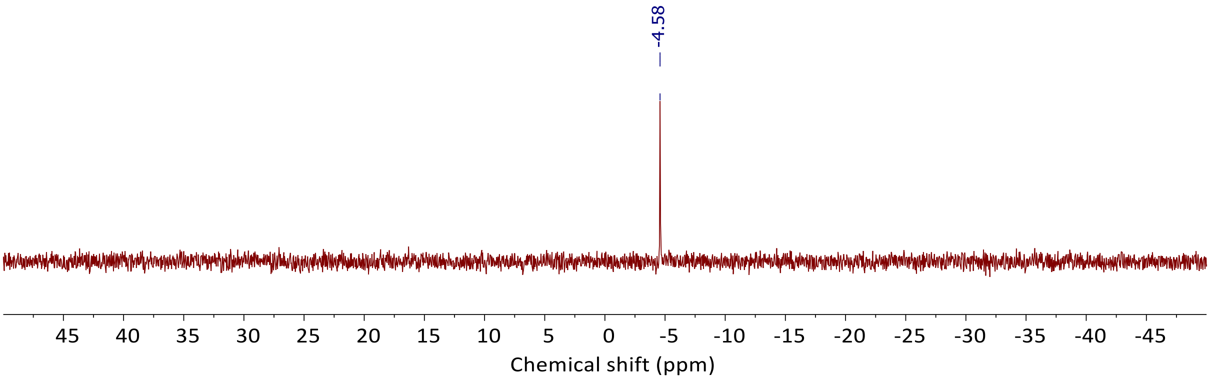

Figure S85. ^{29}Si DEPT90 NMR spectrum (THF-d$_8$) of **18**.

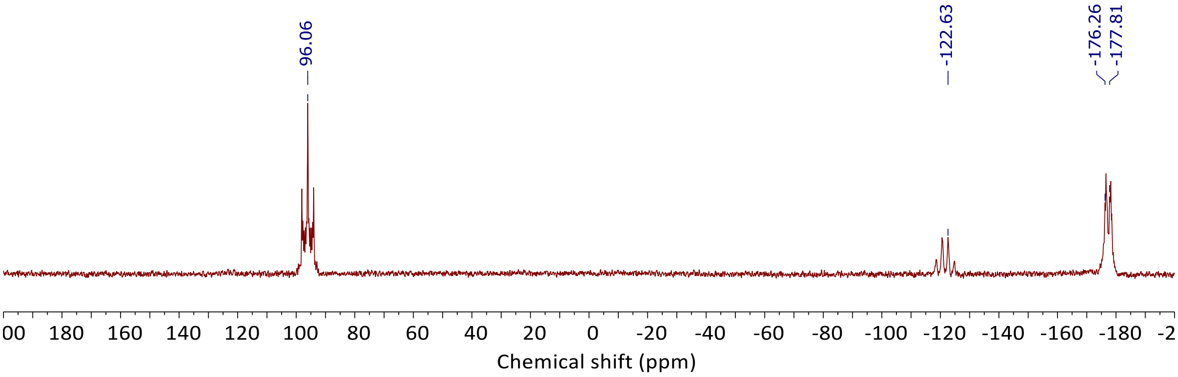

Figure S86. ^{31}P NMR spectrum (THF-d$_8$) of **18**.

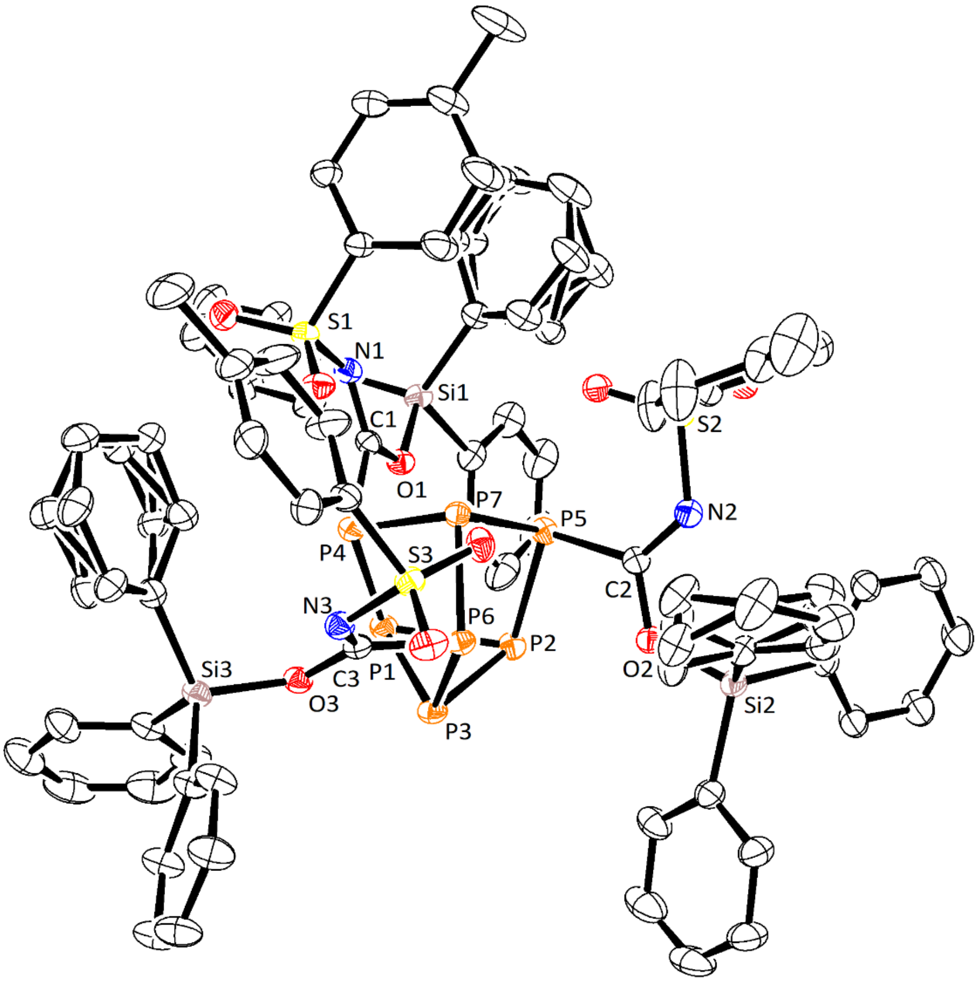

Figure S87. Molecular structure of **18**. Anisotropic displacement ellipsoids pictured at 50% probability. Hydrogen atomsand Et_2O solvent molecules omitted for clarity. Phosphorus: orange; Nitrogen: blue; Oxygen: red; Silicon: pink; Sulfur: yellow; Carbon: white.

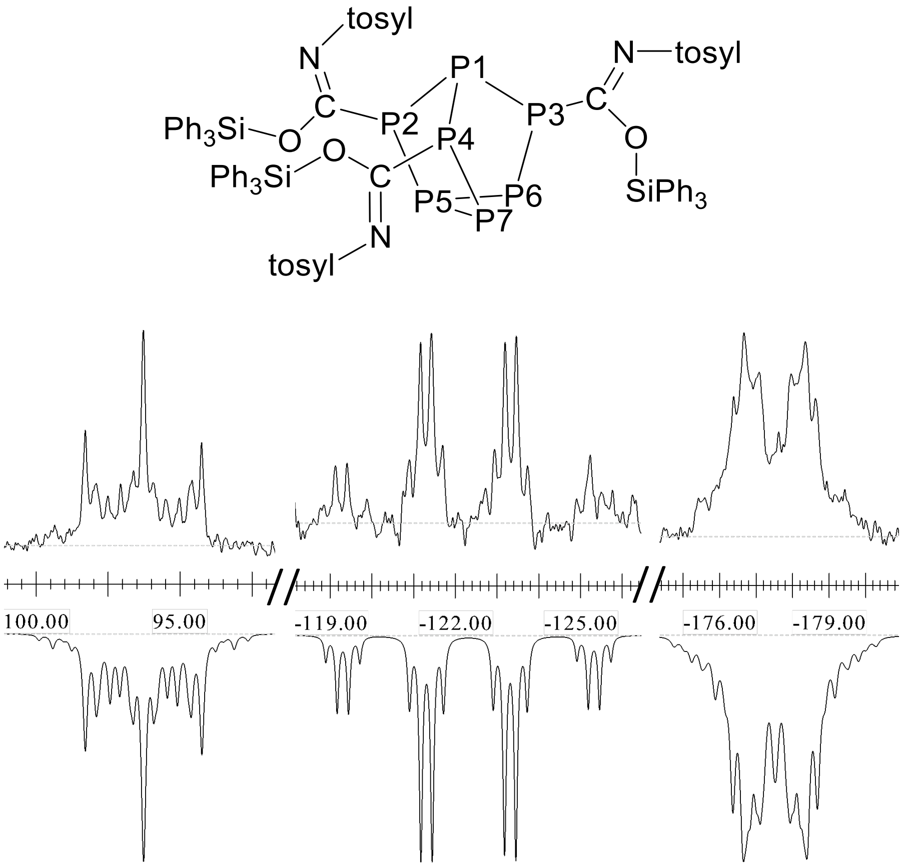

Figure S88. ^{31}P NMR spectra of **18**. Top: experimental, bottom: simulated.

Table S4. Simulated NMR spectroscopic values of **18**.

Nucleus	Chemical shift (ppm)
P1	–122.304
P2, P3, P4	96.253
P5, P6, P7	–177.522
***J* coupling**	**coupling constant (Hz)**
$^1J_{P1–P2}$	324.86
$^1J_{P1–P3}$	324.86
$^1J_{P1–P4}$	324.86
$^2J_{P1–P5}$	44.03
$^2J_{P1–P6}$	44.03
$^2J_{P1–P7}$	44.03
$^1J_{P2–P5}$	331.25
$^1J_{P3–P6}$	331.25
$^1J_{P4–P7}$	331.25
$^1J_{P5–P6}$	213.32
$^1J_{P5–P7}$	213.32
$^1J_{P6–P7}$	213.32
Final residual	3.31e+04

3. Heteroallene Exchange Studies

3.1. Exchange studies

3.1.1. Exchange of PhNCS for PhNCO in 8

A solution of **8** (75 mg, 0.09 mmol, 1.0 eq.) and PhNCO (11 mg, 0.27 mmol, 3.0 eq.) in THF-d_8 (0.5 mL) was sealed in an J Young NMR tube and heated at 50 °C for 1 day. The reaction was monitored by ^{31}P NMR. The solution was cooled to room temperature and solvent removed under reduced pressure. Crystals suitable for X-ray diffraction studies could be obtained by extracting the product in diethyl ether, and was consistent with independently synthesised **2**.

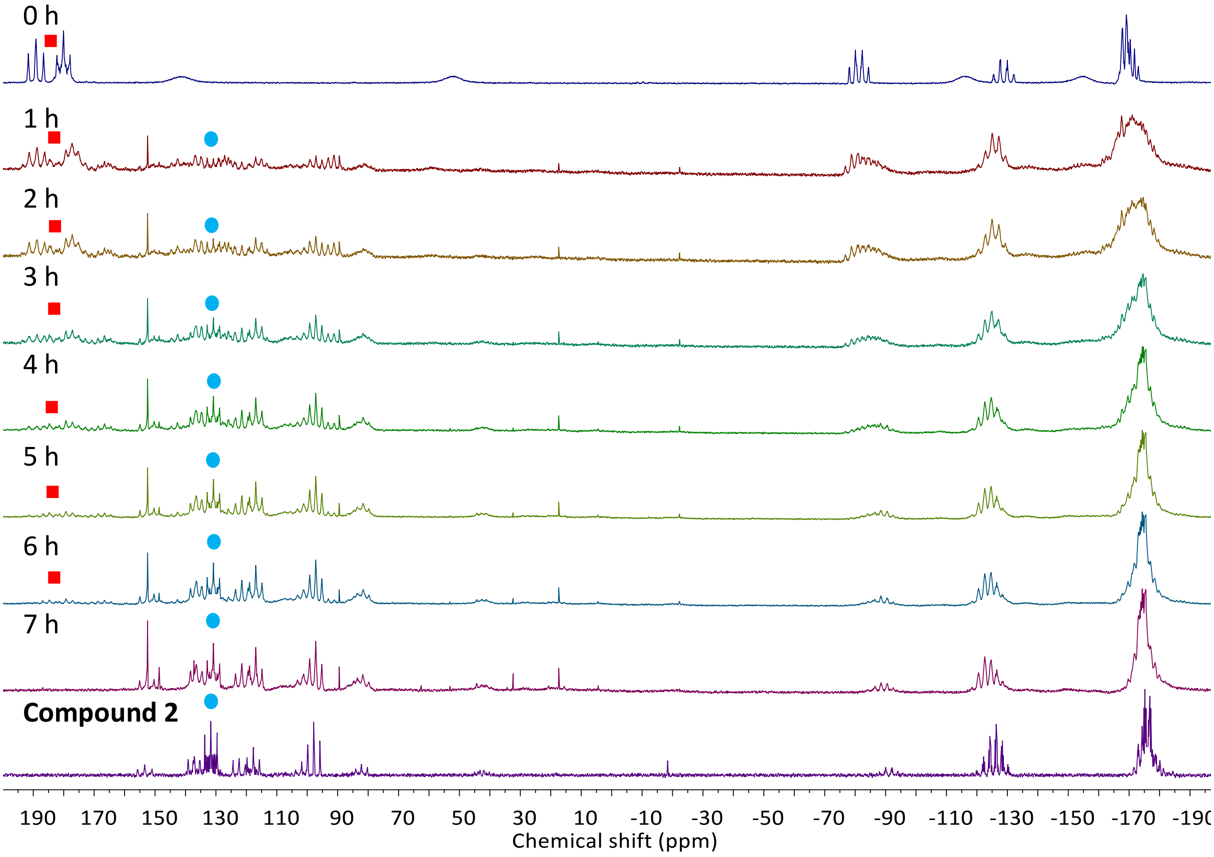

Figure S89. ^{31}P NMR spectra (THF-d_8) of PhNCO addition to **8**.

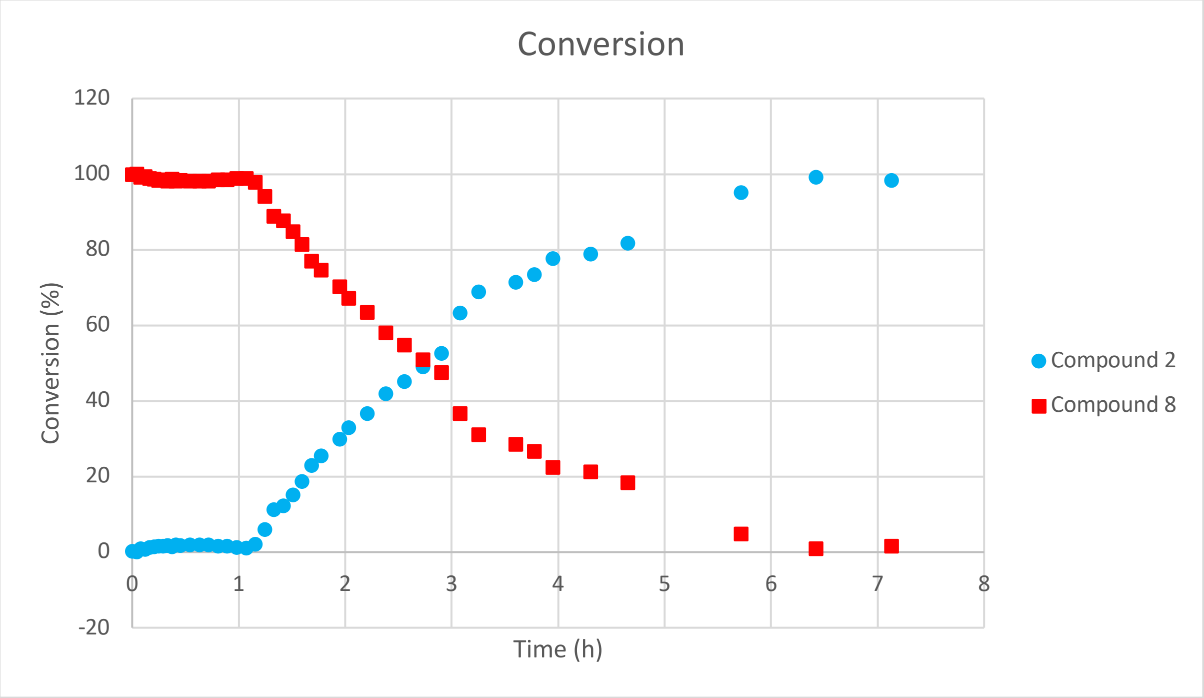

Figure S90. Formation of **2** over time after addition of PhNCO to **8**.

3.1.2. Further exchange studies

Analogously to described above in 3.1.1., a solution of **8** (75 mg, 0.09 mmol, 1.0 eq.) and isocyanate (0.27 mmol, 3.0 eq.) in THF-d_8 (0.5 mL) was sealed in a J Young NMR tube and heated at 50 °C for 1 - 4 days. In case of addition of p-tolylNCO, and 4-MeO(C_6H_4)NCO, crystals suitable for X-ray diffraction studies could be obtained by extracting the product in diethyl ether or pentane. Crystals obtained upon addition of p-tolylNCO, and 4-MeO(C_6H_4)NCO were consistent with independently synthesised **6** and **7**.

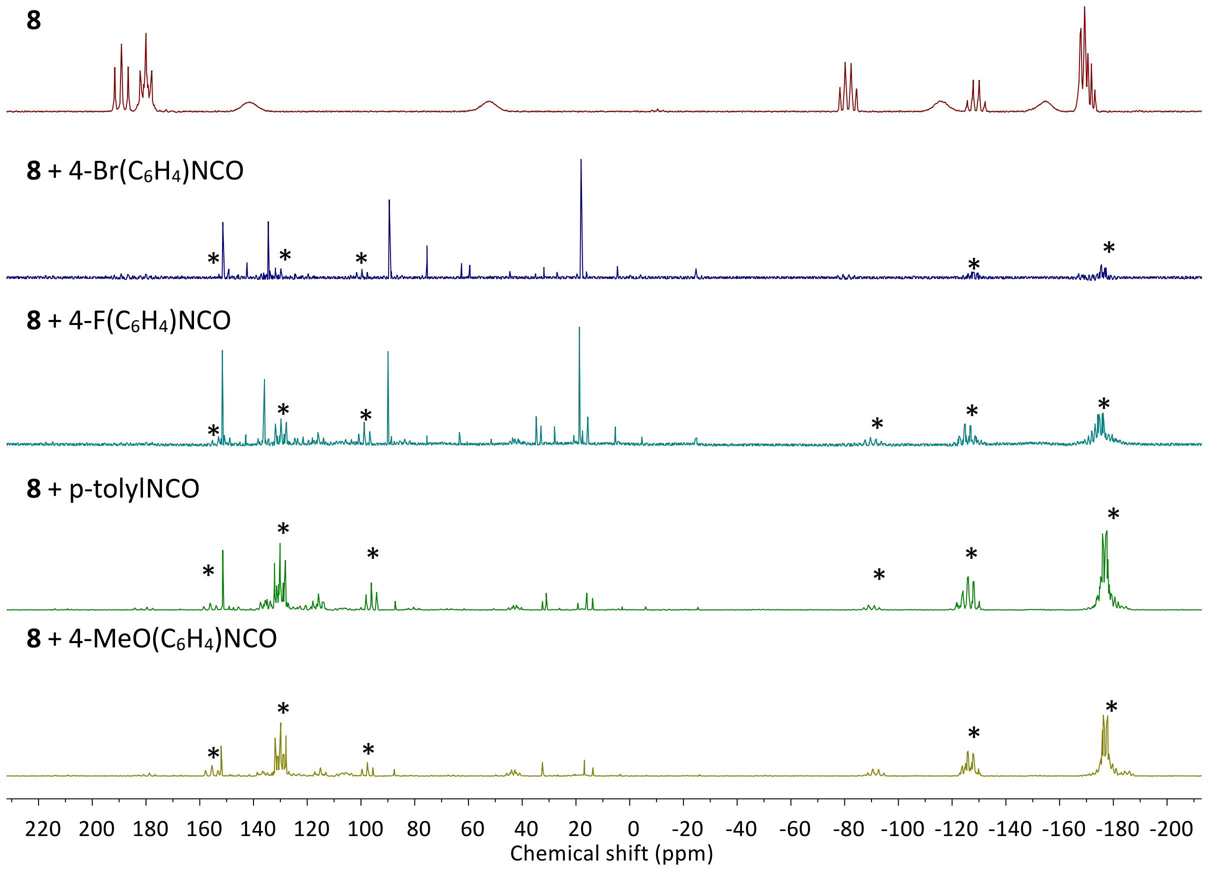

Figure S91. ^{31}P NMR spectra of reversibly studies from addition of RNCO to **8**. NMR spectra recorded after heating at 50 °C. Distinctive resoances for product after exchange marked by *.

4. Crystallography Tables

Table S5. Crystallographic data for molecules **2**, **6** and **7**.

Identification code	**2**	**6**	**7**
Empirical formula	$C_{30}H_{42}N_3O_3P_7Si_3$	$C_{33}H_{48}N_3O_3P_7Si_3$	$C_{33}H_{48}N_3O_6P_7Si_3$
Formula weight	793.72	835.80	883.80
Temperature/K	100.04(11)	100.0(2)	100.02(14)
Crystal system	monoclinic	monoclinic	orthorhombic
Space group	$P2_1/c$	$P2_1/c$	$P2_12_12_1$
a/Å	18.4257(2)	19.9347(4)	19.3067(5)
b/Å	11.9485(2)	38.5269(7)	23.0648(6)
c/Å	18.2308(2)	22.9203(4)	40.3315(9)
α/°	90	90	90
β/°	95.1860(10)	90.222(2)	90
γ/°	90	90	90
Volume/Å^3	3997.25(9)	17603.2(6)	17959.8(8)
Z	4	16	16
ρ_{calc}g/cm^3	1.319	1.261	1.307
μ/mm^{-1}	4.025	3.681	0.398
F(000)	1656.0	7008.0	7392.0
Crystal size/mm^3	0.735 × 0.221 × 0.145	0.531 × 0.46 × 0.11	0.106 × 0.07 × 0.032
Radiation	Cu Kα (λ = 1.54184)	Cu Kα (λ = 1.54184)	Mo Kα (λ = 0.71073)
2Θ range for data collection/°	4.816 to 152.294	4.486 to 152.396	2.92 to 61.538
Index ranges	-22 ≤ h ≤ 21, -14 ≤ k ≤ 11, -21 ≤ l ≤ 15	-24 ≤ h ≤ 15, -48 ≤ k ≤ 40, -28 ≤ l ≤ 28	-24 ≤ h ≤ 27, -31 ≤ k ≤ 25, -56 ≤ l ≤ 54
Reflections collected	23945	110787	153625
Independent reflections	8044 [R_{int} = 0.0195, R_{sigma} = 0.0209]	34704 [R_{int} = 0.0496, R_{sigma} = 0.0493]	44321 [R_{int} = 0.0932, R_{sigma} = 0.1225]
Data/restraints/parameters	8044/0/424	34704/11950/3497	44321/740/2047
Goodness-of-fit on F^2	1.052	1.108	1.055
Final R indexes [I>=2σ (I)]	R_1 = 0.0283, wR_2 = 0.0740	R_1 = 0.1149, wR_2 = 0.2501	R_1 = 0.1002, wR_2 = 0.2416
Final R indexes [all data]	R_1 = 0.0298, wR_2 = 0.0749	R_1 = 0.1552, wR_2 = 0.2735	R_1 = 0.1640, wR_2 = 0.2727
Largest diff. peak/hole / e Å^{-3}	0.39/-0.28	1.01/-0.59	1.38/-0.82
Flack parameter	-	-	0.54(14)
CCDC	2101379	2101381	2101382

Table S6. Crystallographic data for molecules **8**, **9** and **10**.

Identification code	**8**	**9**	**10**
Empirical formula	$C_{30}H_{42}N_3P_7S_3Si_3$	$C_{33}H_{48}N_3O_9P_7S_3Si_3$	$C_{24}H_{33}P_7Si_3$
Formula weight	841.90	1027.98	622.56
Temperature/K	100.02(12)	100.00(10)	150.00(10)
Crystal system	orthorhombic	triclinic	trigonal
Space group	$Pna2_1$	P-1	R3
a/Å	17.4304(9)	13.0370(4)	19.9733(9)
b/Å	13.5633(3)	13.4130(4)	19.9733(9)
c/Å	17.6209(3)	13.8084(5)	6.7702(4)
α/°	90	94.629(3)	90
β/°	90	93.791(3)	90
γ/°	90	92.156(3)	120
Volume/Å^3	4165.8(2)	2399.23(14)	2339.0(3)
Z	4	2	3
ρ_{calc}g/cm^3	1.342	1.423	1.326
μ/mm^{-1}	5.207	0.513	0.525
F(000)	1752.0	1068.0	972.0
Crystal size/mm^3	0.3 × 0.3 × 0.2	0.11 × 0.06 × 0.05	0.39 × 0.06 × 0.06
Radiation	Cu Kα (λ = 1.54184)	Mo Kα (λ = 0.71073)	Mo Kα (λ = 0.71073)
2Θ range for data collection/°	8.226 to 151.764	4.074 to 61.076	4.078 to 61.048
Index ranges	-14 ≤ h ≤ 21, -16 ≤ k ≤ 17, -21 ≤ l ≤ 22	-16 ≤ h ≤ 18, -18 ≤ k ≤ 18, -19 ≤ l ≤ 18	-28 ≤ h ≤ 27, -28 ≤ k ≤ 27, -9 ≤ l ≤ 9
Reflections collected	32869	35628	9541
Independent reflections	7892 [R_{int} = 0.0215, R_{sigma} = 0.0176]	11801 [R_{int} = 0.0397, R_{sigma} = 0.0512]	9541 [R_{int} = 0.068, R_{sigma} = 0.0966]
Data/restraints/parameters	7892/1/424	11801/0/535	9541/1/106
Goodness-of-fit on F^2	0.786	1.041	1.007
Final R indexes [I>=2σ (I)]	R_1 = 0.0263, wR_2 = 0.0810	R_1 = 0.0364, wR_2 = 0.0800	R_1 = 0.0464, wR_2 = 0.1121
Final R indexes [all data]	R_1 = 0.0264, wR_2 = 0.0812	R_1 = 0.0576, wR_2 = 0.0864	R_1 = 0.0505, wR_2 = 0.1145
Largest diff. peak/hole / e Å^{-3}	0.66/-0.38	0.42/-0.42	0.52/-0.30
Flack parameter	0.064(5)	-	0.13(10)
CCDC	2101376	2101377	2101378

Table S7. Crystallographic data for molecules **15** and **18**.

Identification code	**11**	**15**	**18**
Empirical formula	$C_{121}H_{127}OP_{21}Si_9$	$C_{103}H_{130}N_3O_{10}P_7Si_3$	$C_{96}H_{111}N_3O_{13.5}P_7S_3Si_3$
Formula weight	2500.40	1871.15	1920.11
Temperature/K	100.00(10)	99.99(10)	100.01(10)
Crystal system	monoclinic	trigonal	trigonal
Space group	$P2_1/c$	R-3	R3c
a/Å	37.4665(16)	20.8497(15)	18.8973(3)
b/Å	7.7571(6)	20.8497(15)	18.8973(3)
c/Å	43.9966(17)	40.437(6)	48.3946(6)
α/°	90	90	90
β/°	96.227(4)	90	90
γ/°	90	120	120
Volume/Å^3	12711.3(12)	15223(3)	14966.7(5)
Z	4	6	6
ρ_{calc}g/cm^3	1.307	1.225	1.278
μ/mm^{-1}	3.760	1.930	2.578
F(000)	5208.0	5976.0	6066.0
Crystal size/mm^3	0.15 × 0.01 × 0.003	0.5 × 0.44 × 0.38	0.13 × 0.13 × 0.13
Radiation	Cu Kα (λ = 1.54184)	Cu Kα (λ = 1.54184)	Cu Kα (λ = 1.54184)
2Θ range for data collection/°	4.04 to 152.81	5.36 to 153.648	6.52 to 152.152
Index ranges	-46 ≤ h ≤ 47, -9 ≤ k ≤ 9, -55 ≤ l ≤ 53	-26 ≤ h ≤ 26, -25 ≤ k ≤ 18, -43 ≤ l ≤ 47	-17 ≤ h ≤ 23, -22 ≤ k ≤ 17, -60 ≤ l ≤ 58
Reflections collected	77767	19176	28527
Independent reflections	25500 [R_{int} = 0.0998, R_{sigma} = 0.1096]	6610 [R_{int} = 0.0649, R_{sigma} = 0.0721]	6550 [R_{int} = 0.0296, R_{sigma} = 0.0230]
Data/restraints/parameters	25500/552/1443	6610/262/390	6550/419/495
Goodness-of-fit on F^2	1.028	1.179	1.058
Final R indexes [I>=2σ (I)]	R_1 = 0.0801, wR_2 = 0.1656	R_1 = 0.1067, wR_2 = 0.2906	R_1 = 0.0454, wR_2 = 0.1248
Final R indexes [all data]	R_1 = 0.1337, wR_2 = 0.1881	R_1 = 0.1274, wR_2 = 0.3103	R_1 = 0.0471, wR_2 = 0.1268
Largest diff. peak/hole / e Å^{-3}	0.77/-0.64	1.27/-0.64	0.64/-0.35
Flack parameter	-	-	0.14(3)
CCDC	2110872	2101374	2101380

5. Computational studies

5.1. Energies of 1 and 2 Symmetric and Asymmetric Isomers

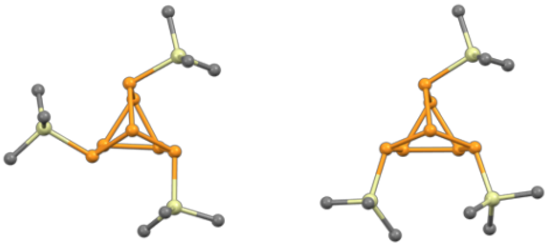

Sym A   Asym B

ΔE_{rel} = 0.0 kJ mol^{-1} ΔE_{rel} = 35.4 kJ mol^{-1}

Figure S92. Top views of the calculated stationary points **1** symmetric and asymmetric isomers. Peformed at the PBE1PBE/6-31G(d,p) level.

Table S8. Computed energies isomerism of **1**. Peformed at the PBE1PBE/6-31G(d,p) level.

Description	E [a.u.]	ΔE [kJ/mol]	G [a.u.]	ΔG [kJ/mol]
**1** Sym (**A**)	–3615.595510	0	–3615.208112	0
**1** Asym (**B**)	–3615.582121	35.1524	–3615.194619	35.4

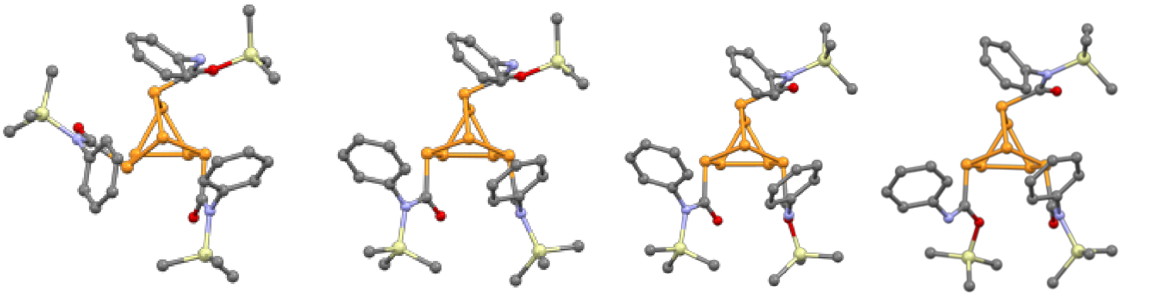

Sym A   Asym B1   Asym B2   Asym B3

ΔE_{rel} = 0.0 kJ mol^{-1} ΔE_{rel} = 25.3 kJ mol^{-1} ΔE_{rel} = 10.9 kJ mol^{-1} ΔE_{rel} = 22.0 kJ mol^{-1}

Figure S93. Top views of the calculated stationary points **2** symmetric and asymmetric isomers.

Table S9. Computed energies isomerism of **2**. Peformed at the PBE1PBE/6-31G(d,p) level.

Description	E [a.u.]	ΔE [kJ/mol]	G [a.u.]	ΔG [kJ/mol]
**2** Sym (**A**)	–4813.518257	0	–4812.786313	0
**2** Asym (**B1**)	–4813.508637	25.25738877	–4812.77668	25.2914
**2** Asym (**B2**)	–4813.514102	10.90811234	–4812.78216	10.9037
**2** Asym (**B3**)	–4813.510092	21.43707622	–4812.777922	22.0306

5.2. Fluoride Ion Affinity of Silyl Unit on compounds 1, 10-12

The Lewis acidity of the silyl substituents was probed by calculation of its Fluoride Ion Affinity (FIA) using the CF_2O-CF_3O^- reference system (also known as Christe's method).[7] This method has been extensively used in literature using the BP86/SV(p) level of theory. The calculated FIA of $B(C_6F_5)_3$, SbF_5 and Me_3Si^+ are consistent with literature values and used as benchmarks.[8]

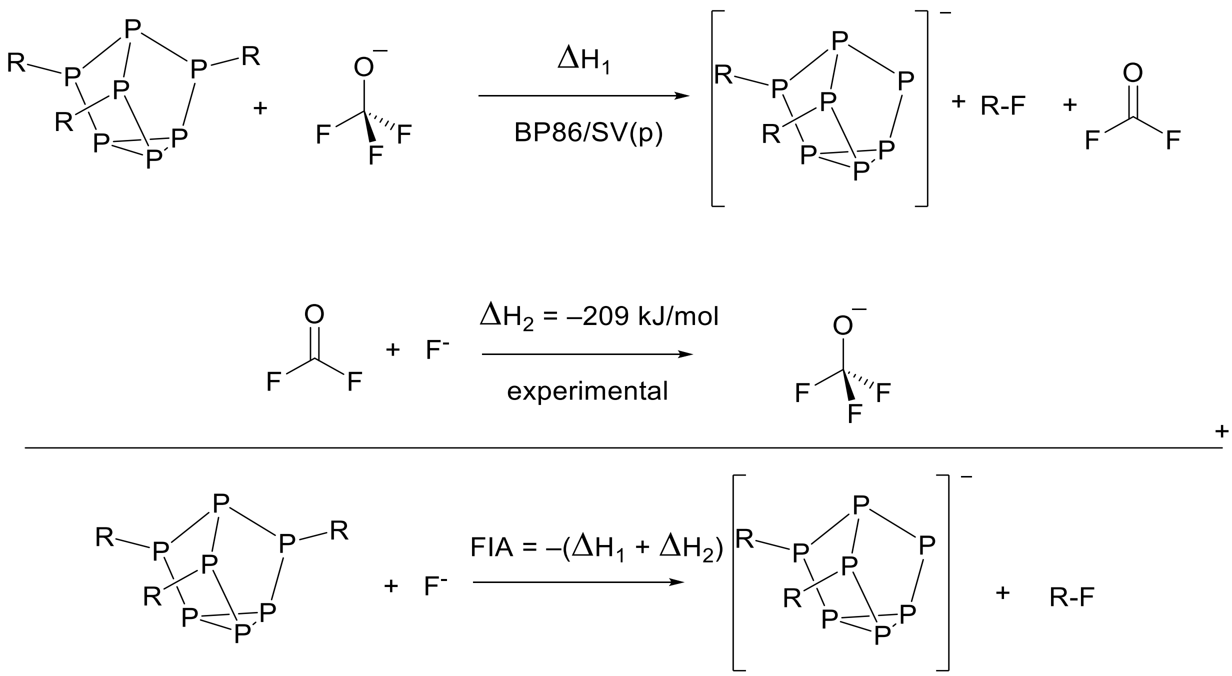

Scheme S1. FIA investigation of **1**, **10-12**. Peformed at the BP86/SV(p) level of theory.

Table S10. FIA values of selected Lewis Acids and **1**, **10**-**12**. Peformed at the BP86/SV(p) level of theory.

Compound	FIA (kJ mol^{-1})
$B(C_6F_5)_3$	444
SbF_5	489
$[Me_3Si]^+$	948
$(Me_3Si)_3P_7$ (**1**)	324
$(Me_2PhSi)_3P_7$ (**10**)	335
$(MePh_2Si)_3P_7$ (**11**)	349
$(Ph_3Si)_3P_7$ (**12**)	353

Table S11. Computed energies of selected Lewis Acids and **1**, **10**-**12**. Peformed at the BP86/SV(p) level of theory.

Compound	E [a.u.]	G [a.u.]
CF_2O	–312.7854163	–312.76742
$[CF_3O]^-$	–412.6141114	–412.594474
$B(C_6F_5)_3$	–2206.628214	–2206.448141
$[FB(C_6F_5)_3]^-$	–2306.547159	–2306.364788
SbF_5	–6814.717684	–6814.698435
$[SbF_6]^-$	–6914.652886	–6914.631992
$[Me_3Si]^+$	–408.8174622	–408.70319
Me_3SiF	–508.9311529	–508.81189
$(Me_3Si)_3P_7$	–3616.461346	–3616.088086
$[(Me_3Si)_2P_7]^-$	–3207.402141	–3207.147222
Me_2PhSiF	–700.5259647	–700.350769
$(Me_2PhSi)_3P_7$	–4191.242756	–4190.701655
$[(Me_2PhSi)_2P_7]^-$	–3590.59256	–3590.225988
$MePh_2SiF$	–892.1205369	–891.889217
$(MePh_2Si)_3P_7$	-4766.0218852	-4765.313120
$[(MePh_2Si)_{32}P_7]$	–3973.782604	–3973.304203
Ph_3SiF	–1083.715251	–1083.427972
$(Ph_3Si)_3P_7$	–5340.805342	–5339.928588
$[(Ph_3Si)_2P_7]$	–4356.972733	–4356.382673

5.3. Heteroallene exchange

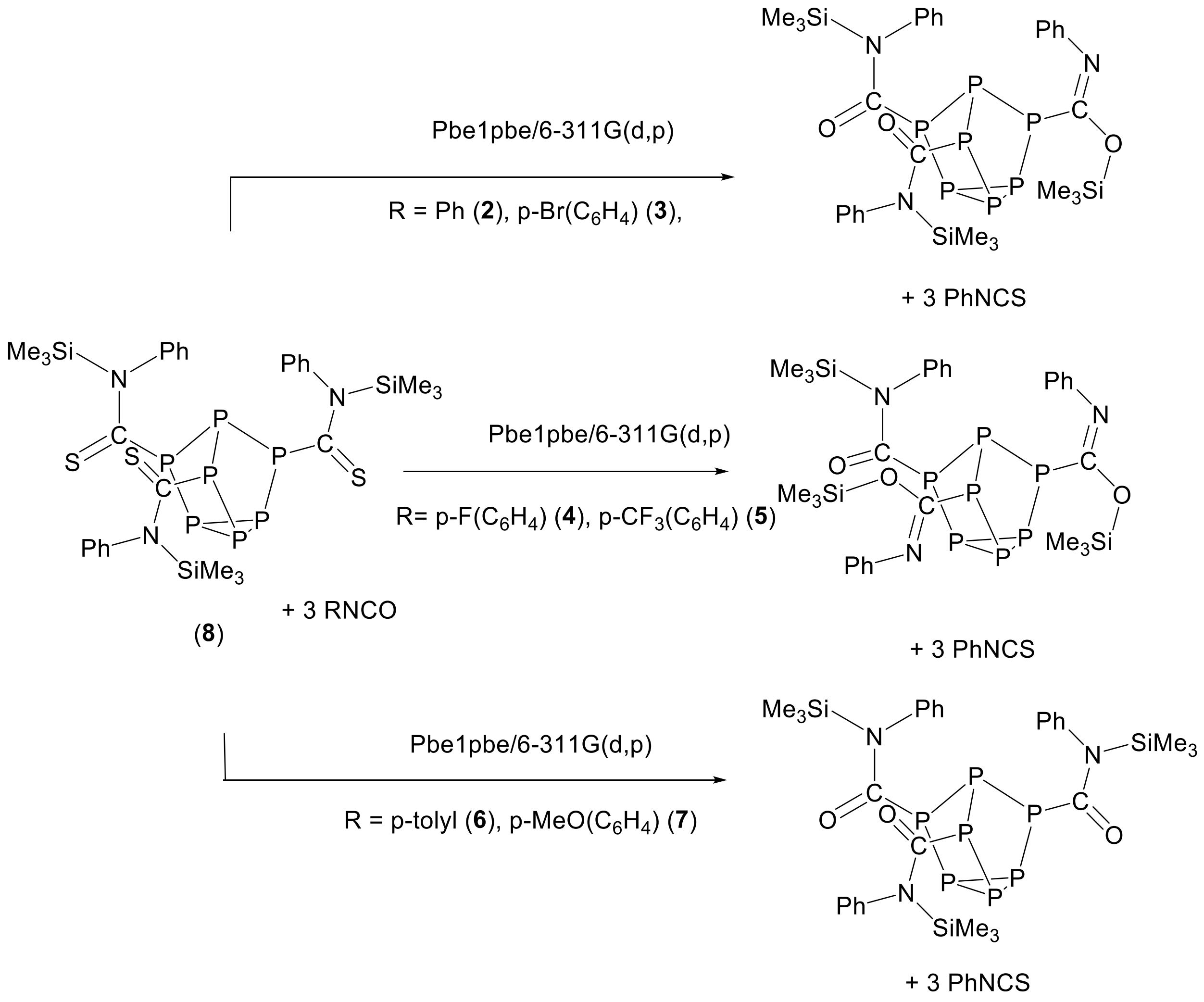

Scheme S2. Heteroallene of isothiocyanates captured at **8** for isocyanates. Peformed at the PBE1PBE/6-311G(d,p) level of theory.

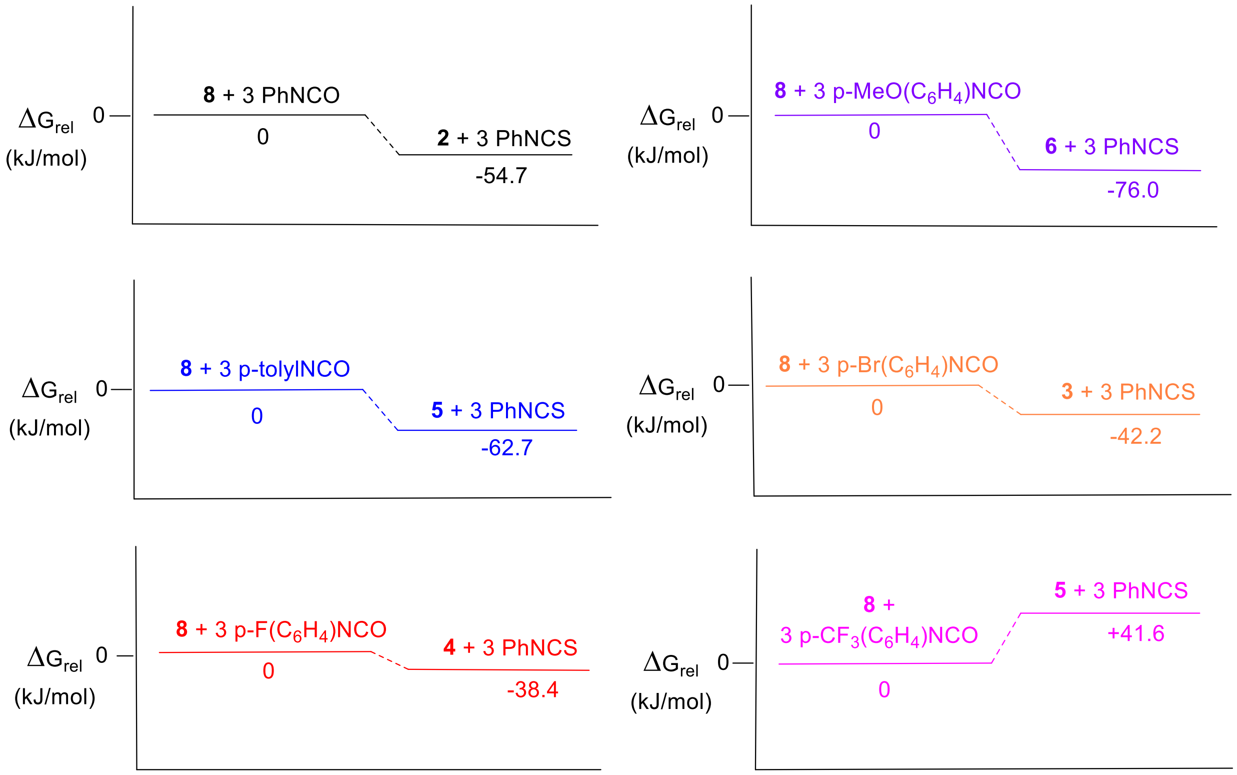

Figure S94. Computed energies for exchange of isothiocyanates captured at **8** for isocyanates. Peformed at PBE1PBE/6-311G(d,p) level of theory.

Table S12. Computed energies for exchange of isothiocyanates captured at **8** for isocyanates. Peformed at PBE1PBE/6-311G(d,p) level of theory

Description	E [a.u.]	ΔE [kJ/mol]	G [a.u.]	ΔG [kJ/mol]
**8** + 3 PhNCO	-6980.769667	0.00	-6979.710750	0.00
**2** + 3 PhNCS	-6980.790683	-55.18	-6979.731588	-54.71
**8** + 3 p-MeO(C_6H_4)NCO	-7324.050465	0.00	-7322.886018	0.00
**6** + 3 PhNCS	-7324.078164	-72.72	-7322.914952	-75.97
**8** + 3 p-TolylNCO	-7098.603887	0.00	-7097.457477	0.00
**5** + 3 PhNCS	-7098.627297	-61.46	-7097.481375	-62.74
**8** + 3 p-Br(C_6H_4)NCO	-14700.438034	0.00	-14699.408445	0.00
**3** + 3 PhNCS	-14700.456227	-47.77	-14699.424525	-42.22
**8** + 3 p-F(C_6H_4)NCO	-7278.301234	0.00	-7277.263074	0.00
**4** + 3 PhNCS	-7278.314457	-34.72	-7277.277695	-38.39
**8** + 3 p-CF_3(C_6H_4)NCO	-7991.283447	0.00	-7990.198986	0.00
**5** + 3 PhNCS	-7991.267570	41.69	-7990.183157	41.56

5.4. Geometry optimised structures

5.4.1. $(Me_3Si)_3P_7$ (1) symmetric isomer

Charge = 0 Multiplicity = 1

P	-0.4531200000	0.0273000000	-0.3213600000
P	0.5239630000	0.2945230000	-2.3048660000
P	0.5912810000	-1.8422420000	0.2898830000
P	-2.3536600000	-0.8906030000	-1.0342170000
P	0.2145770000	-3.2067080000	-1.4476510000
P	-1.6369940000	-2.4250850000	-2.5008900000
P	0.4233020000	-1.7631900000	-3.1850560000
Si	2.7932550000	0.5756030000	-1.8827070000
Si	-0.6831780000	-2.8014950000	1.9817350000
Si	-3.3674480000	0.6933460000	-2.4059720000
C	-3.1718340000	2.3701160000	-1.5427920000
C	-5.1926350000	0.1711030000	-2.4241120000
C	-2.7088550000	0.7554940000	-4.1794280000
C	-1.1547630000	-1.4012860000	3.1710980000
C	0.5269180000	-3.9966870000	2.8253900000
C	-2.2137600000	-3.7464690000	1.3947690000
C	2.9268090000	1.7491280000	-0.3987760000
C	3.4410450000	1.4247780000	-3.4523500000
C	3.7627560000	-1.0213040000	-1.5793210000
H	2.3400790000	2.6773750000	-0.5579190000
H	3.9892070000	2.0376150000	-0.2434130000
H	2.5620310000	1.2653650000	0.5305650000
H	3.3889130000	-1.5510410000	-0.6797870000
H	4.8370210000	-0.7771560000	-1.4267490000
H	3.6880050000	-1.7114100000	-2.4451850000
H	2.9042890000	2.3751110000	-3.6521910000
H	3.3229930000	0.7717400000	-4.3422770000

H	4.5232630000	1.6550150000	-3.3426420000
H	0.0341280000	-4.4926970000	3.6898350000
H	1.4259060000	-3.4645560000	3.1993170000
H	0.8648950000	-4.7905970000	2.1270070000
H	-1.9405850000	-4.5551670000	0.6856300000
H	-2.9294030000	-3.0686290000	0.8867220000
H	-2.7210160000	-4.2111280000	2.2685580000
H	-1.6317540000	-1.8273650000	4.0805470000
H	-1.8747100000	-0.6985020000	2.7037130000
H	-0.2645590000	-0.8217530000	3.4920140000
H	-5.7885580000	0.8899810000	-3.0276750000
H	-5.6186910000	0.1442990000	-1.3999100000
H	-5.3203790000	-0.8356770000	-2.8740320000
H	-3.7553890000	3.1431940000	-2.0885240000
H	-2.1095470000	2.6890280000	-1.5250010000
H	-3.5411550000	2.3369040000	-0.4970530000
H	-1.6345440000	1.0296740000	-4.1980530000
H	-3.2792830000	1.5146430000	-4.7583970000
H	-2.8268180000	-0.2235860000	-4.6884810000

5.4.2. $(Me_3Si)_3P_7$ (1) asymmetric isomer
Charge = 0 Multiplicity = 1

P	0.2886930000	0.2533260000	-0.0152310000
P	0.0520460000	-1.5233120000	-1.2850290000
P	-1.0295840000	1.8401580000	-0.7912230000
P	1.9103130000	1.1557490000	-1.2099020000
P	-1.0483910000	1.4447820000	-2.9483400000
P	1.1023120000	0.9871120000	-3.2856370000
P	-0.3802900000	-0.6787020000	-3.2521280000
Si	-1.6795150000	-2.8957250000	-0.6840770000
Si	-3.2023630000	1.7598850000	-0.1103560000

Si	3.6619980000	-0.3318740000	-1.2350790000
C	-2.3544030000	-2.3993600000	1.0022280000
C	-0.8662240000	-4.5873240000	-0.5280980000
C	-2.9949420000	-2.9794690000	-2.0275970000
C	-4.3031830000	0.4732290000	-0.9312180000
C	-3.8395820000	3.4699900000	-0.5742040000
C	-3.1526370000	1.5707160000	1.7621830000
C	5.1511360000	0.7760230000	-1.5627820000
C	3.5677460000	-1.6621290000	-2.5600780000
C	3.7723730000	-1.0915430000	0.4834570000
H	2.9254610000	-1.7565980000	0.6757960000
H	3.7897400000	-0.3246090000	1.2635260000
H	4.6931790000	-1.6800610000	0.5678980000
H	5.0660670000	1.2811910000	-2.5302110000
H	6.0720060000	0.1818630000	-1.5755070000
H	5.2506240000	1.5448020000	-0.7908550000
H	3.4564010000	-1.2297390000	-3.5593920000
H	2.7262760000	-2.3355800000	-2.3794520000
H	4.4941950000	-2.2487230000	-2.5506180000
H	-0.0820140000	-4.5804990000	0.2349990000
H	-0.4099130000	-4.8950170000	-1.4739110000
H	-1.6074230000	-5.3445970000	-0.2476630000
H	-2.5599840000	-3.3653810000	-2.9550040000
H	-3.4439390000	-2.0091060000	-2.2507500000
H	-3.7940600000	-3.6656520000	-1.7234310000
H	-3.0717920000	-3.1524580000	1.3481460000
H	-2.8600880000	-1.4314670000	0.9906200000
H	-1.5457190000	-2.3472980000	1.7382220000
H	-4.1685380000	1.6093680000	2.1712170000
H	-2.5787930000	2.3865620000	2.2123690000

H	-2.6954750000	0.6284730000	2.0773160000
H	-4.0348590000	-0.5495490000	-0.6607460000
H	-4.2543440000	0.5633660000	-2.0212350000
H	-5.3440610000	0.6389150000	-0.6294470000
H	-4.8776700000	3.5880570000	-0.2427210000
H	-3.8140800000	3.6247970000	-1.6575150000
H	-3.2396990000	4.2564090000	-0.1075390000

5.4.3. (TMS-PhNCO)$_3$P$_7$ (2) symmetric isomer

5.4.3.1. Peformed at the PBE1PBE/6-31G(d,p) level of theory.

Charge = 0 Multiplicity = 1

P	0.0320510000	-0.0511370000	-0.1550610000
P	1.2153560000	0.8925210000	-1.7630080000
P	0.2612040000	-2.1331750000	-0.8549200000
P	-1.9719240000	0.3231580000	-1.0051070000
P	-0.4843450000	-2.0324970000	-2.9536400000
P	-1.7030870000	-0.1899990000	-3.1547720000
P	0.4656730000	-0.1351970000	-3.5958030000
Si	5.1916750000	-0.7942740000	-0.4390980000
Si	-3.7282120000	-3.8314890000	-0.1472050000
Si	-1.8974970000	4.7798390000	-0.3008840000
O	-2.2180550000	-3.2137980000	-0.7338480000
O	3.1825510000	-0.8372670000	-2.5061190000
O	-1.7923310000	2.7141820000	-2.3108100000
N	3.6371450000	0.1561830000	-0.5214690000
N	-2.0240520000	2.9754990000	-0.0720180000
N	-1.2308280000	-3.1101890000	1.3075430000
C	2.8485950000	-0.0729100000	-1.6125890000
C	-2.3071870000	2.3912160000	1.2000920000
C	3.2997470000	1.1397400000	0.4565410000
C	-1.1982750000	-2.8923610000	0.0542550000

C	-1.9074220000	2.2161450000	-1.2009130000
C	-0.2612880000	-1.8797900000	3.1486060000
H	-1.1750870000	-1.2964470000	3.2044670000
C	-0.1419230000	-2.8542190000	2.1507460000
C	1.0307360000	-3.6179990000	2.0794770000
H	1.1118920000	-4.3932150000	1.3237290000
C	2.8482380000	0.7490600000	1.7189220000
H	2.7103920000	-0.3057440000	1.9386770000
C	1.9544380000	-2.4110840000	3.9596320000
H	2.7625020000	-2.2455990000	4.6660640000
C	-4.7078160000	-3.9456590000	-1.7362920000
H	-4.8134020000	-2.9603190000	-2.2007050000
H	-5.7120230000	-4.3415850000	-1.5515060000
H	-4.2133010000	-4.6036940000	-2.4573110000
C	2.0699130000	-3.3918870000	2.9766020000
H	2.9705390000	-3.9964940000	2.9128720000
C	0.7831320000	-1.6593700000	4.0392110000
H	0.6755490000	-0.9009530000	4.8104790000
C	3.4589610000	2.4976760000	0.1678960000
H	3.8167870000	2.7908880000	-0.8144720000
C	4.7884100000	-2.6214870000	-0.3769130000
H	4.1487790000	-2.8503760000	0.4817250000
H	5.7069710000	-3.2114460000	-0.2841250000
H	4.2652040000	-2.9313790000	-1.2842760000
C	-1.3004290000	2.2740820000	2.1594650000
H	-0.2895480000	2.5841310000	1.9120280000
C	6.0250560000	-0.2501370000	1.1536530000
H	6.1942000000	0.8306120000	1.1806530000
H	7.0016130000	-0.7422410000	1.2265820000
H	5.4474640000	-0.5212920000	2.0417760000

C	6.2689470000	-0.3347030000	-1.9024540000
H	5.7695220000	-0.5862450000	-2.8405490000
H	7.2207140000	-0.8755790000	-1.8577460000
H	6.4943530000	0.7366160000	-1.9049620000
C	-3.6061860000	1.9759220000	1.5049540000
H	-4.3827670000	2.0777220000	0.7526170000
C	3.1580410000	3.4566030000	1.1306000000
H	3.2844370000	4.5098770000	0.8977170000
C	-1.5936840000	1.7450420000	3.4127400000
H	-0.8045200000	1.6567450000	4.1538460000
C	-4.4842580000	-2.5959080000	1.0365700000
H	-3.9274050000	-2.5645190000	1.9757960000
H	-5.5250550000	-2.8612460000	1.2529840000
H	-4.4700770000	-1.5920310000	0.5999290000
C	2.7136000000	3.0653570000	2.3918680000
H	2.4924720000	3.8139490000	3.1473120000
C	2.5616860000	1.7112760000	2.6823590000
H	2.2139670000	1.3951680000	3.6615940000
C	-3.4612100000	-5.5175480000	0.6191800000
H	-2.9718300000	-6.1966640000	-0.0860490000
H	-4.4217390000	-5.9637100000	0.8998660000
H	-2.8395450000	-5.4426120000	1.5146100000
C	-3.8916170000	1.4365840000	2.7548130000
H	-4.9018460000	1.1096220000	2.9821010000
C	-2.8869400000	1.3246870000	3.7133510000
H	-3.1127070000	0.9123190000	4.6924260000
C	-3.3116410000	5.3641860000	-1.3834940000
H	-3.2666910000	4.8816690000	-2.3624250000
H	-3.2574640000	6.4490380000	-1.5267610000
H	-4.2804300000	5.1362500000	-0.9274150000

C	-2.0655870000	5.5146170000	1.4191490000
H	-3.0093620000	5.2318500000	1.8952480000
H	-2.0450750000	6.6075760000	1.3414150000
H	-1.2516660000	5.2115280000	2.0837940000
C	-0.2175700000	5.1758700000	-1.0277400000
H	0.5855390000	4.8137700000	-0.3775440000
H	-0.0969700000	6.2580900000	-1.1485060000
H	-0.1038020000	4.7004100000	-2.0045720000

5.4.3.2. Peformed at the PBE1PBE/6-311G(d,p) level of theory.

P	0.0372110000	-0.0457530000	-0.1333470000
P	1.1469840000	0.9125270000	-1.7889380000
P	0.2968270000	-2.1327950000	-0.8221440000
P	-2.0003830000	0.2669810000	-0.9345550000
P	-0.4964450000	-2.0731140000	-2.9107110000
P	-1.7883840000	-0.2761860000	-3.0891570000
P	0.3651870000	-0.1472370000	-3.5984380000
Si	5.0939250000	-0.8399700000	-0.4938650000
Si	-3.7197350000	-3.7584140000	-0.1112390000
Si	-1.8773200000	4.7285370000	-0.3245820000
O	-2.1868460000	-3.1891720000	-0.6728300000
O	3.0981580000	-0.8161340000	-2.5593190000
O	-1.8948870000	2.6276940000	-2.2904280000
N	3.5669950000	0.1506210000	-0.5717670000
N	-1.9977740000	2.9321640000	-0.0533720000
N	-1.1816600000	-3.0797720000	1.3532890000
C	2.7756090000	-0.0585360000	-1.6633240000
C	-2.2323690000	2.3683490000	1.2368940000
C	3.2671630000	1.1650550000	0.3859220000
C	-1.1594380000	-2.8721490000	0.1018590000

C	-1.9479380000	2.1527990000	-1.1718990000
C	-0.1658140000	-1.8103360000	3.1405900000
H	-1.0620410000	-1.2011870000	3.1825510000
C	-0.0806830000	-2.8193500000	2.1779160000
C	1.0718090000	-3.6092620000	2.1189410000
H	1.1290390000	-4.4064730000	1.3856750000
C	2.8462890000	0.8203690000	1.6690910000
H	2.6924170000	-0.2230570000	1.9232450000
C	2.0377710000	-2.3719170000	3.9519310000
H	2.8539170000	-2.2061130000	4.6471760000
C	-4.6813310000	-3.8414620000	-1.7060390000
H	-4.7518890000	-2.8540680000	-2.1712790000
H	-5.6984030000	-4.2055200000	-1.5294960000
H	-4.2013140000	-4.5152600000	-2.4212820000
C	2.1217140000	-3.3820920000	2.9994180000
H	3.0071150000	-4.0076240000	2.9460310000
C	0.8886120000	-1.5897560000	4.0155420000
H	0.8066680000	-0.8053700000	4.7618510000
C	3.4362220000	2.5089760000	0.0530870000
H	3.7663490000	2.7683690000	-0.9471050000
C	4.6485730000	-2.6532020000	-0.4999010000
H	3.9298180000	-2.8778820000	0.2939100000
H	5.5444900000	-3.2604830000	-0.3324990000
H	4.2030840000	-2.9425950000	-1.4531560000
C	-1.2022780000	2.2977730000	2.1716500000
H	-0.2049320000	2.6194010000	1.8917440000
C	5.9144920000	-0.3743910000	1.1246350000
H	6.0934280000	0.7016090000	1.2028490000
H	6.8852880000	-0.8788540000	1.1811360000
H	5.3282190000	-0.6826240000	1.9939890000

C	6.2023590000	-0.3591920000	-1.9207200000
H	5.7119750000	-0.5635730000	-2.8742790000
H	7.1365970000	-0.9291920000	-1.8814740000
H	6.4581070000	0.7039510000	-1.8802030000
C	-3.5089310000	1.9249380000	1.5815750000
H	-4.3052600000	1.9873430000	0.8474630000
C	3.1846710000	3.4988490000	0.9947060000
H	3.3202250000	4.5413960000	0.7267510000
C	-1.4524710000	1.7969450000	3.4428950000
H	-0.6453240000	1.7483540000	4.1663530000
C	-4.4423410000	-2.5005200000	1.0614110000
H	-3.9209250000	-2.5140000000	2.0205400000
H	-5.5036540000	-2.7074920000	1.2344060000
H	-4.3511910000	-1.4952700000	0.6388760000
C	2.7785100000	3.1528690000	2.2789560000
H	2.5979800000	3.9255980000	3.0191490000
C	2.6109040000	1.8130470000	2.6116450000
H	2.2915380000	1.5327780000	3.6100280000
C	-3.5120210000	-5.4434710000	0.6619370000
H	-3.0469800000	-6.1434640000	-0.0383150000
H	-4.4876810000	-5.8521340000	0.9453640000
H	-2.8894710000	-5.3841910000	1.5572980000
C	-3.7509770000	1.4113540000	2.8486870000
H	-4.7452700000	1.0629190000	3.1067090000
C	-2.7246480000	1.3531430000	3.7850650000
H	-2.9167670000	0.9619570000	4.7786560000
C	-3.3430310000	5.2971880000	-1.3362040000
H	-3.3489950000	4.8086040000	-2.3122680000
H	-3.2971310000	6.3805860000	-1.4893310000
H	-4.2853000000	5.0720400000	-0.8275320000

C	-1.9512130000	5.4994990000	1.3814940000
H	-2.8600080000	5.2157660000	1.9197460000
H	-1.9524040000	6.5898100000	1.2757940000
H	-1.0945290000	5.2253790000	2.0024910000
C	-0.2430860000	5.1033400000	-1.1472760000
H	0.5923210000	4.7708370000	-0.5239780000
H	-0.1400110000	6.1812770000	-1.3104530000
H	-0.1713150000	4.5966000000	-2.1115530000

5.4.4. $(TMS\text{-}PhNCO)_3P_7$ (2) asymmetric isomer B1

Charge = 0 Multiplicity = 1

P	-0.4285500000	-0.7262280000	-0.0968400000
P	0.8782390000	-1.4583880000	-1.7219830000
P	-2.0965640000	-1.6353020000	-1.2297450000
P	-0.9624180000	1.3652250000	-0.5700780000
P	-2.0269070000	-0.4923840000	-3.1748300000
P	-1.0031610000	1.4267790000	-2.7634400000
P	0.1736620000	-0.3112460000	-3.4767870000
Si	5.1519220000	-0.5387660000	-0.6711340000
Si	-5.5900890000	0.9765480000	-0.7087350000
Si	1.9265080000	4.3601640000	1.1214220000
O	-4.1913160000	0.0746100000	-1.1897890000
O	2.7051290000	0.6006960000	-1.8041170000
O	0.6139680000	3.4151900000	-1.2472350000
N	3.4879030000	-1.2672080000	-0.7716740000
N	1.0218550000	2.7908370000	0.8876620000
N	-4.0086430000	-1.0958820000	0.7485860000
C	2.5155250000	-0.5343690000	-1.3986240000
C	0.6935260000	1.9591540000	1.9990740000
C	3.2065070000	-2.5758110000	-0.2668880000
C	-3.5775120000	-0.8124630000	-0.4133370000

C	0.4251820000	2.6315820000	-0.3295030000
C	-2.7479800000	-1.7665930000	2.7068390000
H	-2.6124640000	-0.7180020000	2.9512750000
C	-3.4351480000	-2.1051740000	1.5363190000
C	-3.6175530000	-3.4568820000	1.2182190000
H	-4.1721620000	-3.7187220000	0.3222420000
C	3.6325470000	-3.7037030000	-0.9709220000
H	4.1532890000	-3.5729530000	-1.9150670000
C	-2.4030000000	-4.1065530000	3.2026610000
H	-2.0044770000	-4.8822550000	3.8498400000
C	-5.8704660000	2.0355780000	-2.2241160000
H	-4.9976730000	2.6639550000	-2.4270310000
H	-6.7365590000	2.6923940000	-2.0906840000
H	-6.0491350000	1.4165230000	-3.1085850000
C	-3.1009690000	-4.4466620000	2.0469870000
H	-3.2482460000	-5.4909180000	1.7863430000
C	-2.2294550000	-2.7625700000	3.5255150000
H	-1.6869260000	-2.4842110000	4.4249620000
C	2.5193310000	-2.7306150000	0.9380580000
H	2.1855690000	-1.8499130000	1.4789270000
C	5.7628530000	-0.1788000000	-2.4046860000
H	5.8108410000	-1.0928410000	-3.0051970000
H	6.7691060000	0.2529620000	-2.3718720000
H	5.0985560000	0.5284300000	-2.9068810000
C	1.5933190000	0.9830960000	2.4308450000
H	2.5149090000	0.8400900000	1.8751330000
C	6.2288550000	-1.8274960000	0.1659370000
H	5.8315170000	-2.1187600000	1.1430060000
H	7.2280640000	-1.4056400000	0.3234360000
H	6.3402780000	-2.7357830000	-0.4325050000

C	5.0762510000	1.0082200000	0.3837140000
H	4.2757760000	1.6582630000	0.0228530000
H	6.0211600000	1.5593420000	0.3230570000
H	4.8982490000	0.7686460000	1.4372220000
C	-0.4979620000	2.1591580000	2.7005450000
H	-1.1897860000	2.9245230000	2.3615230000
C	2.2377240000	-4.0057830000	1.4200910000
H	1.6771100000	-4.1178980000	2.3432930000
C	1.3049950000	0.2169250000	3.5561410000
H	2.0125100000	-0.5363220000	3.8912140000
C	-5.1779860000	2.0183950000	0.7889020000
H	-4.9894790000	1.3855420000	1.6591180000
H	-6.0054840000	2.6977940000	1.0213380000
H	-4.2846060000	2.6221240000	0.6005580000
C	2.6630570000	-5.1286720000	0.7169630000
H	2.4436360000	-6.1228350000	1.0944010000
C	3.3643140000	-4.9749310000	-0.4769060000
H	3.6915850000	-5.8482990000	-1.0331250000
C	-7.0177790000	-0.1966800000	-0.4137240000
H	-7.1963190000	-0.8246220000	-1.2922950000
H	-7.9368880000	0.3650560000	-0.2131450000
H	-6.8162860000	-0.8472530000	0.4407090000
C	-0.7844350000	1.3878660000	3.8233820000
H	-1.7096300000	1.5535870000	4.3677730000
C	0.1174380000	0.4188370000	4.2550430000
H	-0.1019490000	-0.1739390000	5.1381570000
C	0.6923150000	5.7625220000	0.9485210000
H	0.2524180000	5.7623020000	-0.0518280000
H	1.1867310000	6.7272470000	1.1065540000
H	-0.1147790000	5.6777920000	1.6834790000

C	2.5849410000	4.2871120000	2.8788950000
H	1.7860640000	4.1936390000	3.6203180000
H	3.1333710000	5.2117910000	3.0914600000
H	3.2761660000	3.4510090000	3.0221400000
C	3.3403750000	4.5229300000	-0.0945100000
H	4.1759860000	3.8673990000	0.1648890000
H	3.7105140000	5.5544490000	-0.0867320000
H	3.0052650000	4.2843120000	-1.1059760000

5.4.5. $(TMS\text{-}PhNCO)_3P_7$ (2) asymmetric isomer B2
Charge = 0 Multiplicity = 1

P	0.5089300000	0.5032710000	-0.3769430000
P	0.5552670000	-1.7015350000	-0.2272250000
P	-1.0018390000	1.0261890000	-1.8982720000
P	2.0108540000	0.6256910000	-1.9914840000
P	-0.9201060000	-0.6867940000	-3.2919370000
P	1.2909730000	-0.9433400000	-3.4431440000
P	0.0865030000	-2.3759620000	-2.2666460000
Si	5.6279910000	-0.9168420000	0.2298480000
O	-3.1600710000	-0.4670910000	-1.0546040000
O	-1.6258550000	-3.1911470000	0.0024530000
O	3.7296710000	-1.4773690000	-1.8787720000
N	-1.3558610000	-1.9058260000	1.8548310000
N	4.2134040000	0.0921290000	-0.3172330000
N	-3.2840360000	1.7278380000	-0.4719660000
C	-0.9882940000	-2.2504770000	0.6861220000
C	3.9453720000	1.3821460000	0.2370790000
C	-0.5975810000	-1.0677350000	2.6766730000
C	-2.6588460000	0.6454740000	-1.0275280000
C	3.4590820000	-0.4174080000	-1.3319730000
C	-2.8970040000	3.9895390000	-1.3329400000

H	-3.5924810000	3.7773930000	-2.1395390000
C	-2.6215080000	2.9941890000	-0.3934690000
C	-1.7195070000	3.2476320000	0.6411550000
H	-1.5063490000	2.4683660000	1.3673530000
C	-1.1535780000	0.1245730000	3.1576260000
H	-2.1528350000	0.3989420000	2.8349590000
C	-1.3550080000	5.4726800000	-0.2188740000
H	-0.8569020000	6.4355410000	-0.1560680000
C	-1.0816660000	4.4826590000	0.7197490000
H	-0.3645760000	4.6651180000	1.5145560000
C	-2.2656670000	5.2245050000	-1.2437050000
H	-2.4779200000	5.9918190000	-1.9822170000
C	0.6722090000	-1.4534150000	3.1310150000
H	1.0883750000	-2.3987530000	2.7956530000
C	3.2821170000	1.4944550000	1.4595760000
H	2.9339670000	0.5977930000	1.9635890000
C	4.3787530000	2.5329710000	-0.4252770000
H	4.8962190000	2.4333650000	-1.3748150000
C	1.3748350000	-0.6481880000	4.0217610000
H	2.3515400000	-0.9689870000	4.3746190000
C	3.0477330000	2.7498160000	2.0111160000
H	2.5187790000	2.8238680000	2.9566640000
C	0.8280240000	0.5501340000	4.4756550000
H	1.3738840000	1.1696850000	5.1811140000
C	-0.4399230000	0.9294260000	4.0383940000
H	-0.8858380000	1.8523110000	4.3996480000
C	4.1381920000	3.7866360000	0.1278430000
H	4.4716000000	4.6779000000	-0.3952590000
C	3.4746370000	3.8968710000	1.3473170000
H	3.2911270000	4.8766890000	1.7787410000

C	6.8286730000	-1.1167170000	-1.1955750000
H	6.3437580000	-1.6170660000	-2.0367880000
H	7.6917700000	-1.7155270000	-0.8844400000
H	7.2018990000	-0.1459050000	-1.5371410000
C	6.4292710000	0.0681610000	1.6126860000
H	6.7534660000	1.0570290000	1.2744820000
H	7.3141350000	-0.4699080000	1.9709530000
H	5.7568150000	0.2113990000	2.4633030000
C	4.9930210000	-2.5588370000	0.8701590000
H	4.3081450000	-2.4138850000	1.7118440000
H	5.8249730000	-3.1826130000	1.2150080000
H	4.4580760000	-3.0954690000	0.0832440000
Si	-4.9662370000	1.5044730000	0.1839070000
C	-6.0593850000	0.7633860000	-1.1450910000
C	-5.5714070000	3.2237640000	0.6335060000
C	-4.8785450000	0.4450110000	1.7274650000
H	-6.5633650000	3.1393920000	1.0920850000
H	-5.6617250000	3.8779130000	-0.2383650000
H	-4.9114530000	3.7162110000	1.3538640000
H	-5.8755780000	0.0913010000	2.0116600000
H	-4.4785490000	1.0182900000	2.5703640000
H	-4.2430300000	-0.4287940000	1.5648280000
H	-5.7023400000	-0.2245310000	-1.4428300000
H	-6.0816480000	1.4010880000	-2.0346650000
H	-7.0869060000	0.6699030000	-0.7764020000
Si	-2.8422590000	-4.2510020000	0.6230750000
C	-3.1382040000	-5.3567580000	-0.8547450000
C	-4.3824380000	-3.2975310000	1.0769710000
C	-2.1249980000	-5.1985000000	2.0704830000
H	-2.2183950000	-5.8672000000	-1.1555970000

H	-3.4900920000	-4.7715230000	-1.7098460000
H	-3.8925510000	-6.1187320000	-0.6319580000
H	-5.2354890000	-3.9767890000	1.1833640000
H	-4.6164260000	-2.5701100000	0.2936570000
H	-4.2471120000	-2.7590990000	2.0179020000
H	-1.8935130000	-4.5259380000	2.9004810000
H	-1.2083070000	-5.7236230000	1.7841710000
H	-2.8414570000	-5.9473380000	2.4259080000

5.4.6. (TMS-PhNCO)$_3$P$_7$ (2) asymmetric isomer B3
Charge = 0 Multiplicity = 1

P	0.5012540000	0.6511260000	-0.1470900000
P	0.2215760000	-1.3801610000	-0.9534960000
P	-0.9923220000	1.9715410000	-1.1114190000
P	1.9199840000	1.3279760000	-1.7001540000
P	-1.1743480000	1.0649200000	-3.1273980000
P	0.9488720000	0.5843420000	-3.5878000000
P	-0.4532440000	-1.0306010000	-3.0127420000
Si	-2.5015310000	-3.8543650000	1.5980870000
Si	-4.9697040000	0.3643980000	-1.3812990000
Si	5.4485850000	-1.2510090000	-0.5912240000
O	-3.2647280000	0.5081200000	-1.1152510000
O	-2.1425770000	-2.6872140000	-1.0307280000
O	3.3128280000	-0.8728630000	-2.5071190000
N	-1.3500490000	-2.5393220000	1.0829030000
N	4.1767610000	0.0502140000	-0.6246950000
N	-3.2856290000	2.0912190000	0.5122530000
C	-1.3165200000	-2.2376000000	-0.2504380000
C	4.1874390000	1.1440510000	0.2963130000
C	-0.4133720000	-1.9900210000	2.0089580000
C	-2.6750290000	1.4788490000	-0.4232630000

C	3.2604240000	-0.0205160000	-1.6327750000
C	-3.3870230000	4.3237750000	1.3933370000
H	-4.3268120000	4.4458610000	0.8636020000
C	-2.7015270000	3.1076560000	1.2733120000
C	-1.5063040000	2.9307800000	1.9819890000
H	-0.9930360000	1.9748140000	1.9416410000
C	-0.7843220000	-0.9023560000	2.8019330000
H	-1.7654240000	-0.4571440000	2.6613940000
C	-1.6598140000	5.1806320000	2.8483000000
H	-1.2550600000	5.9861390000	3.4534510000
C	-5.0365840000	-1.1005300000	-2.5362540000
H	-4.6171450000	-0.8445960000	-3.5141120000
H	-6.0645740000	-1.4464470000	-2.6859250000
H	-4.4429470000	-1.9266170000	-2.1318980000
C	-0.9909380000	3.9650450000	2.7581710000
H	-0.0565670000	3.8139920000	3.2917460000
C	-2.8619830000	5.3527050000	2.1627160000
H	-3.3972590000	6.2954340000	2.2322440000
C	0.8446000000	-2.5732800000	2.1771420000
H	1.1217170000	-3.4215530000	1.5582220000
C	-2.1427510000	-4.1371730000	3.4193720000
H	-1.1117890000	-4.4589060000	3.5936300000
H	-2.8064160000	-4.9248320000	3.7936400000
H	-2.3189770000	-3.2396520000	4.0196640000
C	3.6143770000	1.0054390000	1.5606490000
H	3.1220570000	0.0741150000	1.8230730000
C	-2.1207070000	-5.4027350000	0.6132640000
H	-2.2488070000	-5.2223010000	-0.4563800000
H	-2.7927610000	-6.2150040000	0.9111390000
H	-1.0936700000	-5.7393280000	0.7872420000

C	-4.2756170000	-3.2975320000	1.3846280000
H	-4.4790930000	-2.4070530000	1.9876070000
H	-4.9613770000	-4.0867870000	1.7119790000
H	-4.4858350000	-3.0690660000	0.3373750000
C	4.8097130000	2.3441990000	-0.0552890000
H	5.2509110000	2.4406410000	-1.0429830000
C	1.7222480000	-2.0704640000	3.1337110000
H	2.6953350000	-2.5345460000	3.2679090000
C	3.6560990000	2.0647530000	2.4619000000
H	3.1988020000	1.9525550000	3.4407990000
C	-5.5160830000	1.9553650000	-2.2061110000
H	-5.3853650000	2.8122680000	-1.5391230000
H	-6.5746560000	1.9009190000	-2.4824480000
H	-4.9392480000	2.1379030000	-3.1182690000
C	1.3480140000	-0.9878880000	3.9273980000
H	2.0297260000	-0.6062180000	4.6820990000
C	0.0955170000	-0.4028180000	3.7575470000
H	-0.2022260000	0.4421860000	4.3714930000
C	-5.9089930000	0.0634550000	0.2073660000
H	-5.6010810000	-0.8692240000	0.6865650000
H	-6.9820820000	-0.0079310000	-0.0043690000
H	-5.7433480000	0.8856520000	0.9073630000
C	4.8469460000	3.4007450000	0.8481640000
H	5.3248030000	4.3339390000	0.5656350000
C	4.2714320000	3.2624720000	2.1088760000
H	4.3007670000	4.0885590000	2.8131720000
C	6.4967480000	-1.1316190000	-2.1398000000
H	5.8790840000	-1.2746020000	-3.0294480000
H	7.2791920000	-1.8984280000	-2.1320960000
H	6.9871120000	-0.1554230000	-2.2107150000

C	6.4910970000	-0.8818650000	0.9265810000
H	6.9540730000	0.1081240000	0.8734520000
H	7.2943790000	-1.6235190000	1.0003600000
H	5.9074170000	-0.9264760000	1.8508000000
C	4.6051180000	-2.9150270000	-0.4184620000
H	3.9718780000	-2.9370990000	0.4745250000
H	5.3483180000	-3.7146330000	-0.3269580000
H	3.9754960000	-3.1193000000	-1.2873550000

5.4.7. CF_2O
Charge = 0 Multiplicity = 1

O	-0.0466230000	-0.7269130000	0.7078620000
C	-0.0198320000	0.4570640000	0.6604760000
F	-1.0755860000	1.2641720000	0.6177690000
F	1.0710410000	1.2166780000	0.6398920000

5.4.8. $[CF_3O]^-$
Charge = -1 Multiplicity = 1

O	-0.0024660000	-0.0727380000	0.7919310000
C	-0.0077480000	-1.2197580000	1.2200720000
F	-0.0372870000	-1.3904350000	2.6566080000
F	1.1166540000	-2.0616680000	0.8724600000
F	-1.1111530000	-2.0694010000	0.8259280000

5.4.9. $B(C_6F_5)_3$
Charge = 0 Multiplicity = 1

B	-0.1527200000	0.4557970000	-0.1605200000
C	-0.5860590000	1.3581700000	-1.3766080000
C	-0.1083040000	-1.1107320000	-0.3176840000
C	0.2355380000	1.1209860000	1.2133700000
C	-0.0238350000	-3.9535890000	-0.6022600000

C	0.3991710000	-3.1282370000	-1.6617220000
C	0.3396250000	-1.7339100000	-1.5065250000
C	-0.5130710000	-1.9823330000	0.7209530000
C	-0.4887670000	-3.3807870000	0.5971040000
F	0.0160700000	-5.2789060000	-0.7349120000
F	0.8493990000	-3.6771200000	-2.7944170000
F	0.7672890000	-0.9904480000	-2.5413400000
F	-0.9782470000	-1.4844150000	1.8795460000
F	-0.8990550000	-4.1686170000	1.5960760000
C	0.9379580000	2.3272340000	3.7079460000
C	1.6577230000	1.2114750000	3.2392850000
C	1.2910990000	0.6273090000	2.0161560000
C	-0.4498370000	2.2498640000	1.7217490000
C	-0.1271350000	2.8499490000	2.9495890000
F	1.2651870000	2.8894560000	4.8708630000
F	2.6748620000	0.7255680000	3.9577470000
F	2.0184480000	-0.4257380000	1.6056550000
F	-1.4829100000	2.7773710000	1.0427140000
F	-0.8136720000	3.9032240000	3.4036760000
C	-1.3737740000	2.9974720000	-3.5815760000
C	-2.0361030000	1.7786450000	-3.3397880000
C	-1.6274720000	0.9825950000	-2.2575660000
C	0.0416280000	2.5951620000	-1.6551120000
C	-0.3235410000	3.4096670000	-2.7390890000
F	-1.7413530000	3.7617730000	-4.6093870000
F	-3.0391770000	1.3964250000	-4.1365090000
F	-2.3001260000	-0.1641980000	-2.0603520000
F	1.0588850000	3.0253600000	-0.8892410000
F	0.3092550000	4.5625400000	-2.9788190000

5.4.10. $[FB(C_6F_5)_3]^-$
Charge = -1 Multiplicity = 1

B	-1.0312930000	0.2232650000	0.0282600000
C	-0.9890050000	1.2531340000	-1.2823180000
C	-0.3707440000	-1.2773630000	-0.2797500000
C	-0.2759520000	1.0317420000	1.2858070000
C	0.5070170000	-3.9914620000	-0.6547500000
C	0.4773630000	-3.0981990000	-1.7352940000
C	0.0361790000	-1.7742170000	-1.5287690000
C	-0.3270790000	-2.2166340000	0.7693280000
C	0.1011300000	-3.5465250000	0.6148250000
F	0.9186910000	-5.2624450000	-0.8310130000
F	0.8572570000	-3.5187830000	-2.9597680000
F	0.0221920000	-0.9984240000	-2.6379190000
F	-0.6982780000	-1.8584350000	2.0180080000
F	0.1283960000	-4.3989990000	1.6599830000
C	0.8329230000	2.5318650000	3.4745310000
C	1.5555730000	1.4758250000	2.8979060000
C	0.9954700000	0.7624780000	1.8202290000
C	-0.9522780000	2.1116220000	1.8861300000
C	-0.4358920000	2.8532150000	2.9663140000
F	1.3509140000	3.2296770000	4.5048280000
F	2.7781100000	1.1653470000	3.3773000000
F	1.7704130000	-0.2248360000	1.3162040000
F	-2.1605890000	2.5039580000	1.4302660000
F	-1.1318830000	3.8755200000	3.5062330000
C	-0.8508750000	3.1531150000	-3.4367820000
C	-2.0824890000	2.6064350000	-3.0465420000
C	-2.1310160000	1.6724020000	-1.9899130000
C	0.2195170000	1.8229440000	-1.7215220000
C	0.3193290000	2.7532990000	-2.7702760000

F	-0.7891280000	4.0449120000	-4.4458020000
F	-3.2081690000	2.9781200000	-3.6917710000
F	-3.3572600000	1.1913170000	-1.7056160000
F	1.3867140000	1.4601540000	-1.1373620000
F	1.5113150000	3.2615440000	-3.1462110000
F	-2.3785710000	-0.0295660000	0.4192040000

5.4.11. SbF_5
Charge = 0 Multiplicity = 1

F	-1.9354430000	0.7880830000	0.0118850000
F	0.1865520000	1.0762600000	-1.6358360000
F	-1.0232240000	-1.7224270000	-0.3860040000
F	0.2213570000	0.5881340000	1.6245680000
F	1.5235260000	-0.8318660000	-0.2673920000
Sb	-0.2057670000	-0.0211940000	-0.1292210000

5.4.12. $[SbF_6]^-$
Charge = -1 Multiplicity = 1

F	0.6934610000	1.8491390000	0.2844740000
F	0.2883260000	-0.4182980000	1.7840280000
F	-1.8501630000	0.8765200000	0.6436940000
F	-0.6454980000	0.6963010000	-1.8202990000
F	1.4930270000	-0.5985220000	-0.6799630000
F	-1.0505750000	-1.5711420000	-0.3207950000
Sb	-0.1785690000	0.1390010000	-0.0181390000

5.4.13. $[Me_3Si]^+$
Charge = +1 Multiplicity = 1

Si	-3.5559600000	0.9939850000	-2.5394120000
C	-2.7372460000	2.3076500000	-1.5383540000
C	-5.1520740000	0.2646870000	-1.9754330000

C	-2.7785400000	0.4088800000	-4.1049910000
H	-5.9235740000	0.4059700000	-2.7675820000
H	-5.5198920000	0.6941920000	-1.0232680000
H	-5.0357950000	-0.8382910000	-1.8636070000
H	-3.4374640000	3.1651270000	-1.4085070000
H	-1.7890200000	2.6702570000	-1.9807280000
H	-2.5422780000	1.9214260000	-0.5109800000
H	-1.7518180000	0.0316470000	-3.8900920000
H	-2.6418670000	1.2732550000	-4.7955890000
H	-3.3614710000	-0.3817840000	-4.6164560000

5.4.14. Me_3SiF
Charge = 0 Multiplicity = 1

F	-2.4514360000	-0.5755960000	-1.3668210000
Si	-3.2706670000	0.5824720000	-2.2238730000
C	-2.7366820000	2.2546910000	-1.5424270000
C	-5.1083400000	0.2732700000	-1.9531520000
C	-2.7852850000	0.3766130000	-4.0314760000
H	-5.7247850000	1.0162240000	-2.5030130000
H	-5.3718330000	0.3455110000	-0.8770980000
H	-5.4010510000	-0.7374200000	-2.3071960000
H	-3.2481490000	3.0850840000	-2.0745550000
H	-1.6423760000	2.4030790000	-1.6563780000
H	-2.9807280000	2.3435440000	-0.4630480000
H	-1.6909750000	0.5038270000	-4.1688000000
H	-3.2961870000	1.1270910000	-4.6718380000
H	-3.0595060000	-0.6313930000	-4.4073280000

5.4.15. $[(Me_3Si)_2P_7]^-$
Charge = -1 Multiplicity = 1

P	-0.4059540000	-0.0554550000	-0.0693580000

P	0.4140040000	0.2763240000	-2.1612650000
P	0.4510290000	-1.9305040000	0.6251970000
P	-2.3647230000	-0.9015210000	-0.8051280000
P	0.2024040000	-3.1553780000	-1.1596670000
P	-1.6914680000	-2.4654260000	-2.2560010000
P	0.3558760000	-1.7795870000	-3.0066370000
Si	2.6753490000	0.5032530000	-1.8126800000
Si	-3.3211350000	0.6467400000	-2.2172180000
C	-3.0449240000	2.3851230000	-1.4848780000
C	-5.1972200000	0.2748920000	-2.1695290000
C	-2.7904780000	0.6385210000	-4.0441990000
C	2.9190610000	1.7654290000	-0.4053910000
C	3.3842820000	1.2567560000	-3.4207430000
C	3.6335810000	-1.0848000000	-1.4144180000
H	2.3602560000	2.7040980000	-0.6038040000
H	3.9958020000	2.0189910000	-0.2861550000
H	2.5485400000	1.3451580000	0.5526000000
H	3.1756700000	-1.5752110000	-0.5285970000
H	4.7022010000	-0.8549930000	-1.2044360000
H	3.5855800000	-1.8019590000	-2.2602150000
H	2.8844930000	2.2169480000	-3.6688890000
H	3.2318410000	0.5665500000	-4.2774280000
H	4.4765150000	1.4471310000	-3.3235390000
H	-5.7669940000	1.0054730000	-2.7862820000
H	-5.5864270000	0.3180800000	-1.1304050000
H	-5.4061670000	-0.7426610000	-2.5630510000
H	-3.6145130000	3.1507640000	-2.0572390000
H	-1.9674530000	2.6484240000	-1.5184540000
H	-3.3705500000	2.4284610000	-0.4244240000
H	-1.7016430000	0.8314240000	-4.1331150000

H	-3.3439610000	1.4227280000	-4.6078720000
H	-3.0018760000	-0.3457740000	-4.5117770000

5.4.16. Me_2PhSiF
Charge = 0 Multiplicity = 1

F	-0.4609770000	-3.1888230000	-0.4767790000
Si	-1.6515180000	-3.7075760000	0.5538800000
C	-1.1670470000	-3.1790200000	2.2937290000
C	-1.7680990000	-5.5787340000	0.3916080000
C	-3.2287020000	-2.8500660000	-0.0295790000
H	-2.5886520000	-5.9812660000	1.0229160000
H	-0.8236640000	-6.0695750000	0.7074850000
H	-1.9767340000	-5.8699550000	-0.6586270000
H	-1.9557110000	-3.4542310000	3.0259560000
H	-1.0273290000	-2.0794350000	2.3459110000
H	-0.2214540000	-3.6652360000	2.6132730000
C	-5.5841130000	-1.5446020000	-0.9322670000
C	-5.6378360000	-2.4094710000	0.1758270000
C	-4.4707590000	-3.0541260000	0.6203400000
C	-3.1957440000	-1.9759240000	-1.1428120000
C	-4.3609960000	-1.3290140000	-1.5907460000
H	-6.4980800000	-1.0381980000	-1.2823430000
H	-6.5942820000	-2.5818570000	0.6955370000
H	-4.5370700000	-3.7285880000	1.4921960000
H	-2.2403440000	-1.8020160000	-1.6641100000
H	-4.3138870000	-0.6522860000	-2.4593940000

5.4.17. $(Me_2PhSi)_3P_7$ (10)
Charge = 0 Multiplicity = 1

P	0.3888940000	-0.4941970000	1.2973760000
P	-1.3449870000	0.4424730000	0.2579000000

P	2.0255250000	0.4896990000	0.1495550000
P	0.3947870000	-2.4354590000	0.2081790000
P	1.4791130000	0.0389310000	-1.9772890000
P	0.1589640000	-1.8079730000	-1.9285840000
P	-0.7809580000	0.2532720000	-1.9022480000
Si	-1.1447190000	2.7294400000	0.6312270000
Si	3.9349450000	-0.7884020000	0.4940360000
Si	-1.6027880000	-3.5126040000	0.6963740000
C	-1.6400340000	-3.6500470000	2.5867500000
C	-1.3790800000	-5.2263090000	-0.0891010000
C	-3.1952820000	-2.7233900000	0.0375500000
C	3.9540820000	-1.2216400000	2.3411830000
C	5.3511600000	0.4175510000	0.0968790000
C	4.0792160000	-2.3450700000	-0.5700310000
C	-0.7511670000	2.9239590000	2.4776840000
C	-2.8903040000	3.3953250000	0.2721660000
C	0.1256060000	3.6416660000	-0.4325110000
H	-1.4854580000	2.3844280000	3.1101930000
H	-0.7881150000	4.0000540000	2.7524800000
H	0.2612930000	2.5360710000	2.7140570000
H	1.1398590000	3.2297700000	-0.2554770000
H	0.1345240000	4.7217980000	-0.1695820000
H	-0.1005520000	3.5470680000	-1.5145850000
H	4.0698990000	-2.1060670000	-1.6533670000
H	3.2306510000	-3.0276990000	-0.3599620000
H	5.0256680000	-2.8794700000	-0.3368580000
H	4.9141270000	-1.7209620000	2.5939370000
H	3.1222940000	-1.9098700000	2.5974400000
H	3.8628570000	-0.3159270000	2.9748700000
H	-2.2433350000	-5.8736310000	0.1732820000

H	-0.4508670000	-5.7150750000	0.2727070000
H	-1.3248940000	-5.1666430000	-1.1958010000
H	-2.4925290000	-4.2837340000	2.9129820000
H	-1.7380340000	-2.6568770000	3.0713090000
H	-0.7050850000	-4.1180280000	2.9583720000
C	-5.6207350000	-1.6250280000	-0.9564680000
C	-5.4079260000	-1.7282220000	0.4290600000
C	-4.2059170000	-2.2681160000	0.9187110000
C	-3.4335000000	-2.6180510000	-1.3552240000
C	-4.6297890000	-2.0716600000	-1.8488630000
H	-6.5592480000	-1.1956010000	-1.3422550000
H	-6.1821300000	-1.3840530000	1.1338830000
H	-4.0578450000	-2.3330890000	2.0091170000
H	-2.6689910000	-2.9606190000	-2.0734720000
H	-4.7891160000	-1.9925530000	-2.9362330000
C	-5.4959410000	4.4454020000	-0.1730260000
C	-5.3198080000	3.3126090000	0.6421830000
C	-4.0322020000	2.7938090000	0.8573880000
C	-3.0909250000	4.5308040000	-0.5499230000
C	-4.3784760000	5.0523240000	-0.7709810000
H	-6.5055030000	4.8521290000	-0.3453710000
H	-6.1924980000	2.8274540000	1.1086350000
H	-3.9161730000	1.8933280000	1.4841150000
H	-2.2296180000	5.0234350000	-1.0302860000
H	-4.5087640000	5.9369080000	-1.4153030000
C	7.5009870000	2.2067650000	-0.4193060000
C	6.4584390000	2.5856290000	0.4459770000
C	5.3966020000	1.7008820000	0.6966460000
C	6.4082910000	0.0584980000	-0.7741410000
C	7.4730130000	0.9415830000	-1.0301750000

H	8.3332490000	2.9004470000	-0.6202190000
H	6.4712810000	3.5784830000	0.9241520000
H	4.5797860000	2.0218790000	1.3655970000
H	6.4073180000	-0.9279500000	-1.2664170000
H	8.2838600000	0.6396720000	-1.7127690000

5.4.18. $[(Me_2PhSi)_2P_7]^-$
Charge = -1 Multiplicity = 1

P	-0.2996330000	0.1536970000	-0.3345820000
P	0.5068920000	0.3632770000	-2.4478030000
P	0.6522890000	-1.6095560000	0.5155440000
P	-2.1873220000	-0.8668520000	-1.0111420000
P	0.5054060000	-2.9814160000	-1.1702340000
P	-1.4159340000	-2.5004160000	-2.3304620000
P	0.5929270000	-1.7540200000	-3.1198800000
Si	2.7377350000	0.7566300000	-2.0567520000
Si	-3.2544960000	0.5185290000	-2.5051910000
C	-3.3430820000	2.2424230000	-1.6998770000
C	-5.0297610000	-0.1795350000	-2.6293610000
C	-2.6241020000	0.6969260000	-4.3005510000
C	2.8784700000	2.0243160000	-0.6407820000
C	3.4538820000	1.5891650000	-3.6353920000
C	3.8069130000	-0.7569370000	-1.6496460000
H	2.2495050000	2.9169710000	-0.8375030000
H	3.9318520000	2.3597530000	-0.5213320000
H	2.5402230000	1.5638460000	0.3108080000
H	3.3801010000	-1.2596920000	-0.7551350000
H	4.8574540000	-0.4595680000	-1.4371200000
H	3.8057610000	-1.4826030000	-2.4887740000
H	-5.6737930000	0.4802840000	-3.2507720000
H	-5.4828050000	-0.2728430000	-1.6198570000

H	-5.0242650000	-1.1893830000	-3.0905460000
H	-4.0197980000	2.9225900000	-2.2618080000
H	-2.3363030000	2.7052670000	-1.6427530000
H	-3.7273300000	2.1523800000	-0.6623870000
C	-1.8163690000	0.9693910000	-7.0197770000
C	-1.9281200000	2.1041860000	-6.1970590000
C	-2.3243790000	1.9650490000	-4.8547180000
C	-2.5007800000	-0.4325870000	-5.1470950000
C	-2.1013360000	-0.3010480000	-6.4881650000
H	-1.4989460000	1.0736220000	-8.0706910000
H	-1.6990920000	3.1047570000	-6.6009040000
H	-2.3935110000	2.8677280000	-4.2256660000
H	-2.6968580000	-1.4404860000	-4.7414840000
H	-2.0017760000	-1.1991640000	-7.1199410000
C	4.5808600000	2.8471820000	-5.9337570000
C	3.2028680000	2.5745290000	-5.8769120000
C	2.6495580000	1.9539550000	-4.7414550000
C	4.8411350000	1.8701640000	-3.7179050000
C	5.4016120000	2.4920050000	-4.8477270000
H	5.0163380000	3.3324540000	-6.8233330000
H	2.5508160000	2.8434230000	-6.7246880000
H	1.5666370000	1.7413110000	-4.7096840000
H	5.5066680000	1.5952480000	-2.8805110000
H	6.4848880000	2.6980480000	-4.8832370000

5.4.19. $MePh_2SiF$
Charge = 0 Multiplicity = 1

F	2.1980190000	-0.1466170000	0.7710440000
Si	3.3056730000	-1.1412560000	1.5032550000
C	3.1236380000	-0.8752830000	3.3565320000
C	4.9836790000	-0.5700980000	0.8574480000

C	2.9404340000	-2.9216180000	0.9970620000
H	3.8545410000	-1.4883800000	3.9242160000
H	2.1047770000	-1.1359870000	3.7117080000
H	3.3073070000	0.1908030000	3.6036470000
C	2.3492340000	-5.5778250000	0.1823770000
C	1.7990380000	-5.0663810000	1.3710290000
C	2.0936200000	-3.7518570000	1.7719670000
C	3.4877310000	-3.4585950000	-0.1945720000
C	3.1945490000	-4.7717030000	-0.6006230000
H	2.1198540000	-6.6083210000	-0.1336280000
H	1.1371960000	-5.6949320000	1.9884900000
H	1.6505010000	-3.3687540000	2.7063750000
H	4.1570400000	-2.8394350000	-0.8150560000
H	3.6292920000	-5.1695650000	-1.5317980000
C	7.5050010000	0.3492520000	-0.0697260000
C	6.3326730000	1.0501170000	-0.4019910000
C	5.0844620000	0.5942710000	0.0579120000
C	6.1760030000	-1.2655170000	1.1776790000
C	7.4252700000	-0.8111310000	0.7216210000
H	8.4836870000	0.7059000000	-0.4293840000
H	6.3902460000	1.9575720000	-1.0247750000
H	4.1698710000	1.1478270000	-0.2101800000
H	6.1335100000	-2.1835140000	1.7892770000
H	8.3411540000	-1.3659730000	0.9820940000

5.4.20. $(MePh_2Si)_3P_7$ (11)
Charge = 0 Multiplicity = 1

P	-0.2815460000	0.1374450000	0.9055930000
P	-0.6079700000	-1.9849810000	0.2988880000
P	-1.3900710000	1.0875150000	-0.7734680000
P	1.7720160000	0.3699560000	0.0826000000

P	-0.4307260000	0.1879150000	-2.5882340000
P	1.6187960000	-0.5547250000	-1.9484340000
P	-0.1665150000	-1.9535550000	-1.8940080000
Si	-2.9036720000	-2.4284160000	0.3303540000
Si	-0.7702500000	3.3139850000	-0.9613310000
Si	3.1119850000	-1.1014590000	1.2733330000
C	2.9240720000	-0.5661480000	3.0841770000
C	4.8729020000	-0.7170730000	0.6599490000
C	2.7742110000	-2.9555580000	1.0754400000
C	-1.4784510000	4.2224320000	0.5504080000
C	-1.6832860000	3.8818010000	-2.5281990000
C	1.0825430000	3.6767880000	-1.1363150000
C	-3.7483360000	-1.3258810000	1.6282910000
C	-2.9292720000	-4.2232520000	0.9516640000
C	-3.7672250000	-2.3375260000	-1.3580810000
H	-2.4699730000	-4.2760200000	1.9600690000
H	-2.3521760000	-4.8985580000	0.2860830000
H	-3.9715160000	-4.5998260000	1.0262850000
H	-1.5401470000	4.9741330000	-2.6699810000
H	-2.7708890000	3.6841750000	-2.4349590000
H	-1.3149440000	3.3629160000	-3.4373020000
H	3.6354050000	-1.1295250000	3.7247660000
H	1.8947430000	-0.7317260000	3.4642860000
H	3.1574220000	0.5135820000	3.1849090000
C	-5.0682200000	0.1634260000	3.6592600000
C	-5.6853060000	-0.0324250000	2.4116760000
C	-5.0318160000	-0.7700600000	1.4079160000
C	-3.1430770000	-1.1162780000	2.8930110000
C	-3.7933630000	-0.3813700000	3.8983090000
H	-5.5810660000	0.7387390000	4.4469260000

H	-6.6843720000	0.3900300000	2.2169470000
H	-5.5340490000	-0.9179790000	0.4380160000
H	-2.1425580000	-1.5315010000	3.1019980000
H	-3.3021720000	-0.2324040000	4.8734660000
C	-5.0677800000	-2.2719420000	-3.8472540000
C	-4.7486400000	-3.4893020000	-3.2610970000
C	-4.1094530000	-3.5218540000	-2.0328600000
C	-4.0967770000	-1.1177980000	-1.9731340000
C	-4.7408130000	-1.0868380000	-3.1981940000
H	-5.5760290000	-2.2454310000	-4.8237010000
H	-5.0030890000	-4.4319830000	-3.7701590000
H	-3.8704840000	-4.5003570000	-1.5884910000
H	-3.8265250000	-0.1683450000	-1.4830740000
H	-4.9883700000	-0.1183660000	-3.6600330000
C	-2.5808180000	5.7013600000	2.7142830000
C	-2.8198820000	4.3207330000	2.6067380000
C	-2.2744420000	3.5907930000	1.5353580000
C	-1.2482550000	5.6165690000	0.6778070000
C	-1.7925820000	6.3493810000	1.7461380000
H	-3.0077930000	6.2742340000	3.5532680000
H	-3.4352110000	3.8036470000	3.3601940000
H	-2.4780970000	2.5088810000	1.4708150000
H	-0.6283840000	6.1436940000	-0.0677080000
H	-1.5999340000	7.4317310000	1.8238610000
C	3.8572220000	4.2129740000	-1.4201290000
C	3.2244570000	4.4033380000	-0.1785200000
C	1.8524540000	4.1333680000	-0.0389890000
C	1.7377730000	3.4934350000	-2.3791580000
C	3.1113120000	3.7543260000	-2.5207080000
H	4.9325310000	4.4264320000	-1.5323020000

H	3.8035200000	4.7642330000	0.6869780000
H	1.3718150000	4.2818800000	0.9418210000
H	1.1720930000	3.1335110000	-3.2548020000
H	3.6013570000	3.6012890000	-3.4956370000
C	2.2897190000	-5.7384740000	0.7729420000
C	2.0927790000	-5.1193640000	2.0195250000
C	2.3310600000	-3.7417340000	2.1662650000
C	2.9748390000	-3.6002160000	-0.1706780000
C	2.7330140000	-4.9756980000	-0.3228800000
H	2.1005350000	-6.8177050000	0.6550640000
H	1.7497310000	-5.7123390000	2.8828670000
H	2.1648990000	-3.2749800000	3.1509430000
H	3.3262890000	-3.0186920000	-1.0398580000
H	2.8911810000	-5.4552150000	-1.3022700000
C	7.5503390000	-0.1483190000	-0.1076010000
C	6.5693810000	0.8573550000	-0.1639900000
C	5.2455620000	0.5756710000	0.2155350000
C	5.8748950000	-1.7185500000	0.7056230000
C	7.1999740000	-1.4380250000	0.3285800000
H	8.5876160000	0.0718970000	-0.4076670000
H	6.8348450000	1.8690960000	-0.5108550000
H	4.4882590000	1.3762010000	0.1559080000
H	5.6163490000	-2.7379650000	1.0375370000
H	7.9622460000	-2.2328580000	0.3721030000

5.4.21. $[(MePh_2Si)_2P_7]^-$
Charge = -1 Multiplicity = 1

P	-0.4655370000	-1.3662520000	1.6629490000
P	0.7572120000	-3.1686090000	1.7563310000
P	-2.0234300000	-2.2198290000	0.2919240000
P	0.4485660000	-0.0358020000	0.0625580000

P	-0.8112120000	-2.8290820000	-1.4973140000
P	1.0651130000	-1.5249710000	-1.4652060000
P	1.0511650000	-3.5470670000	-0.3701370000
Si	-3.3562700000	-0.5940260000	-0.6416480000
Si	2.4746530000	0.5603180000	0.9916400000
C	2.4900640000	0.2567110000	2.8698230000
C	2.6667140000	2.4517640000	0.7234310000
C	3.9979800000	-0.2922440000	0.2189700000
C	-4.3776420000	0.2126070000	0.7647470000
C	-4.5568660000	-1.5661520000	-1.7655990000
C	-2.6147190000	0.8050960000	-1.7083270000
H	-5.3225880000	-0.8999760000	-2.2186540000
H	-5.0709250000	-2.3644590000	-1.1910250000
H	-3.9935230000	-2.0491690000	-2.5910120000
H	3.4207980000	0.6635840000	3.3206540000
H	2.4279160000	-0.8309230000	3.0806350000
H	1.6189850000	0.7497730000	3.3488290000
C	-5.9155220000	1.4460690000	2.8227400000
C	-4.6161110000	0.9805780000	3.0898890000
C	-3.8585410000	0.3712960000	2.0731220000
C	-5.6896300000	0.6892120000	0.5183990000
C	-6.4518670000	1.2992870000	1.5309340000
H	-6.5110780000	1.9215060000	3.6198870000
H	-4.1860640000	1.0885780000	4.0993590000
H	-2.8418620000	0.0053330000	2.3026150000
H	-6.1308820000	0.5839660000	-0.4872470000
H	-7.4708650000	1.6596910000	1.3108950000
C	-1.5887900000	2.8721510000	-3.3736700000
C	-2.3054910000	3.1929620000	-2.2072310000
C	-2.8100790000	2.1693320000	-1.3848100000

C	-1.8866060000	0.5031440000	-2.8863370000
C	-1.3793160000	1.5221490000	-3.7095420000
H	-1.1872040000	3.6727000000	-4.0169560000
H	-2.4714210000	4.2483860000	-1.9330300000
H	-3.3663430000	2.4381980000	-0.4715190000
H	-1.6912440000	-0.5499820000	-3.1527550000
H	-0.8053110000	1.2596370000	-4.6133400000
C	6.2730080000	-1.6091080000	-0.8672570000
C	5.3573830000	-2.3181760000	-0.0685050000
C	4.2316680000	-1.6686750000	0.4661170000
C	4.9245490000	0.4023570000	-0.5959520000
C	6.0513780000	-0.2464820000	-1.1332010000
H	7.1539460000	-2.1208230000	-1.2898430000
H	5.5122690000	-3.3912700000	0.1317110000
H	3.5043180000	-2.2461050000	1.0665840000
H	4.7638300000	1.4709050000	-0.8158690000
H	6.7582600000	0.3158810000	-1.7663660000
C	2.9868700000	5.2659610000	0.4045710000
C	1.8841020000	4.6077250000	-0.1665910000
C	1.7280000000	3.2179300000	-0.0077970000
C	3.7712400000	3.1373260000	1.2913750000
C	3.9335850000	4.5252180000	1.1359220000
H	3.1100070000	6.3550170000	0.2815140000
H	1.1355760000	5.1782390000	-0.7410290000
H	0.8549650000	2.7118900000	-0.4556030000
H	4.5279830000	2.5751190000	1.8658850000
H	4.8027710000	5.0321860000	1.5878760000

5.4.22. Ph_3SiF

Charge = 0 Multiplicity = 1

Si	3.0389320000	0.5830340000	-1.8089480000

C	3.2209970000	1.8224920000	-0.4017120000
C	3.3158140000	1.3989980000	-3.4877720000
C	4.1025200000	-0.9500740000	-1.5320970000
C	3.7320870000	2.5633560000	-6.0425220000
C	2.5551070000	1.8410010000	-5.7797190000
C	2.3497510000	1.2639480000	-4.5141830000
C	4.4941230000	2.1330380000	-3.7702820000
C	4.7027030000	2.7092120000	-5.0348890000
H	3.8938410000	3.0154750000	-7.0343710000
H	1.7908190000	1.7266340000	-6.5654160000
H	1.4230200000	0.7016300000	-4.3158360000
H	5.2642620000	2.2649660000	-2.9910510000
H	5.6258680000	3.2766480000	-5.2352970000
C	5.6924060000	-3.2793820000	-1.2108710000
C	6.2740920000	-2.0054490000	-1.0795140000
C	5.4849100000	-0.8533370000	-1.2372550000
C	3.5341520000	-2.2411230000	-1.6570530000
C	4.3209700000	-3.3951570000	-1.4977610000
H	6.3097230000	-4.1836910000	-1.0865870000
H	7.3477290000	-1.9094740000	-0.8504390000
H	5.9578750000	0.1369310000	-1.1216020000
H	2.4592700000	-2.3414120000	-1.8782870000
H	3.8602250000	-4.3913230000	-1.5972270000
C	3.3972350000	3.6828970000	1.7337290000
C	3.6635430000	2.3210620000	1.9623010000
C	3.5765780000	1.4007630000	0.9035580000
C	2.9592410000	3.1991940000	-0.6120280000
C	3.0442620000	4.1214760000	0.4449420000
H	3.4664910000	4.4055170000	2.5628880000
H	3.9412870000	1.9746020000	2.9708290000

H	3.7892800000	0.3358190000	1.0955920000
H	2.6851600000	3.5560180000	-1.6188880000
H	2.8357990000	5.1880790000	0.2625920000
F	1.4769270000	0.0236300000	-1.8558250000

5.4.23. $(Ph_3Si)_3P_7$ (12)

Charge = 0 Multiplicity = 1

P	-0.0389000000	-0.0206620000	0.7427600000
P	-1.4589140000	-1.3072750000	-0.3884400000
P	-0.3892190000	1.8809030000	-0.3684230000
P	1.8212160000	-0.6167230000	-0.3248180000
P	-0.0801360000	1.2820920000	-2.5078540000
P	1.2064020000	-0.5832770000	-2.4816740000
P	-1.0562230000	-0.7595580000	-2.5216390000
Si	-3.5954930000	-0.4574610000	0.0298650000
Si	1.4046350000	3.3025190000	0.0758780000
Si	2.1996390000	-2.8723950000	0.0879270000
C	2.3840760000	-3.0133680000	1.9758210000
C	3.8729600000	-3.1944630000	-0.7580680000
C	0.9183020000	-4.1081890000	-0.5699180000
C	1.5643520000	3.3898280000	1.9679910000
C	0.7841330000	4.9662260000	-0.6085950000
C	3.0734980000	2.8763670000	-0.7223820000
C	-3.5960300000	0.7534590000	1.4938630000
C	-4.6033320000	-1.9926160000	0.5358810000
C	-4.3738070000	0.3157060000	-1.5216930000
C	-6.2251000000	-4.2164500000	1.2431060000
C	-5.0622410000	-4.4050500000	0.4762050000
C	-4.2590600000	-3.3039270000	0.1287780000
C	-5.7777480000	-1.8232110000	1.3122040000
C	-6.5816940000	-2.9218680000	1.6616980000

H	-6.8525770000	-5.0793860000	1.5191420000
H	-4.7730580000	-5.4169750000	0.1496730000
H	-3.3399630000	-3.4733190000	-0.4576840000
H	-6.0674200000	-0.8157960000	1.6555800000
H	-7.4889620000	-2.7672820000	2.2680120000
C	-5.5744170000	1.4527150000	-3.8322290000
C	-6.0692420000	0.2503020000	-3.2986830000
C	-5.4748740000	-0.3115920000	-2.1548130000
C	-3.8856890000	1.5256710000	-2.0777060000
C	-4.4809370000	2.0898500000	-3.2185820000
H	-6.0390060000	1.8930710000	-4.7292480000
H	-6.9240990000	-0.2557450000	-3.7758230000
H	-5.8743770000	-1.2540180000	-1.7462740000
H	-3.0233420000	2.0343900000	-1.6146290000
H	-4.0836670000	3.0299720000	-3.6338780000
C	-3.6759120000	2.4823170000	3.7472330000
C	-4.1926870000	2.9076390000	2.5115850000
C	-4.1519150000	2.0515150000	1.3961360000
C	-3.0886130000	0.3393850000	2.7514590000
C	-3.1232950000	1.1943150000	3.8651510000
H	-3.7041860000	3.1539700000	4.6202760000
H	-4.6331640000	3.9131580000	2.4137620000
H	-4.5662780000	2.3993850000	0.4362980000
H	-2.6632080000	-0.6714680000	2.8682990000
H	-2.7178140000	0.8537230000	4.8313770000
C	-0.9395000000	-5.9985430000	-1.5939510000
C	-0.6809200000	-5.9382490000	-0.2136560000
C	0.2386860000	-5.0019890000	0.2920150000
C	0.6521560000	-4.1918240000	-1.9602370000
C	-0.2683400000	-5.1233870000	-2.4677640000

H	-1.6598510000	-6.7316330000	-1.9916860000
H	-1.1957780000	-6.6263210000	0.4762250000
H	0.4318990000	-4.9723260000	1.3764170000
H	1.1726520000	-3.5186950000	-2.6627820000
H	-0.4623950000	-5.1668220000	-3.5514890000
C	2.6743320000	-3.2475250000	4.7934130000
C	1.4276160000	-2.9065890000	4.2380380000
C	1.2863140000	-2.7890520000	2.8447740000
C	3.6308180000	-3.3543330000	2.5547370000
C	3.7755610000	-3.4703990000	3.9489570000
H	2.7865820000	-3.3388400000	5.8858960000
H	0.5601310000	-2.7288020000	4.8939050000
H	0.3028880000	-2.5138050000	2.4280390000
H	4.5030650000	-3.5332320000	1.9050380000
H	4.7552230000	-3.7373760000	4.3773340000
C	6.3994680000	-3.7386330000	-1.9405000000
C	6.1405240000	-2.4769150000	-1.3762910000
C	4.8905730000	-2.2078900000	-0.7922890000
C	4.1524440000	-4.4588550000	-1.3339730000
C	5.4024460000	-4.7294800000	-1.9181900000
H	7.3783510000	-3.9488600000	-2.4010060000
H	6.9147500000	-1.6931030000	-1.3952690000
H	4.7022240000	-1.2083400000	-0.3652440000
H	3.3795430000	-5.2448620000	-1.3299260000
H	5.5973190000	-5.7197850000	-2.3607720000
C	5.5860030000	2.3389610000	-1.9364860000
C	5.4924040000	2.4867060000	-0.5416820000
C	4.2484630000	2.7521490000	0.0583500000
C	3.1906770000	2.7345550000	-2.1279870000
C	4.4311740000	2.4653760000	-2.7296550000

H	6.5598480000	2.1295190000	-2.4078310000
H	6.3941540000	2.3978080000	0.0857300000
H	4.1944980000	2.8683450000	1.1527220000
H	2.2976420000	2.8356080000	-2.7681080000
H	4.4963100000	2.3536940000	-3.8238160000
C	1.8405270000	3.5466850000	4.7919090000
C	2.1331290000	2.3462620000	4.1196490000
C	1.9952270000	2.2695770000	2.7234380000
C	1.2741670000	4.5888550000	2.6634390000
C	1.4110410000	4.6675690000	4.0610550000
H	1.9468780000	3.6073430000	5.8871530000
H	2.4680130000	1.4613920000	4.6843110000
H	2.2277700000	1.3180930000	2.2163350000
H	0.9375400000	5.4773410000	2.1047070000
H	1.1804700000	5.6114900000	4.5811050000
C	-0.1197460000	7.4914980000	-1.5481370000
C	-1.0075050000	6.6266760000	-0.8830250000
C	-0.5600460000	5.3770890000	-0.4209840000
C	1.6626570000	5.8515480000	-1.2802510000
C	1.2161430000	7.1013090000	-1.7456820000
H	-0.4706420000	8.4697360000	-1.9146430000
H	-2.0566780000	6.9256820000	-0.7267200000
H	-1.2704090000	4.7056830000	0.0905660000
H	2.7128520000	5.5598230000	-1.4447400000
H	1.9166000000	7.7735060000	-2.2672550000

5.4.24. $[(Ph_3Si)_2P_7]^-$
Charge = -1 Multiplicity = 1

P	-0.0438370000	-0.7893460000	-1.8534490000
P	-0.6883660000	-0.1397050000	0.2267900000
P	-0.8862140000	-2.7646360000	-2.2037860000

P	1.9473540000	-1.5372560000	-1.1251660000
P	-0.5358390000	-3.6950450000	-0.2622500000
P	1.3905080000	-2.8343250000	0.6212700000
P	-0.6303880000	-2.0516330000	1.3498100000
Si	-2.9464500000	0.2899760000	0.0567030000
Si	3.1575900000	0.1035060000	-0.0568440000
C	3.5139740000	1.5119970000	-1.3056350000
C	4.8424610000	-0.7201310000	0.3441770000
C	2.5112250000	0.8905710000	1.5556050000
C	-3.3129580000	0.9501340000	-1.6966260000
C	-3.2877190000	1.7358160000	1.2686700000
C	-4.1580600000	-1.1185330000	0.4938000000
C	-3.8653240000	3.8367730000	3.1014220000
C	-2.7355040000	3.0216960000	3.2904760000
C	-2.4508570000	1.9856900000	2.3822340000
C	-4.4191210000	2.5723600000	1.0937110000
C	-4.7077700000	3.6104190000	1.9977670000
H	-4.0874180000	4.6519450000	3.8102060000
H	-2.0636620000	3.1953610000	4.1470350000
H	-1.5492400000	1.3669840000	2.5309140000
H	-5.0855680000	2.4134500000	0.2287990000
H	-5.5929910000	4.2486500000	1.8379390000
C	-5.9969890000	-3.1989530000	1.1121690000
C	-6.0804790000	-1.9616530000	1.7749540000
C	-5.1698860000	-0.9343960000	1.4686640000
C	-4.0822730000	-2.3760520000	-0.1560470000
C	-4.9945540000	-3.4019620000	0.1471580000
H	-6.7075720000	-4.0069370000	1.3544650000
H	-6.8574970000	-1.7945720000	2.5397980000
H	-5.2475490000	0.0277900000	2.0009240000

H	-3.2830960000	-2.5633080000	-0.8996260000
H	-4.9081010000	-4.3726690000	-0.3684030000
C	-3.7937960000	1.9806850000	-4.3045700000
C	-4.2678320000	0.7063960000	-3.9473310000
C	-4.0285460000	0.1975930000	-2.6582980000
C	-2.8415760000	2.2320730000	-2.0767850000
C	-3.0777810000	2.7433430000	-3.3643730000
H	-3.9791400000	2.3784040000	-5.3161600000
H	-4.8253800000	0.0989890000	-4.6793350000
H	-4.4040850000	-0.8047580000	-2.3976110000
H	-2.2803300000	2.8448680000	-1.3513430000
H	-2.6988350000	3.7426650000	-3.6359650000
C	1.5726030000	2.0510990000	3.9769040000
C	1.7957840000	2.8566630000	2.8462060000
C	2.2595910000	2.2804170000	1.6502670000
C	2.2781550000	0.0951020000	2.7058050000
C	1.8143980000	0.6670020000	3.9025580000
H	1.2072300000	2.5004500000	4.9153290000
H	1.6025870000	3.9412280000	2.8932810000
H	2.4232810000	2.9241870000	0.7705010000
H	2.4479660000	-0.9941980000	2.6596810000
H	1.6321380000	0.0251580000	4.7800460000
C	4.0688440000	3.6629910000	-3.0864700000
C	2.8187720000	3.0200640000	-3.1186430000
C	2.5457880000	1.9565880000	-2.2391950000
C	4.7691980000	2.1700240000	-1.2930160000
C	5.0455870000	3.2340730000	-2.1700380000
H	4.2839080000	4.4947310000	-3.7779560000
H	2.0478610000	3.3428440000	-3.8376480000
H	1.5639220000	1.4536550000	-2.2904630000

H	5.5493230000	1.8400670000	-0.5864440000
H	6.0312760000	3.7280960000	-2.1404190000
C	7.3836200000	-1.8964700000	0.8682470000
C	6.6701700000	-2.2385330000	-0.2951900000
C	5.4167130000	-1.6572310000	-0.5513920000
C	5.5768720000	-0.3908530000	1.5101040000
C	6.8327240000	-0.9702590000	1.7707630000
H	8.3653860000	-2.3549580000	1.0729990000
H	7.0906300000	-2.9689820000	-1.0062770000
H	4.8598610000	-1.9457270000	-1.4591720000
H	5.1561610000	0.3283280000	2.2324200000
H	7.3819620000	-0.6985370000	2.6878350000

5.4.25. (TMS-BrPhNCO)$_3$P$_7$ (3)
Charge = 0 Multiplicity = 1

P	-0.7083810000	-0.5091800000	-0.6651140000
P	-0.3372890000	-0.8062360000	-2.8256740000
P	-1.3193580000	-2.5917760000	-0.2343460000
P	-2.7419470000	0.3413510000	-0.8476610000
P	-2.9728090000	-2.9539140000	-1.6925420000
P	-3.7254310000	-0.9738780000	-2.3564970000
P	-2.1163990000	-1.9857610000	-3.4962370000
Si	3.2239650000	-3.5275550000	-2.2542810000
Si	-4.7914040000	-2.0079870000	2.3896580000
Si	-1.4878690000	4.2703310000	-2.6798800000
O	-3.6037810000	-2.3802230000	1.1861310000
O	0.5205820000	-3.3535930000	-3.2130500000
O	-2.8417830000	1.7740580000	-3.1561940000
N	2.1168000000	-2.0760740000	-2.2533110000
N	-1.7322610000	2.8782120000	-1.5268140000
N	-1.7798450000	-1.9691370000	2.4613490000

C	0.8660190000	-2.2783310000	-2.7616060000
C	-1.3398770000	2.9433140000	-0.1588140000
C	2.5582890000	-0.7816800000	-1.8575700000
C	-2.2942630000	-2.2598980000	1.3372130000
C	-2.4093820000	1.8009410000	-2.0199950000
C	0.1724390000	-0.7008570000	3.0836090000
H	-0.4628050000	0.1676340000	3.2140470000
C	-0.3979710000	-1.9028690000	2.6583230000
C	0.4220140000	-3.0277170000	2.5221260000
H	-0.0164820000	-3.9749830000	2.2289280000
C	2.7877590000	-0.4979990000	-0.5130770000
H	2.5822390000	-1.2517410000	0.2390670000
C	2.3356580000	-1.7295580000	3.1567810000
Br	4.2040570000	-1.6017980000	3.4672370000
C	-6.3661300000	-2.2305530000	1.4182170000
H	-6.4050580000	-1.5368920000	0.5734100000
H	-7.2425760000	-2.0464870000	2.0475340000
H	-6.4434940000	-3.2466470000	1.0212320000
C	1.7860220000	-2.9441350000	2.7677360000
H	2.4146170000	-3.8209670000	2.6644290000
C	1.5343880000	-0.6074370000	3.3261790000
H	1.9642220000	0.3314620000	3.6554030000
C	2.8042660000	0.2014240000	-2.8156240000
H	2.6346280000	-0.0210330000	-3.8633840000
C	2.4537940000	-4.8814990000	-1.2247670000
H	2.2053520000	-4.5158980000	-0.2240300000
H	3.1539090000	-5.7166560000	-1.1177900000
H	1.5380940000	-5.2498700000	-1.6902320000
C	0.0057140000	2.8596230000	0.1897990000
H	0.7504610000	2.6990510000	-0.5815350000

C	4.8276530000	-2.9423360000	-1.4849770000
H	5.2430030000	-2.0746820000	-2.0052670000
H	5.5608550000	-3.7531860000	-1.5571930000
H	4.7249540000	-2.6842450000	-0.4278040000
C	3.5255970000	-4.0465220000	-4.0245360000
H	2.5880470000	-4.3213440000	-4.5113810000
H	4.1975390000	-4.9106850000	-4.0537760000
H	3.9943120000	-3.2409300000	-4.5977690000
C	-2.2944860000	3.1305770000	0.8401360000
H	-3.3416110000	3.2049530000	0.5679620000
C	3.2625980000	1.4566360000	-2.4386220000
H	3.4520610000	2.2193300000	-3.1843800000
C	0.3992140000	2.9676650000	1.5158070000
H	1.4483270000	2.9127900000	1.7796870000
C	-4.5645330000	-0.2390220000	2.9367400000
H	-3.6883120000	-0.1355750000	3.5796720000
H	-5.4469630000	0.1036050000	3.4871470000
H	-4.4292340000	0.4095730000	2.0657780000
C	3.4930330000	1.7155720000	-1.0935020000
Br	4.1260780000	3.4242410000	-0.5688550000
C	3.2650040000	0.7461540000	-0.1258120000
H	3.4537650000	0.9542950000	0.9204240000
C	-4.6553640000	-3.2306570000	3.7909110000
H	-4.7617070000	-4.2576970000	3.4299210000
H	-5.4465120000	-3.0508000000	4.5264160000
H	-3.6905770000	-3.1377460000	4.2945510000
C	-1.9151210000	3.2223620000	2.1721040000
H	-2.6588250000	3.3619630000	2.9471430000
C	-0.5671120000	3.1437940000	2.4968390000
Br	-0.0374330000	3.2732930000	4.3122100000

C	-3.1610090000	4.9430640000	-3.1708500000
H	-3.7510510000	4.1804010000	-3.6822620000
H	-3.0392120000	5.7973700000	-3.8449870000
H	-3.7191520000	5.2873410000	-2.2949090000
C	-0.5435140000	5.5654520000	-1.7123390000
H	-1.0593920000	5.8514100000	-0.7913520000
H	-0.4508270000	6.4636960000	-2.3325710000
H	0.4661760000	5.2424400000	-1.4461270000
C	-0.4855520000	3.6820040000	-4.1415860000
H	0.4678990000	3.2560830000	-3.8150720000
H	-0.2697250000	4.5211980000	-4.8111030000
H	-1.0268910000	2.9186220000	-4.7031740000

5.4.26. (TMS-FPhNCO)$_3$P$_7$ (4)
Charge = 0 Multiplicity = 1

P	-0.0725230000	-0.0234480000	-0.0915740000
P	0.9298150000	1.4453720000	-1.4091600000
P	-0.5941050000	-1.4682300000	-1.6854470000
P	-2.0418700000	0.9914530000	-0.0103150000
P	-1.6802300000	-0.2196790000	-3.1921770000
P	-2.4115970000	1.5883170000	-2.1314440000
P	-0.4469930000	1.6222800000	-3.1608270000
Si	4.5071500000	-1.0726120000	-2.5312690000
Si	-4.7385100000	-2.7345950000	-1.0326880000
O	-3.1921120000	-2.1344010000	-1.5268730000
O	2.1270780000	0.2161260000	-3.5083120000
O	-1.6293990000	3.6458150000	-0.1643540000
N	3.2677910000	-0.1254520000	-1.5892660000
N	-1.0609170000	2.8429690000	1.8724920000
N	-2.0085410000	-3.0515440000	0.1686280000
C	2.2227860000	0.3745240000	-2.3055570000

C	-0.9654120000	1.8163910000	2.8199830000
C	3.4005550000	0.1314070000	-0.1930920000
C	-2.0563790000	-2.3226320000	-0.8678670000
C	-1.4923970000	2.6462510000	0.6964640000
C	-0.6818850000	-2.8970080000	2.1748900000
H	-1.4887290000	-2.3357550000	2.6314620000
C	-0.8138940000	-3.3163200000	0.8487950000
C	0.2164900000	-4.0601880000	0.2663740000
H	0.1049640000	-4.4216980000	-0.7498590000
C	3.1697450000	-0.8885300000	0.7282180000
H	2.8443760000	-1.8617300000	0.3776210000
C	1.4842890000	-3.8843620000	2.2824600000
F	2.6172680000	-4.1283850000	2.9685730000
C	-5.8419670000	-1.9977980000	-2.3418170000
H	-5.7879630000	-0.9054240000	-2.3288050000
H	-6.8853500000	-2.2865030000	-2.1803960000
H	-5.5487720000	-2.3362220000	-3.3395550000
C	1.3728650000	-4.3443460000	0.9828820000
H	2.1789190000	-4.9246490000	0.5479410000
C	0.4708780000	-3.1688910000	2.8946540000
H	0.5819910000	-2.8350820000	3.9199540000
C	3.7964680000	1.3894030000	0.2592130000
H	3.9861320000	2.1781990000	-0.4602750000
C	3.6843230000	-2.5641840000	-3.2970220000
H	3.1946670000	-3.1777070000	-2.5347950000
H	4.4287420000	-3.1866820000	-3.8043060000
H	2.9307490000	-2.2594140000	-4.0254210000
C	0.2812210000	1.5282110000	3.3804550000
H	1.1491730000	2.0803170000	3.0395700000
C	5.7925160000	-1.5991620000	-1.2752640000

H	6.2198030000	-0.7453260000	-0.7416880000
H	6.6094760000	-2.1043530000	-1.8018410000
H	5.3970450000	-2.2951600000	-0.5311970000
C	5.2941030000	0.0514020000	-3.8007150000
H	4.5511920000	0.4180210000	-4.5113770000
H	6.0664420000	-0.4921590000	-4.3550190000
H	5.7718290000	0.9104700000	-3.3200330000
C	-2.0912740000	1.1274330000	3.2803350000
H	-3.0683060000	1.3735850000	2.8802040000
C	3.9407170000	1.6338620000	1.6183290000
H	4.2394870000	2.6062260000	1.9920310000
C	0.4168380000	0.5371470000	4.3400540000
H	1.3843480000	0.2987040000	4.7661280000
C	-5.1439410000	-2.0668000000	0.6613430000
H	-4.5340470000	-2.5479000000	1.4284390000
H	-6.1998560000	-2.2412860000	0.8932330000
H	-4.9607710000	-0.9889480000	0.7039680000
C	3.7052500000	0.5995660000	2.5067090000
F	3.8287370000	0.8366210000	3.8228920000
C	3.3287180000	-0.6631010000	2.0871010000
H	3.1542020000	-1.4491800000	2.8116960000
C	-4.7279400000	-4.5982890000	-1.1000220000
H	-4.4467890000	-4.9538390000	-2.0955800000
H	-5.7250510000	-4.9907610000	-0.8743750000
H	-4.0231720000	-5.0082410000	-0.3734170000
C	-1.9666480000	0.1396640000	4.2488860000
H	-2.8303280000	-0.4025310000	4.6168000000
C	-0.7117160000	-0.1474670000	4.7533640000
F	-0.5821340000	-1.1296720000	5.6654670000
Si	-1.0928860000	5.2687600000	0.1054480000

C	-2.0962120000	6.0396750000	1.4754090000
C	-1.4640420000	6.0471140000	-1.5472420000
C	0.7393470000	5.2449730000	0.4580000000
H	1.1692210000	6.2406980000	0.3080850000
H	1.2430240000	4.5479830000	-0.2190580000
H	0.9395110000	4.9275760000	1.4832670000
H	-1.1885090000	7.1064480000	-1.5492390000
H	-2.5291360000	5.9757410000	-1.7850040000
H	-0.9077830000	5.5521250000	-2.3484570000
H	-3.1660420000	5.9998720000	1.2506840000
H	-1.8197560000	7.0920620000	1.5994450000
H	-1.9210480000	5.5229300000	2.4214750000

5.4.27. (TMS-CF_3PhNCO)$_3$$P_7$ (5)
Charge = 0 Multiplicity = 1

P	0.6535860000	-0.3704530000	-0.4178530000
P	0.8761830000	-2.5686590000	-0.5197120000
P	0.9253900000	0.0352210000	-2.5817280000
P	2.7381270000	0.0808790000	0.1741510000
P	2.8591520000	-0.9842230000	-3.0499230000
P	3.9519830000	-1.2558510000	-1.1352460000
P	2.6105050000	-2.8561660000	-1.8898850000
Si	-3.0839910000	-3.1077080000	-2.6190890000
Si	3.6323360000	3.4994030000	-2.6319640000
O	2.7777800000	1.9948930000	-2.7361030000
O	-0.2084320000	-3.1522260000	-2.9540330000
O	3.4958400000	-1.9789960000	1.7488200000
N	-1.7841500000	-2.8856700000	-1.3524930000
N	2.2055860000	-0.4881220000	2.8636620000
N	0.7467060000	2.7958480000	-2.1339350000
C	-0.4940670000	-2.9177860000	-1.7966390000

C	1.5098820000	0.7202520000	2.9421310000
C	-2.0813110000	-2.8589730000	0.0408260000
C	1.4990090000	1.8210110000	-2.4417020000
C	2.7816910000	-0.8659700000	1.7971800000
C	-1.1236220000	2.9001380000	-0.6146880000
H	-0.4439470000	3.2239010000	0.1650030000
C	-0.6171460000	2.6255240000	-1.8871310000
C	-1.4985050000	2.2512990000	-2.9080750000
H	-1.1144730000	2.0833210000	-3.9079070000
C	-2.6504400000	-1.7282760000	0.6216450000
H	-2.8357410000	-0.8452930000	0.0218970000
C	-3.3363930000	2.3218740000	-1.3568280000
C	-4.7743380000	2.0473120000	-1.0604210000
C	5.3608690000	2.9598620000	-3.0736210000
H	5.7333640000	2.2167140000	-2.3627340000
H	6.0484360000	3.8114530000	-3.0624730000
H	5.3922880000	2.5148760000	-4.0721080000
C	-2.8485210000	2.0979760000	-2.6411730000
H	-3.5286330000	1.8079790000	-3.4344710000
C	-2.4731550000	2.7380840000	-0.3479580000
H	-2.8547290000	2.9374610000	0.6461950000
C	-1.8391970000	-3.9886460000	0.8219100000
H	-1.4033040000	-4.8660640000	0.3562770000
C	-2.8775710000	-1.7886700000	-3.9223100000
H	-2.8231940000	-0.7985730000	-3.4603170000
H	-3.7355870000	-1.7995040000	-4.6027020000
H	-1.9667720000	-1.9443710000	-4.5027550000
C	0.1412150000	0.7056650000	3.2195900000
H	-0.3607900000	-0.2469640000	3.3410860000
C	-4.7099080000	-2.8987080000	-1.7178060000

H	-4.8111950000	-3.5851380000	-0.8725360000
H	-5.5223030000	-3.1182720000	-2.4193620000
H	-4.8609100000	-1.8800350000	-1.3514650000
C	-2.9455150000	-4.8405700000	-3.3068130000
H	-1.9666480000	-4.9970640000	-3.7645520000
H	-3.7134420000	-5.0047260000	-4.0699870000
H	-3.0899830000	-5.5908940000	-2.5235560000
C	2.1674250000	1.9492080000	2.8140660000
H	3.2364760000	1.9633930000	2.6353260000
C	-2.1499480000	-3.9875030000	2.1746040000
H	-1.9505930000	-4.8673070000	2.7760170000
C	-0.5669800000	1.8919830000	3.3162710000
H	-1.6324640000	1.8693580000	3.5088700000
C	3.5349020000	4.1315250000	-0.8805880000
H	2.5291940000	4.4852970000	-0.6451530000
H	4.2384560000	4.9584680000	-0.7381200000
H	3.7934730000	3.3371440000	-0.1741410000
C	-2.7206550000	-2.8646600000	2.7554240000
H	-2.9690610000	-2.8553430000	3.8105700000
C	-2.9693640000	-1.7407150000	1.9735780000
C	-3.6292170000	-0.5527390000	2.6078280000
C	2.9344680000	4.6935710000	-3.8820970000
H	2.9557090000	4.2673410000	-4.8893040000
H	3.5289240000	5.6131790000	-3.8959630000
H	1.9027330000	4.9550310000	-3.6372700000
C	1.4583280000	3.1342820000	2.9144360000
H	1.9698660000	4.0831040000	2.7963060000
C	0.0865060000	3.1086240000	3.1497640000
C	-0.6668190000	4.3948460000	3.2654570000
F	-0.5744670000	4.9348930000	4.4915180000

F	-1.9766780000	4.2357040000	3.0096140000
F	-0.2051240000	5.3184610000	2.4036910000
F	-5.2170600000	2.7099280000	0.0120650000
F	-4.9805060000	0.7295950000	-0.8122600000
F	-5.5750960000	2.3593330000	-2.0906190000
F	-3.4601160000	0.5675810000	1.8943530000
F	-3.1307680000	-0.3156100000	3.8378120000
F	-4.9472390000	-0.7358100000	2.7572720000
Si	3.6186650000	-3.1450600000	3.0238660000
C	4.5068230000	-2.3849590000	4.4763040000
C	4.6371790000	-4.4712900000	2.2011330000
C	1.9056690000	-3.7528270000	3.4417510000
H	1.9631490000	-4.6751250000	4.0291570000
H	1.3466420000	-3.9628220000	2.5245940000
H	1.3511270000	-3.0074870000	4.0151400000
H	4.8197090000	-5.3037110000	2.8880620000
H	5.6071630000	-4.0814500000	1.8799990000
H	4.1258330000	-4.8674160000	1.3191800000
H	5.4887170000	-2.0032030000	4.1818850000
H	4.6611040000	-3.1348370000	5.2592930000
H	3.9266330000	-1.5614320000	4.8981640000

5.4.28. (TMS-TolylNCO)$_3$P$_7$ (6)
Charge = 0 Multiplicity = 1

P	-1.7048370000	-0.8681760000	-1.6921990000
P	-0.0076060000	0.0247150000	-0.5911820000
P	1.6163450000	-0.9904110000	-1.7001160000
P	0.0641180000	1.9504990000	-1.6775680000
P	-1.2191480000	-0.3876330000	-3.8191280000
P	0.9638510000	-0.7803960000	-3.8272310000
P	0.2124090000	1.3060530000	-3.8115530000

Si	-4.7654040000	1.9488870000	0.0270540000
Si	0.6548080000	-5.0624580000	-0.0388270000
Si	4.0839440000	3.0852210000	-0.0022370000
N	-3.5546380000	0.6102530000	-0.1910760000
O	-3.2846180000	1.2604320000	-2.3369550000
N	1.2301980000	-3.3474820000	-0.2279390000
O	0.5685310000	-3.4198050000	-2.3987960000
C	-3.2850490000	-0.3472200000	0.8327650000
C	1.2600110000	-2.1233090000	1.8929580000
H	0.1767750000	-2.1724080000	1.9226390000
C	-3.8719220000	-1.6105860000	0.7860120000
H	-4.5035120000	-1.8695470000	-0.0571600000
C	-2.9770680000	0.5173750000	-1.4248870000
C	1.0472180000	-2.7875710000	-1.4624060000
C	1.3649950000	2.9901880000	0.8541180000
C	1.9390760000	-2.6636130000	0.8050640000
N	2.3068860000	2.7492140000	-0.1907110000
O	2.6817570000	2.2725560000	-2.3665040000
C	-3.6411730000	-2.5285810000	1.8002530000
H	-4.1012600000	-3.5108260000	1.7438680000
C	1.9652580000	-1.5063960000	2.9186770000
H	1.4212490000	-1.0929480000	3.7636930000
C	-2.4676180000	-0.0194460000	1.9109460000
H	-1.9857170000	0.9521890000	1.9382860000
C	-2.8337750000	-2.2087290000	2.8932010000
C	-2.2520810000	-0.9410240000	2.9272320000
H	-1.6135260000	-0.6697780000	3.7637000000
C	3.3555930000	-1.4027420000	2.8839060000
C	3.3294470000	-2.5676350000	0.7582790000
H	3.8599960000	-2.9844650000	-0.0912910000

C	4.0237420000	-1.9389650000	1.7811080000
H	5.1061350000	-1.8682000000	1.7243300000
C	-2.6215440000	-3.1902090000	4.0093420000
H	-2.6270330000	-4.2193730000	3.6419030000
H	-3.4165780000	-3.1069310000	4.7585970000
H	-1.6713960000	-3.0161030000	4.5202920000
C	0.3568570000	2.3715720000	2.9536790000
H	0.2805260000	1.6728090000	3.7823390000
C	1.9056540000	2.3466130000	-1.4325700000
C	1.2485470000	2.1055420000	1.9223920000
H	1.8435350000	1.1985100000	1.9328180000
C	-5.3372320000	1.8167460000	1.8072060000
H	-5.7590560000	0.8326710000	2.0303790000
H	-6.1193940000	2.5631200000	1.9836940000
H	-4.5296740000	2.0037590000	2.5199130000
C	-0.3275170000	4.3851140000	1.8608730000
H	-0.9436220000	5.2788310000	1.8229760000
C	0.9841880000	-5.5048630000	1.7527460000
H	2.0393660000	-5.3925600000	2.0172210000
H	0.7095190000	-6.5529900000	1.9143990000
H	0.3983580000	-4.8955540000	2.4461820000
C	-3.9291720000	3.5923990000	-0.2689590000
H	-3.0879770000	3.7336960000	0.4164390000
H	-4.6401620000	4.4106010000	-0.1123010000
H	-3.5489450000	3.6521060000	-1.2903720000
C	0.5702560000	4.1355710000	0.8334860000
H	0.6562050000	4.8236260000	-0.0008350000
C	-0.4461460000	3.5118270000	2.9438530000
C	4.1167920000	-0.7579690000	4.0060190000
H	4.9457860000	-0.1527180000	3.6295700000

H	4.5438900000	-1.5142920000	4.6735270000
H	3.4718620000	-0.1140810000	4.6084130000
C	1.6608270000	-6.1710440000	-1.1592730000
H	1.5501650000	-5.8628690000	-2.2007500000
H	1.3242920000	-7.2090870000	-1.0675750000
H	2.7225660000	-6.1394780000	-0.8966940000
C	-1.1756380000	-5.1411300000	-0.4005640000
H	-1.7226800000	-4.4092400000	0.2014950000
H	-1.5641240000	-6.1379240000	-0.1659350000
H	-1.3720880000	-4.9268010000	-1.4524560000
C	-1.3915120000	3.8081530000	4.0721420000
H	-1.6291950000	2.9067850000	4.6418010000
H	-2.3284950000	4.2358310000	3.7056900000
H	-0.9541900000	4.5321560000	4.7683420000
C	-6.2131910000	1.6600510000	-1.1205960000
H	-5.8839820000	1.6518060000	-2.1613660000
H	-6.9570860000	2.4543610000	-0.9982220000
H	-6.7038050000	0.7066640000	-0.9023850000
C	4.5579920000	4.5245870000	-1.0975230000
H	4.3792120000	4.2840140000	-2.1471700000
H	5.6194030000	4.7626260000	-0.9710230000
H	3.9824780000	5.4196190000	-0.8425160000
C	5.0461560000	1.5327930000	-0.3930930000
H	4.9002450000	1.2396570000	-1.4343490000
H	4.7214410000	0.7026230000	0.2415190000
H	6.1151320000	1.6984410000	-0.2217130000
C	4.3138730000	3.5611910000	1.7962230000
H	3.6914480000	4.4151430000	2.0780950000
H	5.3602590000	3.8436450000	1.9553980000
H	4.0822540000	2.7385060000	2.4779080000

5.4.29. (TMS-MeOPhNCO)$_3$P$_3$ (7)
Charge = 0 Multiplicity = 1

P	-1.2529120000	1.3879490000	-1.9461140000
P	0.0234450000	-0.0167070000	-0.8105380000
P	-0.5793930000	-1.8678770000	-1.8564500000
P	1.9100450000	0.3281830000	-1.9176840000
P	-0.6800680000	0.9670090000	-4.0629150000
P	-0.5542280000	-1.2475730000	-4.0032130000
P	1.3004110000	-0.0295190000	-4.0385320000
Si	0.8252650000	5.0106960000	-0.2796350000
Si	-4.7311670000	-1.9281470000	-0.1239650000
Si	3.8521000000	-3.3232830000	-0.1366910000
N	-0.2424040000	3.5573770000	-0.4904850000
O	0.4579590000	3.4035750000	-2.6320420000
N	-2.9323850000	-2.0497460000	-0.3465680000
O	-3.1984050000	-1.4704640000	-2.5147670000
C	-1.1383890000	3.1067760000	0.5252890000
C	-1.7143900000	-1.6869620000	1.7450350000
H	-2.0606810000	-0.6593940000	1.7551000000
C	-2.4963490000	3.3938100000	0.4417820000
H	-2.8681060000	3.9436690000	-0.4162040000
C	-0.1997140000	2.9533810000	-1.7150770000
C	-2.4577340000	-1.7545400000	-1.5929100000
C	3.2088270000	-0.6555820000	0.6387400000
C	-2.0652670000	-2.5154710000	0.6878210000
N	3.1661280000	-1.6564580000	-0.3773940000
O	2.8680790000	-2.1283710000	-2.5504570000
C	-3.3826880000	2.9753710000	1.4277450000
H	-4.4354790000	3.2062280000	1.3246650000
C	-0.9106890000	-2.1507470000	2.7821130000
H	-0.6624090000	-1.4812940000	3.5966120000

C	-0.6660130000	2.4040580000	1.6347450000
H	0.3887170000	2.1627450000	1.7054760000
C	-2.9027960000	2.2679170000	2.5291480000
C	-1.5357390000	1.9953230000	2.6283660000
H	-1.1803830000	1.4554280000	3.4992400000
C	-0.4342750000	-3.4604890000	2.7567860000
C	-1.5979900000	-3.8331040000	0.6799560000
H	-1.8732730000	-4.4873450000	-0.1403970000
C	-0.7850690000	-4.2998050000	1.6951730000
H	-0.4115450000	-5.3174680000	1.6953250000
O	-3.6737480000	1.7996100000	3.5375600000
C	-5.0568630000	2.0770100000	3.4958280000
C	2.3569080000	0.2906780000	2.6869320000
H	1.6517560000	0.2942850000	3.5112240000
C	2.7263170000	-1.3638460000	-1.6347700000
C	2.2975860000	-0.6708990000	1.6959330000
H	1.5225860000	-1.4288290000	1.7211220000
C	0.6018280000	5.5401060000	1.5046160000
H	-0.4403860000	5.7793240000	1.7339610000
H	1.1981880000	6.4414880000	1.6837060000
H	0.9319770000	4.7737750000	2.2114450000
C	4.2438070000	1.3173680000	1.5922170000
H	5.0063380000	2.0835440000	1.5295810000
C	-5.0383390000	-2.3182070000	1.6835710000
H	-4.6486290000	-3.3008800000	1.9635410000
H	-6.1184540000	-2.3229530000	1.8670940000
H	-4.5878800000	-1.5777880000	2.3505320000
C	2.6013290000	4.5205120000	-0.5913710000
H	2.8856400000	3.6655810000	0.0299170000
H	3.2704250000	5.3559550000	-0.3581880000

H	2.7464020000	4.2378750000	-1.6355480000
C	4.1815380000	0.3378740000	0.6066760000
H	4.8946370000	0.3520070000	-0.2107660000
C	3.3241110000	1.2983740000	2.6399000000
O	0.3711540000	-4.0033650000	3.6985430000
C	0.7271390000	-3.2058380000	4.8066460000
C	-5.5730590000	-3.2011590000	-1.2042840000
H	-5.2877000000	-3.0633780000	-2.2491330000
H	-6.6611290000	-3.1031710000	-1.1285750000
H	-5.3067300000	-4.2176760000	-0.8994550000
C	-5.2851770000	-0.1854660000	-0.5091810000
H	-4.7301830000	0.5407940000	0.0925690000
H	-6.3525340000	-0.0715240000	-0.2906930000
H	-5.1165900000	0.0505020000	-1.5614050000
O	3.2782410000	2.2072280000	3.6408680000
C	4.2445430000	3.2360380000	3.6455450000
C	0.2493020000	6.3731570000	-1.4236340000
H	0.3209130000	6.0536470000	-2.4651550000
H	0.8671980000	7.2675420000	-1.2911750000
H	-0.7885060000	6.6503660000	-1.2153620000
C	5.4583740000	-3.4519780000	-1.0862820000
H	5.2820210000	-3.2862770000	-2.1513570000
H	5.8992490000	-4.4462630000	-0.9598130000
H	6.1874160000	-2.7153190000	-0.7351430000
C	2.5986150000	-4.5992970000	-0.6705990000
H	2.3714180000	-4.5056800000	-1.7336710000
H	1.6663500000	-4.4840580000	-0.1099770000
H	2.9891290000	-5.6050290000	-0.4814290000
C	4.1707270000	-3.4751200000	1.7041740000
H	4.7791230000	-2.6548520000	2.0944770000

H	4.7092190000	-4.4104660000	1.8919800000
H	3.2378700000	-3.5126130000	2.2740130000
H	4.0309720000	3.8439990000	4.5235310000
H	4.1708950000	3.8608150000	2.7478270000
H	5.2602630000	2.8315200000	3.7249820000
H	-5.4808360000	1.6239320000	4.3908910000
H	-5.5292760000	1.6358560000	2.6103850000
H	-5.2496260000	3.1560070000	3.5115710000
H	1.3583800000	-3.8315500000	5.4359930000
H	-0.1559050000	-2.8946830000	5.3769180000
H	1.2947920000	-2.3194660000	4.4992490000

5.4.30. (TMS-PhNCS)$_3$P$_7$ (8)
Charge = 0 Multiplicity = 1

P	-0.0725230000	-0.0234480000	-0.0915740000
P	0.9298150000	1.4453720000	-1.4091600000
P	-0.5941050000	-1.4682300000	-1.6854470000
P	-2.0418700000	0.9914530000	-0.0103150000
P	-1.6802300000	-0.2196790000	-3.1921770000
P	-2.4115970000	1.5883170000	-2.1314440000
P	-0.4469930000	1.6222800000	-3.1608270000
Si	4.5071500000	-1.0726120000	-2.5312690000
Si	-4.7385100000	-2.7345950000	-1.0326880000
O	-3.1921120000	-2.1344010000	-1.5268730000
O	2.1270780000	0.2161260000	-3.5083120000
O	-1.6293990000	3.6458150000	-0.1643540000
N	3.2677910000	-0.1254520000	-1.5892660000
N	-1.0609170000	2.8429690000	1.8724920000
N	-2.0085410000	-3.0515440000	0.1686280000
C	2.2227860000	0.3745240000	-2.3055570000
C	-0.9654120000	1.8163910000	2.8199830000

C	3.4005550000	0.1314070000	-0.1930920000
C	-2.0563790000	-2.3226320000	-0.8678670000
C	-1.4923970000	2.6462510000	0.6964640000
C	-0.6818850000	-2.8970080000	2.1748900000
H	-1.4887290000	-2.3357550000	2.6314620000
C	-0.8138940000	-3.3163200000	0.8487950000
C	0.2164900000	-4.0601880000	0.2663740000
H	0.1049640000	-4.4216980000	-0.7498590000
C	3.1697450000	-0.8885300000	0.7282180000
H	2.8443760000	-1.8617300000	0.3776210000
C	1.4842890000	-3.8843620000	2.2824600000
F	2.6172680000	-4.1283850000	2.9685730000
C	-5.8419670000	-1.9977980000	-2.3418170000
H	-5.7879630000	-0.9054240000	-2.3288050000
H	-6.8853500000	-2.2865030000	-2.1803960000
H	-5.5487720000	-2.3362220000	-3.3395550000
C	1.3728650000	-4.3443460000	0.9828820000
H	2.1789190000	-4.9246490000	0.5479410000
C	0.4708780000	-3.1688910000	2.8946540000
H	0.5819910000	-2.8350820000	3.9199540000
C	3.7964680000	1.3894030000	0.2592130000
H	3.9861320000	2.1781990000	-0.4602750000
C	3.6843230000	-2.5641840000	-3.2970220000
H	3.1946670000	-3.1777070000	-2.5347950000
H	4.4287420000	-3.1866820000	-3.8043060000
H	2.9307490000	-2.2594140000	-4.0254210000
C	0.2812210000	1.5282110000	3.3804550000
H	1.1491730000	2.0803170000	3.0395700000
C	5.7925160000	-1.5991620000	-1.2752640000
H	6.2198030000	-0.7453260000	-0.7416880000

H	6.6094760000	-2.1043530000	-1.8018410000
H	5.3970450000	-2.2951600000	-0.5311970000
C	5.2941030000	0.0514020000	-3.8007150000
H	4.5511920000	0.4180210000	-4.5113770000
H	6.0664420000	-0.4921590000	-4.3550190000
H	5.7718290000	0.9104700000	-3.3200330000
C	-2.0912740000	1.1274330000	3.2803350000
H	-3.0683060000	1.3735850000	2.8802040000
C	3.9407170000	1.6338620000	1.6183290000
H	4.2394870000	2.6062260000	1.9920310000
C	0.4168380000	0.5371470000	4.3400540000
H	1.3843480000	0.2987040000	4.7661280000
C	-5.1439410000	-2.0668000000	0.6613430000
H	-4.5340470000	-2.5479000000	1.4284390000
H	-6.1998560000	-2.2412860000	0.8932330000
H	-4.9607710000	-0.9889480000	0.7039680000
C	3.7052500000	0.5995660000	2.5067090000
F	3.8287370000	0.8366210000	3.8228920000
C	3.3287180000	-0.6631010000	2.0871010000
H	3.1542020000	-1.4491800000	2.8116960000
C	-4.7279400000	-4.5982890000	-1.1000220000
H	-4.4467890000	-4.9538390000	-2.0955800000
H	-5.7250510000	-4.9907610000	-0.8743750000
H	-4.0231720000	-5.0082410000	-0.3734170000
C	-1.9666480000	0.1396640000	4.2488860000
H	-2.8303280000	-0.4025310000	4.6168000000
C	-0.7117160000	-0.1474670000	4.7533640000
F	-0.5821340000	-1.1296720000	5.6654670000
Si	-1.0928860000	5.2687600000	0.1054480000
C	-2.0962120000	6.0396750000	1.4754090000

C	-1.4640420000	6.0471140000	-1.5472420000
C	0.7393470000	5.2449730000	0.4580000000
H	1.1692210000	6.2406980000	0.3080850000
H	1.2430240000	4.5479830000	-0.2190580000
H	0.9395110000	4.9275760000	1.4832670000
H	-1.1885090000	7.1064480000	-1.5492390000
H	-2.5291360000	5.9757410000	-1.7850040000
H	-0.9077830000	5.5521250000	-2.3484570000
H	-3.1660420000	5.9998720000	1.2506840000
H	-1.8197560000	7.0920620000	1.5994450000
H	-1.9210480000	5.5229300000	2.4214750000

5.4.31. PhNCO
Charge = 0 Multiplicity = 1

N	2.2513120000	2.0387560000	-0.2793680000
C	2.6120310000	1.6878220000	-1.5761710000
C	2.4874340000	1.7327300000	0.8581420000
C	2.0262230000	2.3789690000	-2.6350130000
H	1.3118240000	3.1639660000	-2.4160580000
C	3.5353120000	0.6723720000	-1.8331570000
H	3.9893520000	0.1364160000	-1.0060850000
C	2.3630630000	2.0549090000	-3.9415040000
H	1.9031580000	2.5970300000	-4.7608540000
C	3.8641810000	0.3573110000	-3.1432020000
H	4.5822380000	-0.4326720000	-3.3364340000
C	3.2814730000	1.0449030000	-4.2020790000
H	3.5423940000	0.7942600000	-5.2243090000
O	2.6330040000	1.5272270000	1.9960930000

5.4.32. 4-Br(C_6H_4)NCO
Charge = 0 Multiplicity = 1

N	3.0474250000	1.7950080000	-0.3921180000
C	3.0032700000	1.4147540000	-1.7269350000
C	2.3049480000	2.0201040000	0.5262130000
O	1.6907390000	2.2618480000	1.4855360000
C	3.0049920000	0.6587220000	-4.3954630000
C	1.7978900000	0.7933500000	-3.7215340000
C	1.7979160000	1.1712230000	-2.3880120000
C	4.2067250000	1.2759360000	-2.4155850000
C	4.2105590000	0.8981130000	-3.7495890000
Br	3.0043370000	0.1423500000	-6.2166260000
H	0.8625330000	0.6045570000	-4.2341540000
H	0.8583730000	1.2781130000	-1.8562360000
H	5.1367240000	1.4673710000	-1.8935090000
H	5.1465700000	0.7905510000	-4.2839880000

5.4.33. 4-F(C_6H_4)NCO
Charge = 0 Multiplicity = 1

N	3.0441160000	1.7837790000	-0.4299440000
C	3.0011930000	1.4038370000	-1.7661750000
C	2.3111750000	2.0090280000	0.4944250000
O	1.7040770000	2.2521010000	1.4589770000
C	3.0038900000	0.6512030000	-4.4226050000
C	1.7950790000	0.7825650000	-3.7610950000
C	1.7953460000	1.1606940000	-2.4272020000
C	4.2056940000	1.2651860000	-2.4538660000
C	4.2104900000	0.8871910000	-3.7881950000
F	3.0040200000	0.2867250000	-5.7122540000
H	0.8701320000	0.5897760000	-4.2913800000
H	0.8569760000	1.2683470000	-1.8938820000
H	5.1342770000	1.4569670000	-1.9297100000
H	5.1365360000	0.7746020000	-4.3390940000

5.4.34. 4-$CF_3(C_6H_4)NCO$
Charge = 0 Multiplicity = 1

N	3.0526980000	1.7775560000	-0.4065550000
C	3.0061560000	1.4056460000	-1.7422040000
C	2.3032260000	2.0038180000	0.5073120000
O	1.6846130000	2.2462420000	1.4622020000
C	3.0040510000	0.6681340000	-4.4221000000
C	1.8008530000	0.8108760000	-3.7390430000
C	1.7973250000	1.1793050000	-2.4046650000
C	4.2114700000	1.2628640000	-2.4287550000
C	4.2079410000	0.8945570000	-3.7633870000
C	3.0060340000	0.2186920000	-5.8506360000
H	0.8637430000	0.6420160000	-4.2564310000
H	0.8598710000	1.2957970000	-1.8716560000
H	5.1402670000	1.4471150000	-1.9025120000
H	5.1441770000	0.7912590000	-4.2994930000
F	4.0391080000	0.7316050000	-6.5346570000
F	1.8823660000	0.5758750000	-6.4893410000
F	3.1011000000	-1.1183570000	-5.9500790000

5.4.35. PhNCS
Charge = 0 Multiplicity = 1

N	2.9228870000	1.6559100000	-0.4900180000
C	2.9686240000	1.4334420000	-1.8470390000
C	2.3705080000	1.7925560000	0.5524980000
S	1.7217790000	1.9865740000	1.9780370000
C	3.0877210000	0.9859860000	-4.5872730000
C	1.8580010000	1.0129850000	-3.9375520000
C	1.7892220000	1.2354780000	-2.5712090000
C	4.2048300000	1.4073770000	-2.4936180000

C	4.2567690000	1.1835480000	-3.8612620000
H	3.1339000000	0.8113790000	-5.6564890000
H	0.9424350000	0.8594810000	-4.4986850000
H	0.8363080000	1.2585640000	-2.0547040000
H	5.1065990000	1.5631620000	-1.9132200000
H	5.2184180000	1.1635590000	-4.3624680000

5.4.36. p-tolylNCO
Charge = 0 Multiplicity = 1

N	3.0428230000	1.7853330000	-0.4136980000
C	3.0023700000	1.4053640000	-1.7513010000
C	2.3083220000	2.0104610000	0.5090230000
O	1.7000850000	2.2533190000	1.4739560000
C	3.0055740000	0.6452010000	-4.4568990000
C	1.8108720000	0.7907930000	-3.7506060000
C	1.7994150000	1.1644440000	-2.4159970000
C	4.2039400000	1.2688240000	-2.4415300000
C	4.1972660000	0.8941620000	-3.7765850000
C	3.0077060000	0.2086440000	-5.8931670000
H	0.8663990000	0.6100530000	-4.2555730000
H	0.8593910000	1.2747700000	-1.8850500000
H	5.1340190000	1.4631880000	-1.9200060000
H	5.1424510000	0.7951650000	-4.3022230000
H	3.8755820000	0.6020850000	-6.4277410000
H	2.1077410000	0.5450180000	-6.4136620000
H	3.0420430000	-0.8838230000	-5.9709390000

5.4.37. 4-MeO(C_6H_4)NCO
Charge = 0 Multiplicity = 1

N	2.2771760000	1.9967380000	-0.1976060000
C	2.6288180000	1.7249100000	-1.5162290000

C	2.4644500000	1.5919340000	0.9165300000
C	2.1212520000	2.5336100000	-2.5249030000
H	1.4652390000	3.3542530000	-2.2587370000
C	3.4759810000	0.6642710000	-1.8546860000
H	3.8781690000	0.0260530000	-1.0747300000
C	2.4460820000	2.2988340000	-3.8561580000
H	2.0341950000	2.9469180000	-4.6193950000
C	3.8012010000	0.4275730000	-3.1750000000
H	4.4563050000	-0.3906360000	-3.4514380000
C	3.2901020000	1.2418190000	-4.1895250000
O	3.6698350000	0.9249820000	-5.4490050000
O	2.5708720000	1.2917660000	2.0392170000
C	3.1790980000	1.7183120000	-6.5052130000
H	3.5006620000	2.7622770000	-6.4082130000
H	2.0846250000	1.6801770000	-6.5631650000
H	3.5999380000	1.2982100000	-7.4177440000

6. References

[1] M. Cicač-Hudi, J. Bender, S. H. Schlindwein, M. Bispinghoff, M. Nieger, H. Grützmacher, D. Gudat, *Eur. J. Inorg. Chem.* **2016**, *2016*, 649–658.

[2] C. Mujica, D. Weber, H.-G. v. Schnering, *Z. Naturforsch.* **1986**, *41b*, 991–999.

[3] a) G. M. Sheldrick, *Acta. Cryst.* **2015**, *A71*, 3–8; b) G. M. Sheldrick, *Acta. Cryst.* **2015**, *C71*, 3–8; c) O. V. Dolomanov, L. J. Bourhis, R. J. Gildea, J. A. K. Howard, H. Puschmann, *J. Appl. Cryst.* **2009**, *42*, 339–341.

[4] a) P. Hohenberg, W. Kohn, *J. Phys. Rev. B* **1964**, *136*, B864; b) W. Kohn, L. Sham, *J. Phys. Rev.* **1965**, *140*, A1133; c) C. Peng, P. Y. Ayala, H. B. Schlegel, M. J. J. Frisch, *Comp. Chem.* **1996**, *17*, 49; d) C. Peng, H. B. Schlegel, *Israel J. of Chem.* **1993**, *33*, 449.

[5] P. H. M. Budzelaar, gNMR: NMR simulation software version 5.09, **2006**.

[6] P. Noblet, A. Dransfeld, R. Fischer, M. Flock, K. Hassler, *J. Organomet. Chem.* **2011**, *696*, 652–660.

[7] K. Christe, D. Dixon, D. McLemore, W. Wilson, J. Sheehy, J. Boatz, *J. Fluor. Chem.* **2000**, *101*, 151–153.

[8] P. Erdmann, J. Leitner, J. Schwarz, L. Greb, *ChemPhysChem* **2020**, *21*, 987–994.
